# Anti-sporozoite monoclonal antibody for malaria prevention: secondary efficacy outcome of a phase 2 randomized trial

**DOI:** 10.1038/s41591-025-03739-y

**Published:** 2025-06-03

**Authors:** Jeff Skinner, Kassoum Kayentao, Aissata Ongoiba, Sara A. Healy, Zonghui Hu, Anne C. Preston, Amadou Niangaly, Philipp Schwabl, Hamidou Cisse, Safiatou Doumbo, Didier Doumtabe, Abdrahamane Traore, Shanping Li, Mary E. Peterson, Annette M. Seilie, Chris Chavtur, Weston Staubus, Ming Chang, Katrina Kelley, Hamadi Traore, Adama Djiguiba, Mamadou Keita, Adama Ouattara, M’Bouye Doucoure, Mohamed Keita, Djelika Diarra, Mamadou Sylla, Dramane Diakite, Mamadou Konate, Siriman Traore, Amatigué Zéguimé, Amagana Dolo, Daniel E. Neafsey, Sean C. Murphy, Boubacar Traore, Robert A. Seder, Peter D. Crompton

**Affiliations:** 1 Malaria Infection Biology and Immunity Section, Laboratory of Immunogenetics, Division of Intramural Research, NIAID, NIH, Rockville, MD USA; 2 Malaria Research and Training Centre, Mali International Center of Excellence in Research, University of Sciences, Techniques and Technologies of Bamako, Bamako, Mali; 3 Biostatistics Research Branch, Division of Clinical Research, NIAID, NIH, Rockville, MD USA; 4 Department of Immunology and Infectious Diseases, Harvard T.H. Chan School of Public Health, Boston, MA USA; 5 Department of Laboratory Medicine and Pathology and Center for Emerging and Re-emerging Infectious Diseases, University of Washington, Seattle, WA USA; 6 Department of Laboratories, Seattle Children’s Hospital, Seattle, WA USA; 7 Cellular Immunology Section, Vaccine Research Center, NIAID, NIH, Bethesda, MD USA

**Keywords:** Randomized controlled trials, Malaria, Drug development

## Abstract

CIS43LS is a long-acting monoclonal antibody specific for the *Plasmodium falciparum* circumsporozoite protein expressed on sporozoites. We previously reported that CIS43LS is protective against *P.* *falciparum* infection as detected by thick blood smear (TBS; primary endpoint) in a phase 2 double-blind randomized trial involving 330 healthy Malian adults receiving placebo or a single intravenous infusion of 10 mg kg^−1^ or 40 mg kg^−1^ of CIS43LS (1:1:1). At enrollment, all participants received artemether–lumefantrine to clear possible *P.* *falciparum* infection. Although TBS examination is the standard assay to assess efficacy in malaria vaccines trials in endemic areas, it has poor analytical sensitivity; therefore, it remained unknown whether CIS43LS had achieved sterile protection against infection. Here we report the prespecified secondary efficacy endpoint that used a *Plasmodium* 18S rRNA quantitative reverse transcription–PCR (qRT–PCR) assay that is ~2,000-fold more sensitive than TBS. We analyzed 5,015 dried blood spots collected before CIS43LS or placebo administration and biweekly thereafter over a 6-month malaria season. At 6 months, efficacy of CIS43LS against qRT–PCR-detected infection assessed in a time-to-event analysis was 87.4% for 40 mg kg^−1^ (adjusted 95% confidence interval (CI), 79.5–92.3; *P* < 0.001) and 77.0% for 10 mg kg^−1^ (adjusted 95% CI, 65.0–84.0; *P* < 0.001) versus placebo. A post hoc analysis with a gametocyte mRNA-specific qRT–PCR assay showed 6-month efficacy against gametocytemia of 87.7% for 40 mg kg^−1^ (adjusted 95% CI, 75.6–93.8; *P* < 0.001) and 73.0% for 10 mg kg^−1^ (adjusted 95% CI, 54.0–84.0; *P* < 0.001), versus placebo. These data indicate that a single dose of anti-sporozoite monoclonal antibodies can achieve durable, sterile protection against *P.* *falciparum* infection, underscoring their potential to reduce malaria disease burden and transmission. ClinicalTrials.gov identifier: NCT04329104.

## Abstract (web summary)

A single dose of the anti-sporozoite monoclonal antibody CIS43LS can achieve durable, sterile protection against *Plasmodium falciparum* infection.

## Main

The mosquito-transmitted parasite *Plasmodium falciparum* causes more than 200 million cases of malaria each year, resulting in approximately 600,000 deaths, mostly among children in Africa^1^. In recent years, progress against malaria has slowed despite widespread use of insecticide-based interventions and antimalarial drugs^1^, underscoring the need to develop new countermeasures.

Malaria infection begins when mosquitoes inject sporozoites into the skin and blood^2^. Within minutes to hours^3^, sporozoites invade hepatocytes, where they replicate and differentiate into merozoites. After about a week, merozoites exit the liver and begin asexual replication cycles in erythrocytes that culminate in disease, while a small percentage differentiate into nonpathogenic sexual forms called gametocytes that are transmitted to mosquitoes. Thus, an intervention that completely blocks sporozoite infection would prevent all subsequent stages that cause disease and onward transmission.

The *P.* *falciparum* circumsporozoite protein (PfCSP) is the dominant protein expressed on the surface of sporozoites and is required for hepatocyte invasion, making it an ideal target for neutralization^4,5^. The RTS,S/AS01 and R21/Matrix-M vaccines are comprised of truncated portions of PfCSP and three doses are recommended for the prevention of malaria disease in children^6^. In phase 3 trials of RTS,S/AS01 and R21/Matrix-M, the primary efficacy endpoint was symptomatic blood-stage infection, as detected by microscopic examination of thick blood smears (TBS)^7,8^. Because blood-stage infections can be asymptomatic^9^, the efficacy of RTS,S/AS01 and R21/Matrix-M in preventing infection in these trials is uncertain, although in the R21/Matrix-M trial the cross-sectional prevalence of asymptomatic parasitemia by TBS was lower in the vaccine group 12 and 18 months after vaccination^8^.

A recent pediatric phase 2b trial in Ghana and Kenya of various RTS,S/AS01 regimens found that its efficacy against genotypically detected infection, assessed through monthly surveillance, was 25–43% over 12–20 months^10^. For adults in endemic areas, there are no efficacy data so far for R21/Matrix-M, and efficacy data for RTS,S is limited. A trial of RTS,S/AS02 in The Gambia that assessed infection risk in adults over 15 weeks through weekly TBS microscopy, demonstrated efficacy against infection of 34%, with efficacy falling to 0% in the last 6 weeks of the trial^11^. Additionally, a phase 2b trial of RTS,S/AS01B and RTS,S/AS02A in Kenyan adults showed no statistically significant efficacy against *P.* *falciparum* infection^12^. Therefore, new tools are needed that block sporozoite infection with high efficacy in all age groups.

CIS43LS is an extended half-life monoclonal antibody (mAb) that targets a highly conserved epitope of PfCSP^13–15^. In a phase 2 trial of adults in Mali (NCT04329104), a single intravenous (IV) infusion of CIS43LS at a dose of 40 mg kg^−1^ or 10 mg kg^−1^ was safe and provided protective efficacy against *P.* *falciparum* infection of 88.2% and 75.0% over 6 months, respectively, as detected by scheduled biweekly TBS examinations—the primary endpoint of the trial^16^. Although TBS examination is historically the standard diagnostic assay to assess efficacy in trials of malaria vaccines and drugs in endemic areas, TBS has poor analytical sensitivity, with a limit of detection (LoD) of ~40,000 parasites per milliliter of blood^17,18^. Therefore, it remained unknown whether CIS43LS had achieved complete protection against sporozoite infection that prevented the subsequent development of submicroscopic infection with disease-causing asexual blood-stage parasites and gametocytes that are responsible for onward transmission.

Here, we present the results of a prespecified analysis of this trial in which the secondary efficacy endpoint was *P.* *falciparum* infection detected by a highly sensitive *Plasmodium* 18S rRNA quantitative reverse transcription–PCR (18S qRT–PCR) assay that detects asexual blood-stage parasites as well as gametocytes with a LoD of ~20 parasites per milliliter of blood^19–21^. With this assay, we analyzed dried blood spots (DBS) collected from trial participants at baseline before the administration of CIS43LS or placebo and at least every 2 weeks thereafter for 6 months. All participants received a treatment course of artemether–lumefantrine (AL) before the administration of CIS43LS or placebo to eliminate possible *P.* *falciparum* infections so that the infection endpoint could be evaluated. Additionally, because the 18S qRT–PCR assay does not distinguish between asexual blood-stage parasites and gametocytes, we conducted a post hoc analysis with a *P.* *falciparum* gametocyte mRNA-specific qRT–PCR assay with a LoD of ~300 gametocytes per milliliter of blood^22^ to determine the rate of gametocyte clearance after AL and the subsequent risk of new gametocytemia following administration of CIS43LS or placebo. Although CIS43LS does not directly target gametocytes or prevent their transmission to mosquitoes, we hypothesized that, after clearance of baseline gametocytemia by AL, CIS43LS would reduce the subsequent risk of gametocytemia by preventing liver stage infection.

## Results

### Study design and participant characteristics

**Fig. 1:**
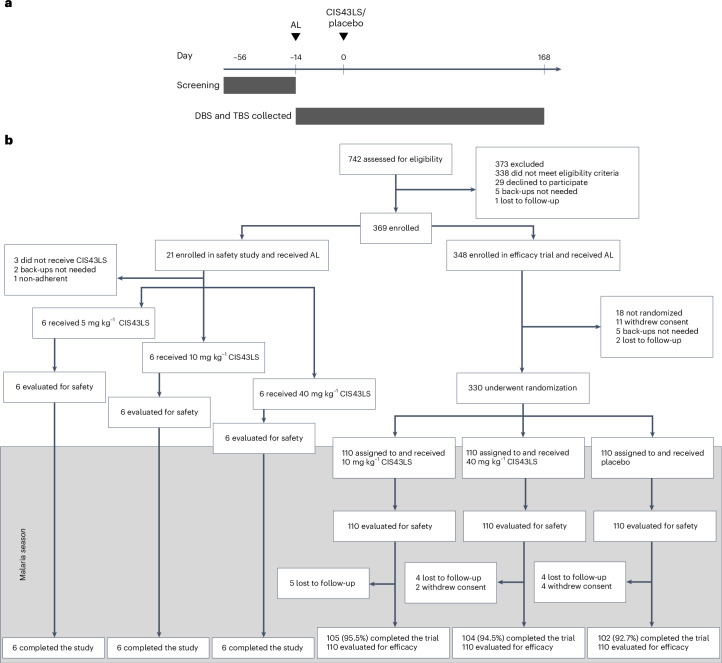
Efficacy trial design and CONSORT diagram. **a**, In the efficacy trial that began before the 6-month malaria transmission season, participants received a standard 3-day treatment course of AL at enrollment (day −14 ± 7). On day 0 they were randomized and received a single IV infusion of normal saline placebo (*n* = 110), or CIS43LS at a dose of 10 mg kg^−1^ (*n* = 110) or 40 mg kg^−1^ (*n* = 110). DBS and TBS were obtained for 18S qRT–PCR and microscopic examination, respectively, at enrollment and on days 0, 1, 3, 7, 14, 21 and 28 and every 2 weeks thereafter for 24 weeks and during unscheduled illness visits when malaria was suspected. DBS from study day 1 were not analyzed by 18S qRT–PCR. **b**, Screening, enrollment, randomization and follow-up for the initial safety study and efficacy trial.

Between 15 February and 26 July 2021, 742 adults aged 18–55 years were assessed for eligibility (Fig. 1). Of these, 373 were excluded because they did not meet eligibility criteria (*n* = 338) or for other reasons (*n* = 35). The remaining 369 participants were enrolled in the initial safety study or the efficacy trial. The results of the safety study have been published^16^. In the efficacy trial, 348 participants were enrolled and received AL (day −14 ± 7). Between 5 May and 6 August 2021, 330 of these participants were randomized and received a single IV infusion of placebo (*n* = 110) or CIS43LS at a dose of 10 mg kg^−1^ (*n* = 110) or 40 mg kg^−1^ (*n* = 110). Eighteen participants were not randomized because they withdrew consent (*n* = 11) or for other reasons (*n* = 7). The median interval between AL administration and CIS43LS or placebo administration (day 0) was 9 days (range 7–15 days) across trial groups. For the remainder of the trial, asymptomatic *P.* *falciparum* infections were not tested for in real time and therefore not treated, in accordance with national guidelines in Mali. All the participants in whom symptomatic malaria developed during the trial were provided standard treatment. The study population consisted of healthy men (57%) and nonpregnant women (43%) with a median age of 35 years (range 18–54 years). Additional inclusion and exclusion criteria can be found in the trial protocol (Supplementary Information). Baseline characteristics were similar across trial groups and are summarized in Table 1. At the last scheduled study visit (day 168 after infusion), 311 participants (94.2%) completed follow-up: 105 (95.5%) who received 10 mg kg^−1^, 104 (94.5%) who received 40 mg kg^−1^ and 102 (92.7%) who received placebo.

**Table 1:** Baseline characteristics of study participants

Characteristic		10 mg kg^−1^ (*N* = 110) *n* (%)	40 mg kg^−1^ (*N* = 110) *n* (%)	Placebo (*N* = 110) *n* (%)
Age (years)	Mean (s.d.)	33.8 (9.3)	34.4 (9.7)	34.1 (9.7)
	Median	34	35	35
	Minimum, maximum	18, 54	18, 53	18, 53
Sex	Male	59 (53.6)	63 (57.3)	66 (60)
	Female	51 (46.4)	47 (42.7)	44 (40)
Ethnicity	Bambara	107 (97.3)	103 (93.6)	107 (97.3)
	Peuth	1 (0.9)	5 (4.5)	0
	Sarakole	2 (1.8)	2 (1.8)	3 (2.7)
Weight (kg)	Mean (s.d.)	64.0 (9.9)	62.3 (11.4)	63.8 (11.4)
	Median	63.5	61.5	63
	Minimum, maximum	44, 95	43, 101	46, 114
Site	Kalifabougou	67 (60.9)	67 (60.9)	66 (60)
	Torodo	43 (39.1)	43 (39.1)	44 (40)
*Plasmodium* detected on TBS at enrollment	Any species	15 (13.6)	9 (8.2)	8 (7.3)
	*P.* *falciparum*	14 (12.7)	8 (7.3)	8 (7.3)
	*Plasmodium* *malariae*	0	1 (0.9)	0
	*Plasmodium ovale*	1 (0.9)	0	0
Interval between administration of AL and CIS43LS	Mean (s.d.)	9.1 (1.7)	9.1 (1.6)	9.2 (1.7)
	Median	9	9	9
	Minimum, maximum	7, 15	7, 14	7, 14
Hemoglobin genotype	AA	98 (89.1)	93 (84.5)	92 (83.6)
	AS	7 (6.4)	7 (6.4)	10 (9.1)
	AC	3 (2.7)	9 (8.2)	8 (7.3)
	CC	0	1 (0.9)	0
	SC	2 (1.8)	0	0

Paired DBS and TBS were collected for 18S qRT–PCR and microscopic examination, respectively, at enrollment (before AL administration) and on days 0, 1, 3, 7, 14, 21 and 28 and every 2 weeks thereafter for 24 weeks (day 168) as well as during unscheduled illness visits when malaria was suspected. We carried out 18S qRT–PCR consecutively on DBS collected at all scheduled and unscheduled visits from enrollment through day 70 after CIS43LS or placebo administration, except DBS collected on study day 1, which were not tested. For participants who were 18S qRT–PCR-negative from days 28–70, 18S qRT–PCR testing continued consecutively through all subsequent scheduled and unscheduled visits until either the last scheduled visit (day 168) or upon reaching a positive result of >100,000 parasites ml^−1^ by 18S qRT–PCR.

### Comparison of *Plasmodium* 18S qRT–PCR and TBS microscopy

**Fig. 2:**
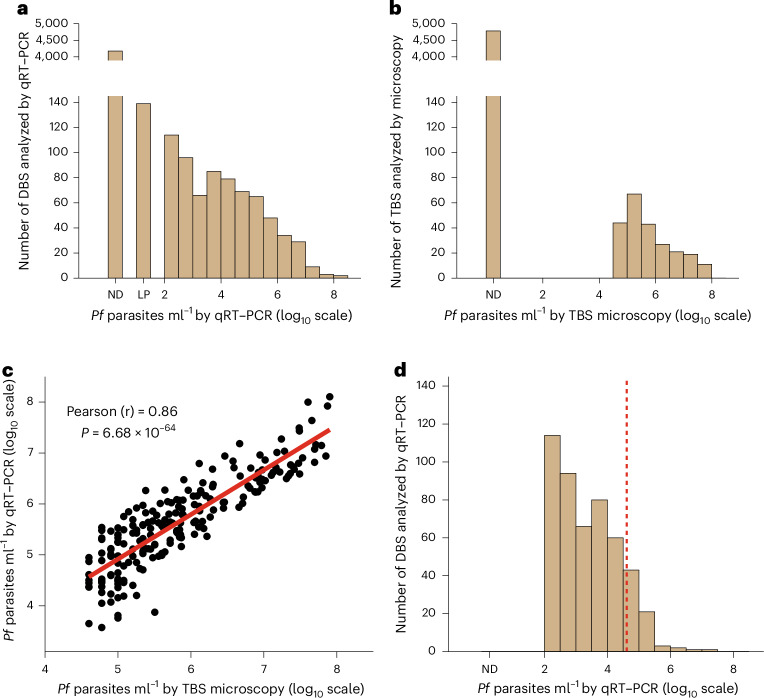
Distribution of *P.* *falciparum* parasitemia as detected by 18S qRT–PCR and TBS examination and their correlation. **a**,**b**, A total of 5,015 paired DBS and TBS collected from 330 participants over the 6-month study period were analyzed by 18S qRT–PCR and TBS microscopy, respectively. 18S qRT–PCR results were plotted as not detected (ND), low positive (LP) or quantitatively positive with a numeric value of ≥2.0 log_10_ parasites ml^−1^; histograms show the qualitative and quantitative results of 18S qRT–PCR (**a**) and TBS microscopy (**b**). **c**, Pearson correlation of *P.* *falciparum* parasite density by 18S qRT–PCR versus TBS among 214 paired samples that were quantitatively positive by both assays. *P* value for the Pearson correlation was determined by two-sided *t*-test. The solid red line shows a linear regression fit. **d**, Distribution of LP and quantitatively positive 18S qRT–PCR results among 486 samples that were negative by TBS. The red dashed vertical line indicates the approximate LoD of TBS microscopy (~40,000 parasites ml^−1^). *Pf*, *P.* *falciparum*.

**Extended Data Fig. 1:**
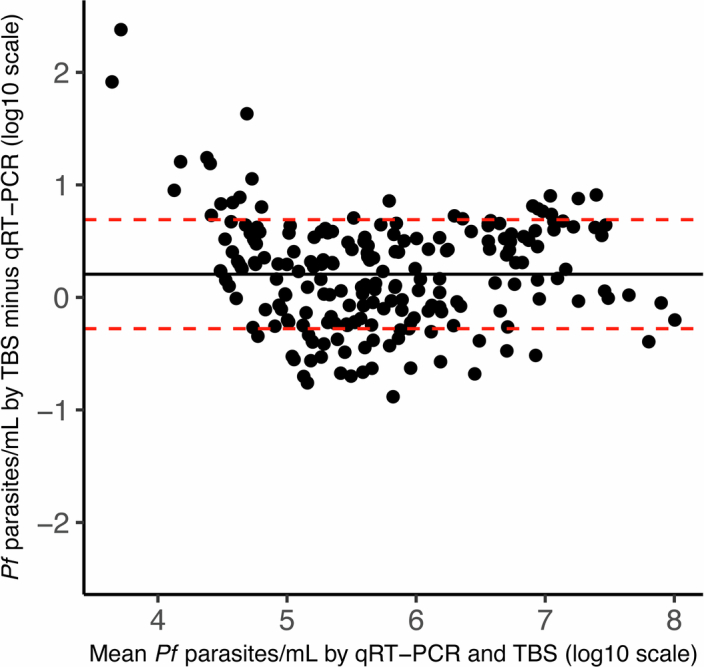
Assessment of bias between 18S qRT-PCR- and TBS-based parasite densities. A Bland-Altman plot demonstrates minimal bias (0.21 log_10_ parasites/mL) between log_10_ transformed *Plasmodium* 18S qRT-PCR- and TBS-based parasite densities. There was no density dependence to the bias (P = 0.19), as determined by simple linear regression with a two-sided P value. The solid black line represents the difference between log_10_ measurements (bias) and the dashed red lines represent mean bias ± 1 SD.

**Extended Data Fig. 2:**
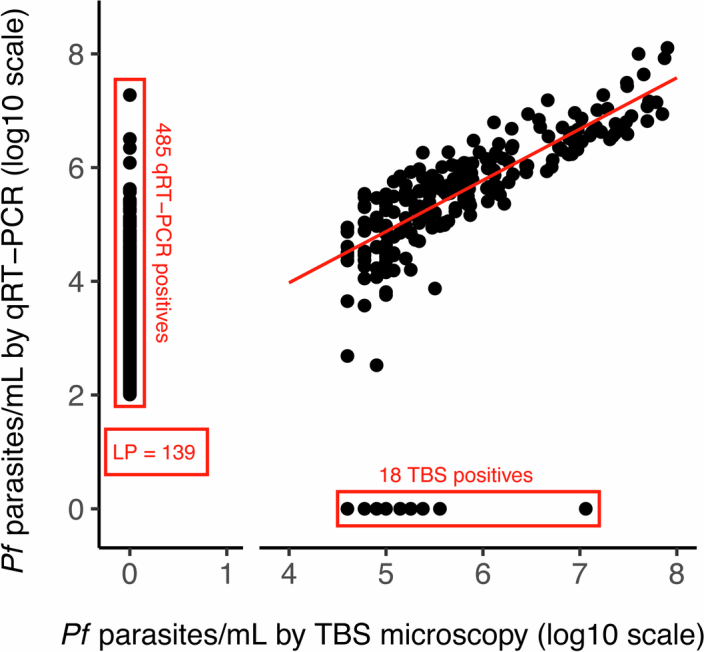
Comparison of *Plasmodium* 18S qRT-PCR and TBS results, including discrepant results. A total of 5015 paired DBS and TBS collected from 330 participants over the 6-month study period were analyzed by 18S qRT-PCR and microscopy, respectively. *P. falciparum* was detected quantitatively by both tests in 214 (4.3%) paired samples, and not detected by either test in 4159 (82.9%) paired samples. Among the remaining 642 discrepant samples, 624 (97.2%) were TBS-negative and either qualitatively low positive (LP; n = 139) or quantitatively positive by 18S qRT-PCR (n = 485). The remaining 18 discrepant samples were 18 s qRT-PCR-negative and TBS-positive (2.8% of discrepant samples and 0.4% of samples overall). Discrepant samples are highlighted by red boxes and the number of such samples is shown.

A total of 5,015 DBS were analyzed by 18S qRT–PCR, all with a paired TBS, allowing for a direct comparison of the two endpoint methods. The LoD for the *Plasmodium* 18S qRT–PCR assay is 20 parasites ml^−1^ (1.3 log_10_ parasites ml^−1^) and the limit of quantification (LoQ) is 100 parasites ml^−1^ (2.0 log_10_ parasites ml^−1^) (Fig. 2a). For this study, the stated LoD and LoQ of TBS is 40,000 parasites ml^−1^ (4.6 log_10_ parasites ml^−1^) (Fig. 2b). Qualitative comparisons of 18S qRT–PCR and TBS were made using samples at or above the LoD, and quantitative comparisons were made using samples at or above the LoQ. Of the 5,015 paired DBS and TBS samples, 838 (16.7%) were qualitatively positive by 18S qRT–PCR (Fig. 2a) and 232 (4.6%) were positive by TBS (Fig. 2b). Among the DBS, 139 (2.8%) were deemed low positive by the 18S qRT–PCR, which corresponds to a density of ~20–100 parasites ml^−1^ (Fig. 2a). Among all samples, 214 (4.3%) were quantitively positive by both tests and 4,159 (82.9%) were negative by both tests. Of the 214 samples quantitatively positive by both assays, parasite densities were highly correlated (*r* = 0.86, *P* < 0.0001; Fig. 2c). There was minimal bias (0.21 log_10_ parasites ml^−1^) between 18S qRT–PCR- and TBS-based parasite densities across >four logs (Extended Data Fig. 1). There were 642 discrepant samples, 624 of which (97.2%) were TBS-negative and qualitatively positive by 18S qRT–PCR. The remaining 18 discrepant samples were 18S qRT–PCR-negative and TBS-positive (2.8% of discrepant samples and 0.4% of samples overall), and most of these (13 of 18) were at parasite densities ≤100,000 ml^−1^ (Extended Data Fig. 2). Among the TBS-negative/qRT–PCR-positive samples, 430 (88.5%) had parasite densities <40,000 ml^−1^ (TBS LoD) (Fig. 2d and Extended Data Fig. 2), demonstrating the superior analytical sensitivity of qRT–PCR for identifying low-density infections.

### Baseline prevalence of infection and clearance after AL

**Fig. 3:**
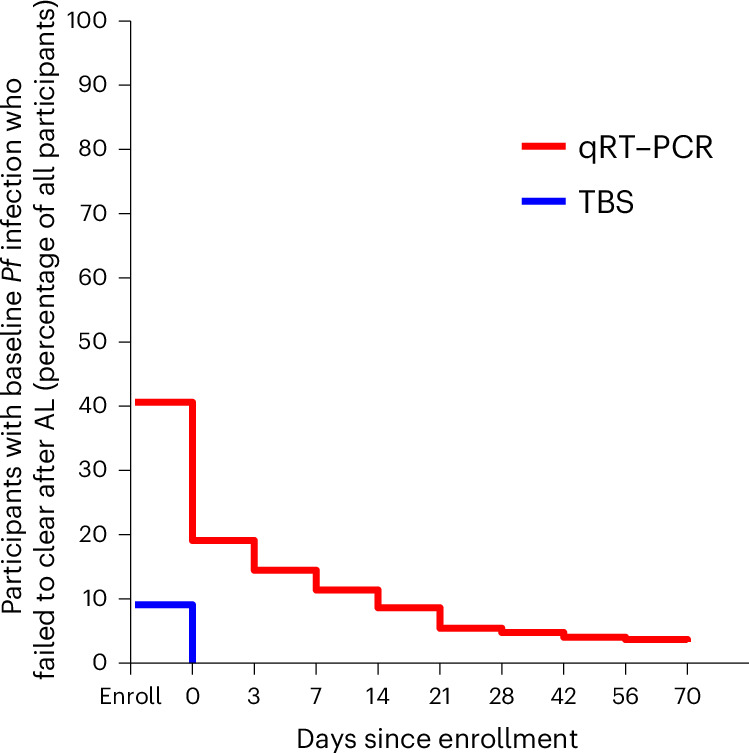
Time to clearance of baseline *P.* *falciparum* infection after AL treatment. Among all study participants (*n* = 330), the percentage who had baseline *P.* *falciparum* at enrollment before AL administration (study day −14 ± 7) is shown, as detected by 18S qRT–PCR (red line) or TBS microscopy (blue line), followed by the percentage of participants who failed to clear their baseline infections by 18S qRT–PCR or TBS by the day of CIS43LS/placebo administration (day 0) or by scheduled visits thereafter through day 70. For each participant who had infection detected by 18S qRT–PCR at enrollment, subsequent clearance of infection was defined as two consecutive negative 18S qRT–PCR assay results. For seven participants (two in the placebo arm, three in the 10 mg kg^−1^ arm and two in the 40 mg kg^−1^ arm), DBS at enrollment were not available for 18S qRT–PCR analysis.

**Extended Data Fig. 3:**
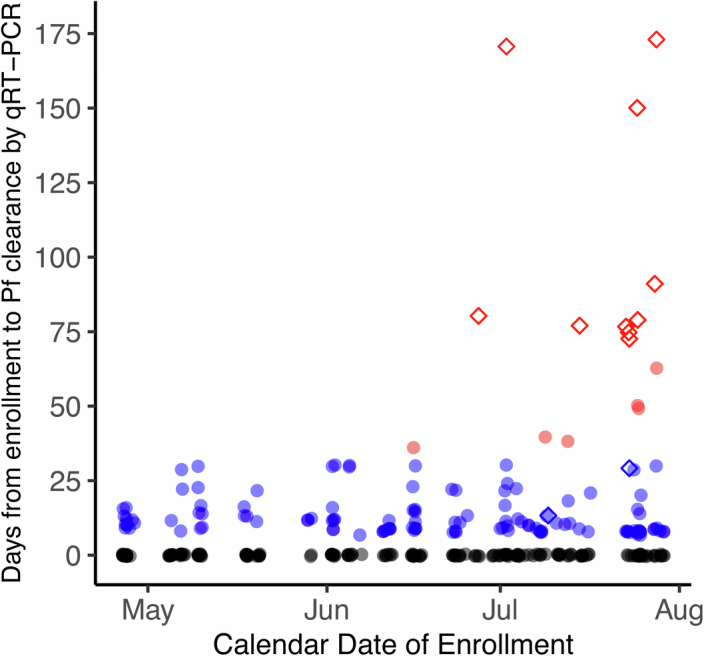
Relationship between enrollment date and time to clearance of baseline *P. falciparum* infection. The y axis represents the number of days from enrollment (when the antimalarial drug AL was given) to the first qualitatively negative *P. falciparum* 18S qRT-PCR, shown by calendar date of enrollment (x axis). Among those who were infected at enrollment, clearance was defined as two consecutive negative 18S qRT-PCR results after enrollment. Each dot represents one participant. The 16 participants who remained 18S qRT-PCR positive through study day 21 (28 – 36 days after enrollment) are shown in shades of red. Participants who were uninfected at enrollment are shown in shades of gray/black (n = 196), and those who were infected at enrollment and cleared infections by study day 21 are shown in shades of blue (n = 118). Diamonds (n = 12) represent participants who were censored (that is participants who were still infected with *P. falciparum* when the last recorded study visit occurred).

**Extended Data Fig. 4:**
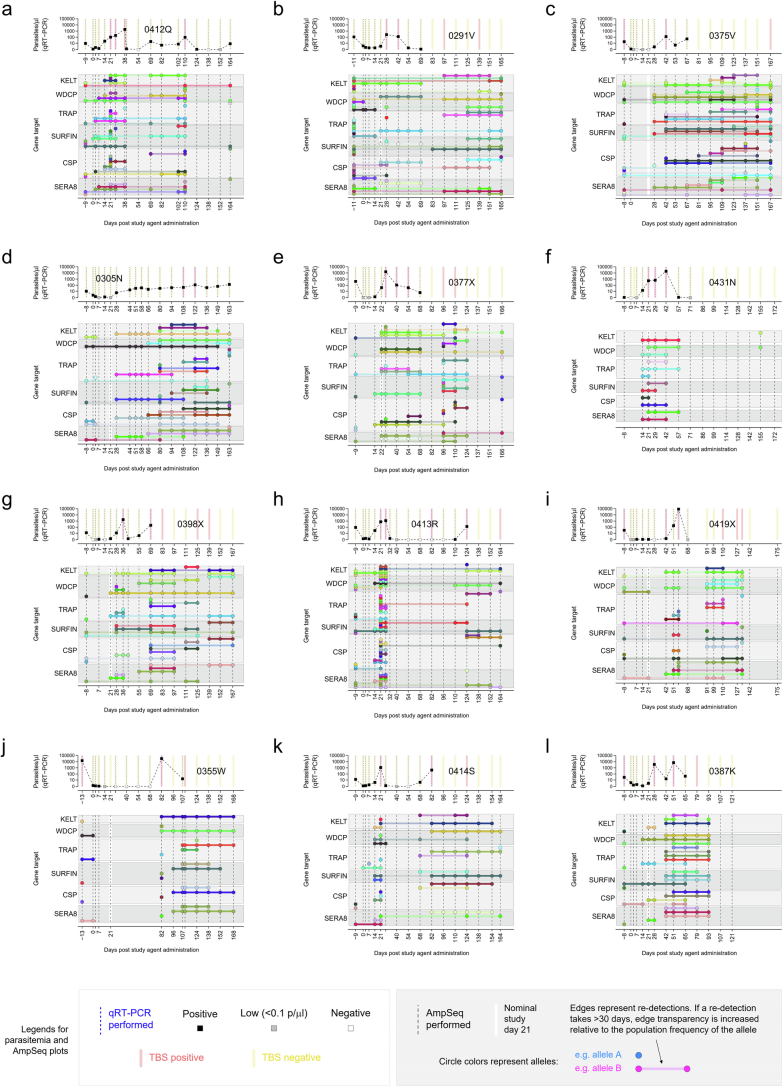
Longitudinal *P. falciparum* genotype analysis of participants who were infected at baseline and remained 18S qRT-PCR positive by study day 21. Panels **a-l** show allelic diversity for 6 *P. falciparum* gene fragments determined via amplicon sequencing (AmpSeq) of DBS from 12 participants who had *P. falciparum* infection detected by 18S qRT-PCR at enrollment (when AL was administered) and who remained infected through at least study day 21 after CIS43LS/placebo administration (study day 0). Clearance of infection was defined as two consecutive negative 18S qRT-PCR results after enrollment. Associated 18S qRT-PCR and thick blood smear (TBS) records are presented at the top of each participant plot. Timepoints ascend left to right from enrollment (8–13 days before study day 0) through 24 weeks for most participants. Circles represent alleles detected by AmpSeq and are grouped according to the analyzed locus (boxes). Circles are ordered vertically within boxes according to timepoint of first detection (earliest at bottom). Matching circle colors represent repeated observations of the same allele, either longitudinally (that is, left to right, see also colored edges) or between participants. The simultaneous detection of multiple alleles within a single locus indicates a polyclonal infection state. A total of 147 unique alleles were observed across all loci for the 12-participant dataset.

Before AL administration at enrollment, *P.* *falciparum* was detected by TBS in 30 of 330 (9.1%) participants (Fig. 3): 8 (7.3%) in the placebo arm, 14 (12.7%) in the CIS43LS 10 mg kg^−1^ arm and 8 (7.3%) in the CIS43LS 40 mg kg^−1^ arm (Table 1). By study day 0, when CIS43LS or placebo was administered (7–15 days after AL administration), infection was undetectable by TBS in all participants (Fig. 3). In marked contrast, before AL administration at enrollment, *P.* *falciparum* was detected by 18S qRT–PCR in 134 of 330 (40.6%) participants (Fig. 3): 47 (42.7%) in the placebo arm, 42 (38.2%) in the CIS43LS 10 mg kg^−1^ arm and 45 (40.9%) in the CIS43LS 40 mg kg^−1^ arm. As it is known that clearance times are longer for 18S qRT–PCR-detected versus TBS-detected infections after drug treatment^24^, and because CIS43LS cannot prevent pre-existing infections, we determined the timepoint at which most participants who were infected at enrollment had cleared their 18S qRT–PCR-detected infections, so that the efficacy of CIS43LS against new infections could be more clearly assessed thereafter. For each participant, we defined clearance as two consecutive negative 18S qRT–PCR results after enrollment. By study day 21, 118 of the 134 participants (88.1%) who were infected at baseline, as detected by 18S qRT–PCR, had cleared their infections (Fig. 3). The 16 participants (4.8% of 330 total participants) who did not clear by study day 21 tended to be enrolled later in the trial (Extended Data Fig. 3), when malaria transmission had probably started, and new infections were possible. Indeed, among the 12 of these 16 participants whose infections we were able to genotype over the study period, nine had evidence of new infections by study day 21 (Extended Data Fig. 4).

### 18S qRT–PCR-defined CIS43LS efficacy against infection

**Fig. 4:**
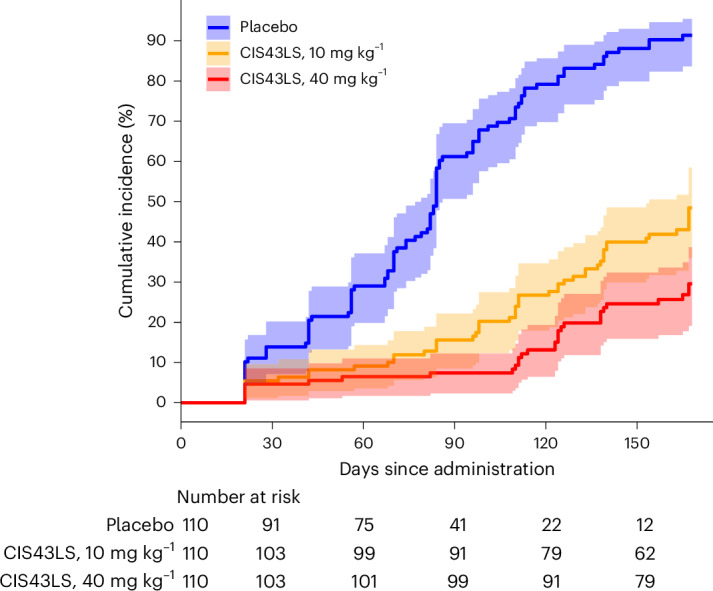
CIS43LS efficacy against *P.* *falciparum* infection as determined by *Plasmodium* 18S qRT–PCR. Cumulative incidence of *P.* *falciparum* blood-stage infection during a 6-month malaria season (irrespective of symptoms being present) after a single IV infusion of 10 mg kg^−1^ of CIS43LS (*n* = 110), 40 mg kg^−1^ of CIS43LS (*n* = 110) or placebo (*n* = 110). *P.* *falciparum* infections were detected by 18S qRT–PCR analysis of DBS collected during scheduled trial visits and unscheduled illness visits when malaria was suspected. DBS were collected before the administration of CIS43LS or placebo on day 0 and then on days 1, 3, 7, 14, 21 and 28 and every 2 weeks thereafter for a total of 24 weeks (day 168). Only DBS collected between days 21 and 168 were included. Estimates of the cumulative risk of infection are plotted along with the 95% CIs (shaded areas).

We conducted a prespecified efficacy analysis among the 330 participants who were included in the modified intention-to-treat population (who were the same participants as the intention-to-treat population) and was based on the time to the first qualitatively positive *P.* *falciparum* infection as detected by 18S qRT–PCR. Infections detected by 18S qRT–PCR with an onset between days 21 and 168 after administration of CIS43LS or placebo occurred in 48 participants (43.6%) who received 10 mg kg^−1^ of CIS43LS, 29 (26.4%) who received 40 mg kg^−1^ of CIS43LS and 96 (87.3%) who received placebo. For comparison, TBS-detected infections with an onset between days 21 and 168 occurred in 38 participants (34.5%) who received 10 mg kg^−1^ of CIS43LS, 19 (17.3%) who received 40 mg kg^−1^ and 85 (77.3%) who received placebo. At 6 months, the 18S qRT–PCR-defined efficacy of 10 mg kg^−1^ of CIS43LS compared with placebo was 77.0% (adjusted 95% CI, 65.0–84.0%; *P* < 0.001) and the efficacy of 40 mg kg^−1^ of CIS43LS compared with placebo was 87.4% (adjusted 95% CI, 79.5–92.3%; *P* < 0.001) (Fig. 4). Schoenfeld residuals indicated no violation of the proportional hazards assumption. A post hoc, sex-stratified analysis of 18S qRT–PCR-defined efficacy suggested that 10 mg kg^−1^ of CIS43LS had higher efficacy in male compared with female participants (81% and 71%, respectively; *P* = 0.01), whereas the efficacy of 40 mg kg^−1^ of CIS43LS was not statistically significantly different by sex.

In a post hoc analysis that excluded participants who had 18S qRT–PCR-detected infections at study day 0 (*n* = 25 from placebo arm, *n* = 15 from 10 mg kg^−1^ arm, *n* = 19 from 40 mg kg^−1^ arm), 18S qRT–PCR-defined efficacy was 83.0% (adjusted 95% CI, 69.0–90.0%; *P* < 0.001) for 10 mg kg^−1^ of CIS43LS and 91.3% (adjusted 95% CI, 82.3–95.7%; *P* < 0.001) for 40 mg kg^−1^ of CIS43LS, compared with placebo.

We conducted an additional prespecified efficacy analysis based on the Kaplan–Meier estimate of the proportion of participants infected with *P.* *falciparum* based on 18S qRT–PCR. Between days 21 and 168, the proportional efficacy of 10 mg kg^−1^ of CIS43LS compared with placebo was 47.0% (adjusted 95% CI, 27.6–60.3%; *P* < 0.001) and the proportional efficacy of 40 mg kg^−1^ of CIS43LS compared with placebo was 67.6% (adjusted 95% CI, 45.6–79.6%; *P* < 0.001).

### Gametocytemia risk after administration of AL and CIS43LS

The 18S qRT–PCR assay detects both asexual and sexual (gametocytes) blood-stage parasites but does not distinguish between these forms. Therefore, in a post hoc analysis we used a *P.* *falciparum* gametocyte mRNA-specific qRT–PCR assay with a LoD of ~300 gametocytes per milliliter of blood^22^ to determine the baseline prevalence of *P.* *falciparum* gametocytemia, the rate of gametocyte clearance after AL and the subsequent risk of new gametocytemia following administration of CIS43LS or placebo. With this assay, we analyzed all DBS that were positive by the more sensitive *Plasmodium* 18S qRT–PCR (*n* = 838) and, if not already tested, all DBS after the first positive 18S qRT–PCR through the last study visit on day 168 (*n* = 204 additional DBS).

**Fig. 5:**
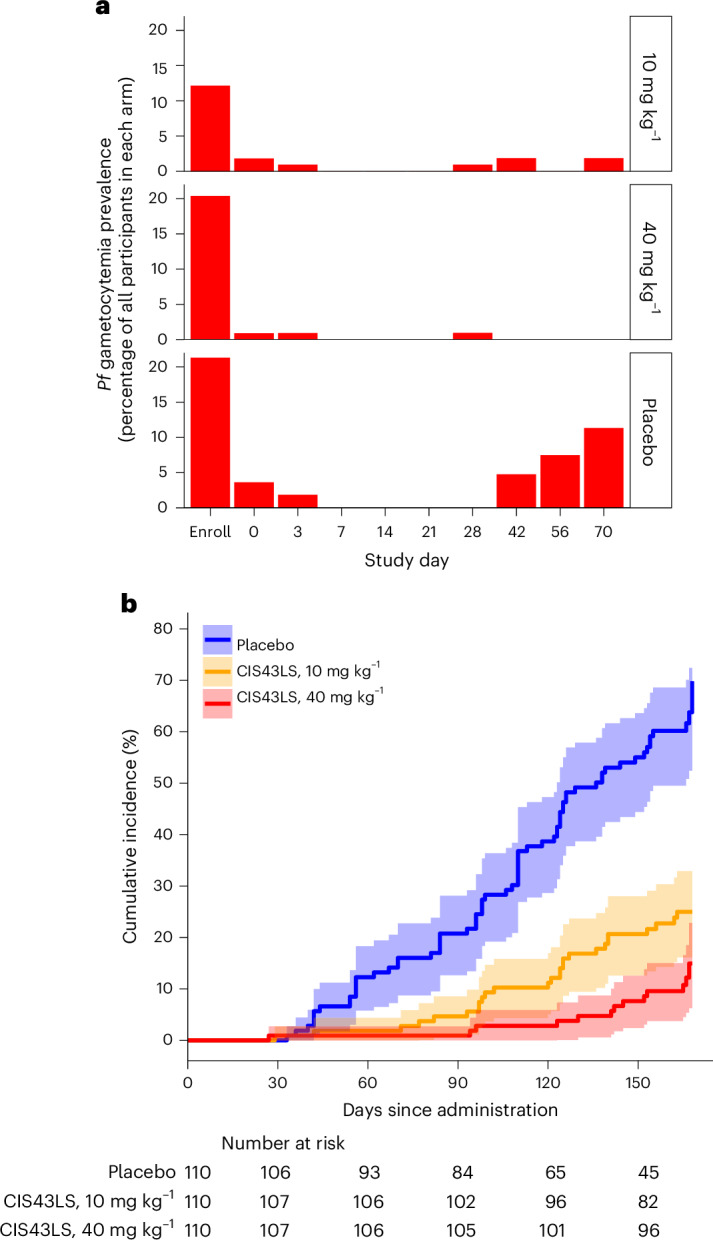
Time to clearance of baseline *P.* *falciparum* gametocytemia after AL administration and subsequent risk of gametocytemia after CIS43LS or placebo administration. **a**, The percentage of participants infected with *P.* *falciparum* gametocytes as detected by a gametocyte mRNA-specific qRT–PCR assay at enrollment before AL administration (study day −14 ± 7), on day 0 when CIS43LS or placebo was administered and at scheduled visits thereafter through day 70, is shown for each trial arm (*n* = 110 for each arm). **b**, Cumulative incidence of *P.* *falciparum* gametocytemia during a 6-month malaria season after a single IV infusion of 10 mg kg^−1^ of CIS43LS (*n* = 110), 40 mg kg^−1^ of CIS43LS (*n* = 110) or placebo (*n* = 110) during scheduled and unscheduled illness visits when malaria was suspected. Only DBS collected between days 7 and 168 were included in this analysis. Estimates of the cumulative risk of gametocytemia are plotted along with the 95% CIs (shaded areas).

Before AL administration at enrollment, *P.* *falciparum* gametocytemia was detected by qRT–PCR in 58 of 330 (17.6%) participants: 13 (11.8%) in the CIS43LS 10 mg kg^−1^ arm, 22 (20.0%) in the CIS43LS 40 mg kg^−1^ arm and 23 (20.9%) in the placebo arm (Fig. 5a). By study day 7 (14–22 days after AL treatment), all participants were free of gametocytemia as detected by qRT–PCR (Fig. 5a).

Among the 330 participants, *P. falciparum* gametocytemia detected by qRT–PCR with an onset between study days 7 and 168 after administration of CIS43LS or placebo occurred in 26 participants (23.6%) who received 10 mg kg^−1^ of CIS43LS, 13 (11.8%) who received 40 mg kg^−1^ of CIS43LS, and 67 (60.9%) who received placebo. The efficacy against gametocytemia of 10 mg kg^−1^ of CIS43LS compared with placebo was 73.0% (adjusted 95% CI, 54.0–84.0%; *P* < 0.001) and the efficacy of 40 mg kg^−1^ of CIS43LS compared with placebo was 87.7% (adjusted 95% CI, 75.6–93.8%; *P* < 0.001) (Fig. 5b).

## Discussion

New antimalarial interventions that are highly efficacious in blocking sporozoite infection, the first stage of the malaria life cycle in humans, would provide an efficient means of preventing disease in susceptible populations while also preventing onward transmission, potentially complementing current malaria countermeasures such as chemoprevention, vaccines and insecticide-treated mosquito nets. Here, using a *Plasmodium* 18S qRT–PCR assay that is ~2,000 times more sensitive than TBS microscopy, we found that the efficacy of one dose of CIS43LS at 40 mg kg^−1^ or 10 mg kg^−1^ against *P. falciparum* infection was 87.4% and 77.0%, respectively, in adults over a 6-month malaria season, during which 87.3% of participants in the placebo group were infected. This is similar to the TBS-defined efficacy that was previously reported for this trial^16^. Moreover, the efficacy of 40 mg kg^−1^ and 10 mg kg^−1^ of CIS43LS in preventing subsequent qRT–PCR-detected gametocytemia was 87.7% and 73.0% over 6 months, respectively. These results provide proof of concept that a single dose of a long-acting anti-sporozoite mAb can confer sterile protection against *P.* *falciparum* infection for up to 6 months, thus preventing the subsequent blood stages that cause disease and onward transmission.

This study provides evidence to support the development of anti-sporozoite mAbs as a strategy to reduce the burden of malaria disease while simultaneously decreasing transmission to accelerate malaria elimination. For example, in annual or semi-annual mass administration campaigns (depending on local malaria transmission patterns), an antimalarial drug with gametocytocidal activity could be given to clear pre-existing infection along with an anti-sporozoite mAb to prevent new infections, thus offering extended protection against disease for susceptible people (for example, infants, children and pregnant women) while reducing malaria transmission at the population level. This approach could be particularly impactful if targeted at populations that disproportionately contribute to transmission, such as school-age children^25^.

In this trial, all participants received a treatment course of the commonly used antimalarial drug AL 7–15 days before the administration of CIS43LS or placebo to clear any pre-existing infections so that the primary efficacy endpoint could be assessed. A post hoc analysis assessed the impact of AL followed by CIS43LS on gametocyte infection risk over the study period using a gametocyte mRNA-specific qRT–PCR with a LoD of ~300 gametocytes per milliliter of blood^22^, a level below which successful mosquito infection is unlikely^26^. The baseline prevalence of qRT–PCR-detected gametocytemia was 17.6%, which dropped to 0% within 14–22 days of AL treatment (study day 7), consistent with previous studies that demonstrated the rapid and potent gametocytocidal and transmission-blocking activity of AL^27–29^. As noted above, CIS43LS was highly efficacious in preventing subsequent qRT–PCR-detected gametocytemia, providing evidence that co-administering an anti-sporozoite mAb with an antimalarial drug has the potential to reduce onward transmission.

Several features of long-acting anti-sporozoite mAbs make their use in mass administration campaigns a potentially feasible and advantageous strategy for malaria elimination. First, unlike vaccines, a single mAb administration provides immediate protection against new infections for a predictable time period. Second, in contrast to small molecule drugs that can select for resistant blood-stage parasites as they rapidly replicate^30,31^, CIS43LS targets a conserved PfCSP epitope on sporozoites, which are nonreplicating^2^, and thus CIS43LS-resistant ‘escape’ variants may be much less likely to arise in vivo under immune pressure of CIS43LS. In the absence of direct selection by CIS43LS on PfCSP variants in the blood stage, the likelihood that a de novo escape variant would rise to a sufficient frequency within a host to make onward transmission likely is reduced. Moreover, the small number of sporozoites injected by mosquitoes reduces the efficiency of selection of any new escape variants that may arise in the oocyst stage in mosquitoes. Third, as a drug class, mAbs are generally safe across all ages and in pregnancy, particularly human-derived mAbs that target pathogens instead of human cells or molecules^32–35^. Like many mAbs in clinical use, CIS43LS is an IgG1 antibody that is not transported across the placenta until the second trimester of pregnancy when the neonatal Fc receptor is expressed^36^. This contrasts with small molecule antimalarial drugs for which safety concerns have limited their use for malaria prevention during the first trimester of pregnancy, and in women of childbearing potential more generally^37^. If future trials confirm the safety and efficacy of anti-sporozoite mAbs in pregnancy, mass administration campaigns could include pregnant women and women of childbearing age, greatly expanding the coverage of malaria elimination efforts.

Although there are promising data for transmission-blocking mAbs^38^ and vaccines^39^ in development that target the nonpathogenic gametocytes, and they may prove to be a powerful complementary strategy for malaria elimination, they do not directly benefit individual recipients, which may complicate their clinical development. The small number of trials in endemic areas that have assessed the ability of the RTS,S/AS01 vaccine to protect against a *P.* *falciparum* infection endpoint have shown low efficacy in children and adults^10–12,40^, thereby limiting its potential as a tool to reduce transmission. The efficacy of the R21/Matrix-M vaccine in preventing infection (and thus transmission) in endemic areas is uncertain; however, an ongoing trial is evaluating the impact of seasonal mass administration of three doses of R21/Matrix-M on the prevalence of malaria at peak transmission (ClinicalTrials.gov identifier: NCT06578572). Additionally, a three-dose IV regimen of the radiation-attenuated *P.* *falciparum* sporozoite vaccine was 51% efficacious against TBS-detected infection at 6 months in Malian adults^41^ but showed no statistically significant efficacy at 6 months in Kenyan infants^42^. Collectively, these data highlight the potential advantage of a single-dose intervention that is highly efficacious in reducing disease and transmission across all age groups.

Given its improved analytical sensitivity, the *Plasmodium* 18S rRNA qRT–PCR assay performed as expected in that it identified more infected samples than the less sensitive TBS method, and quantitatively positive qRT–PCR results correlated with TBS-defined parasite densities. Although TBS is useful for day-to-day safety monitoring in trials, the 18S rRNA biomarker serves as a truer infection detection endpoint that improves the assessment of baseline infections, clearance and subsequent infection risk throughout the course of a trial. This is possible because *Plasmodium* 18S rRNA is highly expressed (5,000–10,000 copies per parasite)^19,21,43^ and stable on DBS^44,45^. Therefore, 18S qRT–PCR can detect a single infected erythrocyte on a 50 µl DBS (20 parasites ml^−1^)—a level of sensitivity that would be difficult to achieve with DNA-based methods that target only two to five copies of the coding genes.

This study has several limitations. First, it was not designed to directly assess the efficacy of combining anti-sporozoite mAbs with antimalarial drugs to reduce transmission, and additional trials are needed to evaluate this strategy. Second, assessing 18S qRT–PCR-defined efficacy from study day 21 onward was a post hoc decision based on the observation that most participants who were infected at enrollment had cleared their infections by study day 21 after AL treatment (Fig. 3), thus allowing the efficacy of CIS43LS against new infections to be more clearly assessed thereafter. An alternative strategy would have been to exclude people who were infected at baseline; however, this approach could have biased the study population toward lower infection risk and overestimated CIS43LS efficacy. Indeed, a post hoc sensitivity analysis that excluded participants with 18S qRT–PCR-detected infections at study day 0 yielded slightly higher estimates of CIS43LS efficacy. Third, CIS43LS was given intravenously, since this was the first trial in Africa in adults and it was important to establish safety and efficacy in a high dose group. This route is less practical in endemic areas. The development of more potent mAbs would enable subcutaneous and intramuscular administration at lower doses across all ages. To that end, L9LS is a mAb that also targets a conserved PfCSP epitope and was more potent than CIS43LS in preclinical studies^46^, and protected adults against CHMI after a single subcutaneous administration^47^. Subsequently, a phase 2 trial involving school-age children in Mali demonstrated that a single subcutaneous dose of L9LS was protective against *P.* *falciparum* infection and clinical malaria over a 6-month malaria season^23^. A phase 2 trial in Kenya is assessing the efficacy of subcutaneous administration of L9LS against intense perennial transmission in children 5 months to 5 years of age (ClinicalTrials.gov identifier: NCT05400655) and a phase 1b trial in Mali is evaluating the safety and tolerability of intramuscular administration of L9LS in infants 1–12 months of age (ClinicalTrials.gov identifier: NCT06461026), the populations at highest risk of severe and fatal malaria, and the highest priority clinical use case for anti-sporozoite mAbs^15^. Since mAb dosing is weight-based, L9LS may prove to be cost effective for disease prevention in infants and children, whereas mass administration across all ages for elimination may currently be cost prohibitive. As more potent anti-sporozoite mAbs continue to be discovered and developed, mass administration for malaria elimination may prove feasible and cost effective.

This study provides evidence to support the continued development of long-acting anti-sporozoite mAbs as a promising tool to simultaneously reduce the burden of malaria disease and decrease transmission to accelerate malaria elimination.

## Methods

### Clinical trial design and oversight

This two-part phase 2 randomized, placebo-controlled trial (NCT04329104) evaluated the safety and tolerability of a single IV administration of CIS43LS, as well as its protective efficacy against naturally occurring *P.* *falciparum* infection over a 6-month malaria season in healthy men and nonpregnant women 18–55 years of age. All recruitment as well as clinical data and biospecimen collection occurred at the Kalifabougou, Mali study site from 15 February 2021 to 24 January 2022. Participant sex was determined based on self-reporting. Additional inclusion and exclusion criteria are detailed in the protocol provided in Supplementary Information. At the study site in Mali, *P.* *falciparum* transmission typically occurs from July through December each year^48^. Regarding disease burden at the study site, the mean number of clinical malaria cases per person-year among 6- to 10-year-old children (who are not eligible for seasonal malaria chemoprevention in Mali) was 3.4 in 2022, where clinical malaria was defined as an illness accompanied by any level of *P.* *falciparum* asexual parasitemia that resulted in the receipt of antimalarial treatment^23^. The first part of the study was an open-label dose-escalation study for safety and tolerability, the results of which have been published^16^. The second part of the study was a randomized, double-blind, placebo-controlled trial (*N* = 330 total, *n* = 110 for each of three treatment arms) to assess the safety and protective efficacy of CIS43LS at a dose of 10 mg kg^−1^ or 40 mg kg^−1^ compared to placebo. The primary efficacy endpoint in the second part of the trial was *P. falciparum* blood-stage infection as detected by microscopic examination of TBS for 24 weeks after administration of CIS43LS or placebo, the results of which have been reported^16^. The portion of the study reported here focused on the prespecified secondary efficacy endpoint in the second part of the trial, namely, *P.* *falciparum* blood-stage infection as detected by 18S qRT–PCR for 24 weeks after administration of CIS43LS or placebo. Block randomization was used with block size varying from six to nine. The random allocation sequence was generated by the protocol statistician who was not involved in the enrollment and study product assignment procedures and remained unaware of the trial-group assignments. Trial participants and trial team members were also unaware of the trial-group assignments. All participants were given a standard treatment course of AL at enrollment 7–15 days before study agent administration to clear any pre-existing *P.* *falciparum* blood-stage infection so that efficacy end points could be assessed. The administration of all doses of AL was observed directly by study staff. For the remainder of the trial, asymptomatic *P.* *falciparum* infections were not treated, in accordance with national guidelines in Mali. All participants in whom symptomatic malaria developed during the trial were provided standard treatment. DBS for 18S qRT–PCR analysis were collected during unscheduled illness visits and scheduled trial visits at enrollment before AL administration, before the administration of CIS43LS or placebo on day 0, and then on days 1, 3, 7, 14, 21 and 28, and every 2 weeks thereafter for a total of 24 weeks (same collection schedule as TBS, although DBS from study day 1 were not analyzed by 18S qRT–PCR).

The trial was conducted in accordance with International Council for Harmonisation Good Clinical Practice guidelines and applicable regulations in Mali. The United States Food and Drug Administration (FDA) reviewed the trial protocol in the investigational new drug application (IND 147485), sponsored by the National Institute of Allergy and Infectious Diseases. The protocol and informed consent forms were approved by the ethics committee at Faculté de Médecine et d’Odonto-Stomatologie and Faculté de Pharmacie at the University of Sciences, Techniques and Technologies of Bamako and by the national regulatory authorities of Mali. Community permission was obtained from participating sites^49^, and all participants provided written informed consent.

### Trial product

CIS43LS is a human IgG1 mAb derived from a Chinese hamster ovary DG44 stably transfected clonal cell line^14^. It was manufactured according to Current Good Manufacturing Practice requirements by the Vaccine Clinical Materials Program (operated under contract with Leidos Biomedical Research) and vialed in a buffered formulation at a concentration of 100 mg ml^−1^.

### Collection of DBS and blood smears

At each scheduled trial visit and at all unscheduled illness visits at which malaria was suspected by the trial clinicians, a finger prick blood (or venipuncture) sample was obtained from which a Giemsa-stained TBS and thin blood smear was obtained, as well as DBS onto Protein Saver 903 DBS cards (Global Life Sciences Solutions). Blood smears were stored at room temperature and were read at the study laboratory in Mali. After collection, DBS were dried at the clinic laboratory for approximately 4 h and then stored in gas impermeable plastic bags with a desiccant packet at −80 °C. DBS were stored at −80 °C (5–6 months) and then shipped at ambient temperature to the University of Washington until processed.

### Blood smear microscopy

Blood smears were analyzed by two independent readers who were unaware of the trial-group assignments. A third reader examined blood smears when discrepancies occurred. A positive blood smear was defined as two independent readers both reporting the presence of any *P.* *falciparum* asexual parasites after counting 2,500 leukocytes or examining 200 high-power fields. The competency of blood-smear readers is assessed regularly at the Mali Research and Training Center laboratory, which is certified by the College of American Pathologists.

### *P.falciparum* 18S rRNA qRT–PCR

The laboratory that performed the Gen3.5DBS *Plasmodium* 18S qRT–PCR was blinded to the trial-group assignments. DBS were laser cut using contact-free methods^50^, deposited into 2 ml NucliSENS lysis buffer (bioMérieux) and incubated at 55 °C for 30 min as described^20^. RNA was extracted from 1 ml of the lysate and eluted into 88 μl of elution buffer by the Abbott m2000*sp* system using the mSample RNA preparation kit (Abbott Molecular). A total of 15 µl of the extracted RNA was combined with 35 µl of SensiFAST Probe Lo-ROX One-Step Kit mastermix (Meridian Bioscience) and subjected to qRT–PCR on the Abbott m2000*rt*. The qRT–PCR primers and probes targeted the *P.* *falciparum* 18S rRNA (forward, PfDDT1451F21: 5′-GCGAGTACACTATATTCTTAT-3′; reverse, PfDDT1562R21: 5′-ATTATTAGTAGAACAGGGAAA-3′; probe, 5′-[6-FAM]-ATTTATTCAGTAATCAAATTAGGAT-3′ [Black Hole Quencher 1 PLUS]; LGC BioSearch Technologies), pan-*Plasmodium* 18S rRNA (forward, PanDDT1043F19: 5′-AAAGTTA[+A]GG GA[+G][+T]GAAGA-3′; reverse, PanDDT1197R22: 5′-AA[+G]ACTTTGA TTTCTC[+A]TAAGG-3′; Qiagen; Probe: 5′-[CAL Fluor Orange 560]-ACCGTCG TAATCTTAACCATAAACTA[T(Black Hole Quencher 1)] GCCGACTAG-3′[Spacer C3]; LGC BioSearch Technologies) and the human TATA binding protein mRNA control (forward, 5′-GATAAGAGAGCCACGAACCAC-3′; reverse, 5′-CAAGAACTTAGCTGGAAAACCC-3′; probe, 5′-[Quasar 670]-CACAGGAGCCAAGAGTGAAGAACAGT-3′[Black Hole Quencher-2]; LGC BioSearch Technologies) except with thermocycling conditions of 10 min at 45 °C, 2 min at 95 °C, followed by 40 cycles of 5 s at 95 °C and 35 s at 54 °C. Absolute quantification was determined using an Armored RNA calibrator encoding the full-length *P.* *falciparum* 18S rRNA and estimation of ring-stage parasites per milliliter of blood was made using a conversion factor as described^19^.

### *P.falciparum* gametocyte mRNA-specific qRT–PCR

qRT–PCR specific for Pf3D7_0630000 gametocyte mRNA was performed on RNA extracted from DBS as described^22^ using Pf3D7-0630000-specific reagents (forward, 5′AACGCAGAAATAGAAAATC-3′; reverse, 5′-GTCATAAGCAGATACATTC-3′; probe, 5′-[6-FAM]-TGGTATATTGGGAAGTGTAGAAGAG-[3′), the aforementioned human TATA binding protein mRNA control reagents and a Quantstudio real time PCR instrument (ThermoFisher). The SensiFAST Probe Lo-ROX One-Step Kit mastermix (Meridian Bioscience) was used with cycling conditions of 48 °C for 10 min followed by 95 °C for 2 min and then 40 cycles of 95 °C for 5 s and 50 °C for 60 s.

### *P.falciparum* antigen genotyping via amplicon sequencing

We used Illumina-based amplicon sequencing (AmpSeq) to assess allelic diversity in six *P.* *falciparum* gene fragments amplified from DBS samples of study participants who had *P.* *falciparum* infection detected by 18S qRT–PCR at enrollment (when AL was administered) and who remained infected through at least study day 21 after CIS43LS/placebo administration. The six target fragments represent short sections (188–320 bp, excluding primer binding sites) of the genes encoding CSP (PF37_0304600), TRAP (PF3D7_1335900), SERA8 (PF3D7_0207300), SURFIN (PF3D7_0424400), KELT (PF3D7_1475900) and WD-repeat containing protein (PF3D7_1410300). The fragments were selected due to their high population-level heterozygosity in Pf3k^51^ variant call data (>0.8 haplotype diversity estimated per locus within Mali using the haplotype diversity function of the scikit-allel package, v.1.2.0 (ref. ^52^)) and due to lower primer cross-reactivity compared to that observed during previous 4CAST-genotyping^53^ of a similar clinical sample set^23^. We extracted total bloodspot DNA via King Fisher Flex instrument using King Fisher Ready DNA Ultra 2.0 Prefilled Plates (ThermoFisher Scientific) and performed PCR1 on DNA extracts (no additional pre-amplification) using an initial incubation step at 95 °C (3 min); 29 amplification cycles at 98 °C (20 s), 57 °C (15 s), and 72 °C (30 s) and a final extension step at 72 °C (1 min). PCR1 products underwent Exonuclease I digestion (ThermoFisher Scientific) and subsequent one-third dilution in nuclease-free water. PCR2 used an initial incubation step at 95 °C (3 min); ten amplification cycles at 98 °C (20 s), 65 °C (30 s) and 72 °C (30 s) and a final extension step at 72 °C (1 min). We applied single-sided size selection (AMPure XP) before sequencing pooled libraries via 500-cycle (2 × 251 bp) Illumina MiSeq and resolved microhaplotypes (alleles) from sequence output using DADA2 (v.1.14.0)—an amplicon denoising algorithm that tests whether nucleotide variation around abundant sequence types is statistically consistent with expected error distribution or a consequence of true biological variation in the sample set^54^. We performed DADA2-denoising by running the malaria amplicon processing pipeline available at https://github.com/broadinstitute/malaria-amplicon-pipeline.git (default parameters) and filtered resultant read count matrices to exclude microhaplotypes supported by <200 read-pairs or by <10% total read-pairs within a locus. We further organized counts according to sample, locus and timepoint using the stringr package (v.1.5.1)^55^ and created longitudinal visualizations using base plotting functions (points, segments, abline, rect) in R (v.2024.04.1).

### Statistical analysis

As prespecified in the protocol, the efficacy analysis reported here was based on the time to first *P.* *falciparum* infection, as well as the proportion of infected participants in each arm, over the 6-month study period as detected by 18S qRT–PCR. The analysis used the modified intention-to-treat dataset, which included all randomized participants who received the study agent and were analyzed according to the randomized assignment. Time-to-event efficacy was calculated as Efficacy (%) = (1 − HR) × 100, in which HR is the hazard ratio of infection in each CIS43LS arm versus the placebo arm, estimated from the Cox proportional hazards model that accounts for interval censoring. The proportional hazards assumptions were tested based on the scaled Schoenfeld residuals, and a *P* value of less than 0.05 was considered to indicate statistical significance. *P* values were reported based on two-sided Wald tests. Proportional efficacy was calculated as Efficacy (%) = (1 – RR) × 100, in which RR is the relative risk of infection in each CIS43LS arm versus the placebo arm, with the proportion of infected participants in each arm estimated via the Kaplan–Meier method and compared across arms with a two-sided test. Holm’s method was used to address multiplicity in comparing the two CIS43LS arms against the placebo arm, and the adjusted 95% CIs were reported. The 18S qRT–PCR experiments were randomized and the investigators who generated the 18S qRT–PCR data were blinded to allocation during experiments and outcome assessment. Clinical trial data were collected with DFexplore 2023 (v.5.7.0), which secures data with AES 256 encryption and is fully compliant with HIPAA, GDPR and FDA 21 CFR Part 11 regulations and is ISO 9001:2015 certified. Analyses were performed with JMP v.16.2.0 and RStudio v.2024.04.1, and the R packages ‘icenReg’ (v.2.0.16) and ‘bpcp’ (v.1.4.2) were used for time-to-event and proportional analysis, respectively.

### Reporting summary

Further information on research design is available in the Nature Portfolio Reporting Summary linked to this article.

## Online content

Any methods, additional references, Nature Portfolio reporting summaries, source data, extended data, supplementary information, acknowledgements, peer review information; details of author contributions and competing interests; and statements of data and code availability are available at 10.1038/s41591-025-03739-y.

## Supplementary information

Supplementary Information Clinical trial protocol.

Reporting Summary

## Data Availability

Requests for access to the individual deidentified trial participant dataset (including data dictionaries) can be submitted to corresponding author P.D.C. Upon reasonable request, the corresponding author will respond within 2 weeks. In addition, reported results from this trial are publicly available at https://clinicaltrials.gov/study/NCT04329104?intr=cis43ls&rank=1. Supporting clinical documents (protocol and statistical analysis plan) are provided as Supplementary Information.
