# Supplementary material for: Anti-sporozoite monoclonal antibody for malaria prevention: secondary efficacy outcome of a phase 2 randomized trial
Source: Nat Med. 2025 Jun 3;31(8):2682–90. doi: 10.1038/s41591-025-03739-y (PMC12353790; doi:10.1038/s41591-025-03739-y)
Supplement: Supplementary file 1 — Clinical trial protocol. [file 41591_2025_3739_MOESM1_ESM.pdf]

nature medicine

Supplementary information https://doi.org/10.1038/s41591-025-03739-y

Anti-sporozoite monoclonal antibody for malaria prevention: secondary efficacy outcome of a phase 2 randomized trial

In the format provided by the authors and unedited

Mali Malaria mAb Trial Protocol Supplement

This supplement contains the following items:

1. Original protocol/SAP (SAP located in section 9 of original protocol, version date February 21, 2020)
2. Final protocol/SAP (SAP located in section 9 of final protocol, version date October 13, 2022)
3. Summary of protocol amendments
4. Summary of SAP amendments

Abbreviated Title: Anti-malaria MAb in Mali

FMPOS Protocol #: [TBD]

Version Date: February 21, 2020

Title: Safety and Efficacy of VRC-MALMAB0100-00-AB (CIS43LS), a Human Monoclonal Antibody against *Plasmodium falciparum*, in a Dose-Escalation Trial and a Randomized, Double-Blind Trial of Adults in Mali

FMPOS Principal Investigator: Kassoum Kayentao, MD, MPH, PhD
Malaria Research and Training Center (MRTC)
Faculté de Médecine Pharmacie d'Odontostomatologie (FMPOS)
University of Sciences, Techniques, & Technologies of Bamako (USTTB)

NIH Principal Investigator: Peter D. Crompton, MD, MPH
Laboratory of Immunogenetics (LIG)
National Institute of Allergy and Infectious Diseases (NIAID)
National Institutes of Health (NIH)

Investigational Agent:

Drug Name	VRC-MALMAB0100-00-AB (CIS43LS)
Investigational New Drug (IND) Number	TBD
Sponsor	Office of Clinical Research Policy and Regulatory Operations (OCRPRO), Division of Clinical Research, NIAID
Manufacturer	Vaccine Research Center (VRC), NIAID

Data and Safety Monitoring Board (DSMB): NIAID Intramural DSMB

STUDY STAFF ROSTER

FMPOS Investigators:

The research activities of the following FMPOS investigators will be reviewed by:

FMPOS Ethics Committee
BP E302, Point G/FMPOS
Bamako, Mali
FWA #00001769

FMPOS Principal Investigator: Kassoum Kayentao, MD, MPH, PhD
MRTC, FMPOS, USTTB
[redacted]
Phone: [redacted]
Email: [redacted]
Role(s): A, B, C, D, E, F

FMPOS Associate Investigators: Boubacar Traore, PharmD, PhD (Senior Investigator)
MRTC, FMPOS, USTTB
Phone: [redacted]
Email: [redacted]
Role(s): A, B, C, D, E, F

Aissata Ongoiba, MD, MPH
MRTC, FMPOS, USTTB
Phone: [redacted]
Email: [redacted]
Role(s): A, B, C, D, E, F

Safiatou Doumbo, MD, MS
MRTC, FMPOS, USTTB
Phone: [redacted]
Email: [redacted]
Role(s): A, B, C, E, F

Didier Doumtabe, PharmD, MS
MRTC, FMPOS, USTTB
Phone: [redacted]
Email: [redacted]
Role(s): A, B, C, E, F

Abdrahamane Traore, MD
MRTC, FMPOS, USTTB
Phone: [redacted]
Email: [redacted]
Role(s): A, B, C, E, F

NIH Investigators:

NIH investigators are involved in study design, implementation, analysis of coded samples and data, and writing and dissemination of reports of study results. Although they may support Malian investigators in monitoring/oversight capacities, NIH investigators will not be engaged in human subjects research.

NIH Principal Investigator and Referral Contact:

Peter D. Crompton, MD, MPH
LIG, NIAID, NIH
[redacted]
Phone: [redacted]
Email: pcrompton@niaid.nih.gov
Role(s): F

NIH Employee Associate Investigators:

Robert A. Seder, MD (Senior Investigator)
Chief, Cellular Immunology Section
VRC, NIAID, NIH
Phone: [redacted]
Email: rseder@mail.nih.gov
Role(s): F

Martin Gaudinski, MD
Medical Director
VRC, NIAID, NIH
Phone: [redacted]
Email: [redacted]
Role(s): F

Non-NIH Employee Associate Investigators: Sara Healy, MD, MPH (Contractor)
Medical Science & Computing, LLC
In support of LIG, NIAID, NIH
Phone: [redacted]
Email: [redacted]
Role(s): F

Statistician: Zonghui Hu, PhD
Biostatistics Research Branch, NIAID, NIH
Phone: [redacted]
Email: [redacted]
Role(s): F

For each person listed above, identify their roles with the appropriate letter:

A. *Obtain information by intervening or interacting with living individuals for research purposes*
B. *Obtaining identifiable private information about living individuals*
C. *Obtaining the voluntary informed consent of individuals to be subjects*
D. *Makes decisions about subject eligibility*
E. *Studying, interpreting, or analyzing identifiable private information or data/specimens for research purposes*
F. *Studying, interpreting, or analyzing coded (linked) data or specimens for research purposes*
G. *Some/all research activities performed outside NIH*

Study Sites:

Kalifabougou MRTC Clinic
Kalifabougou, Mali
Région de Koulikoro
Préfecture de Kati, Commune de Kalifabougou, Centre de Santé Communautaire de Kalifabougou

Torodo MRTC Clinic
Torodo, Mali
Région de Koulikoro
Préfecture de Kati, Commune de Torodo, Centre de Santé Communautaire de Torodo

Sponsor Medical Monitor (SMM): Shirley Jankelevich, MD (Contractor)
Leidos Biomedical Research, Inc.
Support to: OCRPRO, NIAID, NIH
Phone: [redacted]
Email: [redacted]

Abbreviated Title: Anti-malaria MAb in Mali
Version Date: February 21, 2020

TABLE OF CONTENTS

STATEMENT OF COMPLIANCE ... 10
1 PROTOCOL SUMMARY ... 11
1.1 Synopsis ... 11
1.2 Schema ... 13
1.3 Schedule of Activities (SOA) ... 15
1.3.1 Dose-escalation Study ... 15
1.3.2 Efficacy Study ... 17
2 INTRODUCTION ... 20
2.1 Study Rationale ... 20
2.2 Background ... 20
2.2.1 Overview ... 20
2.2.2 Study Product: CIS43LS ... 21
2.2.3 Laboratory Assessments of CIS43LS ... 24
2.3 Risk/Benefit Assessment ... 25
2.3.1 Known Potential Risks ... 25
2.3.2 Known Potential Benefits ... 27
2.3.3 Assessment of Potential Risks and Benefits ... 27
3 OBJECTIVES AND ENDPOINTS ... 27
4 STUDY DESIGN ... 29
4.1 Overall Design ... 29
4.2 Scientific Rationale for Study Design ... 30
4.3 Justification for Dose ... 30
5 STUDY POPULATION ... 30
5.1 Inclusion Criteria ... 30
5.2 Exclusion Criteria ... 31
5.3 Inclusion or Exclusion of Vulnerable Participants ... 32
5.4 Lifestyle Considerations ... 32
5.5 Screen Failures ... 32
5.6 Strategies for Recruitment and Retention ... 33
5.6.1 Costs ... 33
5.6.2 Compensation ... 33

6 STUDY INTERVENTION 34
6.1 Study Intervention Administration 34
6.1.1 Study Intervention Description 34
6.1.2 Dosing and Administration 34
6.2 Preparation/Handling/Storage/Accountability 35
6.2.1 Acquisition and Accountability 35
6.2.2 Formulation, Appearance, Packaging, and Labeling 36
6.2.3 Product Storage and Stability 36
6.2.4 Preparation 36
6.3 Measures to Minimize Bias: Randomization and Blinding 37
6.3.1 Randomization 37
6.3.2 Blinding 37
6.4 Study Intervention Compliance 38
6.5 Concomitant Therapy 38
7 STUDY INTERVENTION DISCONTINUATION AND PARTICIPANT DISCONTINUATION/WITHDRAWAL 38
7.1 Discontinuation of Study Intervention 38
7.2 Participant Discontinuation/Withdrawal from the Study 38
7.3 Lost to Follow-up 38
8 STUDY ASSESSMENTS AND PROCEDURES 39
8.1 Screening Procedures 39
8.2 Efficacy Assessments 40
8.2.1 Clinical Evaluations 40
8.2.2 Biospecimen Evaluations 41
8.2.3 Correlative Studies for Research/Pharmacokinetic Studies 41
8.2.4 Samples for Genetic/Genomic Analysis 42
8.3 Safety and Other Assessments 42
8.4 Safety Definitions, Management, and Sponsor Reporting 43
8.4.1 Definitions 43
8.4.2 Documenting, Assessing, Recording, and Reporting Events 46
8.4.3 Withdrawal Criteria for an Individual Subject 51
8.4.4 Additional Safety Oversight 52

8.4.5 Pausing Rules 53
8.4.6 Halting Rules for the Protocol 54
8.5 Unanticipated Problems 55
8.5.1 Definition of an Unanticipated Problem 55
8.5.2 Unanticipated Problem Reporting 55
8.6 ADDITIONAL REPORTING REQUIREMENTS 55
8.6.1 Reporting to the FMPOS EC 55
8.6.2 Reporting to the NIAID Clinical Director 56
9 STATISTICAL CONSIDERATIONS 56
9.1 Statistical Hypothesis 56
9.2 Sample Size Determination 56
9.2.1 Sample Size Considerations for the Dose-Escalation Study 56
9.2.2 Sample Size Considerations for the Efficacy Study 57
9.3 Populations for Analyses 59
9.3.1 Evaluable for Toxicity 59
9.3.2 Evaluable for Objective Response 59
9.3.3 Evaluable Non-Target Disease Response 60
9.4 Statistical Analyses 60
9.4.1 General Approach 60
9.4.2 Analysis of the Primary Endpoints 60
9.4.3 Analysis of the Secondary Endpoints 60
9.4.4 Efficacy Analyses 60
9.4.5 Safety Analyses 61
9.4.6 Pharmacokinetics Analysis 61
9.4.7 Baseline Descriptive Statistics 62
9.4.8 Planned Interim Analyses 62
9.4.9 Sub-group Analyses 62
9.4.10 Tabulation of Individual Participant Data 62
9.4.11 Exploratory Analyses 63
10 REGULATORY AND OPERATIONAL CONSIDERATIONS 63
10.1 Informed Consent Process 63
10.1.1 Consent Procedures and Documentation 63

10.1.2 Consent for Minors When They Reach the Age of Majority 64
10.1.3 Telephone Consent 64
10.1.4 Telephone Assent 64
10.1.5 Participation of Subjects who are/become Decisionally Impaired 64
10.2 Study Discontinuation and Closure 64
10.3 Confidentiality and Privacy 64
10.4 Future Use of Stored Specimens and Data 65
10.5 Safety Oversight 66
10.6 Clinical Monitoring 66
10.7 Quality Assurance and Quality Control 66
10.8 Data Handling and Record Keeping 66
10.8.1 Data Collection and Management Responsibilities 66
10.8.2 Study Records Retention 67
10.9 Protocol Deviations 67
10.9.1 NIH Definition of Protocol Deviation 67
10.10 Publication and Data Sharing Policy 67
10.10.1 Human Data Sharing Plan 67
10.10.2 Genomic Data Sharing Plan 68
10.11 Collaborative Agreements 68
10.11.1 Agreement Type 68
10.12 Conflict of Interest Policy 68
11 ABBREVIATIONS 68
12 REFERENCES 70
APPENDIX A: MRTC CLINICAL LABORATORY NORMAL VALUES 73

List of Tables

Table 1. Study agent assignment and dosing by arm. 34
Table 2. Standard event recording, assessment, and reporting timeframes. 46
Table 3. Probability (Pr) of events for different safety scenarios within an arm (n=6 or 18). 56
Table 4. Power for efficacy evaluation under a 2-sided type I error rate of 0.05. 57
Table 5. Minimum detectable difference in adverse event rate in arms receiving monoclonal antibody (MAb) or placebo under a 2-sided type I error rate of 0.05. 59

List of Figures

Figure 1. Dose-escalation study flow diagram. 13
Figure 2. Efficacy study flow diagram. 14

STATEMENT OF COMPLIANCE

The trial will be carried out in accordance with International Council on Harmonisation (ICH) Good Clinical Practice (GCP) and the following:

- United States (US) Code of Federal Regulations (CFR) applicable to clinical studies (45 CFR Part 46, 21 CFR Part 50, 21 CFR Part 56, 21 CFR Part 312, and/or 21 CFR Part 812)

NIH-funded investigators and clinical trial site staff who are responsible for the conduct, management, or oversight of NIH-funded clinical trials have completed Human Subjects Protection and ICH GCP Training.

The protocol and informed consent forms will be submitted to the FMPOS Ethics Committee (EC) for review and approval. Approval of both the protocol and the consent form must be obtained before any subject is enrolled. Any amendment to the protocol will require review and approval by the EC before the changes are implemented to the study. In addition, all changes to the consent form will be EC-approved; a determination will be made regarding whether a new consent needs to be obtained from subjects who provided consent, using a previously approved consent form.

1 PROTOCOL SUMMARY

1.1 SYNOPSIS

Title: Safety and Efficacy of VRC MALMAB0100-00-AB (CIS43LS), a Human Monoclonal Antibody against Plasmodium falciparum, in a Dose-Escalation Trial and a Randomized, Double-Blind Trial of Adults in Mali

Study Description: A two-part phase 2 trial evaluating the safety and tolerability of one-time administration of CIS43LS, as well as its protective efficacy against naturally occurring *Plasmodium falciparum* (Pf) infection over a 6-month malaria season. The primary study hypotheses are that CIS43LS will be safe and will produce protection against malaria infection. Before study agent administration, all subjects will be given artemether-lumefantrine to clear any preexisting Pf blood-stage infection.

Dose-escalation study: The first part of the study is an open-label dose-escalation study for safety and tolerability. Subjects will be assigned to 1 of 3 dose arms (N=18 total, n=6 for each dose level). Dosing will begin in the lowest dose arm. Once all subjects in that arm reach day 7 post-infusion, if no safety concerns have arisen, dosing will begin at the subsequent dose level. This process will be repeated until subjects complete the third dose arm. Subjects will be followed for safety to assess adverse events (AEs) at study visits 1, 3, 7, 14, 21, and 28 days after administration, then monthly through 24 weeks after administration.

After the last subject in the highest dose arm reaches day 7 safety follow-up, an interim safety evaluation will be performed before enrollment begins for the second part of the study.

Efficacy study: The second part of the study is a randomized, double-blind, placebo-controlled trial (N=180 total, n=90 for each of 2 treatment arms) to assess safety and protective efficacy of CIS43LS and placebo.

Subjects in the efficacy study will receive the study agent and be followed at study visits 1, 3, 7, 14, 21, and 28 days later, and once every 2 weeks thereafter through 24 weeks. Primary study assessments include physical examination and blood collection for identification of Pf infection and other research laboratory evaluations.

Objectives: Primary Objectives:

1. Dose escalation: To evaluate the safety and tolerability of CIS43LS administered intravenously (IV) at 5, 20, and 40 mg/kg in healthy Malian adults.
2. Efficacy: To determine if IV administration of CIS43LS at 40 mg/kg (compared to placebo) mediates protection against naturally occurring Pf infection in healthy Malian adults during a single malaria season as detected from microscopic examination of thick blood smear.

Secondary Objectives:

1. Dose escalation: To evaluate the pharmacokinetics (PK) of CIS43LS at each dose level throughout the study.

2. Efficacy: To determine if IV administration of CIS43LS at 40 mg/kg (compared to placebo) mediates protection against naturally occurring Pf infection in healthy Malian adults during a single malaria season as detected by polymerase chain reaction (PCR).
3. Efficacy: To evaluate the PK of CIS43LS throughout the study at the dose of 40 mg/kg and to correlate CIS43LS serum concentration with Pf infection risk.

Endpoints:

Primary Endpoints:

1. Dose escalation: Incidence and severity of local and systemic AEs occurring within 7 days after the administration of CIS43LS.
2. Efficacy: Pf blood stage infection as detected by microscopic examination of thick blood smear for 24 weeks after administration of CIS43LS or placebo.

Secondary Endpoints:

1. Dose escalation: Measurement of CIS43LS in sera of recipients.
2. Efficacy: Pf blood stage infection as detected by PCR for 24 weeks after administration of CIS43LS or placebo.
3. Efficacy: Measurement of CIS43LS in sera of recipients.

Study Population: Healthy Malian adults (aged 18 to 50 years) residing in Kalifabougou and Torodo

Phase: 2

Description of Sites/Facilities Enrolling Subjects: The MRTC clinics in Kalifabougou and Torodo. All scheduled study visits (including screening, enrollment, study agent administration, and follow-up) will be performed at Kalifabougou only. Both sites will conduct recruitment and unscheduled study visits.

Description of Study Intervention:

The study agent (CIS43LS or matching placebo) will be administered as a one-time IV infusion.

Dose-escalation study subjects will receive 1 of 3 different dose levels; study agent will be administered starting with the lowest dose level:

- Arm 1: 5 mg/kg IV
- Arm 2: 20 mg/kg IV
- Arm 3: 40 mg/kg IV

Efficacy study subjects will be randomized 1:1 to receive 1 of the following:

- Arm 1: 40 mg/kg IV
- Arm 2: Placebo (normal saline) IV

Study Duration: 21 months

Subject Duration:

Dose-escalation study: 8 months

Efficacy study: 8 months

1.2 SCHEMA

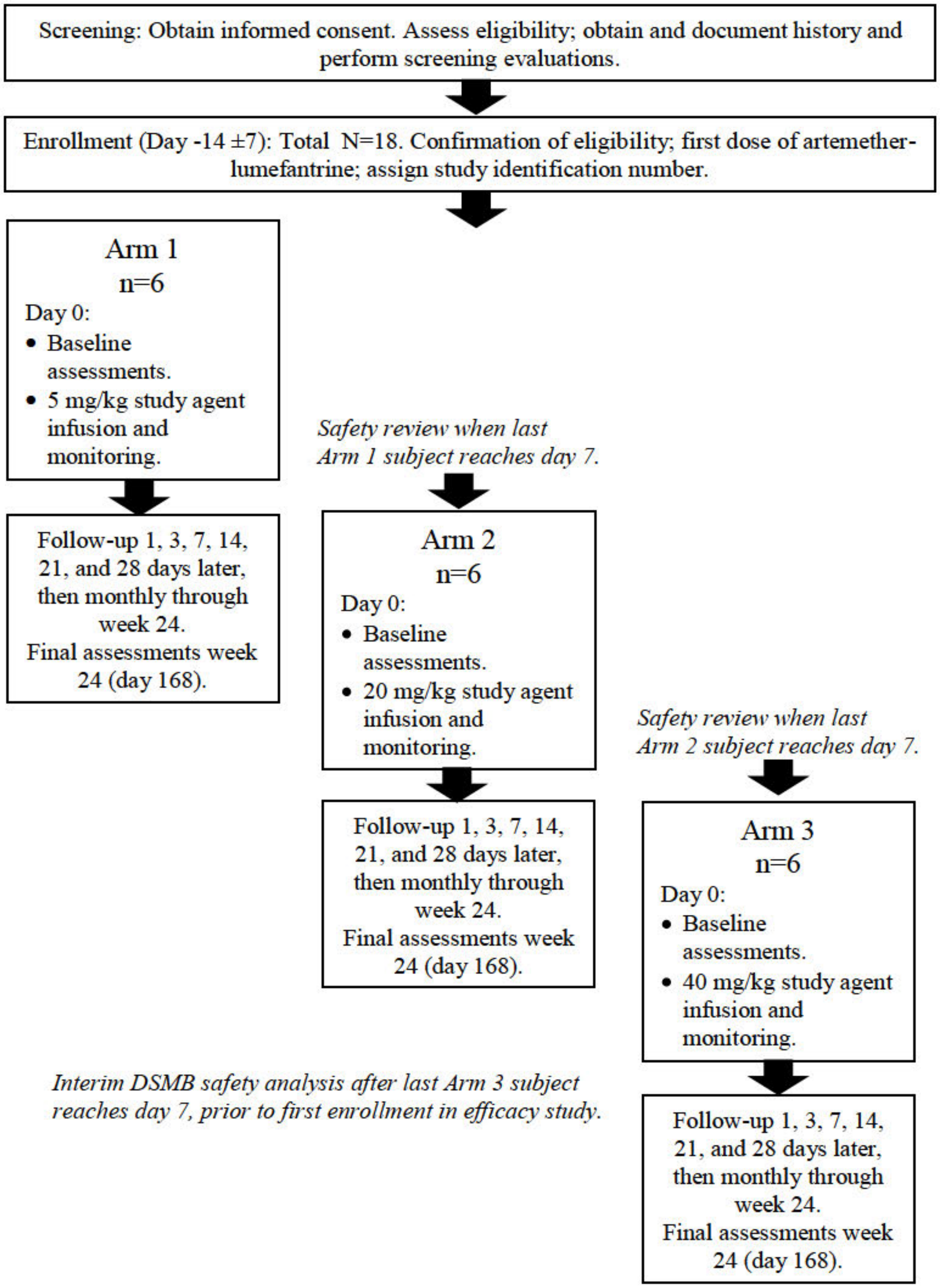

Figure 1. Dose-escalation study flow diagram.

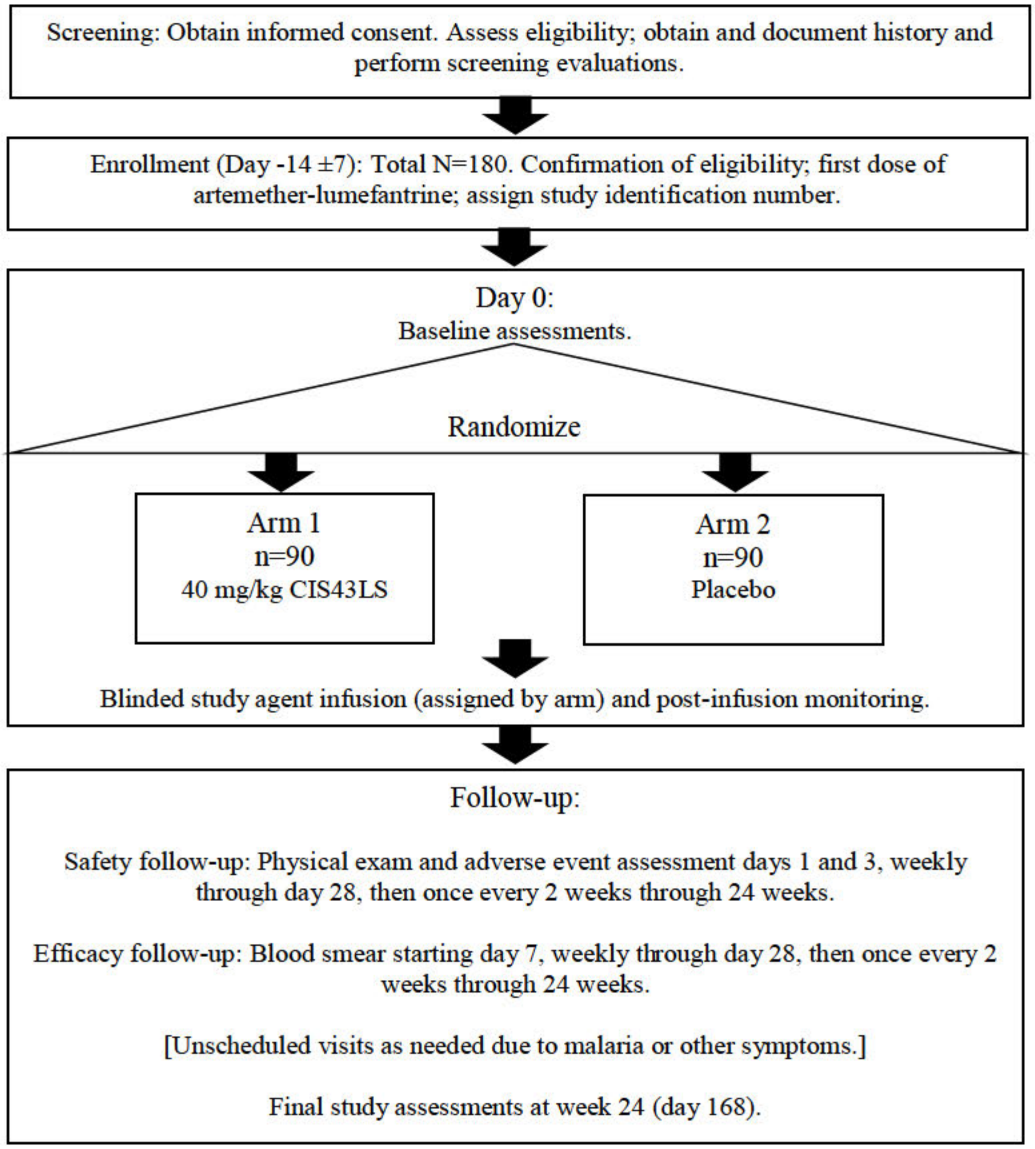

Figure 2. Efficacy study flow diagram.

1.3 SCHEDULE OF ACTIVITIES (SOA)

1.3.1 Dose-escalation Study

Study Day		Screen	Enroll[1]	0	1	3	7	14	21	28	56	84	112	140	168	Illness Visit	ET Visit
Window (days)		-56 to -7	-14±7	-	+1	±1	±2	±3	±3	±3	±7	±7	±7	±7	±7		
**Clinical Procedures/Evaluations**																	
Study comprehension exam		X															
Informed consent		X															
Confirmation of identity, age, and residency		X															
Physical exam [2]		X	X	X	X	X	X	X	X	X	X	X	X	X	X	X	X
Height		X															
Weight		X		X													
Vital signs (temperature, blood pressure, and pulse)		X		X	X	X	X	X	X	X	X	X	X	X	X	X	X
Medical history [2]		X	X	X	X	X	X	X	X	X	X	X	X	X	X	X	X
Concomitant medications [2]		X	X	X	X	X	X	X	X	X	X	X	X	X	X	X	X
Artemether-lumefantrine administration			X[3]														
ECG		X															
Study agent administration				X[4]													
Adverse events			X	X	X	X	X	X	X	X	X	X	X	X	X		X
Pregnancy prevention counseling		X	X	X	X	X	X	X	X	X	X	X	X	X	X		X
**Laboratory Evaluations**																	
Test	Tube	(Blood volume in mL)															
Urinalysis	-	X															
Pregnancy test (urine/serum; for women)	-	X	X	X[5]						X	X	X	X	X	X		
HIV, HBV, HCV screen [6]	SST	3															
CBC with differential	EDTA	3	3	3[5]		3	3	3									

**Study Day**		**Screen**	**Enroll**[1]	**0**	**1**	**3**	**7**	**14**	**21**	**28**	**56**	**84**	**112**	**140**	**168**	**Illness Visit**	**ET Visit**
Window (days)		-56 to -7	-14±7	-	+1	±1	±2	±3	±3	±3	±7	±7	±7	±7	±7		
Hemoglobin type	EDTA	(X)[7]															
ALT, Cr	SST	3	3	3[5]		3	3	3									
Finger prick blood smear and dried blood spot for Pf PCR[8]	-		X	X				X		X	X	X	X	X	X	X	
PK studies	SST			8[9]	4		4	4		4	4	4	4	4	4		
Serum storage	SST			8			8			8		8			8		
ADA	SST			(X)			(X)			(X)		(X)			(X)		
PBMC storage	CPT			16			16										
**Daily volume (mL)**		**9**	**6**	**38**	**4**	**6**	**34**	**10**	-	**12**	**4**	**12**	**4**	**4**	**12**		
**Cumulative volume (mL)**		**9**	**15**	**53**	**57**	**63**	**97**	**107**	**107**	**119**	**123**	**135**	**139**	**143**	**155**	-	-

Abbreviations: ADA, anti-drug antibodies; ALT, alanine transaminase; CBC, complete blood count; CPT, cell preparation tube; Cr, creatinine; ECG, electrocardiogram; EDTA, ethylenediaminetetraacetic acid; ELISA, enzyme-linked immunosorbent assay; ET, early termination; HBV, hepatitis B virus; HCV, hepatitis C virus; HIV, human immunodeficiency virus; PBMC, peripheral blood mononuclear cell; PCR, polymerase chain reaction; Pf, *Plasmodium falciparum*; PK, pharmacokinetics; RDT, rapid diagnostic test; SST, serum-separating tube.

(X) indicates that no additional blood will be drawn; the test will be performed from the tube shown in the preceding row.

Notes:

- Screening, enrollment, day 0, and all follow-up visits will take place at Kalifabougou for all subjects, regardless of residence.
- At any time during the study, the subject may have an unscheduled illness visit if experiencing malaria symptoms or other symptoms. The subject may be referred for standard care according to local guidelines. Unscheduled illness visits will take place at Kalifabougou or Torodo, depending on the subject's residence.

Footnotes:

[1] If enrollment is within 2 days of screening, duplicate procedures will not be repeated.
[2] Complete/comprehensive at screening; targeted/interim at other visits.
[3] Enrollment is defined as the time of first artemether-lumefantrine administration. (For women, negative pregnancy test must be confirmed prior to administration/enrollment.) The first dose will be directly observed in the clinic. The subsequent 5 doses given over 3 days will be observed by guides in the subject's home or at the clinic.
[4] All other study procedures must be completed prior to study agent infusion. Subject will be monitored during and after each infusion, and vital signs will be recorded directly after infusion and hourly during post-infusion monitoring. The first subject in a dose arm will be monitored for at least 4 hours after completion of infusion; all other subjects will be monitored for at least 2 hours after completion of infusion. Prior to discharge, subject will be assessed for local and systemic reactogenicity. If the subject is assessed as being unwell or has ongoing reactogenicity symptoms, he or she will be asked to remain in the clinic until evaluation and discharge by a study clinician.
[5] Collected prior to study agent infusion. For women, negative pregnancy test must be confirmed prior to infusion. Other indicated tests may be performed after infusion.
[6] Viral screenings will be performed according to international guidelines. HIV testing will be 2 rapid diagnostic tests (RDTs), plus enzyme-linked immunosorbent assay (ELISA) if the RDTs are discordant; subject will be referred for medical care for 2 positive RDTs or a positive ELISA. Pre- and post-test HIV counseling will be provided.

Study Day	Screen	Enroll[1]	0	1	3	7	14	21	28	56	84	112	140	168	Illness Visit	ET Visit
Window (days)	-56 to -7	-14±7	-	+1	±1	±2	±3	±3	±3	±7	±7	±7	±7	±7		

Hepatitis testing will be an HBV surface antigen test (ELISA) and HCV test (ELISA, PCR if indicated). A subject who is HBV and/or HCV positive will be referred for care regardless of the ALT result.

[7] EDTA tube will be stored at screening, and hemoglobin typing will be performed if the subject is enrolled; this test will not be used in eligibility assessments.

[8] If positive for malaria parasite infection, parasite genotyping may be performed.

[9] For PK, 4 mL serum will be collected prior to study agent infusion, and an additional 4 mL serum will be collected about 1 hour (±30 minutes) after the infusion.

1.3.2 Efficacy Study

Study Day	Screen	Enroll[1]	0	1	3	7	14	21	28	42	56	70	84	98	112	126	140	154	168	Illness Visit	ET
Window (days)	-56 to -7	-14±7	-	+1	±1	±2	±3	±3	±3	±7	±7	±7	±7	±7	±7	±7	±7	±7	±7		
**Clinical Procedures/Evaluations**																					
Study comprehension exam	X																				
Informed consent	X																				
Confirmation of identity, age, and residency	X																				
Physical exam[2]	X	X	X	X	X	X	X	X	X	X	X	X	X	X	X	X	X	X	X	X	X
Height	X																				
Weight	X		X																		
Vital signs (temperature, blood pressure, and pulse)	X		X	X	X	X	X	X	X	X	X	X	X	X	X	X	X	X	X	X	X
Medical history[2]	X	X	X	X	X	X	X	X	X	X	X	X	X	X	X	X	X	X	X	X	X
Concomitant medications[2]	X	X	X	X	X	X	X	X	X	X	X	X	X	X	X	X	X	X	X	X	X
Artemether-lumefantrine administration		X[3]																			
ECG	X																				
Randomization			X																		
Study agent administration			X[4]																		

**Study Day**		**Screen**	**Enroll** [1]	**0**	**1**	**3**	**7**	**14**	**21**	**28**	**42**	**56**	**70**	**84**	**98**	**112**	**126**	**140**	**154**	**168**	**Illness Visit**	**ET**
Window (days)		-56 to -7	-14±7	-	+1	±1	±2	±3	±3	±3	±7	±7	±7	±7	±7	±7	±7	±7	±7	±7		
Adverse events			X	X	X	X	X	X	X	X	X	X	X	X	X	X	X	X	X	X		X
Pregnancy prevention counseling		X	X	X	X	X	X	X	X	X	X	X	X	X	X	X	X	X	X	X		X
**Laboratory Evaluations**																						
Test	Tube	(Blood volume in mL)																				
Urinalysis	-	X																				
Pregnancy test (urine/serum; for women)	-	X	X	X [5]						X		X		X		X		X		X		
HIV, HBV, HCV screen [6]	SST	3																				
CBC with differential	EDTA	3	3	3 [5]		3	3	3														
Hemoglobin type	EDTA	(X) [7]																				
ALT, Cr	SST	3	3	3 [5]		3	3	3														
Finger prick blood smear and dried blood spot for Pf PCR [8]	-		X	X		X	X	X	X	X	X	X	X	X	X	X	X	X	X	X	X	
PK studies	SST			8 [9]	4		4	4		4		4		4		4		4		4		
Serum storage	SST			8			8			8				8						8		
ADA	SST			(X)			(X)			(X)				(X)						(X)		
PBMC storage	CPT			16				16														
**Daily volume (mL)**		**9**	**6**	**38**	**4**	**6**	**18**	**26**	-	**12**	-	**4**	-	**12**	-	**4**	-	**4**	-	**12**		
**Cumulative volume (mL)**		**9**	**15**	**53**	**57**	**63**	**81**	**107**	**107**	**119**	**119**	**123**	**123**	**135**	**135**	**139**	**139**	**143**	**143**	**155**	-	-

Abbreviations: ADA, anti-drug antibodies; ALT, alanine transaminase; CBC, complete blood count; CPT, cell preparation tube; Cr, creatinine; ECG, electrocardiogram; EDTA, ethylenediaminetetraacetic acid; ELISA, enzyme-linked immunosorbent assay; ET, early termination; HBV, hepatitis B virus; HCV, hepatitis C virus; HIV, human immunodeficiency virus; PBMC, peripheral blood mononuclear cell; PCR, polymerase chain reaction; Pf, *Plasmodium falciparum*; PK, pharmacokinetics; SST, serum-separating tube; RDT, rapid diagnostic test.

(X) indicates that no additional blood will be drawn; the test will be performed from the tube shown in the preceding row.

Study Day	Screen	Enroll[1]	0	1	3	7	14	21	28	42	56	70	84	98	112	126	140	154	168	Illness Visit	ET
Window (days)	-56 to -7	-14±7	-	+1	±1	±2	±3	±3	±3	±7	±7	±7	±7	±7	±7	±7	±7	±7	±7		

Notes:

- Screening, enrollment, day 0, and all follow-up visits will take place at Kalifabougou for all subjects, regardless of residence.
- At any time during the study, the subject may have an unscheduled illness visit if experiencing malaria symptoms or other symptoms. The subject may be referred for standard care according to local guidelines. Unscheduled illness visits will take place at Kalifabougou or Torodo, depending on the subject's residence.

Footnotes:

[1] If enrollment is within 2 days of screening, duplicate procedures will not be repeated.

[2] Complete/comprehensive at screening; targeted/interim at other visits.

[3] Enrollment is defined as the time of first artemether-lumefantrine administration. (For women, negative pregnancy test must be confirmed prior to administration/enrollment.) The first dose will be directly observed in the clinic. The subsequent 5 doses given over 3 days will be observed by guides in the subject's home or at the clinic.

[4] All other study procedures must be completed prior to study agent infusion. Subject will be monitored during and for 60 minutes after each infusion, and vital signs will be recorded directly and 1 hour after infusion. Prior to discharge, subject will be assessed for local and systemic reactogenicity. If the subject is assessed as being unwell or has ongoing reactogenicity symptoms, he or she will be asked to remain in the clinic until evaluation and discharge by a study clinician.

[5] Collected prior to study agent infusion. For women, negative pregnancy test must be confirmed prior to infusion. Other indicated tests may be performed after infusion.

[6] Viral screenings will be performed according to international guidelines. HIV testing will be 2 rapid diagnostic tests (RDTs), plus enzyme-linked immunosorbent assay (ELISA) if the RDTs are discordant; subject will be referred for medical care for 2 positive RDTs or a positive ELISA. Pre- and post-test HIV counseling will be provided. Hepatitis testing will be an HBV surface antigen test (ELISA) and HCV test (ELISA, PCR if indicated). A subject who is HBV and/or HCV positive will be referred for care regardless of the ALT result.

[7] EDTA tube will be stored at screening, and hemoglobin typing will be performed if the subject is enrolled; this test will not be used in eligibility assessments.

[8] If positive for malaria parasite infection, parasite genotyping may be performed.

[9] For PK, 4 mL serum will be collected prior to randomization and study agent infusion, and an additional 4 mL serum will be collected about 1 hour (±30 minutes) after the infusion.

2 INTRODUCTION

2.1 Study Rationale

Malaria is a mosquito-borne protozoan disease belonging to the genus *Plasmodium* that affects approximately 219 million people and kills approximately 435,000 individuals annually, with an enormous economic impact in the developing world, especially sub-Saharan Africa.[1] The 5 recognized species of *Plasmodium* that cause human malaria infection are Pf, *P. vivax, P. ovale, P. malariae,* and *P. knowlesi*. Among these, Pf causes more deaths in children worldwide than any other single infectious agent. An estimated 30,000 travelers from North America, Europe, and Japan contract malaria per year. Although malaria is preventable with chemoprophylaxis and completely curable with early intervention, drug treatment is not readily accessible in many parts of the world. Additionally, the use of antimalarial drugs over time has been associated with the emergence of drug-resistant strains. Lack of compliance with preventive drug treatment by individuals travelling to endemic areas may also result in fatal malaria infection. The world's first malaria vaccine, RTS,S/AS01 (Mosquirix™), a recombinant protein-based vaccine targeting Pf, was approved for use by European regulatory authorities in 2015. It is currently being evaluated in immunization programs in sub-Saharan Africa despite having been found to provide only partial protection against malaria to children and infants.[2,3] Therefore, the development of safe and effective malaria vaccines or protective antibodies for complete prevention and ultimate elimination of malaria remains an urgent unmet medical need with the potential to have a major impact on improving public health worldwide.

The VRC, NIAID, NIH, has developed a monoclonal antibody (MAb) called CIS43LS as a possible preventive therapeutic against Pf infection. CIS43LS is currently being evaluated in an ongoing phase 1, first-in-human study in healthy volunteers at the VRC. As a follow-up to the VRC study, this protocol is designed to evaluate the safety and efficacy of CIS43LS in healthy adults in Mali, where Pf malaria infection is endemic.

2.2 Background

2.2.1 Overview

The VRC, NIAID, has been investigating broadly-neutralizing human MAbs targeting a variety of pathogens that may be utilized in clinical applications including preventive and therapeutic measures. In the case of malaria, prevention by passive immunization has potential applications for use in travelers, military personnel, season control, and elimination campaigns in endemic areas.

VRC investigators isolated a MAb, termed CIS43, from a subject immunized with an attenuated Pf whole-sporozoite (SPZ) vaccine (PfSPZ Vaccine [Sanaria]). The MAb was then adapted using site-directed mutagenesis in the Fc region, resulting in an LS mutation (CIS43LS) that has been found to increase product half-life in plasma,[4,5] which will be critical for optimizing its use. This LS modification has been applied to multiple VRC clinical MAbs and has been shown to be safe and significantly increase the half-life in plasma.[6, and unpublished data]

CIS43LS recognizes a unique and conserved epitope at the junction of the N- and repeat regions of the Pf circumsporozoite protein (CSP). In a published study in 2 different mouse models of

malaria infection, passive immunization with CIS43 conferred high-level protection (70%-100%) following malaria challenge with concentrations of antibody *in vivo* ranging between 10-200 μg/mL respectively.[7] Similar protection data were observed with CIS43LS (unpublished). These data show that CIS43LS is highly protective *in vivo* and suggest further exploration was warranted for passive prevention of malaria in humans.

This protocol is a two-part phase 2 trial evaluating the safety and tolerability of one-time administration of CIS43LS in healthy Malian adults as well as its protective efficacy against naturally occurring Pf infection over a 6-month malaria season.

2.2.2 Study Product: CIS43LS

MAb VRC-MALMAB0100-00-AB (CIS43LS) was discovered and developed by the VRC, NIAID, NIH. CIS43LS binds a unique and conserved epitope at the junction of the N- and repeat regions of the Pf CSP. The study product was manufactured under current Good Manufacturing Practice (GMP) by the Vaccine Clinical Materials Program (VCMP) operated under contract by Leidos Biomedical Research, Inc., Frederick, MD.

2.2.2.1 Previous Human Experience

A separate phase 1 trial of CIS43LS is currently ongoing at the VRC. The study is a two-part, dose-escalation, adaptive design study evaluating the safety, tolerability, PK, and protective efficacy of CIS43LS. Doses being evaluated range from 5 mg/kg subcutaneously (SC) to 40 mg/kg IV. The second part of the VRC study involves assessing protective efficacy by controlled human malarial infection. Initial safety data is planned to be available in March 2020.

Prior clinical experience in completed and ongoing phase 1 and 2 trials of healthy adults with other human MAbs manufactured and formulated by the VRC that recognize pathogen-specific epitopes (i.e., VRC01, VRC01LS, VRC07-523LS, and MAb114) [6,8-10] are used to summarize the general safety risk associated with MAbs.

Treatments with these MAbs have been generally well tolerated, with no reported deaths or serious adverse events (SAEs) assessed as related to the study products. Typical for MAbs, the predominant local reactogenicity complaint has been mild pain/tenderness, although reports of mild injection-site pruritus, redness and swelling have occurred at modestly higher frequencies with SC administration. Malaise, muscle pain, and headache have been the most frequently reported solicited complaints noted in the 3 days after product administration, and these have also been mostly transient and mild in severity. Urticaria and infusion reactions including chills, rigors, myalgia, headache, and/or fever have been reported after IV infusions at product doses of 10 to 40 mg/kg; these reactions have been transient, resolved without sequelae within 24 hours of onset, and were treated with over-the-counter analgesics and antipyretics.

2.2.2.2 Preclinical Experience

To assess CIS43LS as a candidate for clinical trials, recombinant, research grade MAb was evaluated on binding properties, auto-reactivity, PK, 2 mouse models of *in vivo* protection following challenge, and PK studies in nonhuman primates. In mice, CIS43LS conferred 100% protection following mosquito bite challenge. In nonhuman primate PK studies, CIS43LS

exhibited substantially longer half-life, both in blood and in skin biopsy samples as compared to CIS43 antibody without the LS mutation.

Preclinical toxicology studies were conducted to assess safety in compliance with current Good Laboratory Practices (GLP) in Sprague Dawley rats using CIS43LS. The toxicology pre-clinical material was tested in the final formulation buffer and manufactured using the same VCMP manufacturing process as used to produce clinical trial material. There were no findings of toxicologic significance attributed to CIS43LS from the results.

2.2.2.2.1 *In vitro* Studies

Differences in the binding properties of CIS43LS and CIS43 were assessed. Binding of CIS43LS and CIS43 to a specific target antigen expressed on malaria parasites was determined by enzyme-linked immunosorbent assay (ELISA). CIS43LS and CIS43 exhibited similar binding to recombinant Pf circumsporozoite protein (rPfCSP). Avidity and stoichiometry of CIS43LS and CIS43 binding to rPfCSP were also comparable as assessed by isothermal titration calorimetry.

CIS43LS autoreactivity was assessed using the anti-nuclear antibody (ANA) HEp2 cell staining system from Zeus Scientific (Branchburg, NJ). The assay was conducted according to the manufacturer's instructions. Antibodies were tested at 25 and 50 mcg/mL. At 25 mcg/mL, test antibodies were scored in comparison to negative and positive control samples. Neither CIS43 nor CIS43LS shows an autoreactivity signal by ANA staining. Autoreactivity was also assessed by an anti-cardiolipin QUANTA Lite ACA immunoglobulin (Ig) GIII ELISA (Inova Diagnostics, San Diego, CA). Using a similar control schema to that described for ANA HEp2 cell staining, the assay was conducted according to the manufacturer's instructions.

The potential for CIS43LS, a MAb directed against Pf CSP on the surface of PfSPZ, to cross-react with normal human and Sprague-Dawley rat tissues was assessed in a GLP study (Charles River Laboratories, Inc., Frederick, MD, Study No. 20188989) by immunohistochemical staining of tissue cryosections with CIS43LS or appropriate controls. The human and rat tissue panels evaluated included all of the tissues on the "suggested list of human tissues to be used for immunohistochemical or cytochemical investigations of cross reactivity of monoclonal antibodies" in Annex I of the European Medicines Agency document "Guideline on Development, Production, Characterization and Specifications for Monoclonal Antibodies and Related Products," adopted by the Committee for Medicinal Products for Human Use, and all of the tissues recommended in the US Food and Drug Administration (FDA) Center for Biologics Evaluation and Research document, "Points to Consider in the Manufacture and Testing of Monoclonal Antibody Products for Human Use." The only difference between the 2 species in tissue types evaluated was tonsil; inasmuch as rats do not have tonsils, tonsil tissue was evaluated from humans only. To detect binding, CIS43LS was applied to cryosections of normal human tissues from 3 donors per human tissue, except for bladder, for which only 2 were available. Samples from at least 2 individual rats were evaluated except for fallopian tube, parathyroid, and ureter, for which only 1 was available. All tissues were incubated with test or control article at 2 concentrations (1 µg/mL and 10 µg/mL). As a negative control, a monoclonal human $IgG1_{\kappa}$ antibody, HuIgG1 (control article), with a different antigenic specificity from that of CIS43LS, was tested in parallel. Other controls included omission of the test or control articles from the assay (assay controls). At both concentrations, CIS43LS produced moderate to intense membrane and cytoplasmic staining of positive control HEK293 cells, which expressed

the Pf CSP epitope targeted by CIS43LS, and did not specifically react with the negative control material, cryosections of negative HEK293 cells, at either staining concentration. The control article, HuIgG1, did not specifically react with either the positive or negative control materials, and there was similarly no staining of the assay control slides. The specific reactions of CIS43LS with the positive control cells in all staining runs, the absence of specific reactivity with the negative control cells, and the lack of reactivity of the control article indicated that the assay was sensitive, specific, and reproducible. CIS43LS produced membrane and cytoplasmic staining of epithelium in sebaceous glands and cytoplasmic staining of epithelium in sweat glands in the human skin. This staining was weak to strong in intensity and frequently observed in sebaceous glands and rarely or occasionally observed in sweat glands at the higher concentration of CIS43LS, with a reduction in the intensity of the staining at the lower concentration. No other staining was observed with CIS43LS in the human tissue panel. Similarly to its interaction with human tissue, CIS43LS weakly stained the membrane and cytoplasm of occasional to frequent sebaceous gland epithelium in the rat skin at the higher concentration only. No other staining was observed with CIS43LS in the rat tissue panel. The consonance of results between human and rat tissues confirm the appropriateness of the rat model for the CIS43LS toxicity study.

2.2.2.2.2 *In vivo* Studies

2.2.2.2.2.1 Protection studies in C57BL/6 mice and Rhesus macaques

To assess protection following passive transfer of MAb, naïve C57BL/6 mice received 300 mcg by IV inoculation of CIS43, CIS43LS, or negative control antibody. Chimeric parasites comprising *Plasmodium berghei* (Pb) SPZ which naturally infect mice that expressed Pf CSP (Pb-PfCSP SPZ) were administered by 1 of 2 routes (injected by tail vein or by exposure to bites by infected mosquitos). In the 2 mouse challenge models, passive transfer of CIS43 and CIS43LS yielded nearly indistinguishable results. Administration of either CIS43 or CIS43LS reduced liver parasite burden by about 2 logs when challenged by IV route. Moreover, passive transfer of CIS43 or CIS43LS conferred 100% sterile protection in mice as assessed by parasitemia in blood following a natural challenge by 5 mosquito bites in the skin. This type of challenge is similar to what is used in the controlled human malaria infection (CHMI) procedures at the VRC.

To inform clinical protocol development, particularly concerning the timing of CHMI, a 20-week PK study was conducted in nonhuman primates (Rhesus macaques). Two animals per group received CIS43 or CIS43LS, administered by IV route at a dose of 10 mg/kg. CIS43LS exhibited substantially longer half-life, both in blood and skin biopsy samples than CIS43. In blood, CIS43LS maintained a titer of more than 35 mcg/mL on the last sample day (day 140). In contrast, CIS43 dropped to approximately 30 mcg/mL by day 49. Skin biopsy data were concordant.

2.2.2.2.2.2 Toxicology Studies in Sprague Dawley Rats

A study of repeat dosing at 10-day intervals by IV and SC administration of CIS43LS in male and female Sprague Dawley rats (SRI Study No. M416-19) was conducted in compliance with current GLP to evaluate toxicity and toxicokinetics. The test article and control (formulation buffer) were administered on days 1 and 11 by IV administration at doses of 0, 40, or

400 mg/kg/day (Groups 1−3, respectively) or by SC administration at doses of 5 or 50 mg/kg/day (Groups 4−5, respectively). Necropsies were performed on days 12 and 46. Endpoints included clinical observations, body weight, food consumption, body temperature, dose site irritation, clinical pathology, and organ weights.

All animals survived to their scheduled sacrifice timepoint. No test article-related changes were observed in clinical observations, body weights, food consumption, dose site irritation, clinical chemistry, coagulation, and organ weights. Body temperature was normal (≤101.0°F) for all males at 24 hours post-dose. Elevated body temperatures ranging from 101.1 to 102.1°F were seen from 1 or more females in high-dose IV or both SC treated groups for at least 24 hours after dosing on days 1 and/or 11. Body temperature returned to normal levels within 48 hours post-dose. These findings are considered test article-related although reversible and at levels considered to be non-adverse.

Statistically significant and dose-dependent changes in some hematology parameters were observed on days 12 and 46. The changes included increased platelet count (1.4-fold) and absolute reticulocyte count (1.2-fold) in Group 5 females, and decreased percent monocyte (30.6% to 32.2%) in Group 3 and 4 females on day 12. On day 46, increased absolute neutrophils (2.0-to 2.3-fold) in Group 3 and 4 males and decreased hemoglobin (6.1% to 7.6%) and/or hematocrit (5.6% to 7.6%) in all treated-group females were observed. The changes in neutrophils and monocytes seen after SC administration are consistent with an immune response and are considered test article-related although reversible and at levels considered to be non-adverse. Without changes in red blood cell counts, the findings in platelet count, reticulocytes, hemoglobin, or hematocrit are not considered to be biologically meaningful or toxicologically adverse. In summary, no findings of toxicologic significance were attributed to CIS43LS from the results available as of this report.

Taken together, these studies support the continued development of CIS43LS for clinical evaluation. The data confirm the predicted longer half-life effect of the LS mutation and show that there is no functional difference in terms of *in vitro* binding or *in vivo* protection compared to CIS43. No safety, efficacy, or manufacturability concerns were noted that precluded proceeding to further toxicology studies, GMP manufacturing, IND filing, and clinical investigations.

2.2.3 Laboratory Assessments of CIS43LS

Some laboratory assessments in this study are designed to characterize the investigational product. This includes PK analysis and evaluation for anti-drug antibody (ADA) development after product exposure. Other assays may also be completed from stored samples at a later date.

The LIG International Center of Excellence in Research Lab at the USTTB in Bamako will process blood and store coded samples and will either perform sample testing or ship coded samples to designated research laboratories at LIG/NIAID and VRC/NIAID or other approved collaborators. The key to the code will remain at the USTTB. See section 1.3 for schedules, blood volumes, and tube types to be used for research sample collection. Research assays will be performed on samples from both study product recipients and placebo controls at baseline and throughout the study.

Tube types for clinical labs are according to institutional requirements and are shown in section 1.3 to estimate blood volumes. Different tubes for clinical evaluations may be used to meet site requirements. Research sample tube types and blood volumes must be used as shown or as otherwise instructed by the manufacturer. In some instances, coded samples may be transported directly by study staff to the laboratory of an approved collaborator.

2.2.3.1 Pharmacokinetic Analysis

Concentrations of CIS43LS will be measured by a Meso Scale Discovery LLC–based automation platform and similar methodology as previously described for other VRC MAb products.[8]

2.2.3.2 Detection of Anti-Drug Antibodies

Assays for detection of ADA will be performed at specified timepoints following product administration and compared to baseline status using a similar methodology as previously described for other VRC MAb products.[8]

2.2.3.3 Measures of MAb-Mediated Protection and Parasitemia

CIS43LS-mediated protection against naturally occurring Pf infection during a single 6-month malaria season will be assessed and compared to control subjects. The endpoint defining MAb-mediated protection from Pf infection is the absence of Pf parasites in blood samples obtained from CIS43LS-recipients collected from day 7 through week 24 (day 168) after study agent administration. The criteria for a case of Pf infection is based on blood smear analysis.

Giemsa-stained thick blood films will be prepared and examined by trained personnel following the standard operating procedure (SOP) based on the standard World Health Organization (WHO) protocol.

Thick blood smears will be prepared from the blood remaining in the collection device, or (at timepoints when no blood collection is planned) from a finger prick sample. The smears will be examined microscopically. Thick blood smears will be used for diagnosis throughout the study.

Additionally, as a secondary endpoint, the Malaria Real-time PCR ABI 7500 Assay will be performed at the NIH Clinical Center Microbiology Service.[11-13]

Research blood samples may also be used for Pf malaria parasite genome analysis.

2.3 Risk/Benefit Assessment

2.3.1 Known Potential Risks

Risk of CIS43LS: At the time of the development of this protocol, there is no prior human experience with administration of CIS43LS and therefore risks are unknown at this time.[14] This study will not begin enrollment until initial safety data is reviewed under the ongoing phase 1 VRC trial. At that time, the protocol and consent will be updated prior to the initiation of this study if new information about risks and side effects becomes available.

Risks of MAb Administration: Administration of MAbs may cause immune reactions such as acute anaphylaxis, serum sickness, and the generation of antibodies. However, these reactions

are rare and more often associated with MAbs targeted to human proteins or with the use of mouse MAbs that would have a risk of human anti-mouse antibodies.[15] In this regard, because CIS43LS is targeted to a parasite antigen and is a human MAb, it is expected to have a low risk of such side effects.

Typically, the side effects of MAbs are mild to moderate and may include local reactions at the injection site (including pain, redness, bruising, swelling) and systemic reactions such as fever, chills, rigors, nausea, vomiting, pain, headache, dizziness, fatigue, shortness of breath, bronchospasm, hypotension, hypertension, pruritus, rash, urticaria, angioedema, diarrhea, tachycardia, or chest pain. Healthcare staff will be appropriately trained and necessary medical equipment will be readily available at the clinic where the study agent is administered. Clinical use of MAbs that are targeted to cytokines or antigens associated with human cells may be associated with an increased risk of infections[15]; however, this is not expected to be a risk for a MAb targeted to a parasite antigen.

Severe reactions, such as anaphylaxis, angioedema, bronchospasm, hypotension, and hypoxia, are infrequent and more often associated with MAbs targeted to human proteins or with non-human MAbs, such as a mouse MAb.[15] Most infusion-related events occur within the first 24 hours after initiation of MAb administration.

Published experience with human MAbs directed against cell surface targets on lymphocytes shows that infusion of a MAb may be associated with cytokine release, causing a reaction known as cytokine release syndrome (CRS).[16] CRS reactions commonly occur within the first few hours of infusion start and with the first MAb infusion received. This is because the cytokine release is associated with lysis of the cells targeted by the MAb and the burden of target cells is greatest at the time of the first MAb treatment. With licensed therapeutic MAbs, CRS is managed by temporarily stopping the infusion, administering histamine blockers and restarting the infusion at a slower rate.[17] Supportive treatment may also be indicated for some signs and symptoms.

Delayed allergic reactions that include a serum sickness type of reaction characterized by urticaria, fever, lymph node enlargement, and joint pains, typically occur several days after MAb exposure and are more commonly associated with chimeric types of MAbs.[15] In general, and with due consideration of the needs dictated by individual subject symptoms and treating clinician discretion, immediate and delayed reactions to study product will be managed according to the principles of the American Academy of Allergy, Asthma, and Immunology guidelines.[18]

Participation in this study may limit a subject's eligibility for future MAb studies.

Risks of Placebo Administration: There are no risks of the placebo (normal saline) other than infusion-related events such as headache, dizziness, and hypertension.

Risks of Blood Drawing or IV Placement: Drawing blood or placing an IV may cause pain, bruising, and a feeling of lightheadedness or fainting. Rarely, it may cause infection at the site where blood is taken.

Risks of Artemether-Lumefantrine Administration: Adverse reactions (ARs) occurring in more than 30% of adults are headache, anorexia, dizziness, asthenia, arthralgia, and myalgia. Reactions typically do not require stopping treatment. Individuals who may have any contraindication for the use of this drug (e.g., prolonged QTc) will be excluded at screening. In

postmarketing experience, serious hypersensitivity reactions including anaphylaxis and serious skin reactions (bullous eruption) have been reported. Individuals with known sensitivity or contraindications to the antimalarials administered in this study are excluded from participation.

A complete list of side effects and contraindications is provided in the package insert.[19]

2.3.2 Known Potential Benefits

Subjects in the dose-escalation study will not directly benefit from study participation, since the study is too early in the year to be expected to provide protection during the malaria transmission season. In the efficacy study, subjects may not receive direct health benefit from study participation. Depending on whether CIS43LS confers protective efficacy, subjects receiving a sufficient dose of CIS43LS may experience some protection against Pf infection during part or all of the malaria transmission season.

In the future, others may benefit from knowledge gained in this study that may aid in the development of malaria prevention.

2.3.3 Assessment of Potential Risks and Benefits

The study population lives in an area where malaria is endemic and so is at significant risk of malarial infection and disease. As described above, it is possible that some subjects may benefit from study participation by receiving some protection during the malaria season. Therefore, the value of the information that will be gained from this study for developing malaria prevention strategies justifies the potential risks of study participation described above. Additionally, potential risks are minimized by careful design of subject eligibility criteria and post-vaccination monitoring.

3 OBJECTIVES AND ENDPOINTS

OBJECTIVES	ENDPOINTS	JUSTIFICATION FOR ENDPOINTS
Primary		
1. Dose escalation: To evaluate the safety and tolerability of CIS43LS administered IV at 5, 20, and 40 mg/kg in healthy Malian adults.<br>2. Efficacy: To determine if IV administration of CIS43LS at 40 mg/kg (compared to placebo) mediates protection against naturally occurring Pf infection in healthy Malian adults during a single malaria season as detected from	1. Dose escalation: Incidence and severity of local and systemic AEs occurring within 7 days after the administration of CIS43LS.<br>2. Efficacy: Pf blood stage infection as detected by microscopic examination of thick blood smear for 24 weeks after administration of CIS43LS or placebo.	1. Assessment of AEs is a standard measure of study agent safety and tolerability.<br>2. Blood smear is the gold standard for diagnosis of blood-stage Pf infection.

OBJECTIVES	ENDPOINTS	JUSTIFICATION FOR ENDPOINTS
microscopic examination of thick blood smear.		
Secondary		
1. Dose escalation: To evaluate the PK of CIS43LS at each dose level throughout the study.<br>2. Efficacy: To determine if IV administration of CIS43LS at 40 mg/kg (compared to placebo) mediates protection against naturally occurring Pf infection in healthy Malian adults during a single malaria season as detected by PCR.<br>3. Efficacy: To evaluate the PK of CIS43LS throughout the study at the dose of 40 mg/kg and to correlate CIS43LS serum concentration with Pf infection risk.	1. Dose escalation: Measurement of CIS43LS in sera of recipients.<br>2. Efficacy: Pf blood stage infection as detected by PCR for 24 weeks after administration of CIS43LS or placebo.<br>3. Efficacy: Measurement of CIS43LS in sera of recipients.	1. Concentrations of CIS43LS in blood will help assess durability of CIS43LS at each dose level.<br>2. PCR is more sensitive than blood smear for detecting Pf blood-stage infection.<br>3. Concentrations of CIS43LS in blood will help assess durability of CIS43LS and will allow for correlation with Pf infection risk.
Tertiary/Exploratory		
1. To determine whether ADA to CIS43LS can be detected in sera of recipients at specific timepoints throughout the study.<br>2. To assess for IgG1 allotypes and allotype-specific effects on CIS43LS PK.<br>3. To explore and characterize the cellular immune response to CIS43LS.<br>4. To determine if the efficacy of CIS43LS is specific to certain Pf parasite genotypes at the CSP locus.<br>5. To explore the impact of pre-existing parasitemia on the	1. Measurement of ADA to CIS43LS in sera of recipients.<br>2. Assessment of IgG1 allotypes and allotype-specific effects on CIS43LS PK.<br>3. Characterization of the cellular immune response to CIS43LS.<br>4. CSP genotyping of parasites isolated from study subjects.<br>5. Pre-existing parasitemia detected by microscopic examination of thick blood smears or PCR before CIS43LS infusion.<br>6. Pre-existing CSP-specific antibodies measured in sera collected before CIS43LS infusion.	1. ADA to CIS43LS may impact the PK and activity of CIS43LS.<br>2. Subject IgG1 allotype may impact the PK and activity of CIS43LS.<br>3. CIS43LS-induced cellular immune responses may be associated with Pf infection risk.<br>4. CIS43LS efficacy may be specific to certain Pf parasite

OBJECTIVES	ENDPOINTS	JUSTIFICATION FOR ENDPOINTS
protective efficacy and PK of CIS43LS.<br>6. To explore the impact of pre-existing CSP antibodies on the protective efficacy and PK of CIS43LS.		genotypes at the CSP locus.<br>5. Pre-existing parasitemia may impact the protective efficacy and PK of CIS43LS.<br>6. Pre-existing CSP-specific antibodies may impact the protective efficacy and PK of CIS43LS.

4 STUDY DESIGN

4.1 Overall Design

This is a two-part, phase 2 trial evaluating the safety and tolerability of one-time administration of CIS43LS, as well as its protective efficacy against naturally occurring Pf infection over a 6-month malaria season. Initial safety data from the ongoing VRC phase 1 trial of CIS43LS will be reviewed prior to starting enrollment for this study; if needed, the protocol and relevant study documents will be amended to reflect any new information.

The primary study hypotheses are that CIS43LS will be safe and will confer protection against Pf infection. The study will recruit from 2 MRTC clinics, 1 in Torodo and 1 in Kalifabougou. All scheduled study visits will take place at Kalifabougou for all subjects, regardless of residence.

The rural village of Kalifabougou is situated 46 km from the MRTC laboratory in Bamako where biological samples collected for this protocol will be processed and stored. Torodo is located 12 km north of Kalifabougou. The economy is based on subsistence farming. Kalifabougou and Torodo are similar in terms of geographic, demographic, and epidemiological characteristics, and both typically experience intense seasonal Pf transmission from July through December each year.[20] Based on data collected in Kalifabougou from 2011 through 2018, 68%-90% of adults who are uninfected (PCR negative) before the malaria season become infected with Pf during the ensuing 6-month malaria season.[20]

The dose-escalation study is an open-label study for safety and tolerability. Subjects will be assigned to 1 of 3 dose arms. Dosing will begin in the lowest dose arm and will escalate sequentially as described in section 6.1.2.1. Subjects will be followed for safety to assess AEs at

study visits 1, 3, 7, 14, 21, and 28 days after administration, then monthly for about 6 months after administration.

After the final subject in the dose-escalation study reaches day 7 safety follow-up, an interim safety evaluation will be performed by the DSMB before enrollment begins for the efficacy study (section 9.4.8).

The efficacy study is a randomized, double-blind, placebo-controlled trial (N=180 total, n=90 for each of 2 treatment arms) to assess safety and protective efficacy of a dose arm and a placebo arm. Subjects will receive 1 dose of study agent and be followed at regular study visits for about 6 months. Primary study assessments include physical examination and blood collection for research laboratory evaluations, including assessment of protection against naturally occurring Pf infection over 6 months (a single malaria season). The primary endpoint is the absence of Pf parasites in blood smears obtained between 1 week and 6 months after study agent administration in recipients of CIS43LS and placebo.

4.2 Scientific Rationale for Study Design

This two-part study was designed to test CIS43LS in the setting of naturally occurring Pf infection and in a population that could potentially benefit from a novel therapeutic for malaria prevention.

The efficacy study will use both randomization and a double blind to minimize bias in subject selection and study assessments. The placebo will be inactive (normal saline) rather than a comparator MAb, as currently there are no licensed anti-malaria MAbs available.

4.3 Justification for Dose

The doses used in this study were selected based off preclinical data and will complement the data generated in the ongoing phase 1 VRC study, which will test all dose levels included in this protocol. The dose used in the efficacy study (40 mg/kg IV) will complement the data generated in the ongoing phase 1 VRC study. This dose was derived from 1) efficacy data from the challenge studies performed in mice showing that the protective concentration of antibody *in vivo* is between 10-200 µg/mL in 2 different mouse models of malaria infection; and 2) PK data from nonhuman primate studies with CIS43LS and prior clinical experience in healthy adults with human MAbs targeting HIV (i.e., VRC01, VRC01LS, and VRC07-523LS) and ebolavirus (MAb114) at the same dose.

5 STUDY POPULATION

5.1 Inclusion Criteria

Individuals must meet all of the following criteria to be eligible for study participation:

1. Aged ≥18 and ≤50 years.
2. Able to provide proof of identity to the satisfaction of the study clinician completing the enrollment process.
3. In good general health and without clinically significant medical history.
4. Able to provide informed consent.

5. Willing to have blood samples and data stored for future research.
6. Resides in or near Kalifabougou or Torodo, Mali, and available for the duration of the study.
7. Females of childbearing potential must be willing to use reliable contraception from 21 days prior to study day 0 through the final study visit as described below.
    a. Reliable methods of birth control include 1 of the following: confirmed pharmacologic contraceptives via parenteral delivery or intrauterine or implantable device.
    b. Nonchildbearing women will be required to report date of last menstrual period, history of surgical sterility (i.e., tubal ligation, hysterectomy) or premature ovarian insufficiency, and will have urine or serum pregnancy test performed per protocol.

5.2 Exclusion Criteria

Individuals meeting any of the following criteria will be excluded from study participation:

1. Pregnancy, as determined by a positive urine or serum beta-human choriogonadotropin (β-hCG) test (if female).
2. Currently breastfeeding.
3. Behavioral, cognitive, or psychiatric disease that in the opinion of the investigator affects the ability of the subject to understand and comply with the study protocol.
4. Study comprehension examination score of <80% correct or per investigator discretion.
5. Hemoglobin, white blood cell, absolute neutrophil, or platelet count outside the local laboratory–defined limits of normal. (Subjects may be included at the investigator's discretion for "not clinically significant" values.)
6. Alanine transaminase (ALT) or creatinine (Cr) level above the local laboratory–defined upper limit of normal. (Subjects may be included at the investigator's discretion for "not clinically significant" values.)
7. Infected with human immunodeficiency virus (HIV), hepatitis C virus (HCV), or hepatitis B virus (HBV).
8. Known or documented sickle cell disease by history. (Note: Known sickle cell trait is NOT exclusionary.)
9. Clinically significant abnormal electrocardiogram (ECG; corrected QT interval [QTc] >460 or other significant abnormal findings, including unexplained tachycardia or bradycardia).
10. Evidence of clinically significant neurologic, cardiac, pulmonary, hepatic, endocrine, rheumatologic, autoimmune, hematological, oncologic, or renal disease by history, physical examination, and/or laboratory studies including urinalysis.
11. Receipt of any investigational product within the past 30 days.
12. Participation or planned participation in an interventional trial with an investigational product until the last required protocol visit. (Note: Past, current, or planned participation in observational studies is NOT exclusionary.)
13. Medical, occupational, or family problems as a result of alcohol or illicit drug use during the past 12 months.
14. History of a severe allergic reaction or anaphylaxis.

15. Severe asthma (defined as asthma that is unstable or required emergent care, urgent care, hospitalization, or intubation during the past 2 years, or that has required the use of oral or parenteral corticosteroids at any time during the past 2 years).
16. Pre-existing autoimmune or antibody-mediated diseases including but not limited to: systemic lupus erythematosus, rheumatoid arthritis, multiple sclerosis, Sjögren's syndrome, or autoimmune thrombocytopenia.
17. Known immunodeficiency syndrome.
18. Known asplenia or functional asplenia.
19. Use of chronic (≥14 days) oral or IV corticosteroids (excluding topical or nasal) at immunosuppressive doses (i.e., prednisone >10 mg/day) or immunosuppressive drugs within 30 days of day 0.
20. Receipt of a live vaccine within the past 4 weeks or a killed vaccine within the past 2 weeks prior to study agent administration.
21. Receipt of immunoglobulins and/or blood products within the past 6 months.
22. Previous receipt of an investigational malaria vaccine in the last 5 years.
23. Known allergies or contraindication against artemether-lumefantrine.
24. Other condition(s) that, in the opinion of the investigator, would jeopardize the safety or rights of a subject participating in the trial, interfere with the evaluation of the study objectives, or render the subject unable to comply with the protocol.

5.3 Inclusion or Exclusion of Vulnerable Participants

- **Children:** Children are not eligible to participate in this clinical trial because CIS43LS has not been previously evaluated in children to be safe. If the product is assessed as safe for further study, other protocols specifically designed for children may be conducted.
- **Pregnant Women:** Pregnant women are excluded from this study because the effects of CIS43LS on pregnant women or the developing human fetus are unknown with the potential for teratogenic or abortifacient effects.
- **Decisionally Impaired Adults:** Individuals must be able to provide consent in order to be eligible for participation in this study. Enrolled subjects who lose decision-making capability during study participation will be withdrawn (see sections 8.4.3 and 10.1.5).
- **Illiterate Individuals:** We anticipate that many individuals eligible for this study will be illiterate in French, so the study team will translate the consent orally into local languages when appropriate, as described in section 10.1.

5.4 Lifestyle Considerations

In addition to prohibited treatments and procedures listed in section 6.5, subjects must refrain from donating blood for at least 1 year after study drug administration.

Women must not breastfeed during the study because there could be unknown risks to the child secondary to the mother's receipt of the study drug.

5.5 Screen Failures

Screen failures are defined as subjects who consent to participate in the clinical trial but are not subsequently enrolled in the study. A minimal set of screen failure information is required to ensure transparent reporting of screen failure subjects, to meet the Consolidated Standards of

Reporting Trials (CONSORT) publishing requirements and to respond to queries from regulatory authorities. Minimal information includes demography, screen failure details, eligibility criteria, and any SAE.

Individuals who initially do not meet the criteria for participation in this trial (screen failure) because of an acute illness or a transient lab or other screening procedure findings may be rescreened at the investigator's discretion. Rescreened subjects should be assigned the same subject number as for the initial screening.

5.6 Strategies for Recruitment and Retention

Healthy adult volunteers will be selected based on the eligibility criteria described in sections 5.1 and 5.2. The total target sample size across both study sites is 18 subjects in the dose-escalation study (accrual ceiling 30) and 180 subjects in the efficacy study (accrual ceiling 250). We expect to enroll all subjects for both parts of the study within the first 4 months of the study. Efforts will be made to include women in proportions similar to that of the community from which they are recruited. Subject selection will not be limited based on sex, race, or ethnicity.

The study team will hold a community meeting in Kalifabougou and Torodo to explain and discuss the study and obtain community permission from the village elders, heads of families, and other community members in each village where the study will take place (section 10.1.1.1). Afterward, an announcement via local radio or another traditional channel of communication may be made to invite households to come to participating clinics to learn about the study.

5.6.1 Costs

There are no costs associated with participation in this trial.

5.6.2 Compensation

Subjects will be compensated for the time and inconvenience of participating in the study as follows:

- 5,000 Communauté Financière Africaine (CFA) Franc for day 0 (infusion).
- 3,000 CFA Franc for all other visits with venipuncture.
- 1,500 CFA Franc for all other visits without venipuncture.

The total compensation amount for dose-escalation study subjects will be 39,500 CFA Franc (valued at approximately USD $67) and for the efficacy study subjects will be 50,000 CFA Franc (valued at approximately USD $85). Payment will be provided in cash after the completion of each visit.

Subjects will be provided with transportation to and from study visits but will not receive additional reimbursement for travel.

6 STUDY INTERVENTION

6.1 Study Intervention Administration

6.1.1 Study Intervention Description

The study intervention involves a single administration of CIS43LS or placebo to each subject. All study agent administration will take place at the Kalifabougou study site.

6.1.2 Dosing and Administration

Study agent and dosing will be dependent on the progress of the study and study arm assignment (Table 1). The dose-escalation study dosing plan is described in section 6.1.2.1. Procedures for IV infusion are described in section 6.1.2.4.

Table 1. Study agent assignment and dosing by arm.

Arm	Subjects	Study Agent and Dose
**Dose-escalation study**		
1	6	5 mg/kg IV
2	6	20 mg/kg IV
3	6	40 mg/kg IV
**Efficacy study**		
1	90	40 mg/kg IV
2	90	Placebo (normal saline) IV

6.1.2.1 Dose Escalation

In the dose-escalation study, dose escalation will proceed in 3 study arms. Enrollment will begin with the arm receiving the lowest of 3 dose levels of CIS43LS. Subjects enrolled in this arm will be scheduled for study intervention, with at least 1 hour of observation between study agent administration in each subject, and follow-up procedures. Once all subjects at that dose level complete day 7 post-infusion, if no safety concerns have arisen, then enrollment will begin for the arm at the subsequent dose level. This process will be repeated to evaluate the second dose level prior to commencing with the highest dose level.

6.1.2.2 Dose Limiting Toxicity

Pausing and halting rules are provided in sections 8.4.5 and 8.4.6.

6.1.2.3 Dose Modifications

This study involves administering a single weight-based dose to each subject, so there will be no dose modifications.

6.1.2.4 Drug Administration

Prior to study agent administration on day 0, subjects will undergo vital signs measurement, a targeted physical examination (as needed based on signs, reported symptoms, or interim medical

history), and, for women, a urine pregnancy test (confirmed negative prior to administration). Subjects will have IV access placed in an arm vein in an aseptic manner. A different site may be used for collection of PK blood samples; however, the same site may be used after flushing the line if another site is not available.

The study agent will be administered with approximately 100 mL of normal saline IV over about 30-60 minutes, with a target of 30 minutes. Infusions lasting longer than 30 minutes are allowed. The rate of infusion may range from 10-20 mg/kg/hr at the lowest dose level to 80-160 mg/kg/hr at the highest dose level. The mL/hr infusion rate may vary based on the total volume needed to administer a full dose. Vital signs will be monitored directly after infusion, and hourly during monitoring. If the subject experiences side effects during the infusion, the rate of infusion may be slowed or stopped to alleviate the symptoms. At the end of product administration, the IV administration set must be flushed with about 30 mL (or appropriate volume) of normal saline. The IV will remain in for safety through the end of post-infusion observation.

In the dose-escalation study, the first subject in each dose arm to receive a unique dose level will be observed for at least 4 hours following completion of initial product administration. All other subjects will be observed for at least 2 hours following completion of product administration. In the efficacy study, all subjects will be observed for at least 60 minutes following administration. Prior to discharge from the clinic, subjects will be assessed for local and systemic reactogenicity and vital signs will be recorded. Any subject who is assessed as being unwell or has ongoing reactogenicity symptoms will be asked to remain in the clinic until evaluation and discharge by a study clinician. This includes the possibility of an overnight inpatient stay or referral to the district hospital to evaluate for safety and possible treatment.

6.2 PREPARATION/HANDLING/STORAGE/ACCOUNTABILITY

6.2.1 Acquisition and Accountability

Acquisition: CIS43LS will be shipped from the US to the study site where administration will take place, in compliance with all FDA, US Department of Transportation, and United Nations transport guidelines for shipping biohazardous materials. The placebo product will either be purchased in the US and shipped to Mali at ambient temperature or purchased in Mali.

Accountability: The study pharmacist will be responsible for maintaining an accurate record of the study arm codes, inventory, and an accountability record of study agent supplies. Electronic documentation as well as paper copies may be used.

The empty vials and the unused portion of a vial will be discarded in a biohazard containment bag and incinerated or autoclaved in accordance with the institutional or pharmacy policy. Any unopened vials that remain at the end of the study will be returned to the production facility or discarded at the discretion of the manufacturer in accordance with policies that apply to investigational agents. Partially used vials will not be administered to other subjects or used for *in vitro* experimental studies. These vials will be disposed of in accordance with institutional or pharmacy policy.

6.2.2 Formulation, Appearance, Packaging, and Labeling

VRC-MALMAB0100-00-AB (CIS43LS) is a sterile buffered solution that is filled into single-dose vials. The placebo product (used in the efficacy study) will be sterile isotonic (0.9%) normal saline. Both CIS43LS and placebo are clear liquids and are indistinguishable from one another. The products will be prepared by an unblinded pharmacist and placed in a standard IV infusion bag when preparing for administration.

Vials of CIS43LS will be individually labeled with the name of the material, volume, lot number, concentration, storage instructions, Investigational Use Statement ("Limited by Federal Law to Investigational Use"), and manufacturer information.

6.2.3 Product Storage and Stability

CIS43LS: CIS43LS vials should be stored frozen at −35°C to −15°C in a qualified, continuously-monitored, temperature-controlled freezer.[14] The site pharmacist must promptly report any storage temperature excursions outside of the normal allowance for the storage device to the manufacturer (VRC). The affected product must be quarantined in a separate area under protocol-specific temperature ranges until further notice from the manufacturer. If the excursion results in thawed material, DO NOT REFREEZE; store the thawed, vialed material at 2°C to 8°C.

Provide the following information regarding the excursion to the manufacturer: lot number, fill volume and number of vials affected; temperature range and length of excursion, including data log reports; handling of materials post excursion (e.g., transfer to alternate storage, including times); visual inspection data of the materials (e.g., did the materials appear to have remained frozen, were vials cracked, etc.). The manufacturer will notify the site pharmacist if continued clinical use of the product is acceptable.

Prior to preparation for IV administration, thaw and equilibrate vials for a minimum of 90 minutes at ambient temperature (15°C to 32°C). If thawed vials are removed from 2°C to 8°C, equilibrate at ambient temperature for a minimum of 30 minutes. Prior to preparation for administration in the IV bag, vials should be gently swirled for approximately 30 seconds while avoiding foaming. DO NOT SHAKE THE VIAL.[14]

After product preparation in an IV bag, the prepared CIS43LS may be stored at 2°C to 8°C for a maximum of 24 hours and/or at ambient temperature (15°C to 32°C) for a maximum of 4 hours total including the infusion time. Product may not be stored in direct sunlight.[14]

Placebo: Normal saline will be stored at room temperature in a controlled room per product standards.

6.2.4 Preparation

For each IV infusion order, the subject's weight, dose level, and study arm code will be included in the pharmacy order. To prepare an IV infusion, the pharmacist will do the following:

- For subjects assigned to CIS43LS:
    1) Calculate the total milligrams of CIS43LS needed.

2) Retrieve the minimum number of thawed vials required to prepare the full dose. After vials are thawed, prior to preparation for administration in the IV bag, vials should be gently swirled for 30 seconds while avoiding foaming. DO NOT SHAKE THE VIAL.
3) Withdraw the necessary amount of CIS43LS.
4) Add this volume to a 150 mL capacity, partial fill, 100 mL bag of normal saline using sterile compounding techniques to maintain sterility.

- For subjects assigned to placebo:
    1) Obtain a 100 mL bag of normal saline for administration.

An in-line filter infusion set must be used for IV product administrations and MUST comply with the following specifications: 1.2-micron polyethersulfone filter membrane, diethylhexylphthalate-free, latex-free (equivalent to Braun #473994 filter extension set). When the in-line filter is added to the tubing, the administration set must then be primed.

6.3 Measures to Minimize Bias: Randomization and Blinding

6.3.1 Randomization

In the efficacy study, subjects will be randomized to 1 of 2 treatment arms, including a CIS43LS arm and a placebo arm. Randomization lists will be generated by the study statistician, and the randomization code list will be maintained by a designated pharmacist at the study site where the study intervention will take place.

Randomization is further described in section 9.4.1.

6.3.2 Blinding

The efficacy study will be conducted with a double blind. The subjects, the clinical staff, and the study team will be blinded to study treatment allocation. The pharmacy team at the study site where administration is taking place will be unblinded, and they are responsible for maintaining security of study treatment assignments.

Data will remain blinded until the last subject completes the final study visit. Subjects will then be informed about their study treatment assignment.

Unscheduled unblinding, either intentional (e.g., in the case of a medical emergency in a subject) or unintentional, will be handled according to SOPs. Intentional and unintentional unscheduled unblinding will be documented in the appropriate source and/or research record and will include the reason for the unscheduled unblinding, the date it occurred, who approved the unblinding, who was unblinded, who was notified of the unblinding, and the plan for the subject.

The principal investigator will report all cases of intentional and unintentional unscheduled unblinding to the DSMB in writing within 1 business day after site awareness via email to the DSMB mailbox (niaiddsmbia@niaid.nih.gov) outlining the reason for the unblinding and the date it occurred. The report will also be submitted to the EC. If an SAE has resulted in unblinding, this information will be included in the SAE Report.

6.4 Study Intervention Compliance

Study intervention administration will be documented by study staff.

6.5 Concomitant Therapy

All concomitant prescription and nonprescription (including over-the-counter, herbal, or traditional) medications taken during study participation will be recorded. For this protocol, a prescription medication is defined as a medication that can be prescribed only by a properly authorized/licensed clinician.

Treatment with the following drugs and procedures will not be permitted unless discussed with and approved by the investigator:

- Live vaccines within 4 weeks of study agent administration.
- Killed vaccine within 2 weeks of study agent administration.
- Immunoglobulins and/or blood products for the duration of the study.
- Receipt of any investigational product or co-enrollment in other clinical studies of investigational products.
- Oral or IV corticosteroids at immunosuppressive doses (i.e., prednisone >10 mg/day) or immunosuppressive drugs for the duration of the study.
- Antimalarials and antibiotics with known antimalarial activity.

7 STUDY INTERVENTION DISCONTINUATION AND PARTICIPANT DISCONTINUATION/WITHDRAWAL

7.1 Discontinuation of Study Intervention

Study intervention may be discontinued for a protocol-defined group or arm (i.e., pausing), or it may be discontinued for all subjects and enrollment suspended (i.e., halting). Pausing and halting are described in section sections 8.4.5 and 8.4.6. Subjects who have already received the study agent at the time of a pause or halt will continue planned follow-up under the protocol.

7.2 Participant Discontinuation/Withdrawal from the Study

Plans for managing the involuntary withdrawal of a subject are provided in section 8.4.3. The reason for subject discontinuation or withdrawal from the study will be recorded on the case report form (CRF).

Subjects who withdraw after receiving study agent but prior to study completion will be encouraged to attend an early termination visit, where they will complete as many of the procedures and evaluations indicated in the schedule of activities (section 1.3) as possible.

7.3 Lost to Follow-up

A subject will be considered lost to follow-up if he or she fails to return for 3 consecutive scheduled visits and is unable to be contacted by the study site staff.

The following actions must be taken if a subject fails to return to the clinic for a required study visit:

- The site will attempt to contact the subject and reschedule the missed visit within 7 days, counsel the subject on the importance of maintaining the assigned visit schedule, and ascertain if the subject wishes to and/or should continue in the study.
- Before a subject is deemed lost to follow-up, the investigator or designee will make every effort to regain contact with the subject (where possible, 3 telephone calls). These contact attempts should be documented in the subject's medical record or study file.
- Should the subject continue to be unreachable, he or she will be considered to have withdrawn from the study with a primary reason of lost to follow-up.

8 STUDY ASSESSMENTS AND PROCEDURES

8.1 SCREENING PROCEDURES

Screening will be performed at the Kalifabougou study site. The study staff will explain the study to the prospective subject, complete the study comprehension examination and obtain consent (section 10.1), and assess eligibility. Consent will be obtained before any study-related procedures are performed.

The following screening procedures and evaluations must be performed within -56 to -7 days of study intervention. Screening may take place over multiple visits if necessary.

- Confirmation of identity, age, and residency.
- Complete review of medical history and medication use.
- Complete physical examination, including height and weight.
- Vital signs (temperature, blood pressure, and pulse).
- 12-lead ECG.
- Urine collection for urinalysis (urine dipstick or formal urinalysis; acceptable laboratory parameters defined in Appendix A).
- For women, serum β-hCG pregnancy test.
- Blood collection via venipuncture for screening evaluations (acceptable laboratory parameters defined in Appendix A):
    - HIV tests: 2 rapid diagnostic tests (RDTs), plus ELISA if the RDTs are discordant. A subject will be referred for medical care for 2 positive RDTs or a positive ELISA. If the ELISA is negative, no further work-up will be done. Pre- and post-test HIV counseling will be provided.
    - HBV and HCV tests. If either test is positive, the subject will be referred for care regardless of the ALT result.
    - Hemoglobin typing.
    - Complete blood count (CBC) with differential.
    - ALT.
    - Cr.
- Pregnancy prevention confirmation and counseling.
- Pre- and post-test HIV counseling.

A prospective subject who has any clinically significant abnormal finding and/or is diagnosed with a medical condition at screening or during the conduct of the study will be notified and referred for medical care. Per national requirements for reporting communicable diseases, confirmed positive test results for HIV, HBV, and HCV will be reported to the local health department according to applicable laws and appropriate medical referrals initiated. The cost of initial and long-term treatment and care of medical conditions diagnosed during the screening process will not be reimbursed by the study but referrals to relevant specialist will be provided.

If screening is completed outside the specified window, all screening procedures and evaluations must be repeated. If an individual screens and is enrolled into the dose-escalation study but for any reason does not receive study agent, they may later consent to be screened and enrolled in the efficacy study.

Enrollment: If the individual is eligible and agrees to participate, he or she will be scheduled to come for an enrollment visit, as described in section 1.3. Enrollment is defined as the time of artemether-lumefantrine administration; at this time, the subject will be assigned a unique study identification number in the clinical database. A clinician will discuss the target dates and timing of the study agent administration and sample collections before completing an enrollment to help ensure that the subject can comply with the projected schedule. The identification number will link subject samples and data collected throughout the study.

8.2 Efficacy Assessments

8.2.1 Clinical Evaluations

The following clinical evaluations will be performed as efficacy assessments.

Medical History and Medication Review: A complete review of all medical history and medications will be conducted at screening. Subsequent visits will include a targeted review of changes in medical history or medications since the last study visit.

Physical Examination: A complete physical examination (including height and weight) will be done at screening. A targeted physical examination based on signs, reported symptoms, and medical history will be conducted at subsequent study visits. Weight will also be recorded on day 0 prior to study agent administration.

Artemether-Lumefantrine: At enrollment, all subjects will be orally administered standard artemether-lumefantrine treatment (4 tablets twice daily for 3 days) to clear any possible Pf blood-stage infection prior to study agent administration.

Randomization Procedures: Randomization for the efficacy study is described in sections 6.3.1 and 9.4.1.

Study Agent Administration and Monitoring: Study agent infusion and monitoring will be performed according to the assigned arm, as described in section 6.1.

Illness Visit: A subject will be instructed to come in for an unscheduled visit if he or she has symptoms of malaria or other symptoms. The subject will be evaluated by the study team and referred for standard care according to local guidelines. At an illness visit, the subject will undergo review of medical history and concomitant medications, a focused physical exam for symptoms of malaria or other diseases, vital sign measurement, and a fingerprick blood

collection for blood smear for malaria diagnosis as well as a dried blood spot for Pf PCR for research purposes.

Malaria Diagnosis and Management: If a subject has a malarial infection, we will share these results with the subject and provide standard treatment in accordance with the recommendations of the Mali National Malaria Control Program. According to the national guidelines, asymptomatic malarial infections in adults are not treated. Malaria treatment is given only when symptoms are present along with positive blood smear results. PCR is not commonly used for routine malaria diagnosis.

8.2.2 Biospecimen Evaluations

Blood will be collected under this protocol by the following methods:

- Venipuncture will be performed with single-use needles. Venous blood samples will be used as follows:
    - Safety evaluations described in section 8.3.
    - Shipment to the research laboratory in Bamako and NIAID for evaluation (including assays described in section 8.2.3) and storage.
- Fingerprick will be performed using single-use disposable lancets. Fingerprick blood samples will be used as follows:
    - Blood smear and dried blood spot for Pf PCR.

The amount of blood drawn for research purposes will be within the limits allowed for adult research subjects by the NIH Clinical Center: no more than 10.5 mL/kg or 550 mL (whichever is smaller) over an 8-week period.

The collection schedule, volumes, and test tubes are presented in section 1.3.

8.2.3 Correlative Studies for Research/Pharmacokinetic Studies

The following evaluations will be performed according to the schedule presented in section 1.3.

Blood smear: Thick blood smears will be prepared and analyzed by the standard WHO method (section 2.2.3.3) to identify Pf infection for the primary endpoint. This evaluation will be performed centrally in the laboratory in Bamako.

Pf PCR: PCR will be performed to identify Pf infection as a secondary endpoint. This evaluation will be performed at the NIAID.

Pharmacokinetic studies: Blood CIS43LS concentrations will be measured by Meso Scale Discovery LLC–based automation platform. The concentration at the visit prior to the first Pf infection will be used to assess CIS43LS-mediated protection. This evaluation will be performed at the NIAID.

ADA detection: Assays for detection of ADA will be performed at specified timepoints (section 1.3) following product administration and compared to baseline status. This evaluation will be performed at the NIAID.

Parasite genotyping: For subjects who become infected during the study, blood samples collected around the time of the first infection will be used to perform a genotypic sieve analysis

to analyze sequences of breakthrough parasites (section 9.4.11). This evaluation will be performed at the NIAID.

8.2.4 Samples for Genetic/Genomic Analysis

8.2.4.1 Description of the scope of genetic/genomic analysis

Genetic testing performed in this protocol will be limited to analyzing the genetic material of infection-inducing parasites in blood samples. No human genetic analyses will be performed.

8.2.4.2 Description of how privacy and confidentiality of medical information/biological specimens will be maximized

Privacy and confidentiality of medical information and biological samples is described in section 10.3.

8.2.4.3 Management of Results

As no human genetic analyses will be performed in this protocol, no genetic results will be returned to subjects.

8.2.4.4 Genetic counseling

Not applicable.

8.3 Safety and Other Assessments

The following study procedures and evaluations will be done according to the schedule in section 1.3 to monitor safety and support the understanding of the study intervention's safety. The assessment and collection of safety events such as AEs are described in section 8.4.2.

Physical Examination: As described in section 8.2.1, physical examination will also be performed for assessment of safety.

Vital Signs: Vital signs (temperature, blood pressure, and heart rate) will be collected at visits, including before and after study agent infusion, as described in section 8.2.1.

Safety Blood Laboratory Evaluations: The following safety laboratory evaluations will be performed at a frequency presented in section 1.3:

- CBC with differential.
- ALT, Cr.

Pregnancy Testing: Women will have a serum pregnancy test at screening and urine tests at enrollment, day 0 (confirmed negative prior to administration of the study agent), and monthly through the final study visit.

Pregnancy Prevention Counseling: Female subjects will be counseled on the importance of not becoming pregnant during study participation and on acceptable methods of contraception.

8.4 SAFETY DEFINITIONS, MANAGEMENT, AND SPONSOR REPORTING

8.4.1 Definitions

Adverse Event: An AE is any untoward or unfavorable medical occurrence in a human subject, including any abnormal sign (e.g., abnormal physical exam or laboratory finding), symptom, or disease, temporally associated with the subject's participation in the research, whether or not considered related to the research.

Adverse Reaction: An AR means any AE caused (see "Causality" below) by a study agent. ARs are a subset of all suspected adverse reactions (SARs; defined below) where there is reason to conclude that the study agent caused the event.

Suspected Adverse Reaction: SAR means any AE for which there is a reasonable possibility that the study agent caused the AE.

Per US FDA guidance:

For the purposes of IND safety reporting, "reasonable possibility" means there is evidence to suggest a causal (see "Causality" below) relationship between the study agent and the AE. A SAR implies a lesser degree of certainty about causality than an AR, which means any AE caused by a study agent.

SARs are the subset of all AEs for which there is a reasonable possibility that the study agent caused (see "Causality" below) the event. Inherent in this definition, and in the requirement to report SARs, is the need for the sponsor to evaluate the available evidence and make a judgment about the likelihood that the study agent actually caused the AE.

The sponsor is responsible for making the causality judgment.

Serious Adverse Event: An SAE:

- is an AE that results in death.
- is an AE that is life-threatening event (places the subject at immediate risk of death from the event as it occurred).
- is an AE that requires inpatient hospitalization or prolongs an existing hospitalization.

  NOTE:

  - Hospitalization is considered required if outpatient treatment would generally be considered inappropriate.
  - Same-day surgical procedures that are required to address an AE are considered hospitalizations, even if they do not involve an overnight admission.
  - Hospitalization due to a condition that has not worsened and that pre-dates study participation (e.g., elective correction of an unchanged baseline skin lesion), or due to social circumstance (e.g., prolonged stay to arrange aftercare), or that is planned/required "per protocol" AND that proceeds without prolongation or complication, is NOT considered an SAE by this criterion.
- is, or results in, a persistent or significant incapacity or substantial disruption of the ability to conduct normal life functions.
- is a congenital anomaly/birth defect/miscarriage/stillbirth.

NOTE: This definition is more inclusive than some commonly published definitions. It includes an affected conceptus/neonate whose:

- biological mother was exposed to a study agent at any point from conception through the end of the pregnancy, AND/OR, if breastfeeding, the 30-day neonatal period; or
- biological father was exposed to a study agent at any point during the 90 days prior to conception.

This is separate from, and in addition to, general reporting of pregnancy in a study participant or female partner of a male participant (see section 8.4.2.3.4 below).

- is a medically important event.

NOTE: Medical and scientific judgment should be exercised. Events that significantly jeopardize the subject and/or require intervention to prevent one of the SAE outcomes listed above are generally considered medically important, and are thus SAEs.

Unexpected Adverse Event: An AE is unexpected if it is not listed in the investigator's brochure or package insert (for marketed products) at the frequency, AND specificity, AND severity that has been observed.

NOTE:

- Such events should also be evaluated for possible reporting as unanticipated problems (UPs) (see section 8.4.2.3.3 below).
- Unexpected, as used in this definition, also refers to AEs or SARs that are mentioned in the investigator's brochure as occurring with a class of drugs/biologics, or as anticipated from the pharmacological properties of the study agent but are not specifically mentioned as occurring with the particular study agent under investigation.

Serious and Unexpected Suspected Adverse Reaction (SUSAR): A SUSAR is an SAR (defined above) that is both serious and unexpected.

Unanticipated Problem: A UP is any incident, experience, or outcome that meets all the following criteria:

1. Unexpected (in terms of nature, severity, or frequency) given
   a. the research procedures that are described in the protocol-related documents, such as the EC-approved research protocol and informed consent document; and
   b. the characteristics of the subject population being studied; and
2. Related or possibly related to participation in the research (possibly related means there is a reasonable possibility that the incident, experience, or outcome may have been caused by the procedures involved in the research), and
3. Suggests the research places subjects or others (which may include research staff, family members or other individuals not directly participating in the research) at a greater risk of harm (including physical, psychological, economic, or social harm) related to the research than was previously known or expected.

NOTE:

- Per the sponsor, an SAE always meets this "greater risk" criterion.

- An incident, experience, or outcome that meets the definition of a UP generally will warrant consideration of changes to the protocol or informed consent form, or to study procedures (e.g., the manual of procedures for the study), in order to protect the safety, welfare, or rights of participants or others. Some UPs may warrant a corrective and preventive action plan at the discretion of the sponsor or other oversight entities.

Unanticipated Problem that is not an Adverse Event (UPnonAE): A UPnonAE belongs to a subset of UPs that:

- meets the definition of a UP, AND
- does NOT fit the definition of an AE or an SAE.

  NOTE: Examples of UPnonAEs include, but are <u>not limited to</u>:

  - a breach of confidentiality
  - prolonged shedding of a vaccine virus beyond the anticipated timeline
  - unexpectedly large number of pregnancies on a study
  - subject departure from an isolation unit prior to meeting all discharge criteria
  - accidental destruction of study records
  - unaccounted-for study agent
  - overdosage, underdosage, or other significant error in administration or use of study agent or intervention, even if there is no AE/SAE
  - development of an actual or possible concern for study agent purity, sterility, potency, dosage, etc.

    NOTE: A decision to temporarily quarantine, or to permanently not use all or part of study agent supply due to an unexpected finding or event (e.g., particulate, cloudiness, temperature excursion), even if there is no known or proven issue (i.e., out of an "abundance of caution"), is considered a UPnonAE.

Protocol Deviation: Any change, divergence, or departure from the EC-approved research protocol.

1. **Major deviations:** Deviations from the EC-approved protocol that have, or may have the potential to, negatively impact the rights, welfare, or safety of the subject, or to substantially negatively impact the scientific integrity or validity of the study.
2. **Minor deviations:** Deviations that do not have the potential to negatively impact the rights, safety, or welfare of subjects or others, or the scientific integrity or validity of the study.

Noncompliance: Failure of investigator(s) to follow the applicable laws, regulations, or institutional policies governing the protection of human subjects in research, or the requirements or determinations of the EC, whether intentional or not.

1. **Serious noncompliance:** Noncompliance, whether intentional or not, that results in harm or otherwise materially compromises the rights, welfare and/or safety of the subject. Noncompliance that materially affects the scientific integrity or validity of the research

may be considered serious non-compliance, even if it does not result in direct harm to research subjects.

2. **Continuing noncompliance:** A pattern of recurring noncompliance that either has resulted, or, if continued, may result in harm to subjects or otherwise materially compromise the rights, welfare and/or safety of subjects, affect the scientific integrity of the study or validity of the results. The pattern may comprise repetition of the same non-compliant action(s), or different noncompliant events. Such non-compliance may be unintentional (e.g., due to lack of understanding, knowledge, or commitment), or intentional (e.g., due to deliberate choice to ignore or compromise the requirements of any applicable regulation, organizational policy, or determination of the EC).

8.4.2 Documenting, Assessing, Recording, and Reporting Events

ALL AEs, including those that may appear to have a non-study cause (see "Causality" below), will be documented (e.g., on the clinical chart/progress notes/clinical laboratory record), recorded (e.g., in the study-specified CRF/research database), and reported (e.g., cumulatively from the research database, or according to protocol-specified expedited reporting mechanism) to the sponsor from the time informed consent is obtained through the timeframe specified below. At each contact with the subject, information regarding AEs will be elicited by open-ended questioning and examinations.

AEs and SAEs will generally be recorded, assessed, and reported according to the timeframes outlined in Table 2.

Table 2. Standard event recording, assessment, and reporting timeframes.

Event type	Record, assess, and report through
Related SAEs	End of subject participation in study, or if study personnel become aware thereafter
Unrelated SAEs	End of subject participation in study
Related non-serious AEs of grade 1 to 3	End of subject participation in study
All other related non-serious AEs	End of subject participation in study
Unrelated non-serious AEs	End of subject participation in study

8.4.2.1 Investigator Assessment of Adverse Events

The investigator will assess all AEs with respect to **seriousness** (according to SAE definition above), **severity** (intensity or grade, see below), and **causality** (relationship to study agent and relationship to participation in the research, see below).

8.4.2.1.1 Severity Grading

The investigator will grade the severity of each AE, including laboratory and testing abnormalities and results, according to the "Guidance for Industry: Toxicity Grading Scale for Healthy Adult and Adolescent Volunteers Enrolled in Preventive Vaccine Clinical Trials" which can be found at: https://www.fda.gov/regulatory-information/search-fda-guidance-documents/toxicity-grading-scale-healthy-adult-and-adolescent-volunteers-enrolled-preventive-vaccine-clinical

Events that are NOT gradable using the above specified table will be graded as follows:

- Mild = grade 1
- Moderate = grade 2
- Severe = grade 3
- Potentially life threatening = grade 4
- Death = grade 5

NOTE: A subject death should always be reported as grade 5.

8.4.2.1.1.1 Laboratory Value Assessment and Clinical Significance Criteria

Except as specified below, ALL abnormal lab values of grade 1 or above are REPORTABLE.

Grade 1 and 2 abnormal laboratory values are considered CLINICALLY SIGNIFICANT, and are to be recorded in the research database, and reported, if they meet ONE or more of the following criteria:

- result in a study agent dosage adjustment, interruption, or discontinuation
- are accompanied by clinically abnormal signs or symptoms that are likely related to the laboratory abnormality (e.g., clinical jaundice)
- indicate a possible organ toxicity (e.g., elevated serum creatinine)
- result in additional/repeat testing or medical intervention (procedures/treatments) (e.g., ECG to evaluate arrhythmia potential with a high serum potassium; one or more ECGs to assess an elevated troponin level; potassium supplementation for hypokalemia)
- indicates possible over-dosage
- are considered clinically significant by the investigator or SMM

8.4.2.1.2 Causality

Causality (likelihood that the event is caused by the study agents) will be assessed by the principal investigator considering the factors listed under the following categories:

Definitely Related

- reasonable temporal relationship
- follows a known response pattern
- clear evidence to suggest a causal relationship
- there is no alternative etiology

Probably Related

- reasonable temporal relationship
- follows a suspected response pattern (based on similar agents)
- no evidence of a more likely alternative etiology

Possibly Related

- reasonable temporal relationship
- little evidence for a more likely alternative etiology

Unlikely Related

- does not have a reasonable temporal relationship

  AND/OR

- there is good evidence for a more likely alternative etiology

Not Related

- does not have a temporal relationship

  AND/OR

- definitely due to an alternative etiology

Note: Other factors (e.g., dechallenge, rechallenge, if applicable) should also be considered for each causality category when appropriate. Causality assessment is based on available information at the time of the assessment of the AE. The investigator may revise the causality assessment as additional information becomes available.

Causality assessment will be reviewed by the sponsor. The sponsor may make a separate and final determination on the "reasonable possibility" that the event was "related" (comprising definitely, probably, and possibly related) or "unrelated" (comprising unlikely and not related) to the study agent, in keeping with applicable (US FDA) guidance on sponsor IND safety reporting.

8.4.2.2 Recording of Events

AEs will be promptly recorded in the research database, regardless of possible relationship to study interventions. If a diagnosis is clinically evident (or subsequently determined), the diagnosis rather than the individual signs and symptoms or laboratory abnormalities will be recorded as the AE. The investigator will review events regularly to ensure they have been captured correctly and to perform assessment of events individually and cumulatively to assess possible safety trends.

8.4.2.3 Investigator Reporting Responsibilities

The principal investigators and/or equally qualified designees will check daily for events that may require expedited reporting.

The principal investigators and/or equally qualified designees will also monitor all accumulating data no less than weekly, or according to superseding NIH or NIAID policy, whichever is more frequent.

Data will be reviewed by the principal investigators/designees on a regular basis for accuracy and completeness.

Data will be submitted to the sponsor in keeping with all applicable agreements and when requested, such as for periodic safety assessments, review of IND annual reports, review of IND safety reports, and preparation of final study reports.

The principal investigators and/or other study designee will ensure prompt reporting to safety oversight bodies (e.g., Clinical Safety Office [CSO], DSMB), regulatory entities, and stakeholders as specified below, and according to any additional requirements or agreements.

8.4.2.3.1 Adverse Events

Unless otherwise specified above, AE data will be entered into the research database no less than every other week and will include all data through 1 week prior to database entry.

8.4.2.3.2 Serious Adverse Events (Expedited Reporting)

Unless otherwise specified above, all SAEs (regardless of relationship and whether or not they are also UPs) must be reported to the CSO as specified by the CSO (e.g., Research Electronic Data Capture [REDCap] system; use the Safety Expedited Report Form [SERF]/email if REDCap is not available). If the preferred/indicated mechanism for reporting is not available, the CSO/SMM should be contacted by telephone, fax, or other reasonable mechanism to avoid delays in reporting.

CSO CONTACT INFORMATION:

Clinical Safety Office

5705 Industry Lane
Frederick, MD 21704
Phone: [redacted]
Fax: [redacted]
Email: [redacted]

Unless otherwise specified above, deaths and immediately life-threatening SAEs must be reported to the CSO promptly, and no later than the **first business day** following the day of study personnel awareness.

All other SAEs must be reported to the CSO no later than the **third business day** following the day of study personnel awareness.

If an individual subject experiences multiple SAEs in a closely timed/overlapping "cause-and-effect" (cascade) sequence, the principal investigators, after careful evaluation, will report ONLY primary/precipitating event(s) individually. SAEs that are determined to be definitely secondary to other SAEs will be detailed in the narrative portion of the report of the relevant primary/precipitating SAE. A clinical rationale and findings to support such reporting should be part of the narrative.

For each SAE report, the research database entry MUST match the corresponding entries on the SAE report (e.g., start and stop dates, event type, relationship, and grade), and **must be updated if necessary** (e.g., if the SAE report was generated after the corresponding AE was entered in the research database).

Unless otherwise specified above, SAEs that have not resolved by the end of the per-protocol follow-up period for the subject are to be followed until final outcome is known (to the degree permitted by the EC-approved informed consent form). If it is not possible to obtain a final outcome for an SAE (e.g., the subject is lost to follow-up), and to update the CSO, the last known status and the reason a final outcome could not be obtained will be recorded by the investigator on an SAE report update and the CRF.

8.4.2.3.3 Unanticipated Problems

Unless otherwise specified above, UPs (as defined in this protocol, or as defined by the EC of record, whichever definition is more conservative) that are also AEs or SAEs, must be reported to the CSO (by REDCap, or by email and SERF if REDCap is not available) no later than when they are due to be reported to the EC.

UPnonAEs are NOT reported to the CSO but must be reported to the Clinical Trials Management (CTM) group according to their requirements and preferred methods. If the UPnonAE raises a significant potential subject safety concern, the SMM should be consulted by email or phone no later than when reports are made to the CTM.

8.4.2.3.4 Pregnancy

Unless otherwise specified above, all pregnancies will be reported (by REDCap, or by email and SERF if REDCap is not available) to the CSO no later than the first business day following the day of study personnel awareness.

Pregnancy outcome data (e.g., delivery outcome, spontaneous or elective termination of the pregnancy) will be reported to the CSO no later than the third business day following the day of study personnel awareness (by REDCap, or by email and SERF if REDCap is not available).

Pregnancy itself is not an AE. Events that meet AE or SAE criteria in relation to pregnancy, delivery, or the conceptus/neonate (see section 8.4.1) are reportable (by REDCap, or by email and SERF if REDCap is not available).

In the event of pregnancy in a study subject exposed to study agent, the following actions will be taken, with the goal of ensuring maternal and fetal well-being, in consultation with the SMM, independent safety monitor (ISM), and DSMB:

- Discontinue the following study-specific procedures:
  - Research sample collections.
- Continue to follow for safety for the duration of the pregnancy and for a period of up to 6 months (per investigator discretion) following delivery for assessment of the neonate.
- Request to unblind the subject, if applicable, AND if doing so would offer a benefit to the subject.
- Report, no later than the first business day after study personnel awareness, to the ISM and DSMB.
- Advise subject to notify the obstetrician of study participation and study agent exposure, providing contact information for the obstetrician to contact the study principal investigator, should this be required, and with the subject's consent.

8.4.2.4 Sponsor's Reporting Responsibilities

Events reported to the sponsor will be promptly evaluated and will be reported as required according to FDA IND safety reporting guidance and regulations. IND safety reports will be sent to other investigators conducting research under the same IND and will be shared with other stakeholders according to applicable agreements.

The sponsor will also submit an IND annual report of the progress of the investigation to the FDA as defined in 21 CFR 312.33.

All UPs will be evaluated by the sponsor, and a summary of the event, and any necessary (corrective/preventative) actions, will be distributed to investigators conducting research under the same IND as may be relevant and appropriate.

8.4.3 Withdrawal Criteria for an Individual Subject

An individual subject will be withdrawn from the study for any of the following:

- An individual subject's decision. (The investigator should attempt to determine the reason for the subject's decision.)
- Non-compliance with study procedures to the extent that it is potentially harmful to the subject or to the integrity of the study data.
- A change in the subject's condition as follows:
  - Loss of the ability to provide informed consent.
  - Withdraws permission to have blood samples or data stored for future research.
- The investigator determines that continued participation in the study would not be in the best interest of the subject.

8.4.3.1 Re-enrollment and Unplanned Procedure Repetition

Unless otherwise specified within this protocol, each person who is a subject in this study may be enrolled and may pass through each step and process outlined in the protocol, only **ONCE** (i.e., subjects may not "go back" and repeat a protocol step already completed). On a case-by-case basis, a request for re-enrollment, or for repetition of a protocol step or procedure already completed, may be submitted to, reviewed by, and approved by the SMM in writing. The SMM may also recommend or require consultation of the EC and/or DSMB and ISM.

8.4.3.2 Replacement of Withdrawn Subjects or Subjects Who Discontinue Study Agent

In the dose-escalation study, subjects withdrawn prior to the day 7 safety evaluation will be replaced. In the efficacy study, subjects withdrawn prior to study agent administration will be replaced.

All subjects exposed to study agents MUST be included in the safety dataset.

8.4.4 Additional Safety Oversight

8.4.4.1 Safety Review and Communications Plan

A safety review and communication plan (SRCP) is required for this protocol. The SRCP is an internal communications document between the principal investigators and the CSO, as sponsor representative, which delineates key safety oversight responsibilities of the principal investigators, the CSO, and other stakeholders. The SRCP includes a plan for conducting periodic safety surveillance assessments by the CSO.

8.4.4.2 Sponsor Medical Monitor

A SMM, representing the sponsor, has been appointed for oversight of safety in this clinical study. The SMM will be responsible for performing safety assessments as outlined in the SRCP.

8.4.4.3 Oversight Committees

8.4.4.3.1 Independent Safety Monitor in Mali

The ISM is an expert who does not have direct involvement in the conduct of the study and has no significant conflicts of interest as defined by NIAID policy. An ISM in Mali will review the study prior to initiation and will be available to advise the investigators on study-related medical issues and act as a representative for the welfare of the subjects. The ISM will conduct independent safety monitoring and recommend appropriate action regarding AEs and other safety issues. The ISM is an expert in the field of oversight of clinical trials conducted in Mali and internal medicine, specifically in the population under study in Mali.

All deaths, SAEs, UPs, pregnancies, and FDA IND safety reports will be reported by the principal investigators to the ISM prior to or at the same time they are submitted to the EC or CSO unless otherwise specified herein. The ISM will be notified immediately if any pausing rule is met and the ISM will provide recommendation for continuation, modification, or termination

of the study. The principal investigators will also notify the ISM if intentional or unintentional unblinding occurs. The ISM will be a voting member of the DSMB.

8.4.4.3.2 Data and Safety Monitoring Board

The NIAID intramural DSMB includes independent experts that do not have direct involvement in the conduct of the study and have no significant conflicts of interest as defined by NIAID policy. The DSMB will review the study protocol, consent documents, and investigator brochure prior to initiation and twice a year thereafter, or as may be determined by the DSMB. Additionally, the DSMB will conduct 1 interim analysis when safety data are available from the dose-escalation study subjects (section 9.4.8).

The DSMB may convene additional reviews as necessary. The DSMB will review the study data as needed to evaluate the safety, efficacy, study progress, and conduct of the study.

All deaths, SAEs, UPs, pregnancies, and IND safety reports will be reported to the DSMB at the same time they are submitted to the EC and CSO unless otherwise specified herein.

All cases of intentional or unintentional unblinding will be reported to the DSMB not later than 1 business day from the time of study personnel awareness.

The principal investigators will notify the DSMB at the time pausing or halting criteria are met and obtain a recommendation concerning continuation, modification, or termination of the study.

8.4.5 Pausing Rules

"Pausing" is discontinuation of study intervention/treatment/dosing (agent/placebo/procedure, etc.) in a protocol-defined group or "arm," until a decision is made to either resume or permanently discontinue such activity. Subjects continue to be followed for safety during a pause.

The pausing criteria for a group or arm (e.g., a specific dosing group) in this study include any one or more of the following:

- A subject experiences an SAE that is unexpected (per the investigator's brochure or product label) and possibly, probably, or definitely related to a study agent;
- A subject experiences 2 grade 3 or greater AEs that are unexpected (per the investigator's brochure or product label) and possibly, probably, or definitely related to a study agent.

The principal investigators or the CSO may also pause dosing/study interventions for one or more subjects for any safety issue. The study safety oversight bodies (e.g., DSMB, ISM) may recommend a pause to the CSO.

8.4.5.1 Reporting a Pause

If a pausing criterion is met, a description of the AE(s) or safety issue must be reported by the principal investigators within 1 business day to the CSO and the EC according to their requirements. The principal investigators will also notify the DSMB and ISM. In addition, the CSO or designee will notify all other site investigators by email or through the specified pathway.

8.4.5.2 Resumption Following a Pause

The CSO, in collaboration with the principal investigators and DSMB and ISM, will determine if study activities, including agent administration and/or other study interventions may be resumed, and any additional modifications or requirements that may apply, for the impacted subject(s), or whether the events that triggered the pause require expansion to a study halt (see below).

The CSO or sponsor designee will notify the principal investigators of the decision. The principal investigators will notify the EC of the decision according to the EC’s process.

8.4.5.3 Discontinuation of Study Agent

A subject who does not resume study agent/intervention/treatment will continue to be followed for protocol-specified safety assessments or as clinically indicated, whichever is more conservative.

8.4.6 Halting Rules for the Protocol

“Halting” is discontinuation of study intervention/treatment/dosing (agent/placebo/procedure, etc.) for all subjects in a study and suspension of enrollment until a decision is made to either resume or permanently discontinue such activity. Subjects continue to be followed for safety during a halt.

The halting rules are:

- Two or more subjects experience the same or similar grade 3 or greater AEs that are unexpected and possibly, probably, or definitely related to a study agent;

  OR

- Any safety issue that the principal investigators or the CSO determines should halt the study. The study safety oversight bodies (e.g., DSMB) may recommend a halt to the CSO.

In addition, the FDA, Malian Ministry of Health, FMPOS EC, or any regulatory body having oversight authority may halt the study at any time. The DSMB or ISM may recommend a study halt.

8.4.6.1 Reporting a Study Halt

If a halting criterion is met, a description of the AE(s) or safety issue must be reported by the principal investigators, within 1 business day to the CSO and the EC according to their requirements. The principal investigators will also notify the DSMB and ISM. In addition, the CSO or designee will notify all other site investigators by email or through the specified pathway.

8.4.6.2 Resumption of a Halted Study

The CSO, in collaboration with the principal investigators and DSMB and ISM will determine if study activities, including enrollment, study agent administration, and/or other study interventions, may be resumed and any additional modifications or requirements that may apply.

The CSO or sponsor designee will notify the principal investigators of the decision. The principal investigators will notify the EC of the decision according to the EC's process.

8.4.6.3 Discontinuation of Study Agent

Subjects who do not resume study agent/study intervention will continue to be followed for protocol-specified safety assessments or as clinically indicated, whichever is more conservative.

8.5 UNANTICIPATED PROBLEMS

8.5.1 Definition of an Unanticipated Problem

The definition of a UP is provided in section 8.4.1.

8.5.2 Unanticipated Problem Reporting

The investigator will report UPs to the FMPOS EC according to NIH Human Research Protection Program (HRPP) Policy 801, as described in section 8.6.1.

8.6 ADDITIONAL REPORTING REQUIREMENTS

8.6.1 Reporting to the FMPOS EC

Non-compliance and other reportable events will be reported to the FMPOS EC according to NIH HRPP Policy 801, which requires reporting as described below.

The following will be reported within 7 calendar days of any investigator or individual associated with the protocol first becoming aware:

- Actual or suspected noncompliance.
- Actual or suspected major deviation.
- Actual or suspected UPs.
- New information that might affect the willingness of a subject to enroll or remain in the study.
- Suspension or termination of research activities, including holds on new enrollment, placed upon the research by the sponsor, NIH or NIAID leadership, or any regulatory agency.

Any death of a research subject that is possibly, probably, or definitely related to the research must be reported within 24 hours of the investigator becoming aware of the death.

Additionally, investigators must provide the following information to the EC in summary format at the time of continuing review, or when otherwise specifically requested by the EC or the Office of Human Subjects Research Protections (OHSRP) Office of Compliance and Training:

- Major and minor protocol deviations.
- Noncompliance reported to the EC that is not related to a protocol deviation.
- AEs and SAEs that do not meet the definition of an UP.
- UPs reported to the EC.

8.6.2 Reporting to the NIAID Clinical Director

The principal investigator will report UPs, major protocol deviations, and deaths to the NIAID clinical director according to institutional timelines.

9 STATISTICAL CONSIDERATIONS

9.1 Statistical Hypothesis

The hypotheses are that CIS43LS will be safe and will confer protection against Pf infection. In the dose-escalation study, the primary objective is to evaluate the safety and tolerability. In the efficacy study, the primary objective is to evaluate the safety and efficacy of CIS43LS compared to placebo.

Primary Endpoints:

- Dose escalation: Incidence and severity of local and systemic AEs occurring within 7 days after the administration of CIS43LS
- Efficacy: Pf blood stage infection as detected by microscopic examination of thick blood smear for 24 weeks after administration of CIS43LS or placebo

Secondary Endpoints:

- Dose escalation: Measurement of CIS43LS in sera of recipients
- Efficacy: Pf blood stage infection as detected by PCR for 24 weeks after administration of CIS43LS or placebo
- Efficacy: Measurement of CIS43LS in sera of recipients

9.2 Sample Size Determination

9.2.1 Sample Size Considerations for the Dose-Escalation Study

The ability of the study to identify safety events can be expressed in terms of the probability of observing 1 or more event of interest (e.g., AEs) within each arm. With the sample size n=6 in each arm, there is over a 90% chance to observe at least 1 AE if the true rate is at least 0.319 and over a 90% chance to observe no AE if the true rate is no more than 0.017. With n=18 over 3 dose arms, there is over a 90% chance to observe at least 1 AE if the true rate is no less than 0.121 and over a 90% chance of observing no AE if the true rate is no more than 0.005. Probabilities of observing 0 or more than 1 AE within a group are presented in Table 3 for a range of possible true event rates.

Table 3. Probability (Pr) of events for different safety scenarios within an arm (n=6 or 18).

**True event rate**	**n=6**		**n=18**	
	**Pr(0)**	**Pr(>1)**	**Pr(0)**	**Pr(>1)**
0.005	0.970	0.000	0.914	0.004

True event rate	n=6		n=18	
	Pr(0)	Pr(>1)	Pr(0)	Pr(>1)
0.01	0.941	0.001	0.835	0.014
0.02	0.886	0.006	0.695	0.050
0.035	0.808	0.017	0.527	0.130
0.05	0.735	0.033	0.397	0.226
0.1	0.531	0.114	0.150	0.550
0.15	0.377	0.224	0.054	0.776
0.2	0.262	0.345	0.018	0.901
0.3	0.118	0.580	0.002	0.986

9.2.2 Sample Size Considerations for the Efficacy Study

The efficacy study is designed to evaluate protective efficacy (PE) by testing the null hypothesis,

H0: protective efficacy ≤ 0%,

versus the alternative hypothesis,

H1: protective efficacy > 0%,

where PE is 1 minus the ratio of the infection rate under CIS43LS over the infection rate under placebo.

The sample size considerations are based on the power of rejecting H0 over a range of possible protective efficacy and infection rates under placebo. Assuming a drop-out rate of 10%, Table 4 presents the power under a 2-sided significance level of 0.05. With 90 subjects enrolled in each arm, the trial has at least 80% power to claim protective efficacy of CIS43LS if the underlying efficacy is greater than or equal to 0.5 and the infection rate under placebo is no less than 0.4.

Table 4. Power for efficacy evaluation under a 2-sided type I error rate of 0.05.

Sample size per arm	Infection rate under placebo	Protective efficacy	Power
80	0.4	0.4	54
	0.4	0.5	75
	0.4	0.6	90
	0.5	0.4	69
	0.5	0.5	88
	0.5	0.6	97

Sample size per arm	Infection rate under placebo	Protective efficacy	Power
	0.6	0.4	83
	0.6	0.5	96
	0.6	0.6	100
	0.7	0.4	93
	0.7	0.5	99
	0.7	0.6	100
90	0.4	0.4	59
	**0.4**	**0.5**	**80**
	0.4	0.6	93
	0.5	0.4	74
	0.5	0.5	92
	0.5	0.6	98
	0.6	0.4	87
	0.6	0.5	98
	0.6	0.6	100
	0.7	0.4	96
	0.7	0.5	100
	0.7	0.6	100
100	0.4	0.4	64
	0.4	0.5	84
	0.4	0.6	95
	0.5	0.4	79
	0.5	0.5	94
	0.5	0.6	99
	0.6	0.4	90
	0.6	0.5	99
	0.6	0.6	100
	0.7	0.4	97
	0.7	0.5	100
	0.7	0.6	100

Table 5 presents the minimum detectable difference between 2 arms in terms of the AE rate based on a two-sided proportion test with type I error rate of 5%.

Table 5. Minimum detectable difference in adverse event rate in arms receiving monoclonal antibody (MAb) or placebo under a 2-sided type I error rate of 0.05.

Sample size within each arm (#)	Event rate (in placebo) (%)	Detectable with 80% power		Detectable with 90% power	
		Difference (%)	Event rate (in MAb) (%)	Difference (%)	Event rate (in MAb) (%)
80	0.1	9.4	9.5	12.2	12.3
	0.5	10	10.5	12.8	13.3
	1	10.7	11.7	13.5	14.5
	2	11.9	13.9	14.7	16.7
	5	14.4	19.4	17.4	22.4
90	0.1	8.4	8.5	11	11.1
	0.5	9.1	9.6	11.6	12.1
	1	9.8	10.8	12.3	13.3
	2	10.9	12.9	13.5	15.5
	5	13.3	18.3	16.1	21.1
100	0.1	7.6	7.7	10	10.1
	0.5	8.3	8.8	10.6	11.1
	1	9	10	11.3	12.3
	2	10.1	12.1	12.5	14.5
	5	12.4	17.4	15	20

9.3 POPULATIONS FOR ANALYSES

The following datasets will be considered in study analyses:

- Intention-to-treat (ITT) analysis dataset will include all subjects that receive assignment and will be analyzed according to the initial randomization assignment.
- Modified intention-to-treat (MITT) analysis dataset will include all randomized subjects that receive the study intervention and will be analyzed according to the initial randomization assignment.
- Per-protocol analysis dataset will include all randomized subjects that receive the study intervention and will be analyzed according to the actual intervention received.

9.3.1 Evaluable for Toxicity

All subjects will be evaluable for toxicity from the time of their first treatment with CIS43LS or placebo.

9.3.2 Evaluable for Objective Response

Not applicable.

9.3.3 Evaluable Non-Target Disease Response

Not applicable.

9.4 Statistical Analyses

9.4.1 General Approach

In general, descriptive statistics will be tabulated by treatment arm for endpoints of interest. This will include point estimates (mean, geometric mean, median, or proportions) and their respective 95% confidence intervals. Formal comparisons will use standard methods, contingency tables for categorical variables, t-tests for comparing means if data follow a normal distribution or geometric means if data after log transformation follow a normal distribution, or nonparametric analogs for comparing medians. Unless specified in the subsequent sections, comparisons will be two-sided with type I error rate of 0.05.

Missing data will be considered as "missing completely at random" provided missing data is modest (e.g., <10%). We will examine the "missing completely at random" assumption if missing data is more than 10%. If the assumption does not hold, missing data will be handled under the "missing at random" assumption (that is, missingness depends only on observed variables) via methods such as multiple imputation and inverse propensity weighting. To handle the possibility of "missing not at random," a sensitivity analysis will additionally be considered by imputing missing binary observations with the observed proportion in the opposite arm.

Randomization: In the efficacy study, randomization will be 1:1 allocation to the CIS43LS arm and the placebo arm. To limit the number of dropouts before vaccination, randomization and CIS43LS administration will occur as close in time as possible.

9.4.2 Analysis of the Primary Endpoints

Analysis for the primary endpoint for safety and tolerability is described in section 9.4.5.

Analysis for the primary efficacy endpoint, protective efficacy with Pf infection determined by blood smear, is described in section 9.4.4.

9.4.3 Analysis of the Secondary Endpoints

Analysis of the secondary efficacy endpoint, with Pf infection determined by PCR, is described in section 9.4.4.

Analyses of the secondary endpoints for the PK of CIS43LS and the association of CIS43LS concentration with Pf infection risk are described in section 9.4.6.

9.4.4 Efficacy Analyses

The primary efficacy endpoint (efficacy study primary endpoint) is the incidence of malaria infection defined as blood smear–positive Pf infection through 24 weeks after administration. The secondary efficacy endpoint is the incidence of malaria infection as determined by PCR. The efficacy analyses will be MITT.

The primary efficacy analysis will be based on time to the first infection. The survival patterns will be described by Kaplan-Meier curves for each arm and compared by the logrank test across different arms. The protective efficacy of the study product will be estimated by the hazard ratio from the Cox proportional hazards model. These analyses will be carried out by R packages that account for interval censoring: package “interval” for deriving a nonparametric maximum likelihood estimation based on the Kaplan-Meier survival curve and logrank test, and package “icenReg” for Cox proportional hazards regression. To address the heterogeneity of the study population, a Cox regression with regressors other than the study arm will be additionally performed to account for potential differences among participants. The regressors will include time of enrollment and possibly those covariates that are significantly different between the study product arm and the placebo arm in spite of randomization.

The secondary efficacy analysis will be based on the proportion of infection. The proportions of infection will be estimated for each arm and compared across arms based on Kaplan-Meier estimates along with 95% confidence intervals via R package “bpcp.” This estimation accounts for right censoring and the comparison should be equivalent to Fisher’s exact test in case of no censoring. Though not accounting for interval censoring, the Kaplan-Meier estimates are appropriate as the interest is on infection at 24 weeks only.

The above analyses will apply to both the primary and the secondary efficacy endpoints.

9.4.5 Safety Analyses

Safety analysis will be primarily MITT where individuals who receive assignment but do not receive any product are excluded. Because of blinding and the brief length of time between assignment and administration, such cases will be very few.

Safety data will be presented by line listing and tables at the individual level to provide details on safety events such as severity, duration, and relationship to study product. The number and percentage of subjects with 1 or more AEs will be summarized by dose arm along with the exact 95% confidence intervals of the AE rate. For subjects experiencing more than 1 AE, the subjects will be counted once under the event of highest severity.

In the efficacy study, comparisons between the dose arm and the control arm will be additionally performed in terms of the proportions of solicited AEs, related AEs, and SAEs.

In the rare case of subjects receiving a regimen different from assignment, a per-protocol analysis will be performed as a secondary analysis, which will include subjects according to the product they actually receive in the study.

9.4.6 Pharmacokinetics Analysis

PK analysis will be carried out for subjects in the dose-escalation study and the efficacy study with blood samples collected at defined timepoints as listed in section 1.3. The following PK analysis will be performed as needed.

Individual Subject Pharmacokinetic Analysis: A non-compartmental (NC) PK analysis will be performed on the CIS43LS concentration data generated from each subject. Individual subject and dosing arm concentration-versus-time profiles will be constructed in linear and semi-log scales. In the NC analysis, the maximum concentration (Cmax) and time of maximal

concentration (Tmax) will be taken directly from the observed data. The area under the concentrations vs. time curve (AUC) will be calculated using the trapezoidal method and determined out to the final concentration collected. If a subject's CIS43LS concentration falls below the quantitative limit (QL) of the assay, the sample with concentration below the QL will be assigned a CIS43LS concentration value of "0" for AUC calculations. In addition to the total AUC, partial AUCs will also be determined over certain time intervals. The time-weighted average concentrations (Cave) during these intervals will be calculated as the AUC divided by the AUC collection interval (e.g., $Cave_{0\text{-}16WK} = (AUC_{0\text{-}16WK}) / 16$ weeks). The terminal slope, λ_z, will be determined by regression of the terminal, log-linear portion of the concentration-versus-time profile. If the final PK sample has measurable CIS43LS concentrations greater than the assay QL, the AUC post-final PK collection ($AUC_{last\text{-}infinity}$) will be estimated as C_{last} / λ_z and $AUC_{0\text{-}infinity}$ will be calculated as the sum of $AUC_{0\text{-}last} + AUC_{last\text{-}infinity}$.

Population Pharmacokinetic Analyses: Based on preclinical PK results for CIS43LS and known PK behavior studies of MAbs, the two-compartment model will be used for population PK analysis. The population analysis will estimate compartmental PK parameters such as the clearance (CL), central and peripheral volumes of distribution (Vd1 and Vd2), and intercompartmental clearance (Q). Total volume of distribution at steady-state (Vdss), will be calculated as the sum of Vd1 + Vd2. Alpha and beta half-lives will be calculated from CL, Q, Vd1, and Vd2 using standard equations.[21]

To assess the association of CIS43LS concentration with protection, we will perform a Cox proportional hazards regression for the time to the first infection with CIS43LS concentration as a time-varying covariate. A logistic regression analysis will be additionally performed to model the infection rate as a function of CIS43LS concentration via the method of generalized estimating equation to account for repeated measures.

9.4.7 Baseline Descriptive Statistics

Treatment arms will be compared for baseline subject characteristics using descriptive statistics. For continuous variables, the mean or median will be calculated for each treatment arm. For categorial variables, the proportion under each category will be calculated for each arm.

9.4.8 Planned Interim Analyses

One interim safety analysis will be performed when safety data are available from the dose-escalation study subjects. The purpose of the interim analysis on safety data is to clear safety concerns for proceeding to the efficacy study. This interim analysis will not affect the power or type I error in the primary analysis on efficacy in the efficacy study.

9.4.9 Sub-group Analyses

Not applicable.

9.4.10 Tabulation of Individual Participant Data

Safety data will be presented by line listing and tables at the individual level, as described in section 9.4.5.

9.4.11 Exploratory Analyses

To explore the impact of CIS43LS on the genotype of infection-inducing parasites at the CSP locus, a genotypic sieve analysis will be performed to analyze CSP sequences of breakthrough parasites in the blood samples of infected subjects. The sieve analysis will differentiate protective efficacy against different genotypes of infection-inducing parasites with genotype defined by, for example, number of mismatches to the CIS43LS footprint.

10 REGULATORY AND OPERATIONAL CONSIDERATIONS

10.1 INFORMED CONSENT PROCESS

10.1.1 Consent Procedures and Documentation

The informed consent process for this study will involve obtaining initial community permission followed by individual informed consent.

10.1.1.1 Community Permission for the Conduct of the Study

Prior to the start of this study, community permission will be obtained as described in section 5.6. Following the process of Diallo and colleagues,[22] the community permission process will involve the following:

1. Study investigators/personnel explain the study to village leaders, including the village chief, family heads, women association, and elders.
2. The village leaders discuss the study with family heads and community members and relay any additional questions or concerns to the study personnel.
3. The study and the informed consent process are explained in detail to heads of families by study investigators/personnel.

Discussions during the community permission process will address the need for both a husband and wife to agree to avoid pregnancy for the specified period if a wife chooses to volunteer for the study.

10.1.1.2 Individual Informed Consent

The study informed consent form will be written in French. The study team will review the consent form word-for-word and will translate it orally into local languages, since most potential study subjects do not read or speak French. An independent witness who is not a member of the study team will verify that oral translations are accurate and that potential subjects understand the contents of the consent form.

Local households and families will be invited to come to the study clinic for review of the informed consent. At the consenting visit, the subject will read the consent form or have it explained to them (in cases of illiteracy). Individuals in each family will be separately consented, and not all individuals from a household need to participate. Individuals who agree to participate will sign or fingerprint (if illiterate) the consent form.

Also, a study comprehension examination will be conducted to make sure that the study is understood by the potential subjects prior to signing consent. The exam will be written in French

and translated orally into local languages. All incorrect responses will be reviewed, and individuals must orally confirm their understanding of all incorrect responses. A score of at least 80% correct responses is mandatory to enroll. For individuals scoring below 80%, study staff may choose to review study details again and reassess comprehension by repeating the examination. At the discretion of the investigator, any individual whose comprehension is questionable, regardless of score, may be excluded from enrollment.

10.1.2 Consent for Minors When They Reach the Age of Majority

Not applicable.

10.1.3 Telephone Consent

Not applicable.

10.1.4 Telephone Assent

Not applicable.

10.1.5 Participation of Subjects who are/become Decisionally Impaired

Adults unable to give consent are excluded from enrolling in the protocol. However, reconsent may be necessary and there is a possibility, though unlikely, that subjects could become decisionally impaired. For this reason and because subjects might not benefit from research participation (section 2.3.3), subjects who lose the ability to consent during participation will be withdrawn.

10.2 Study Discontinuation and Closure

The study may be temporarily suspended or permanently terminated as described in the halting rules (section 8.4.6). In addition to the reporting described in that section, the principal investigator(s) will promptly contact the study subjects, provide the reason(s) for the termination or suspension, and, if applicable, inform them of changes to study visit schedule.

The principal investigators will consult with the EC prior to resuming the study following a halt.

10.3 Confidentiality and Privacy

All records will be kept confidential to the extent provided by federal, state, and local law. The study monitors and other authorized representatives of the sponsor may inspect all documents and records required to be maintained by the investigator, including but not limited to, medical records. Records will be kept locked and data will be coded. Any personally identifiable information maintained for this study will be kept on restricted-access computers and networks. Personally identifiable information will only be shared with individuals authorized to receive it under this protocol. Individuals not authorized to receive personally identifiable information will be provided with coded information only, as needed. Clinical information will not be released without written permission of the subject, except as necessary for monitoring by the FMPOS EC, FDA, NIAID, Office for Human Research Protections (OHRP), the VRC, or the sponsor's designee.

To further protect the privacy of study subjects, a Certificate of Confidentiality has been issued by the NIH. This certificate protects identifiable research information from forced disclosure. It allows the investigator and others who have access to research records to refuse to disclose identifying information on research participation in any civil, criminal, administrative, legislative, or other proceeding, whether at the federal, state, or local level. By protecting researchers and institutions from being compelled to disclose information that would identify research subjects, Certificates of Confidentiality help achieve the research objectives and promote participation in studies by helping assure confidentiality and privacy to subjects.

Samples and data will be collected and stored under this protocol. All of the stored study research samples are labeled by a code that only the investigators can link to the subject. Samples are stored in secure research laboratories in locked freezers with limited access at the USTTB, Bamako, and the NIH. Data will be kept in password-protected computers. Only investigators or their designees will have access to the samples and data.

Samples and data acquired under this protocol will be tracked using BSI Systems software.

Any loss or unanticipated destruction of samples (for example, due to freezer malfunction) or data (for example, misplacing a printout of data with identifiers) that meets the definition of a reportable event will be reported to the FMPOS EC.

Additionally, subjects may decide at any point not to have their samples stored. In this case, the principal investigator will destroy all known remaining samples and report what was done to both the subject and to the EC. This decision will not affect the individual's participation in this protocol or any other protocols at NIH.

10.4 Future Use of Stored Specimens and Data

Subjects are consented at enrollment for permission to indefinite storage and future use of specimens and data. Samples, specimens, and data collected under this protocol may be used to study malaria and the immune system. Genetic testing may be performed.

Storage and Tracking: Access to and tracking of stored samples and data will be secured and limited as described above (section 10.3).

Disposition:

- In the future, other investigators (both at NIH and outside) may wish to use these samples and/or data for research purposes. If the planned research falls within the category of "human subjects research" on the part of the NIH researchers, EC review and approval will be obtained. This includes the NIH researchers sending out coded and linked samples or data and getting results that they can link back to their subjects.
- At the time of protocol completion, samples will be either destroyed or, after EC approval, transferred to another existing protocol. Data will be archived by the study team in compliance with requirements for retention of research records; alternatively, after EC and study sponsor approval, the data may be either destroyed or transferred to another repository.

10.5 Safety Oversight

Safety oversight is described in section 8.4.4.

10.6 Clinical Monitoring

According to the ICH E6(R2) GCP guidelines, section 5.18, and FDA 21 CFR 312.50, clinical protocols are required to be adequately monitored by the study sponsor. This study monitoring will be conducted according to the "NIAID Intramural Clinical Monitoring Guidelines." Monitors under contract to the NIAID/OCRPRO will visit the clinical research site to monitor aspects of the study in accordance with the appropriate regulations and the approved protocol. The objectives of a monitoring visit will be: 1) to verify the existence of signed informed consent documents and documentation of the consent process for each monitored subject; 2) to verify the prompt and accurate recording of all monitored data points in DFdiscover and prompt reporting of all SAEs; 3) to compare abstracted information entered into DFdiscover with individual subjects' records and source documents (subjects' charts, laboratory analyses and test results, physicians' progress notes, nurses' notes, and any other relevant original subject information); and 4) to help ensure investigators are in compliance with the protocol. The monitors also will inspect the clinical site regulatory files to ensure that regulatory requirements (OHRP, FDA) and applicable guidelines (ICH GCP) are being followed. During the monitoring visits, the investigator (and/or designee) and other study personnel will be available to discuss the study progress and monitoring visit.

The investigator (and/or designee) will make study documents (e.g., consent forms, DFdiscover abstracts) and pertinent hospital or clinical records readily available for inspection by the local EC, FDA, the site monitors, and the NIAID staff for confirmation of the study data.

A specific protocol monitoring plan will be discussed with the principal investigator and study staff prior to enrollment. The plan will outline the frequency of monitoring visits based on such factors as study enrollment, data collection status, and regulatory obligations.

10.7 Quality Assurance and Quality Control

During the study, the principal investigator and study team will be responsible for ensuring study activities are conducted in compliance with the protocol, ICH GCP, and applicable regulatory requirements. Each clinical site will perform internal quality management of study conduct, data and biological specimen collection, and documentation according to study standard operating procedures.

10.8 Data Handling and Record Keeping

10.8.1 Data Collection and Management Responsibilities

Study data will be maintained in CRFs and collected directly from subjects during study visits and telephone calls or will be abstracted from subjects' medical records. Source documents include all recordings of observations or notations of clinical activities, including CRFs, and all reports and records necessary to confirm the data abstracted for this study. Data entry into CRFs will be performed by authorized individuals. The investigator is responsible for assuring that the data collected are complete, accurate, and recorded in a timely manner. Study data, including

cumulative subject accrual numbers, should be generated via the chosen data capture method and submitted to the EC as needed.

10.8.2 Study Records Retention

The investigator is responsible for retaining all essential documents listed in the ICH GCP guidelines. Study records will be maintained by the principal investigator according to the timelines specified in 21 CFR 312.62 or a minimum of 7 years, and in compliance with institutional, EC, state, and federal medical records retention requirements, whichever is longest. All stored records will be kept confidential to the extent required by federal, state, and local law.

Should the investigator wish to assign the study records to another party and/or move them to another location, the investigator will provide written notification of such intent to OCRPRO/NIAID with the name of the person who will accept responsibility for the transferred records and/or their new location. NIAID will be notified in writing and written OCRPRO/NIAID permission shall be obtained by the site prior to destruction or relocation of research records.

10.9 PROTOCOL DEVIATIONS

It is the responsibility of the investigator to use continuous vigilance to identify and report deviations to the FMPOS EC according to NIH HRPP Policy 801 (as described in section 8.6.1). All deviations must be addressed in study source documents and reported to the NIAID Program Official and sponsor. The investigator is responsible for knowing and adhering to the reviewing EC requirements.

10.9.1 NIH Definition of Protocol Deviation

The definition of a protocol deviation is provided in section 8.4.1.

10.10 PUBLICATION AND DATA SHARING POLICY

10.10.1 Human Data Sharing Plan

This study will be conducted in accordance with the following publication and data sharing policies and regulations:

- NIH Public Access Policy, which ensures that the public has access to the published results of NIH funded research. It requires scientists to submit final peer-reviewed journal manuscripts that arise from NIH funds to the digital archive PubMed Central upon acceptance for publication.
- This study will comply with the NIH Data Sharing Policy and Policy on the Dissemination of NIH-Funded Clinical Trial Information and the Clinical Trials Registration and Results Information Submission rule. As such, this trial will be registered at ClinicalTrials.gov, and results information from this trial will be submitted to ClinicalTrials.gov. In addition, every attempt will be made to publish results in peer-reviewed journals. Data from this study may be requested from other researchers indefinitely after the completion of the primary endpoint by contacting Peter Crompton or LIG.

Human data generated in this study for future research will be shared as follows:

- De-identified or identified data with approved outside collaborators under appropriate agreements.
- De-identified results or data in publication and/or public presentations.

Data will be shared at the time of publication or shortly thereafter.

10.10.2 Genomic Data Sharing Plan

Not applicable.

10.11 COLLABORATIVE AGREEMENTS

10.11.1 Agreement Type

10.12 CONFLICT OF INTEREST POLICY

The independence of this study from any actual or perceived influence, such as by the pharmaceutical industry, is critical. Therefore, any actual conflict of interest of persons who have a role in the design, conduct, analysis, publication, or any aspect of this trial will be disclosed and managed. Furthermore, persons who have a perceived conflict of interest will be required to have such conflicts managed in a way that is appropriate to their participation in the design and conduct of this trial. The study leadership will follow policies and procedures for all study group members to disclose and manage all conflicts of interest and will establish a mechanism for the management of all reported dualities of interest.

11 ABBREVIATIONS

ADA	Anti-drug antibody
AE	Adverse event
ALT	Alanine transaminase
ANA	Anti-nuclear antibody
AR	Adverse reaction
AUC	Area under the curve
β-hCG	Beta-human choriogonadotropin
Cave	Time-weighted average concentrations
CBC	Complete blood count
CFA	Communauté Financière Africaine
CFR	Code of Federal Regulations
CHMI	Controlled human malaria infection
CIS43LS	VRC-MALMAB0100-00-AB
CL	Clearance
Cmax	Maximum concentration
CONSORT	Consolidated Standards of Reporting Trials
CPT	Cell preparation tube
Cr	Creatinine

CRF	Case report form
CRS	Cytokine release syndrome
CSO	Clinical Safety Office
CSP	Circumsporozoite protein
CTM	Clinical Trials Monitoring
DSMB	Data and safety monitoring board
EC	Ethics committee
ECG	Electrocardiogram
EDTA	Ethylenediaminetetraacetic acid
ELISA	Enzyme-linked immunosorbent assay
FDA	Food and Drug Administration
FMPOS	Faculté de Médecine Pharmacie d'Odontostomatologie
GCP	Good clinical practice
GLP	Good laboratory practices
GMP	Good manufacturing practices
HBV	Hepatitis B virus
HCV	Hepatitis C virus
HIV	Human immunodeficiency virus
HPF	High power field
HRPP	Human Research Protection Program
ICH	International Council on Harmonisation
Ig	Immunoglobulin
IND	Investigational new drug
ISM	Independent safety monitor
ITT	Intention to treat
IV	Intravenous(ly)
LIG	Laboratory of Immunogenetics
MAb	Monoclonal antibody
MITT	Modified intention to treat
MRTC	Malaria Research and Training Center
NC	Non-compartmental
NIAID	National Institute of Allergy and Infectious Diseases
NIH	National Institutes of Health
OCRPRO	Office of Clinical Research Policy and Regulatory Operations
OHRP	Office for Human Research Protections
OHSRP	Office of Human Subjects Research Protections
Pb	*Plasmodium berghei*
PBMC	Peripheral blood mononuclear cell
PCR	Polymerase chain reaction
Pf	*Plasmodium falciparum*
PK	Pharmacokinetics
Pr	Probability
Q	Intercompartmental clearance
QL	Quantitative limit

QTc	QT interval, corrected
RBC	Red blood cell
RDT	Rapid diagnostic test
REDCap	Research Electronic Data Capture
rPfCSP	Recombinant *Plasmodium falciparum* circumsporozoite protein
SAE	Serious adverse event
SAR	Suspected adverse reaction
SC	Subcutaneous(ly)
SERF	Safety Expedited Report Form
SMM	Sponsor medical monitor
SOA	Schedule of Activities
SOP	Standard operating procedure
SPZ	Sporozoite
SRCP	Safety review and communications plan
SST	Serum-separating tube
SUSAR	Serious and unexpected suspected adverse reaction
Tmax	Time of maximum concentration
UP	Unanticipated problem
UPnonAE	Unanticipated problem that is not an adverse event
US	United States
USTTB	University of Science, Techniques and Technologies of Bamako
VCMP	Vaccine Clinical Materials Program
Vd1, Vd2	Volumes of distribution
Vdss	Total volume of distribution at steady-state
VRC	Vaccine Research Center
WBC	White blood cell
WHO	World Health Organization

12 REFERENCES

1. WHO. World Malaria Report 2018. 2018.
2. Efficacy and safety of RTS,S/AS01 malaria vaccine with or without a booster dose in infants and children in Africa: final results of a phase 3, individually randomised, controlled trial. *Lancet (London, England).* 2015;386(9988):31-45.
3. Mahmoudi S, Keshavarz H. Efficacy of phase 3 trial of RTS, S/AS01 malaria vaccine: The need for an alternative development plan. *Hum Vaccin Immunother.* 2017;13(9):2098-2101.
4. Zalevsky J, Chamberlain AK, Horton HM, et al. Enhanced antibody half-life improves in vivo activity. *Nature biotechnology.* 2010;28(2):157-159.
5. Ko SY, Pegu A, Rudicell RS, et al. Enhanced neonatal Fc receptor function improves protection against primate SHIV infection. *Nature.* 2014;514(7524):642-645.

6. Gaudinski MR, Coates EE, Houser KV, et al. Safety and pharmacokinetics of the Fc-modified HIV-1 human monoclonal antibody VRC01LS: A Phase 1 open-label clinical trial in healthy adults. *Plos Med.* 2018;15(1):e1002493.

7. Kisalu NK, Idris AH, Weidle C, et al. A human monoclonal antibody prevents malaria infection by targeting a new site of vulnerability on the parasite. *Nat Med.* 2018;24(4):408-416.

8. Ledgerwood JE, Coates EE, Yamshchikov G, et al. Safety, pharmacokinetics and neutralization of the broadly neutralizing HIV-1 human monoclonal antibody VRC01 in healthy adults. *Clinical and experimental immunology.* 2015.

9. Gaudinski MR, Houser KV, Doria-Rose NA, et al. Safety and pharmacokinetics of broadly neutralising human monoclonal antibody VRC07-523LS in healthy adults: a phase 1 dose-escalation clinical trial. *The lancet HIV.* 2019;6(10):e667-e679.

10. Gaudinski MR, Coates EE, Novik L, et al. Safety, tolerability, pharmacokinetics, and immunogenicity of the therapeutic monoclonal antibody mAb114 targeting Ebola virus glycoprotein (VRC 608): an open-label phase 1 study. *Lancet (London, England).* 2019;393(10174):889-898.

11. Mejia R, Booth GS, Fedorko DP, et al. Peripheral blood stem cell transplant-related Plasmodium falciparum infection in a patient with sickle cell disease. *Transfusion.* 2012;52(12):2677-2682.

12. Seder RA, Chang LJ, Enama ME, et al. Protection against malaria by intravenous immunization with a nonreplicating sporozoite vaccine. *Science.* 2013;341(6152):1359-1365.

13. Shokoples SE, Ndao M, Kowalewska-Grochowska K, Yanow SK. Multiplexed real-time PCR assay for discrimination of Plasmodium species with improved sensitivity for mixed infections. *J Clin Microbiol.* 2009;47(4):975-980.

14. CIS43LS [investigator's brochure]. Bethesda, MD: NIAID Vaccine Research Center; 2019.

15. Hansel TT, Kropshofer H, Singer T, Mitchell JA, George AJ. The safety and side effects of monoclonal antibodies. *Nat Rev Drug Discov.* 2010;9(4):325-338.

16. Bugelski PJ, Achuthanandam R, Capocasale RJ, Treacy G, Bouman-Thio E. Monoclonal antibody-induced cytokine-release syndrome. *Expert Rev Clin Immunol.* 2009;5(5):499-521.

17. Vogel WH. Infusion reactions: diagnosis, assessment, and management. *Clinical journal of oncology nursing.* 2010;14(2):E10-21.

18. Drug allergy: an updated practice parameter. *Annals of allergy, asthma & immunology : official publication of the American College of Allergy, Asthma, & Immunology.* 2010;105(4):259-273.

19. Coartem (artemether and lumefantrine) tablets [package insert]. East Hanover, NJ: Novartis; 2019.

20. Tran TM, Li S, Doumbo S, et al. An intensive longitudinal cohort study of Malian children and adults reveals no evidence of acquired immunity to Plasmodium falciparum infection. *Clin Infect Dis.* 2013;57(1):40-47.

21. Gibaldi M, Perrier D. *Pharmacokinetics.* New York, NY: Marcel Dekker, Inc.; 1975.

22. Diallo DA, Doumbo OK, Plowe CV, Wellems TE, Emanuel EJ, Hurst SA. Community permission for medical research in developing countries. *Clinical infectious diseases : an official publication of the Infectious Diseases Society of America.* 2005;41(2):255-259.

APPENDIX A: MRTC CLINICAL LABORATORY NORMAL VALUES

Chemistry

Chemistry[1]	Reference Range
Creatinine (Female) – µmol/L	< 72
Creatinine (Male) – µmol/L	48-98
ALT – U/L	< 41

Abbreviations: ALT, alanine transaminase.

[1]The laboratory values provided in the table are based on Bancoumana, Malian adults (age 18-45 years).

Hematology

Hematology[1]	Reference Range
Hemoglobin (Female) – gm/dL	9.1 – 13.8
Hemoglobin (Male) – gm/dL	10.8 – 15.8
WBC – 10^3/µL	3.6 – 9.0
Absolute Neutrophil/Granulocyte Count – 10^3/µL	1.3 – 4.4
Absolute Lymphocyte Count – 10^3/µL	1.3 – 4.4
Platelet Count (Female) – 10^3/µL	144 – 413
Platelet Count (Male) – 10^3/µL	114 – 335

Abbreviations: ALT, alanine transaminase; WBC, white blood cell.

[1] The laboratory values provided in the table are based on Bancoumana, Malian adults (age 18-45 years).

Urine Dip/Urinalysis

Urine[1]	Reference Ranges
Protein	None or Trace
Blood (Microscopic) –<br><br>RBC/HPF	None or Trace<br><br>< 5

Abbreviations: HPF, high power field; RBC, red blood cell.

[1] The laboratory values provided in the table are based on Bancoumana, Malian adults (age 18-45 years).

Abbreviated Title: Anti-malaria MAb in Mali

FMPOS Protocol #: 2020/32/CE/FMOS/FAPH

Version Date: October 13, 2022

Title: Safety and Efficacy of VRC-MALMAB0100-00-AB (CIS43LS), a Human Monoclonal Antibody against *Plasmodium falciparum*, in a Dose-Escalation Trial and a Randomized, Double-Blind Trial of Adults in Mali

FMPOS Principal Investigator: Kassoum Kayentao, MD, MPH, PhD
Malaria Research and Training Center (MRTC)
Faculté de Médecine Pharmacie d'Odontostomatologie (FMPOS)
University of Sciences, Techniques, & Technologies of Bamako (USTTB)

NIH Principal Investigator: Peter D. Crompton, MD, MPH
Laboratory of Immunogenetics (LIG)
National Institute of Allergy and Infectious Diseases (NIAID)
National Institutes of Health (NIH)

Investigational Agent:

Drug Name	VRC-MALMAB0100-00-AB (CIS43LS)
Investigational New Drug (IND) Number	147485
Sponsor	Office of Clinical Research Policy and Regulatory Operations (OCRPRO), Division of Clinical Research, NIAID
Manufacturer	Vaccine Research Center (VRC), NIAID

Data and Safety Monitoring Board (DSMB): NIAID Intramural DSMB

STUDY STAFF ROSTER

FMPOS Investigators:

The research activities of the following FMPOS investigators will be reviewed by:

FMPOS Ethics Committee
BP E302, Point G/FMPOS
Bamako, Mali
FWA #00001769

FMPOS Principal Investigator: Kassoum Kayentao, MD, MPH, PhD
MRTC, FMPOS, USTTB
[redacted]
Phone: [redacted]
Email: [redacted]
Role(s): A, B, C, D, E, F

FMPOS Associate Investigators: Boubacar Traore, PharmD, PhD (Senior Investigator)
MRTC, FMPOS, USTTB
Phone: [redacted]
Email: [redacted]
Role(s): A, B, C, D, E, F

Aissata Ongoiba, MD, MPH
MRTC, FMPOS, USTTB
Phone: [redacted]
Email: [redacted]
Role(s): A, B, C, D, E, F

Safiatou Doumbo, MD, MS
MRTC, FMPOS, USTTB
Phone: [redacted]
Email: [redacted]
Role(s): A, B, C, E, F

Didier Doumtabe, PharmD, MS
MRTC, FMPOS, USTTB
Phone: [redacted]
Email: [redacted]
Role(s): A, B, C, E, F

Abdrahamane Traore, MD
MRTC, FMPOS, USTTB
Phone: [redacted]
Email: [redacted]
Role(s): A, B, C, E, F

US Investigators:

NIH investigators are involved in study design, implementation, analysis of coded samples and data, and writing and dissemination of reports of study results. Although they may support Malian investigators in monitoring/oversight capacities, NIH investigators will not be engaged in human subjects research.

NIH Principal Investigator and Referral Contact: Peter D. Crompton, MD, MPH
LIG, NIAID, NIH
[redacted]
Phone: [redacted]
Email: pcrompton@niaid.nih.gov
Role(s): F

NIH Employee Associate Investigators: Robert A. Seder, MD (Senior Investigator)
Chief, Cellular Immunology Section
VRC, NIAID, NIH
Phone: [redacted]
Email: [redacted]
Role(s): F

Non-NIH Employee Associate Investigators:

Sara Healy, MD, MPH (Contractor)
Medical Science & Computing, LLC
In support of LIG, NIAID, NIH
Phone: [redacted]
Email: [redacted]
Role(s): F

Anne C. Preston, RN, BS, CCRC (Contractor)
Gap Solutions, Inc.
In support of LIG, NIAID, NIH
Phone: [redacted]
Email: [redacted]
Role(s): F

Sean C. Murphy, MD, PhD
University of Washington
Department of Laboratory Medicine, [redacted]
Phone: [redacted]
Email: [redacted]
Role(s): F

Daniel Neafsey, PhD
Harvard T.H. Chan School of Public Health
[redacted]
Phone: [redacted]
Email: [redacted]
Role(s): F

Statistician:

Zonghui Hu, PhD
Biostatistics Research Branch, NIAID, NIH
Phone: [redacted]
Email: [redacted]
Role(s): F

For each person listed above, identify their roles with the appropriate letter:

A. *Obtain information by intervening or interacting with living individuals for research purposes*
B. *Obtaining identifiable private information about living individuals*
C. *Obtaining the voluntary informed consent of individuals to be subjects*

D. *Makes decisions about subject eligibility*
E. *Studying, interpreting, or analyzing identifiable private information or data/specimens for research purposes*
F. *Studying, interpreting, or analyzing coded (linked) data or specimens for research purposes*
G. *Some/all research activities performed outside NIH*

Study Sites:

Kalifabougou MRTC Clinic
Kalifabougou, Mali
Région de Koulikoro
Préfecture de Kati, Commune de Kalifabougou, Centre de Santé Communautaire de Kalifabougou

Torodo MRTC Clinic
Torodo, Mali
Région de Koulikoro
Préfecture de Kati, Commune de Torodo, Centre de Santé Communautaire de Torodo

Sponsor Medical Monitor (SMM):

Shirley Jankelevich, MD (Contractor)
Leidos Biomedical Research, Inc.
Support to: OCRPRO, NIAID, NIH
Phone: [redacted]
Email: [redacted]

Independent Safety Monitor (ISM):

Daouda K. Minta, MD
Point-G Hospital
USTTB
Phone: [redacted]
Email: [redacted]

TABLE OF CONTENTS

STATEMENT OF COMPLIANCE 11
1 PROTOCOL SUMMARY 12
1.1 Synopsis 12
1.2 Schema 15
1.3 Schedule of Activities (SOA) 17
1.3.1 Dose-escalation Study 17
1.3.2 Efficacy Study 19
2 INTRODUCTION 22
2.1 Study Rationale 22
2.2 Background 22
2.2.1 Overview 22
2.2.2 Study Product: CIS43LS 23
2.2.3 Laboratory Assessments of CIS43LS 27
2.3 Risk/Benefit Assessment 28
2.3.1 Known Potential Risks 28
2.3.2 Known Potential Benefits 30
2.3.3 Assessment of Potential Risks and Benefits 30
3 OBJECTIVES AND ENDPOINTS 30
4 STUDY DESIGN 32
4.1 Overall Design 32
4.2 Scientific Rationale for Study Design 33
4.3 Justification for Dose 33
5 STUDY POPULATION 34
5.1 Inclusion Criteria 34
5.2 Exclusion Criteria 34
5.3 Inclusion or Exclusion of Vulnerable Participants 35
5.4 Lifestyle Considerations 36
5.5 Screen Failures 36
5.6 Strategies for Recruitment and Retention 36
5.6.1 Costs 36
5.6.2 Compensation 37

6 STUDY INTERVENTION ... 37
6.1 Study Intervention Administration ... 37
6.1.1 Study Intervention Description ... 37
6.1.2 Dosing and Administration ... 37
6.2 Preparation/Handling/Storage/Accountability ... 39
6.2.1 Acquisition and Accountability ... 39
6.2.2 Formulation, Appearance, Packaging, and Labeling ... 39
6.2.3 Product Storage and Stability ... 39
6.2.4 Preparation ... 40
6.3 Measures to Minimize Bias: Randomization and Blinding ... 40
6.3.1 Randomization ... 40
6.3.2 Blinding ... 40
6.4 Study Intervention Compliance ... 41
6.5 Concomitant Therapy ... 41
7 STUDY INTERVENTION DISCONTINUATION AND PARTICIPANT DISCONTINUATION/WITHDRAWAL ... 41
7.1 Discontinuation of Study Intervention ... 41
7.2 Participant Discontinuation/Withdrawal from the Study ... 42
7.3 Lost to Follow-up ... 42
8 STUDY ASSESSMENTS AND PROCEDURES ... 42
8.1 Screening Procedures ... 42
8.2 Efficacy Assessments ... 43
8.2.1 Clinical Evaluations ... 43
8.2.2 Biospecimen Evaluations ... 44
8.2.3 Correlative Studies for Research/Pharmacokinetic Studies ... 45
8.2.4 Samples for Genetic/Genomic Analysis ... 45
8.3 Safety and Other Assessments ... 45
8.4 Safety Definitions, Management, and Sponsor Reporting ... 46
8.4.1 Definitions ... 46
8.4.2 Documenting, Assessing, Recording, and Reporting Events ... 49
8.4.3 Withdrawal Criteria for an Individual Subject ... 55
8.4.4 Additional Safety Oversight ... 56

8.4.5 Pausing Rules ... 57
8.4.6 Halting Rules for the Protocol ... 58
8.5 Unanticipated Problems ... 59
8.5.1 Definition of an Unanticipated Problem ... 59
8.5.2 Unanticipated Problem Reporting ... 59
8.6 ADDITIONAL REPORTING REQUIREMENTS ... 59
8.6.1 Reporting to the FMPOS EC ... 59
8.6.2 Reporting to the NIAID Clinical Director ... 60
8.6.3 Reporting Protocol Deviations that Result from the COVID-19 Pandemic ... 60
9 STATISTICAL CONSIDERATIONS ... 60
9.1 Statistical Hypothesis ... 60
9.2 Sample Size Determination ... 61
9.2.1 Sample Size Considerations for the Dose-Escalation Study ... 61
9.2.2 Sample Size Considerations for the Efficacy Study ... 61
9.3 Populations for Analyses ... 64
9.3.1 Evaluable for Toxicity ... 64
9.3.2 Evaluable for Objective Response ... 64
9.3.3 Evaluable Non-Target Disease Response ... 64
9.4 Statistical Analyses ... 64
9.4.1 General Approach ... 64
9.4.2 Analysis of the Primary Endpoints ... 65
9.4.3 Analysis of the Secondary Endpoints ... 65
9.4.4 Efficacy Analyses ... 65
9.4.5 Safety Analyses ... 66
9.4.6 Pharmacokinetics Analysis ... 66
9.4.7 Baseline Descriptive Statistics ... 67
9.4.8 Planned Interim Analyses ... 67
9.4.9 Sub-group Analyses ... 67
9.4.10 Tabulation of Individual Participant Data ... 67
9.4.11 Exploratory Analyses ... 67
10 REGULATORY AND OPERATIONAL CONSIDERATIONS ... 68
10.1 Informed Consent Process ... 68

10.1.1 Consent Procedures and Documentation ... 68
10.1.2 Consent for Minors When They Reach the Age of Majority ... 69
10.1.3 Telephone Consent ... 69
10.1.4 Telephone Assent ... 69
10.1.5 Participation of Subjects who are/become Decisionally Impaired ... 69
10.2 Study Discontinuation and Closure ... 69
10.3 Confidentiality and Privacy ... 69
10.4 Future Use of Stored Specimens and Data ... 70
10.5 Safety Oversight ... 70
10.6 Clinical Monitoring ... 70
10.7 Quality Assurance and Quality Control ... 71
10.8 Data Handling and Record Keeping ... 71
10.8.1 Data Collection and Management Responsibilities ... 71
10.8.2 Study Records Retention ... 71
10.9 Protocol Deviations ... 72
10.9.1 NIH Definition of Protocol Deviation ... 72
10.10 Publication and Data Sharing Policy ... 72
10.10.1 Human Data Sharing Plan ... 72
10.10.2 Genomic Data Sharing Plan ... 72
10.11 Collaborative Agreements ... 73
10.11.1 Agreement Type ... 73
10.12 Conflict of Interest Policy ... 73
11 ABBREVIATIONS ... 73
12 REFERENCES ... 75
APPENDIX A: MRTC URINE LABORATORY NORMAL VALUES ... 77
APPENDIX B: MALI ADVERSE EVENT GRADING SCALE ... 78
APPENDIX C: DOSE ESCALATION STUDY EXTENSION ... 79
APPENDIX D: PREGNANCY FOLLOW-UP ... 81

List of Tables

Table 1. Study agent assignment and dosing by arm. ... 37
Table 2. Standard event recording, assessment, and reporting timeframes. ... 50

Table 3. Probability (Pr) of events for different safety scenarios within an arm (n=6 or 18)....... 61
Table 4. Power for efficacy evaluation under a 2-sided type I error rate of 0.025. 62
Table 5. Minimum detectable difference in adverse event rate in arms receiving monoclonal antibody (MAb) or placebo under a 2-sided type I error rate of 0.025.. 63
Table 6. Schedule of assessments for neonate/infant participants. 81

List of Figures

Figure 1. Dose-escalation study flow diagram. 15
Figure 2. Efficacy study flow diagram. 16

STATEMENT OF COMPLIANCE

The trial will be carried out in accordance with International Council on Harmonisation (ICH) Good Clinical Practice (GCP) and the following:

- United States (US) Code of Federal Regulations (CFR) applicable to clinical studies (45 CFR Part 46, 21 CFR Part 50, 21 CFR Part 56, 21 CFR Part 312, and/or 21 CFR Part 812)

NIH-funded investigators and clinical trial site staff who are responsible for the conduct, management, or oversight of NIH-funded clinical trials have completed Human Subjects Protection and ICH GCP Training.

The protocol and informed consent forms will be submitted to the FMPOS Ethics Committee (EC) for review and approval. Approval of both the protocol and the consent form must be obtained before any subject is enrolled. Any amendment to the protocol will require review and approval by the EC before the changes are implemented to the study. In addition, all changes to the consent form will be EC-approved; a determination will be made regarding whether a new consent needs to be obtained from subjects who provided consent, using a previously approved consent form.

1 PROTOCOL SUMMARY

1.1 SYNOPSIS

**Title:**	Safety and Efficacy of VRC MALMAB0100-00-AB (CIS43LS), a Human Monoclonal Antibody against Plasmodium falciparum, in a Dose-Escalation Trial and a Randomized, Double-Blind Trial of Adults in Mali
**Study Description:**	A two-part phase 2 trial evaluating the safety and tolerability of one-time administration of CIS43LS, as well as its protective efficacy against naturally occurring *Plasmodium falciparum* (Pf) infection over a 6-month malaria season. The primary study hypotheses are that CIS43LS will be safe and will produce protection against malaria infection. Before study agent administration, all subjects will be given artemether-lumefantrine to clear any preexisting Pf blood-stage infection.<br><br>**Dose-escalation study:** The first part of the study is an open-label dose-escalation study for safety and tolerability. Subjects will be assigned to 1 of 3 dose arms (N=18 total, n=6 for each dose level). Dosing will begin in the lowest dose arm. Once all subjects in that arm reach day 7 post-infusion, if no safety concerns have arisen, dosing will begin at the subsequent dose level. This process will be repeated until subjects complete the third dose arm. Subjects will be followed for safety to assess adverse events (AEs) at study visits 1, 3, 7, 14, 21, and 28 days after administration, then monthly through 24 weeks after administration. Participants who complete participation in the dose-escalation will be offered participation in extension visits to collect extra bi-weekly blood samples for pharmacokinetic (PK) analysis and to monitor for Pf infection through the end of December 2021.<br><br>After the last subject in the highest dose arm reaches day 7 safety follow-up, an interim safety evaluation will be performed before enrollment begins for the second part of the study.<br><br>**Efficacy study:** The second part of the study is a randomized, double-blind, placebo-controlled trial (N=330 total, n=110 for each of 3 treatment arms) to assess safety and protective efficacy of CIS43LS and placebo.<br><br>Subjects in the efficacy study will receive the study agent and be followed at study visits 1, 3, 7, 14, 21, and 28 days later, and once every 2 weeks thereafter through 24 weeks. Primary study assessments include physical examination and blood collection for identification of Pf infection and other research laboratory evaluations.
**Objectives:**	Primary Objectives:<br>1. Dose escalation: To evaluate the safety and tolerability of CIS43LS administered intravenously (IV) at 5, 10, and 40 mg/kg in healthy Malian adults.<br>2. Efficacy: To determine if IV administration of CIS43LS at 10 mg/kg and 40 mg/kg (compared to placebo) mediates protection against naturally occurring Pf infection in healthy Malian adults during a

	single malaria season as detected from microscopic examination of thick blood smear.
	Secondary Objectives:<br>1. Dose escalation: To evaluate the PK of CIS43LS at each dose level throughout the study.<br>2. Efficacy: To determine if IV administration of CIS43LS at 10 mg/kg and 40 mg/kg (compared to placebo) mediates protection against naturally occurring Pf infection in healthy Malian adults during a single malaria season as detected by reverse transcription polymerase chain reaction (RT-PCR).<br>3. Efficacy: To evaluate the PK of CIS43LS throughout the study at the dose of 10 mg/kg and 40 mg/kg and to correlate CIS43LS serum concentration with Pf infection risk.
**Endpoints:**	Primary Endpoints:<br>1. Dose escalation: Incidence and severity of local and systemic AEs occurring within 7 days after the administration of CIS43LS.<br>2. Efficacy: Pf blood stage infection as detected by microscopic examination of thick blood smear for 24 weeks after administration of CIS43LS or placebo.<br>Secondary Endpoints:<br>1. Dose escalation: Measurement of CIS43LS in sera of recipients.<br>2. Efficacy: Pf blood stage infection as detected by RT-PCR for 24 weeks after administration of CIS43LS or placebo.<br>3. Efficacy: Measurement of CIS43LS in sera of recipients.
**Study Population:**	Healthy Malian adults (aged 18 to 55 years) residing in Kalifabougou and Torodo
**Phase:**	2
**Description of Sites/Facilities Enrolling Subjects:**	The MRTC clinics in Kalifabougou and Torodo. All screening, day 0 (study agent administration) and day 7 visits will be performed at Kalifabougou only. Both sites will conduct recruitment, enrollment, follow-up visits (other than day 7 as noted above) and unscheduled study visits.
**Description of Study Intervention:**	The study agent (CIS43LS or matching placebo) will be administered as a one-time IV infusion.<br>Dose-escalation study subjects will receive 1 of 3 different dose levels; study agent will be administered starting with the lowest dose level:<br>• Arm 1: 5 mg/kg IV<br>• Arm 2: 10 mg/kg IV<br>• Arm 3: 40 mg/kg IV<br>Efficacy study subjects will be randomized 1:1:1 to receive 1 of the following:

	• Arm 1: 10 mg/kg IV<br>• Arm 2: 40 mg/kg IV<br>• Arm 3: Placebo (normal saline) IV
**Study Duration:**	23 months
**Subject Duration:**	Dose-escalation study: 8 months, plus approximately 3 months optional extension visits<br><br>Efficacy study: 8 months

1.2 SCHEMA

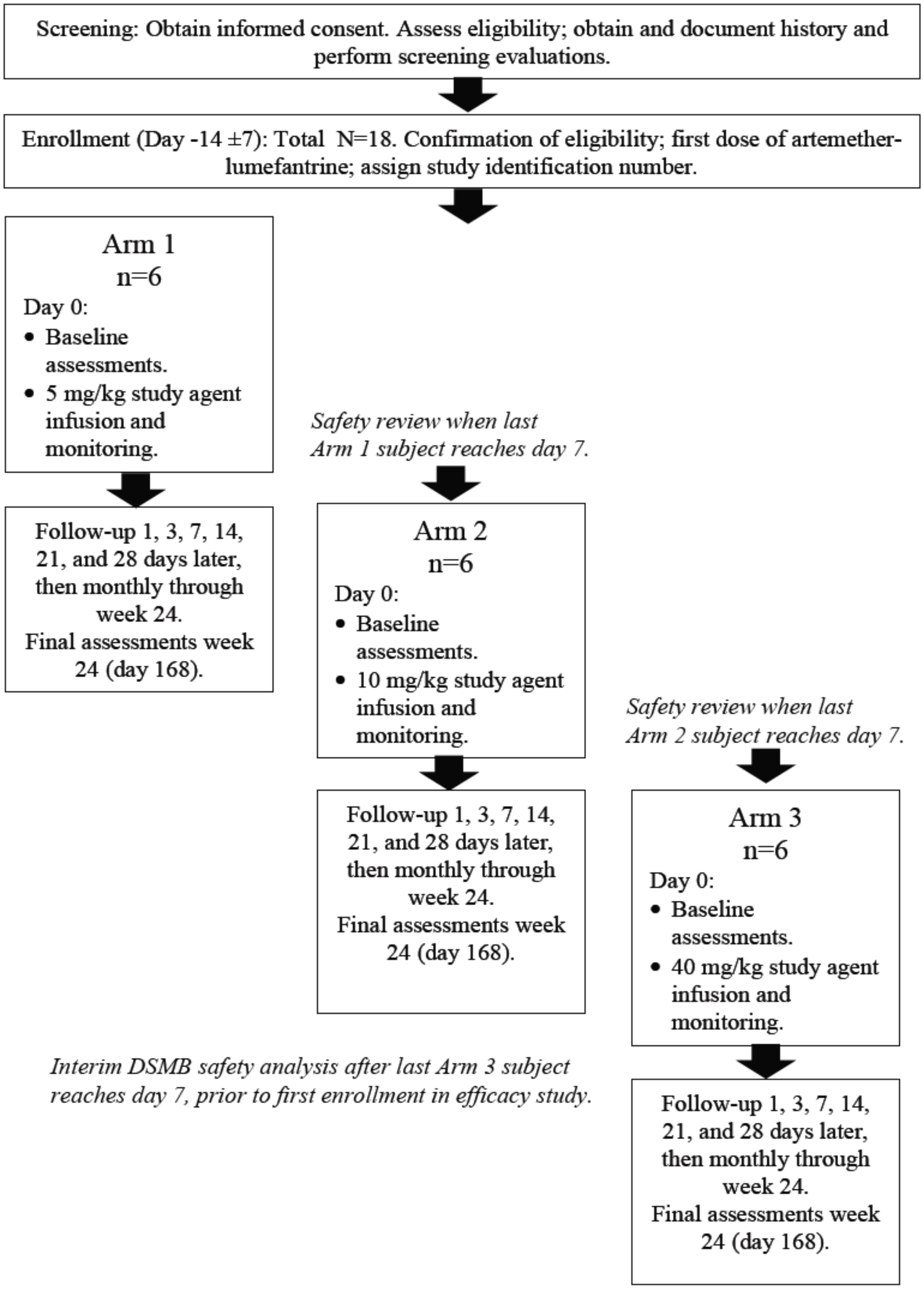

Figure 1. Dose-escalation study flow diagram.

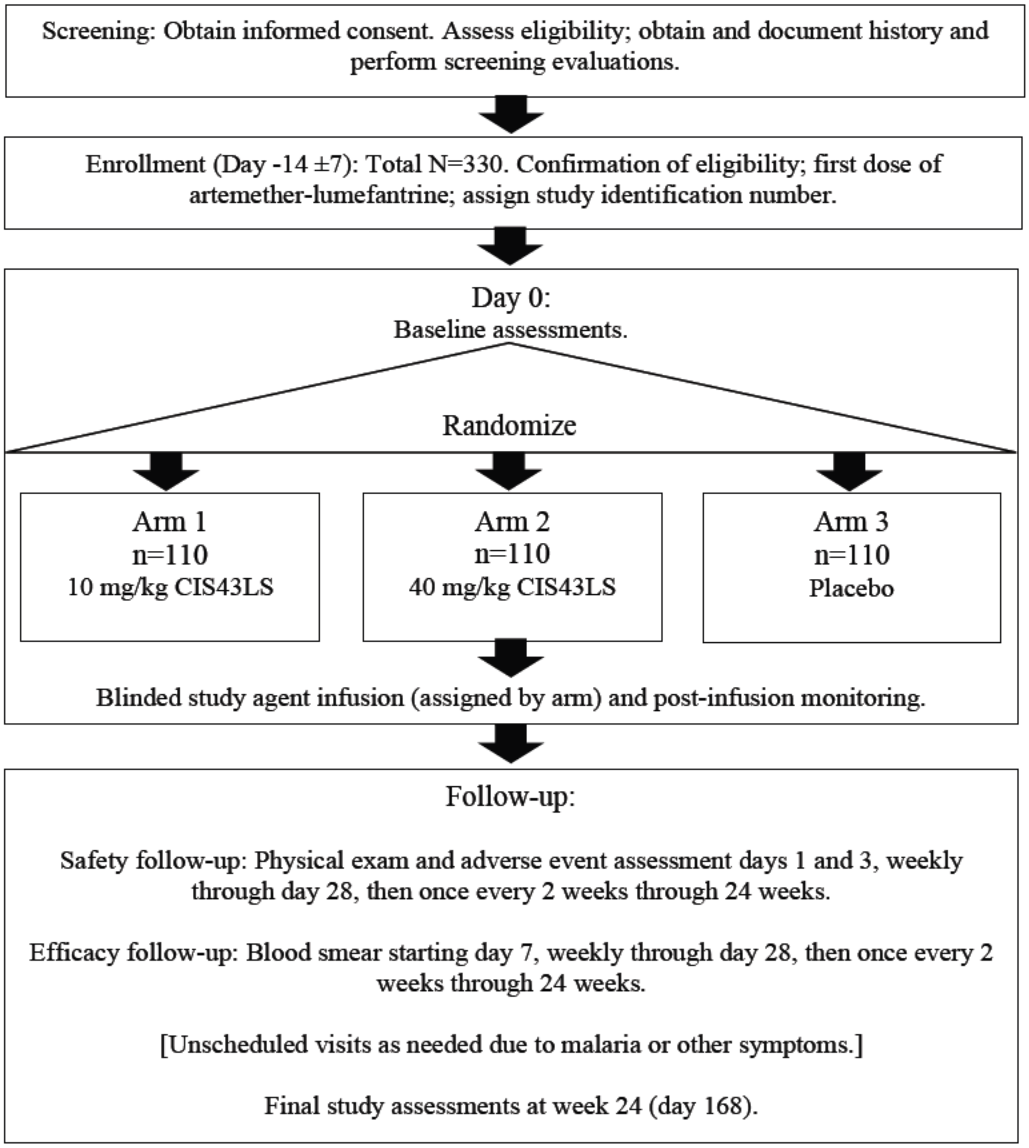

Figure 2. Efficacy study flow diagram.

1.3 Schedule of Activities (SOA)

1.3.1 Dose-escalation Study

**Study Day**		**Screen**	**Enroll**[1]	**0**	**1**	**3**	**7**	**14**	**21**	**28**	**56**	**84**	**112**	**140**	**168**	**Illness Visit**	**ET Visit**
Window (days)		-56 to -7	-14±7	-	+1	±1	±2	±3	±3	±3	±7	±7	±7	±7	±7		
**Clinical Procedures/Evaluations**																	
Study comprehension exam		X															
Informed consent		X															
Confirmation of identity, age, and residency		X															
Physical exam [2]		X	X	X	X	X	X	X	X	X	X	X	X	X	X	X	X
Height		X															
Weight		X		X													
Vital signs (temperature, blood pressure, and pulse)		X		X	X	X	X	X	X	X	X	X	X	X	X	X	X
Medical history [2]		X	X	X	X	X	X	X	X	X	X	X	X	X	X	X	X
Concomitant medications [2]		X	X	X	X	X	X	X	X	X	X	X	X	X	X	X	X
Artemether-lumefantrine administration			X [3]														
ECG		X															
Study agent administration				X [4]													
Adverse events			X	X	X	X	X	X	X	X	X	X	X	X	X		X
Pregnancy prevention counseling		X	X	X	X	X	X	X	X	X	X	X	X	X	X		X
**Laboratory Evaluations**																	
Test	Tube	(Blood volume in mL)															
Urinalysis	-	X															
Pregnancy test (urine/serum; for women)	-	X	X	X [5]						X	X	X	X	X	X		
HIV, HBV, HCV screen [6]	SST	3															
CBC with differential	EDTA	3	3	3 [5]		3	3	3									

**Study Day**		**Screen**	**Enroll**[1]	**0**	**1**	**3**	**7**	**14**	**21**	**28**	**56**	**84**	**112**	**140**	**168**	**Illness Visit**	**ET Visit**
Window (days)		-56 to -7	-14±7	-	+1	±1	±2	±3	±3	±3	±7	±7	±7	±7	±7		
Hemoglobin type	EDTA	(X)[7]															
ALT, Cr	SST	3	3	3[5]		3	3	3									
Blood smear and dried blood spot for Pf RT-PCR[8]	-		X	X				X	0.5	X	X	X	X	X	X	X	
PK studies	SST			8[9]	4		4	4		4	4	4	4	4	4		
Serum storage	SST			8			8			8		8			8		
ADA	SST			(X)			(X)			(X)		(X)			(X)		
PBMC storage	CPT			16			16										
**Daily volume (mL)**		**9**	**6**	**38**	**4**	**6**	**34**	**10**	**0.5**	**12**	**4**	**12**	**4**	**4**	**12**		
**Cumulative volume (mL)**		**9**	**15**	**53**	**57**	**63**	**97**	**107**	**107.5**	**119.5**	**123.5**	**135.5**	**139.5**	**143.5**	**155.5**	-	-

Abbreviations: ADA, anti-drug antibodies; ALT, alanine transaminase; CBC, complete blood count; CPT, cell preparation tube; Cr, creatinine; ECG, electrocardiogram; EDTA, ethylenediaminetetraacetic acid; ELISA, enzyme-linked immunosorbent assay; ET, early termination; HBV, hepatitis B virus; HCV, hepatitis C virus; HIV, human immunodeficiency virus; PBMC, peripheral blood mononuclear cell; Pf, *Plasmodium falciparum*; PK, pharmacokinetics; RDT, rapid diagnostic test; RT-PCR, reverse transcription polymerase chain reaction; SST, serum-separating tube.

(X) indicates that no additional blood will be drawn; the test will be performed from the tube shown in the preceding row.

Notes:

- Screening, day 0, and day 7 visits will take place at Kalifabougou for all subjects, regardless of residence. All other visits can take place at either site.
- At any time during the study, the subject may have an unscheduled illness visit if experiencing malaria symptoms or other symptoms. The subject may be referred for standard care according to local guidelines. Unscheduled illness visits will take place at Kalifabougou or Torodo, depending on the subject's residence.
- Participants who complete participation in the dose-escalation will be offered participation in extension visits to collect extra bi-weekly blood samples for PK analysis and to monitor for Pf infection through the end of December 2021. See Appendix C for details.

Footnotes:

[1] If enrollment is within 2 days of screening, duplicate procedures will not be repeated.

[2] Complete/comprehensive at screening; targeted/interim at other visits.

[3] Enrollment is defined as the time of first artemether-lumefantrine administration. (For women, negative pregnancy test must be confirmed prior to administration/enrollment.) The first dose will be directly observed in the clinic. The subsequent 5 doses given over 3 days will be observed by guides in the subject's home or at the clinic. All artemether-lumefantrine doses will be completed prior to day 0.

[4] All other study procedures must be completed prior to study agent infusion. Subject will be monitored during and after each infusion, and vital signs will be recorded directly after infusion and hourly during post-infusion monitoring. The first subject in a dose arm will be monitored for at least 4 hours after completion of infusion; all other subjects will be monitored for at least 2 hours after completion of infusion. Prior to discharge, subject will be assessed for local reactogenicity (including pain/tenderness, swelling, redness, bruising, and pruritus at the site of infusion) and systemic reactogenicity (including fever, feeling unusually tired or unwell, muscle aches, headache, chills, nausea, and joint

**Study Day**	**Screen**	**Enroll**[1]	**0**	**1**	**3**	**7**	**14**	**21**	**28**	**56**	**84**	**112**	**140**	**168**	**Illness Visit**	**ET Visit**
Window (days)	-56 to -7	-14±7	-	+1	±1	±2	±3	±3	±3	±7	±7	±7	±7	±7		

pain). If the subject is assessed as being unwell or has ongoing reactogenicity symptoms, he or she will be asked to remain in the clinic until evaluation and discharge by a study clinician. Clinicians will follow any solicited symptoms that are ongoing until they have resolved.
[5] Collected prior to study agent infusion. For women, negative pregnancy test must be confirmed prior to infusion. Other indicated tests may be performed after infusion.
[6] Viral screenings will be performed according to international guidelines. HIV testing will be 2 rapid diagnostic tests (RDTs), plus enzyme-linked immunosorbent assay (ELISA) if the RDTs are discordant; subject will be referred for medical care for 2 positive RDTs or a positive ELISA. Pre- and post-test HIV counseling will be provided. Hepatitis testing will be an HBV surface antigen test (ELISA) and HCV test (ELISA, PCR if indicated). A subject who is HBV and/or HCV positive will be referred for care regardless of the ALT result.
[7] EDTA tube will be stored at screening, and hemoglobin typing will be performed if the subject is enrolled; this test will not be used in eligibility assessments.
[8] If positive for malaria parasite infection, parasite genotyping may be performed. Sample can be obtained via finger prick or venipuncture.
[9] For PK, 4 mL serum will be collected prior to study agent infusion, and an additional 4 mL serum will be collected about 1 hour (±30 minutes) after the infusion.

1.3.2 Efficacy Study

**Study Day**	**Screen**	**Enroll**[1]	**0**	**1**	**3**	**7**	**14**	**21**	**28**	**42**	**56**	**70**	**84**	**98**	**112**	**126**	**140**	**154**	**168**	**Illness Visit**	**ET**
Window (days)	-56 to -7	-14±7	-	+1	±1	±2	±3	±3	±3	±7	±7	±7	±7	±7	±7	±7	±7	±7	±7		
**Clinical Procedures/Evaluations**																					
Study comprehension exam	X																				
Informed consent	X																				
Confirmation of identity, age, and residency	X																				
Physical exam [2]	X	X	X	X	X	X	X	X	X	X	X	X	X	X	X	X	X	X	X	X	X
Height	X																				
Weight	X		X																		
Vital signs (temperature, blood pressure, and pulse)	X		X	X	X	X	X	X	X	X	X	X	X	X	X	X	X	X	X	X	X
Medical history [2]	X	X	X	X	X	X	X	X	X	X	X	X	X	X	X	X	X	X	X	X	X
Concomitant medications [2]	X	X	X	X	X	X	X	X	X	X	X	X	X	X	X	X	X	X	X	X	X

**Study Day**		**Screen**	**Enroll**[1]	**0**	**1**	**3**	**7**	**14**	**21**	**28**	**42**	**56**	**70**	**84**	**98**	**112**	**126**	**140**	**154**	**168**	**Illness Visit**	**ET**
Window (days)		-56 to -7	-14±7	-	+1	±1	±2	±3	±3	±3	±7	±7	±7	±7	±7	±7	±7	±7	±7	±7		
Artemether-lumefantrine administration			X[3]																			
ECG		X																				
Randomization				X																		
Study agent administration				X[4]																		
Adverse events			X	X	X	X	X	X	X	X	X	X	X	X	X	X	X	X	X	X		X
Pregnancy prevention counseling		X	X	X	X	X	X	X	X	X	X	X	X	X	X	X	X	X	X	X		X
**Laboratory Evaluations**																						
Test	Tube	(Blood volume in mL)																				
Urinalysis	-	X																				
Pregnancy test (urine/serum; for women)	-	X	X	X[5]						X		X		X		X		X		X		
HIV, HBV, HCV screen[6]	SST	3																				
CBC with differential	EDTA	3	3	3[5]		3	3	3														
Hemoglobin type	EDTA	(X)[7]																				
ALT, Cr	SST	3	3	3[5]		3	3	3														
Blood smear and dried blood spot for Pf RT-PCR[8]	-		X	X		X	X	X	0.5	X	0.5	X	0.5	X	0.5	X	0.5	X	0.5	X	X	
PK studies	SST			8[9]	4		4	4		4		4		4		4		4		4		
Serum storage	SST			8			8			8				8						8		
ADA	SST			(X)			(X)			(X)				(X)						(X)		
PBMC storage	CPT			16				16												8[10]		
**Daily volume (mL)**		**9**	**6**	**38**	**4**	**6**	**18**	**26**	**0.5**	**12**	**0.5**	**4**	**0.5**	**12**	**0.5**	**4**	**0.5**	**4**	**0.5**	**20**		
**Cumulative volume (mL)**		**9**	**15**	**53**	**57**	**63**	**81**	**107**	**107.5**	**119.5**	**120**	**124**	**124.5**	**136.5**	**137**	**141**	**141.5**	**145.5**	**146**	**166**	-	-

Study Day	Screen	Enroll[1]	0	1	3	7	14	21	28	42	56	70	84	98	112	126	140	154	168	Illness Visit	ET
Window (days)	-56 to -7	-14±7	-	+1	±1	±2	±3	±3	±3	±7	±7	±7	±7	±7	±7	±7	±7	±7	±7		

Abbreviations: ADA, anti-drug antibodies; ALT, alanine transaminase; CBC, complete blood count; CPT, cell preparation tube; Cr, creatinine; ECG, electrocardiogram; EDTA, ethylenediaminetetraacetic acid; ELISA, enzyme-linked immunosorbent assay; ET, early termination; HBV, hepatitis B virus; HCV, hepatitis C virus; HIV, human immunodeficiency virus; PBMC, peripheral blood mononuclear cell; Pf, *Plasmodium falciparum*; PK, pharmacokinetics; SST, serum-separating tube; RDT, rapid diagnostic test; RT-PCR, reverse transcription polymerase chain reaction.

(X) indicates that no additional blood will be drawn; the test will be performed from the tube shown in the preceding row.

Notes:

- Screening, day 0, and day 7 visits will take place at Kalifabougou for all subjects, regardless of residence. All other visits can take place at either site.
- At any time during the study, the subject may have an unscheduled illness visit if experiencing malaria symptoms or other symptoms. The subject may be referred for standard care according to local guidelines. Unscheduled illness visits will take place at Kalifabougou or Torodo, depending on the subject's residence.

Footnotes:

[1] If enrollment is within 2 days of screening, duplicate procedures will not be repeated.

[2] Complete/comprehensive at screening; targeted/interim at other visits.

[3] Enrollment is defined as the time of first artemether-lumefantrine administration. (For women, negative pregnancy test must be confirmed prior to administration/enrollment.) The first dose will be directly observed in the clinic. The subsequent 5 doses given over 3 days will be observed by guides in the subject's home or at the clinic. All artemether-lumefantrine doses will be completed prior to day 0.

[4] All other study procedures must be completed prior to study agent infusion. Subject will be monitored during and for 60 minutes after each infusion, and vital signs will be recorded directly and 1 hour after infusion. Prior to discharge, subject will be assessed for local reactogenicity (including pain/tenderness, swelling, redness, bruising, and pruritus at the site of infusion) and systemic reactogenicity (including fever, feeling unusually tired or unwell, muscle aches, headache, chills, nausea, and joint pain). If the subject is assessed as being unwell or has ongoing reactogenicity symptoms, he or she will be asked to remain in the clinic until evaluation and discharge by a study clinician. Clinicians will follow any solicited symptoms that are ongoing until they have resolved.

[5] Collected prior to study agent infusion. For women, negative pregnancy test must be confirmed prior to infusion. Other indicated tests may be performed after infusion.

[6] Viral screenings will be performed according to international guidelines. HIV testing will be 2 rapid diagnostic tests (RDTs), plus enzyme-linked immunosorbent assay (ELISA) if the RDTs are discordant; subject will be referred for medical care for 2 positive RDTs or a positive ELISA. Pre- and post-test HIV counseling will be provided. Hepatitis testing will be an HBV surface antigen test (ELISA) and HCV test (ELISA, PCR if indicated). A subject who is HBV and/or HCV positive will be referred for care regardless of the ALT result.

[7] EDTA tube will be stored at screening, and hemoglobin typing will be performed if the subject is enrolled; this test will not be used in eligibility assessments.

[8] If positive for malaria parasite infection, parasite genotyping may be performed. Sample can be obtained via finger prick or venipuncture.

[9] For PK, 4 mL serum will be collected prior to randomization and study agent infusion, and an additional 4 mL serum will be collected about 1 hour (±30 minutes) after the infusion.

[10] This PBMC tube is optional and the subject will have the option to decline.

2 INTRODUCTION

2.1 Study Rationale

Malaria is a mosquito-borne protozoan disease belonging to the genus *Plasmodium* that affects approximately 219 million people and kills approximately 435,000 individuals annually, with an enormous economic impact in the developing world, especially sub-Saharan Africa.[1] The 5 recognized species of *Plasmodium* that cause human malaria infection are Pf, *P. vivax, P. ovale, P. malariae,* and *P. knowlesi*. Among these, Pf causes more deaths in children worldwide than any other single infectious agent. An estimated 30,000 travelers from North America, Europe, and Japan contract malaria per year. Although malaria is preventable with chemoprophylaxis and completely curable with early intervention, drug treatment is not readily accessible in many parts of the world. Additionally, the use of antimalarial drugs over time has been associated with the emergence of drug-resistant strains. Lack of compliance with preventive drug treatment by individuals travelling to endemic areas may also result in fatal malaria infection. The world's first malaria vaccine, RTS,S/AS01 (Mosquirix™), a recombinant protein-based vaccine targeting Pf, was approved for use by European regulatory authorities in 2015. It is currently being evaluated in immunization programs in sub-Saharan Africa despite having been found to provide only partial protection against malaria to children and infants.[2,3] Therefore, the development of safe and effective malaria vaccines or protective antibodies for complete prevention and ultimate elimination of malaria remains an urgent unmet medical need with the potential to have a major impact on improving public health worldwide.

The VRC, NIAID, NIH, has developed a monoclonal antibody (MAb) called CIS43LS as a possible preventive therapeutic against Pf infection. CIS43LS is currently being evaluated in an ongoing phase 1, first-in-human study in healthy volunteers at the VRC. As a follow-up to the VRC study, this protocol is designed to evaluate the safety and efficacy of CIS43LS in healthy adults in Mali, where Pf malaria infection is endemic.

2.2 Background

2.2.1 Overview

The VRC, NIAID, has been investigating broadly-neutralizing human MAbs targeting a variety of pathogens that may be utilized in clinical applications including preventive and therapeutic measures. In the case of malaria, prevention by passive immunization has potential applications for use in travelers, military personnel, season control, and elimination campaigns in endemic areas.

VRC investigators isolated a MAb, termed CIS43, from a subject immunized with an attenuated Pf whole-sporozoite (SPZ) vaccine (PfSPZ Vaccine [Sanaria]). The MAb was then adapted using site-directed mutagenesis in the Fc region, resulting in an LS mutation (CIS43LS) that has been found to increase product half-life in plasma,[4,5] which will be critical for optimizing its use. This LS modification has been applied to multiple VRC clinical MAbs and has been shown to be safe and significantly increase the half-life in plasma.[6, and unpublished data]

CIS43LS recognizes a unique and conserved epitope at the junction of the N- and repeat regions of the Pf circumsporozoite protein (CSP). In a published study in 2 different mouse models of

malaria infection, passive immunization with CIS43 conferred high-level protection (70%-100%) following malaria challenge with concentrations of antibody *in vivo* ranging between 10-200 µg/mL respectively.[7] Similar protection data were observed with CIS43LS (unpublished). These data show that CIS43LS is highly protective *in vivo* and suggest further exploration was warranted for passive prevention of malaria in humans.

This protocol is a two-part phase 2 trial evaluating the safety and tolerability of one-time administration of CIS43LS in healthy Malian adults as well as its protective efficacy against naturally occurring Pf infection over a 6-month malaria season.

2.2.2 Study Product: CIS43LS

MAb VRC-MALMAB0100-00-AB (CIS43LS) was discovered and developed by the VRC, NIAID, NIH. CIS43LS binds a unique and conserved epitope at the junction of the N- and repeat regions of the Pf CSP. The study product was manufactured under current Good Manufacturing Practice (GMP) by the Vaccine Clinical Materials Program (VCMP) operated under contract by Leidos Biomedical Research, Inc., Frederick, MD.

2.2.2.1 Previous Human Experience

VRC 612: A Phase 1, Dose Escalation, Open-Label Clinical Trial With Experimental Controlled Human Malaria Infections (CHMI) to Evaluate Safety and Protective Efficacy of an Anti-Malaria Human Monoclonal Antibody, VRC-MALMAB0100-00-AB (CIS43LS), in Healthy, Malaria-Naive Adults (NCT04206332) has completed controlled human malarial infection (CHMI) protective efficacy testing and remains in long-term follow-up at the VRC. The study is a two-part, dose-escalation, adaptive design study evaluating the safety, tolerability, PK, and protective efficacy of CIS43LS. Doses being evaluated range from 5 mg/kg subcutaneously (SC) to 40 mg/kg IV. The VRC study involves assessing protective efficacy by CHMI. All data from VRC 612 is interim and preliminary with final monitoring and datalock yet to occur.

To date, 25 subjects have received 29 administrations of CIS43LS IV or SC, and there have been no SAEs attributable to CIS43LS or safety pauses. All IV infusions were well-tolerated. Of the 12 subjects who received the highest dose, 40 mg/kg IV, 2 reported mild infusion-site pain or tenderness and 1 reported moderate infusion-site bruising. All systemic symptoms (headache, fever, malaise, joint pain, and muscle aches) were mild to moderate and self-limited. Of the 21 subjects enrolled in Part A, the first part of the adaptive design study, 18 were scheduled to undergo CHMI on March 17, 2020, but the CHMI was cancelled due to the coronavirus disease 2019 (COVID-19) pandemic. The additional 3 subjects that received CIS43LS IV were planned to be followed without CHMI to assess safety in the absence of CHMI. Safety follow-up was maintained for all 21 subjects up to August 17, 2020 following local public health guidelines and practices.

Part B of the trial included Part A veteran subjects as well as newly enrolled subjects. To continue with Part B of this study, including CHMI, the protocol was amended to include the following 3 components: follow subjects after a single administration of CIS43LS in Part A, boost subjects who had previously received CIS43LS in Part A, and enroll new subjects for

CIS43LS administration and/or CHMI. Part B of the trial opened to accrual in September 2020 and included 16 subjects as follows:

- 2 subjects who received 40 mg/kg IV in Part A received no additional product in Part B.
- 2 subjects who received 5 mg/kg (1 SC and 1 IV) in Part A were boosted with 20 mg/kg IV in Part B.
- 2 subjects who received 20 mg/kg IV in Part A were boosted with 20 mg/kg IV in Part B.
- 4 new subjects received 40 mg/kg IV in Part B.
- 6 CHMI control subjects who had never received CIS43LS did not receive CIS43LS in Part B.

A total of 15 subjects underwent CHMI on October 20, 2020 and were followed for 21 days with daily polymerase chain reaction (PCR) from days 7-18. One subject who received 40 mg/kg IV in September 2020 did not undergo CHMI because of a concomitant, unrelated SAE that was unlikely to resolve by the time of challenge. Any subjects who were PCR-positive from day 7-18 were treated with Malarone. On day 21, all remaining untreated subjects were treated with Malarone. Five of the 6 control subjects were PCR-positive by day 9 post-CHMI and were treated. The 9 CIS43LS recipients never developed parasitemia nor did they require treatment. Two of these subjects had received CIS43LS (40 mg/kg IV) approximately 9 months in Part A with no additional product in Part B prior to CHMI. The *in vivo* concentration of CIS43LS at the time of CHMI in the 9 protected subjects in the phase 1 VRC study ranged from 50-500 μg/mL. Thus, in this small phase 1 trial, CIS43LS was completely protective across a range of *in vivo* concentrations and provided durable protection.

Prior clinical experience in completed and ongoing phase 1 and 2 trials of healthy adults with other human MAbs manufactured and formulated by the VRC that recognize pathogen-specific epitopes (i.e., VRC01, VRC01LS, VRC07-523LS, and MAb114)[6,8-10] are used to summarize the general safety risk associated with MAbs.

Treatments with these MAbs have been generally well tolerated, with no reported deaths or serious adverse events (SAEs) assessed as related to the study products. Typical for MAbs, the predominant local reactogenicity complaint has been mild pain/tenderness, although reports of mild injection-site pruritus, redness and swelling have occurred at modestly higher frequencies with SC administration. Malaise, muscle pain, and headache have been the most frequently reported solicited complaints noted in the 3 days after product administration, and these have also been mostly transient and mild in severity. Urticaria and infusion reactions including chills, rigors, myalgia, headache, and/or fever have been reported after IV infusions at product doses of 10 to 40 mg/kg; these reactions have been transient, resolved without sequelae within 24 hours of onset, and were treated with over-the-counter analgesics and antipyretics.

2.2.2.2 Preclinical Experience

To assess CIS43LS as a candidate for clinical trials, recombinant, research grade MAb was evaluated on binding properties, auto-reactivity, PK, 2 mouse models of *in vivo* protection following challenge, and PK studies in nonhuman primates. In mice, CIS43LS conferred 100% protection following mosquito bite challenge. In nonhuman primate PK studies, CIS43LS exhibited substantially longer half-life, both in blood and in skin biopsy samples as compared to CIS43 antibody without the LS mutation.

Preclinical toxicology studies were conducted to assess safety in compliance with current Good Laboratory Practices (GLP) in Sprague Dawley rats using CIS43LS. The toxicology pre-clinical material was tested in the final formulation buffer and manufactured using the same VCMP manufacturing process as used to produce clinical trial material. There were no findings of toxicologic significance attributed to CIS43LS from the results.

2.2.2.2.1 *In vitro* Studies

Differences in the binding properties of CIS43LS and CIS43 were assessed. Binding of CIS43LS and CIS43 to a specific target antigen expressed on malaria parasites was determined by enzyme-linked immunosorbent assay (ELISA). CIS43LS and CIS43 exhibited similar binding to recombinant Pf circumsporozoite protein (rPfCSP). Avidity and stoichiometry of CIS43LS and CIS43 binding to rPfCSP were also comparable as assessed by isothermal titration calorimetry.

CIS43LS autoreactivity was assessed using the anti-nuclear antibody (ANA) HEp2 cell staining system from Zeus Scientific (Branchburg, NJ). The assay was conducted according to the manufacturer's instructions. Antibodies were tested at 25 and 50 mcg/mL. At 25 mcg/mL, test antibodies were scored in comparison to negative and positive control samples. Neither CIS43 nor CIS43LS shows an autoreactivity signal by ANA staining. Autoreactivity was also assessed by an anti-cardiolipin QUANTA Lite ACA immunoglobulin (Ig) GIII ELISA (Inova Diagnostics, San Diego, CA). Using a similar control schema to that described for ANA HEp2 cell staining, the assay was conducted according to the manufacturer's instructions.

The potential for CIS43LS, a MAb directed against Pf CSP on the surface of PfSPZ, to cross-react with normal human and Sprague-Dawley rat tissues was assessed in a GLP study (Charles River Laboratories, Inc., Frederick, MD, Study No. 20188989) by immunohistochemical staining of tissue cryosections with CIS43LS or appropriate controls. The human and rat tissue panels evaluated included all of the tissues on the "suggested list of human tissues to be used for immunohistochemical or cytochemical investigations of cross reactivity of monoclonal antibodies" in Annex I of the European Medicines Agency document "Guideline on Development, Production, Characterization and Specifications for Monoclonal Antibodies and Related Products," adopted by the Committee for Medicinal Products for Human Use, and all of the tissues recommended in the US Food and Drug Administration (FDA) Center for Biologics Evaluation and Research document, "Points to Consider in the Manufacture and Testing of Monoclonal Antibody Products for Human Use." The only difference between the 2 species in tissue types evaluated was tonsil; inasmuch as rats do not have tonsils, tonsil tissue was evaluated from humans only. To detect binding, CIS43LS was applied to cryosections of normal human tissues from 3 donors per human tissue, except for bladder, for which only 2 were available. Samples from at least 2 individual rats were evaluated except for fallopian tube, parathyroid, and ureter, for which only 1 was available. All tissues were incubated with test or control article at 2 concentrations (1 µg/mL and 10 µg/mL). As a negative control, a monoclonal human $IgG1_{\kappa}$ antibody, HuIgG1 (control article), with a different antigenic specificity from that of CIS43LS, was tested in parallel. Other controls included omission of the test or control articles from the assay (assay controls). At both concentrations, CIS43LS produced moderate to intense membrane and cytoplasmic staining of positive control HEK293 cells, which expressed the Pf CSP epitope targeted by CIS43LS, and did not specifically react with the negative control material, cryosections of negative HEK293 cells, at either staining concentration. The control

article, HuIgG1, did not specifically react with either the positive or negative control materials, and there was similarly no staining of the assay control slides. The specific reactions of CIS43LS with the positive control cells in all staining runs, the absence of specific reactivity with the negative control cells, and the lack of reactivity of the control article indicated that the assay was sensitive, specific, and reproducible. CIS43LS produced membrane and cytoplasmic staining of epithelium in sebaceous glands and cytoplasmic staining of epithelium in sweat glands in the human skin. This staining was weak to strong in intensity and frequently observed in sebaceous glands and rarely or occasionally observed in sweat glands at the higher concentration of CIS43LS, with a reduction in the intensity of the staining at the lower concentration. No other staining was observed with CIS43LS in the human tissue panel. Similarly to its interaction with human tissue, CIS43LS weakly stained the membrane and cytoplasm of occasional to frequent sebaceous gland epithelium in the rat skin at the higher concentration only. No other staining was observed with CIS43LS in the rat tissue panel. The consonance of results between human and rat tissues confirm the appropriateness of the rat model for the CIS43LS toxicity study.

2.2.2.2.2 *In vivo* Studies

2.2.2.2.2.1 Protection studies in C57BL/6 mice and Rhesus macaques

To assess protection following passive transfer of MAb, naïve C57BL/6 mice received 300 mcg by IV inoculation of CIS43, CIS43LS, or negative control antibody. Chimeric parasites comprising *Plasmodium berghei* (Pb) SPZ which naturally infect mice that expressed Pf CSP (Pb-PfCSP SPZ) were administered by 1 of 2 routes (injected by tail vein or by exposure to bites by infected mosquitos). In the 2 mouse challenge models, passive transfer of CIS43 and CIS43LS yielded nearly indistinguishable results. Administration of either CIS43 or CIS43LS reduced liver parasite burden by about 2 logs when challenged by IV route. Moreover, passive transfer of CIS43 or CIS43LS conferred 100% sterile protection in mice as assessed by parasitemia in blood following a natural challenge by 5 mosquito bites in the skin. This type of challenge is similar to what is used in the CHMI procedures at the VRC.

To inform clinical protocol development, particularly concerning the timing of CHMI, a 20-week PK study was conducted in nonhuman primates (Rhesus macaques). Two animals per group received CIS43 or CIS43LS, administered by IV route at a dose of 10 mg/kg. CIS43LS exhibited substantially longer half-life, both in blood and skin biopsy samples than CIS43. In blood, CIS43LS maintained a titer of more than 35 mcg/mL on the last sample day (day 140). In contrast, CIS43 dropped to approximately 30 mcg/mL by day 49. Skin biopsy data were concordant.

2.2.2.2.2.2 Toxicology Studies in Sprague Dawley Rats

A study of repeat dosing at 10-day intervals by IV and SC administration of CIS43LS in male and female Sprague Dawley rats (SRI Study No. M416-19) was conducted in compliance with current GLP to evaluate toxicity and toxicokinetics. The test article and control (formulation buffer) were administered on days 1 and 11 by IV administration at doses of 0, 40, or 400 mg/kg/day (Groups 1−3, respectively) or by SC administration at doses of 5 or 50 mg/kg/day (Groups 4−5, respectively). Necropsies were performed on days 12 and 46. Endpoints

included clinical observations, body weight, food consumption, body temperature, dose site irritation, clinical pathology, and organ weights.

All animals survived to their scheduled sacrifice timepoint. No test article-related changes were observed in clinical observations, body weights, food consumption, dose site irritation, clinical chemistry, coagulation, and organ weights. Body temperature was normal (≤101.0°F) for all males at 24 hours post-dose. Elevated body temperatures ranging from 101.1 to 102.1°F were seen from 1 or more females in high-dose IV or both SC treated groups for at least 24 hours after dosing on days 1 and/or 11. Body temperature returned to normal levels within 48 hours post-dose. These findings are considered test article-related although reversible and at levels considered to be non-adverse.

Statistically significant and dose-dependent changes in some hematology parameters were observed on days 12 and 46. The changes included increased platelet count (1.4-fold) and absolute reticulocyte count (1.2-fold) in Group 5 females, and decreased percent monocyte (30.6% to 32.2%) in Group 3 and 4 females on day 12. On day 46, increased absolute neutrophils (2.0-to 2.3-fold) in Group 3 and 4 males and decreased hemoglobin (6.1% to 7.6%) and/or hematocrit (5.6% to 7.6%) in all treated-group females were observed. The changes in neutrophils and monocytes seen after SC administration are consistent with an immune response and are considered test article-related although reversible and at levels considered to be non-adverse. Without changes in red blood cell counts, the findings in platelet count, reticulocytes, hemoglobin, or hematocrit are not considered to be biologically meaningful or toxicologically adverse. In summary, no findings of toxicologic significance were attributed to CIS43LS from the results available as of this report.

Taken together, these studies support the continued development of CIS43LS for clinical evaluation. The data confirm the predicted longer half-life effect of the LS mutation and show that there is no functional difference in terms of *in vitro* binding or *in vivo* protection compared to CIS43. No safety, efficacy, or manufacturability concerns were noted that precluded proceeding to further toxicology studies, GMP manufacturing, IND filing, and clinical investigations.

2.2.3 Laboratory Assessments of CIS43LS

Some laboratory assessments in this study are designed to characterize the investigational product. This includes PK analysis and evaluation for anti-drug antibody (ADA) development after product exposure. Other assays may also be completed from stored samples at a later date.

The LIG International Center of Excellence in Research Lab at the USTTB in Bamako will process blood and store coded samples and will either perform sample testing or ship coded samples to designated research laboratories at LIG/NIAID and VRC/NIAID or other approved collaborators. The key to the code will remain at the USTTB. See section 1.3 for schedules, blood volumes, and tube types to be used for research sample collection. Research assays will be performed on samples from both study product recipients and placebo controls at baseline and throughout the study.

Tube types for clinical labs are according to institutional requirements and are shown in section 1.3 to estimate blood volumes. Different tubes for clinical evaluations may be used to meet site requirements. Research sample tube types and blood volumes must be used as shown or

as otherwise instructed by the manufacturer. In some instances, coded samples may be transported directly by study staff to the laboratory of an approved collaborator.

2.2.3.1 Pharmacokinetic Analysis

Concentrations of CIS43LS will be measured by a Meso Scale Discovery LLC–based automation platform and similar methodology as previously described for other VRC MAb products.[8]

2.2.3.2 Detection of Anti-Drug Antibodies

Assays for detection of ADA will be performed at specified timepoints following product administration and compared to baseline status using a similar methodology as previously described for other VRC MAb products.[8]

2.2.3.3 Measures of MAb-Mediated Protection and Parasitemia

CIS43LS-mediated protection against naturally occurring Pf infection during a single 6-month malaria season will be assessed and compared to control subjects. The endpoint defining MAb-mediated protection from Pf infection is the absence of Pf parasites in blood samples obtained from CIS43LS-recipients collected from day 7 through week 24 (day 168) after study agent administration. The criteria for a case of Pf infection is based on blood smear analysis.

Giemsa-stained thick blood films will be prepared and examined by trained personnel following the standard operating procedure (SOP) based on the standard World Health Organization (WHO) protocol.

Thick blood smears will be prepared from the blood remaining in the collection device, or (at timepoints when no blood collection is planned) from a finger prick sample or from a small venipuncture sample. The smears will be examined microscopically. Thick blood smears will be used for diagnosis throughout the study.

Additionally, as a secondary endpoint, *Plasmodium* 18S rRNA RT-PCR will be performed by Dr. Sean Murphy, University of Washington. The assay will be applied to dried blood spots using methods substantially equivalent to those reviewed extensively by the FDA for Biomarker Qualification of the 18S rRNA by the University of Washington for CHMI trials.[11] The assay has been validated for use on dried blood spots.

Research blood samples may also be used for Pf malaria parasite genome analysis that will be conducted by Dr. Daniel Neafsey, Harvard School of Public Health.

2.3 RISK/BENEFIT ASSESSMENT

2.3.1 Known Potential Risks

Risk of CIS43LS: CIS43LS is being evaluated in an ongoing phase 1 study in healthy adult volunteers at the VRC to assess safety, tolerability, PK, and protective efficacy, as described in section 2.2.2.1. Since January 2020, 25 subjects have received CIS43LS either IV or SC, and there have been no SAEs assessed as related to CIS43LS and no safety pauses. All systemic symptoms were only mild to moderate and self-limited. Mild or moderate headache was reported

by 6 subjects (29%). Other solicited systemic AEs include mild myalgia (5 subjects), moderate malaise (1), moderate nausea (1), mild joint pain (1), and mild fever (1). There were also 3 related unsolicited AEs that were reported (blood creatinine increase, neutropenia and dizziness), all of which were graded as mild. All IV infusions were well-tolerated. Of the 12 subjects who received 40 mg/kg IV, 2 reported mild infusion-site pain or tenderness and 1 reported moderate infusion-site bruising. The protocol and consent will be updated if new information about risks and side effects becomes available from the ongoing phase 1 study.

Risks of MAb Administration: Administration of MAbs may cause immune reactions such as acute anaphylaxis, serum sickness, and the generation of antibodies. However, these reactions are rare and more often associated with MAbs targeted to human proteins or with the use of mouse MAbs that would have a risk of human anti-mouse antibodies.[12] In this regard, because CIS43LS is targeted to a parasite antigen and is a human MAb, it is expected to have a low risk of such side effects.

Typically, the side effects of MAbs are mild to moderate and may include local reactions at the injection site (including pain, redness, bruising, swelling, pruritis) and systemic reactions such as fever, chills, rigors, nausea, vomiting, pain, headache, myalgia, arthralgia, dizziness, fatigue, shortness of breath, bronchospasm, hypotension, hypertension, pruritus, rash, urticaria, angioedema, diarrhea, tachycardia, or chest pain. Healthcare staff will be appropriately trained and necessary medical equipment will be readily available at the clinic where the study agent is administered. Clinical use of MAbs that are targeted to cytokines or antigens associated with human cells may be associated with an increased risk of infections[12]; however, this is not expected to be a risk for a MAb targeted to a parasite antigen.

Severe reactions, such as anaphylaxis, angioedema, bronchospasm, hypotension, and hypoxia, are infrequent and more often associated with MAbs targeted to human proteins or with non-human MAbs, such as a mouse MAb.[12] Most infusion-related events occur within the first 24 hours after initiation of MAb administration.

Published experience with human MAbs directed against cell surface targets on lymphocytes shows that infusion of a MAb may be associated with cytokine release, causing a reaction known as cytokine release syndrome (CRS).[13] CRS reactions commonly occur within the first few hours of infusion start and with the first MAb infusion received. This is because the cytokine release is associated with lysis of the cells targeted by the MAb and the burden of target cells is greatest at the time of the first MAb treatment. With licensed therapeutic MAbs, CRS is managed by temporarily stopping the infusion, administering histamine blockers and restarting the infusion at a slower rate.[14] Supportive treatment may also be indicated for some signs and symptoms.

Delayed allergic reactions that include a serum sickness type of reaction characterized by urticaria, fever, lymph node enlargement, and joint pains, typically occur several days after MAb exposure and are more commonly associated with chimeric types of MAbs.[12] In general, and with due consideration of the needs dictated by individual subject symptoms and treating clinician discretion, immediate and delayed reactions to study product will be managed according to the principles of the American Academy of Allergy, Asthma, and Immunology guidelines.[15]

Participation in this study may limit a subject's eligibility for future MAb studies.

Risks of Placebo Administration: There are no risks of the placebo (normal saline) other than infusion-related events such as headache, dizziness, and hypertension.

Risks of Blood Drawing or IV Placement: Drawing blood via venipuncture or heel/finger stick or placing an IV may cause pain, bruising, and a feeling of lightheadedness or fainting. Rarely, it may cause infection at the site where blood is taken.

Risks of Artemether-Lumefantrine Administration: Adverse reactions (ARs) occurring in more than 30% of adults are headache, anorexia, dizziness, asthenia, arthralgia, and myalgia. Reactions typically do not require stopping treatment. Individuals who may have any contraindication for the use of this drug (e.g., prolonged QTc) will be excluded at screening. In postmarketing experience, serious hypersensitivity reactions including anaphylaxis and serious skin reactions (bullous eruption) have been reported. Individuals with known sensitivity or contraindications to the antimalarials administered in this study are excluded from participation.

A complete list of side effects and contraindications is provided in the package insert.[16]

2.3.2 Known Potential Benefits

Subjects in the dose-escalation study will not directly benefit from study participation, since the study is too early in the year to be expected to provide protection during the malaria transmission season. In the efficacy study, subjects may not receive direct health benefit from study participation. Depending on whether CIS43LS confers protective efficacy, subjects receiving a sufficient dose of CIS43LS may experience some protection against Pf infection during part or all of the malaria transmission season.

In the future, others may benefit from knowledge gained in this study that may aid in the development of malaria prevention.

2.3.3 Assessment of Potential Risks and Benefits

The study population lives in an area where malaria is endemic and so is at significant risk of malarial infection and disease. As described above, it is possible that some subjects may benefit from study participation by receiving some protection during the malaria season. Therefore, the value of the information that will be gained from this study for developing malaria prevention strategies justifies the potential risks of study participation described above. Additionally, potential risks are minimized by careful design of subject eligibility criteria and post-vaccination monitoring.

3 OBJECTIVES AND ENDPOINTS

OBJECTIVES	ENDPOINTS	JUSTIFICATION FOR ENDPOINTS
Primary		
1. Dose escalation: To evaluate the safety and tolerability of CIS43LS administered IV at 5,	1. Dose escalation: Incidence and severity of local and systemic AEs occurring within 7 days after the administration of CIS43LS.	1. Assessment of AEs is a standard measure of study

OBJECTIVES	ENDPOINTS	JUSTIFICATION FOR ENDPOINTS
10, and 40 mg/kg in healthy Malian adults.<br>2. Efficacy: To determine if IV administration of CIS43LS at 10 mg/kg and 40 mg/kg (compared to placebo) mediates protection against naturally occurring Pf infection in healthy Malian adults during a single malaria season as detected from microscopic examination of thick blood smear.	2. Efficacy: Pf blood stage infection as detected by microscopic examination of thick blood smear for 24 weeks after administration of CIS43LS or placebo.	agent safety and tolerability.<br>2. Blood smear is the gold standard for diagnosis of blood-stage Pf infection.
Secondary		
1. Dose escalation: To evaluate the PK of CIS43LS at each dose level throughout the study.<br>2. Efficacy: To determine if IV administration of CIS43LS at 10 mg/kg and 40 mg/kg (compared to placebo) mediates protection against naturally occurring Pf infection in healthy Malian adults during a single malaria season as detected by RT-PCR.<br>3. Efficacy: To evaluate the PK of CIS43LS throughout the study at the dose of 10 mg/kg and 40 mg/kg and to correlate CIS43LS serum concentration with Pf infection risk.	1. Dose escalation: Measurement of CIS43LS in sera of recipients.<br>2. Efficacy: Pf blood stage infection as detected by RT-PCR for 24 weeks after administration of CIS43LS or placebo.<br>3. Efficacy: Measurement of CIS43LS in sera of recipients.	1. Concentrations of CIS43LS in blood will help assess durability of CIS43LS at each dose level.<br>2. RT-PCR is more sensitive than blood smear for detecting Pf blood-stage infection.<br>3. Concentrations of CIS43LS in blood will help assess durability of CIS43LS and will allow for correlation with Pf infection risk.
Tertiary/Exploratory		
1. To determine whether ADA to CIS43LS can be detected in sera of recipients at specific timepoints throughout the study.<br>2. To assess for IgG1 allotypes and allotype-specific effects on CIS43LS PK.	1. Measurement of ADA to CIS43LS in sera of recipients.<br>2. Assessment of IgG1 allotypes and allotype-specific effects on CIS43LS PK.<br>3. Characterization of the cellular immune response to CIS43LS.	1. ADA to CIS43LS may impact the PK and activity of CIS43LS.<br>2. Subject IgG1 allotype may impact the PK and activity of CIS43LS.

OBJECTIVES	ENDPOINTS	JUSTIFICATION FOR ENDPOINTS
3. To explore and characterize the cellular immune response to CIS43LS.<br>4. To determine if the efficacy of CIS43LS is specific to certain Pf parasite genotypes at the CSP locus.<br>5. To explore the impact of pre-existing parasitemia on the protective efficacy and PK of CIS43LS.<br>6. To explore the impact of pre-existing CSP antibodies on the protective efficacy and PK of CIS43LS.	4. CSP genotyping of parasites isolated from study subjects.<br>5. Pre-existing parasitemia detected by microscopic examination of thick blood smears or RT-PCR before CIS43LS infusion.<br>6. Pre-existing CSP-specific antibodies measured in sera collected before CIS43LS infusion.	3. CIS43LS-induced cellular immune responses may be associated with Pf infection risk.<br>4. CIS43LS efficacy may be specific to certain Pf parasite genotypes at the CSP locus.<br>5. Pre-existing parasitemia may impact the protective efficacy and PK of CIS43LS.<br>6. Pre-existing CSP-specific antibodies may impact the protective efficacy and PK of CIS43LS.

4 STUDY DESIGN

4.1 OVERALL DESIGN

This is a two-part, phase 2 trial evaluating the safety and tolerability of one-time administration of CIS43LS, as well as its protective efficacy against naturally occurring Pf infection over a 6-month malaria season. Interim safety data from the ongoing VRC phase 1 trial of CIS43LS will be regularly reviewed, and if needed, the protocol and relevant study documents will be amended to reflect any new information.

The primary study hypotheses are that CIS43LS will be safe and will confer protection against Pf infection. The study will recruit from 2 MRTC clinics, 1 in Torodo and 1 in Kalifabougou. All of the screening, day 0 and day 7 visits will take place at Kalifabougou for all subjects, regardless of residence.

The rural village of Kalifabougou is situated 46 km from the MRTC laboratory in Bamako where biological samples collected for this protocol will be processed and stored. Torodo is located 12 km north of Kalifabougou. The economy is based on subsistence farming. Kalifabougou and Torodo are similar in terms of geographic, demographic, and epidemiological characteristics, and

both typically experience intense seasonal Pf transmission from July through December each year.[17] Based on data collected in Kalifabougou from 2011 through 2018, 68%-90% of adults who are uninfected (PCR negative) before the malaria season become infected with Pf during the ensuing 6-month malaria season.[17]

The dose-escalation study is an open-label study for safety and tolerability. Subjects will be assigned to 1 of 3 dose arms. Dosing will begin in the lowest dose arm and will escalate sequentially as described in section 6.1.2.1. Subjects will be followed for safety to assess AEs at study visits 1, 3, 7, 14, 21, and 28 days after administration, then monthly for about 6 months after administration. Participants who complete participation in the dose-escalation will be offered participation in extension visits to collect extra bi-weekly blood samples for PK analysis and to monitor for Pf infection through the end of December 2021.

After the final subject in the dose-escalation study reaches day 7 safety follow-up, an interim safety evaluation will be performed by the DSMB before enrollment begins for the efficacy study (section 9.4.8).

The efficacy study is a randomized, double-blind, placebo-controlled trial (N=330 total, n=110 for each of 3 treatment arms) to assess safety and protective efficacy of 2 dose arms and a placebo arm. Subjects will receive 1 dose of study agent and be followed at regular study visits for about 6 months. Primary study assessments include physical examination and blood collection for research laboratory evaluations, including assessment of protection against naturally occurring Pf infection over 6 months (a single malaria season). The primary endpoint is the absence of Pf parasites in blood smears obtained between 1 week and 6 months after study agent administration in recipients of CIS43LS and placebo.

4.2 Scientific Rationale for Study Design

This two-part study was designed to test CIS43LS in the setting of naturally occurring Pf infection and in a population that could potentially benefit from a novel therapeutic for malaria prevention.

The efficacy study will use both randomization and a double blind to minimize bias in subject selection and study assessments. The placebo will be inactive (normal saline) rather than a comparator MAb, as currently there are no licensed anti-malaria MAbs available.

4.3 Justification for Dose

The doses used in this study were selected based off preclinical data and will complement the data generated in the ongoing phase 1 VRC study, which tested the dose range included in this protocol. The doses used in the efficacy study (10 mg/kg and 40 mg/kg IV) will complement the data generated in the ongoing phase 1 VRC study. The 40-mg/kg dose was derived from 1) efficacy data from the challenge studies performed in mice showing that the protective concentration of antibody *in vivo* is between 10-200 µg/mL in 2 different mouse models of malaria infection; and 2) PK data from nonhuman primate studies with CIS43LS and prior clinical experience in healthy adults with human MAbs targeting HIV (i.e., VRC01, VRC01LS, and VRC07-523LS) and ebolavirus (MAb114) at the same dose. The addition of the 10-mg/kg dose was based on more recent PK data from the phase 1 VRC study. Extrapolating PK data from the 5-mg/kg and 20-mg/kg groups in the phase 1 VRC study, we estimate that 10 mg/kg

will give an *in vivo* concentration of approximately 90 µg/mL at 3 months (mid-malaria season) and approximately 30 µg/mL at 6 months (end of malaria season). The *in vivo* concentration of CIS43LS at the time of CHMI in the 9 protected subjects in the phase 1 VRC study ranged from 50-500 µg/mL.

5 STUDY POPULATION

5.1 INCLUSION CRITERIA

Individuals must meet all of the following criteria to be eligible for study participation:

1. Aged ≥18 and ≤55 years.
2. Able to provide proof of identity to the satisfaction of the study clinician completing the enrollment process.
3. In good general health and without clinically significant medical history.
4. Able to provide informed consent.
5. Willing to have blood samples and data stored for future research.
6. Resides in or near Kalifabougou or Torodo, Mali, and available for the duration of the study.
7. Females of childbearing potential must be willing to use reliable contraception from 21 days prior to study day 0 through the final study visit as described below.
    a. Reliable methods of birth control include 1 of the following: confirmed pharmacologic contraceptives via parenteral delivery or intrauterine or implantable device.
    b. Nonchildbearing women will be required to report date of last menstrual period, history of surgical sterility (i.e., tubal ligation, hysterectomy) or premature ovarian insufficiency, and will have urine or serum pregnancy test performed per protocol.

5.2 EXCLUSION CRITERIA

Individuals meeting any of the following criteria will be excluded from study participation:

1. Pregnancy, as determined by a positive urine or serum beta-human choriogonadotropin (β-hCG) test (if female).
2. Currently breastfeeding.
3. Behavioral, cognitive, or psychiatric disease that in the opinion of the investigator affects the ability of the subject to understand and comply with the study protocol.
4. Study comprehension examination score of <80% correct or per investigator discretion.
5. Hemoglobin, white blood cell, absolute neutrophil, or platelet count outside the local laboratory–defined limits of normal. (Subjects may be included at the investigator's discretion for "not clinically significant" values.)
6. Alanine transaminase (ALT) or creatinine (Cr) level above the local laboratory–defined upper limit of normal. (Subjects may be included at the investigator's discretion for "not clinically significant" values.)
7. Infected with human immunodeficiency virus (HIV), hepatitis C virus (HCV), or hepatitis B virus (HBV).

8. Known or documented sickle cell disease by history. (Note: Known sickle cell trait is NOT exclusionary.)
9. Clinically significant abnormal electrocardiogram (ECG; corrected QT interval [QTc] >460 or other significant abnormal findings, including unexplained tachycardia or bradycardia).
10. Evidence of clinically significant neurologic, cardiac, pulmonary, hepatic, endocrine, rheumatologic, autoimmune, hematological, oncologic, or renal disease by history, physical examination, and/or laboratory studies including urinalysis.
11. Receipt of any investigational product within the past 30 days.
12. Participation or planned participation in an interventional trial with an investigational product until the last required protocol visit. (Note: Past, current, or planned participation in observational studies is NOT exclusionary.)
13. Medical, occupational, or family problems as a result of alcohol or illicit drug use during the past 12 months.
14. History of a severe allergic reaction or anaphylaxis.
15. Severe asthma (defined as asthma that is unstable or required emergent care, urgent care, hospitalization, or intubation during the past 2 years, or that has required the use of oral or parenteral corticosteroids at any time during the past 2 years).
16. Pre-existing autoimmune or antibody-mediated diseases including but not limited to: systemic lupus erythematosus, rheumatoid arthritis, multiple sclerosis, Sjögren's syndrome, or autoimmune thrombocytopenia.
17. Known immunodeficiency syndrome.
18. Known asplenia or functional asplenia.
19. Use of chronic (≥14 days) oral or IV corticosteroids (excluding topical or nasal) at immunosuppressive doses (i.e., prednisone >10 mg/day) or immunosuppressive drugs within 30 days of day 0.
20. Receipt of a live vaccine within the past 4 weeks or a killed vaccine within the past 2 weeks prior to study agent administration.
21. Receipt of immunoglobulins and/or blood products within the past 6 months.
22. Previous receipt of an investigational malaria vaccine in the last 5 years.
23. Known allergies or contraindication against artemether-lumefantrine.
24. Other condition(s) that, in the opinion of the investigator, would jeopardize the safety or rights of a subject participating in the trial, interfere with the evaluation of the study objectives, or render the subject unable to comply with the protocol.

5.3 INCLUSION OR EXCLUSION OF VULNERABLE PARTICIPANTS

- **Children:** Children are not eligible to participate in this clinical trial because CIS43LS has not been previously evaluated in children to be safe. If the product is assessed as safe for further study, other protocols specifically designed for children may be conducted.
- **Pregnant Women:** Pregnant women are excluded from this study because the effects of CIS43LS on pregnant women or the developing human fetus are unknown with the potential for teratogenic or abortifacient effects.
- **Decisionally Impaired Adults:** Individuals must be able to provide consent in order to be eligible for participation in this study. Enrolled subjects who lose decision-making capability during study participation will be withdrawn (see sections 8.4.3 and 10.1.5).

- **Illiterate Individuals:** We anticipate that many individuals eligible for this study will be illiterate in French, so the study team will translate the consent orally into local languages when appropriate, as described in section 10.1.

5.4 Lifestyle Considerations

In addition to prohibited treatments and procedures listed in section 6.5, subjects must refrain from donating blood for at least 1 year after study drug administration.

Women must not breastfeed during the study because there could be unknown risks to the child secondary to the mother's receipt of the study drug.

5.5 Screen Failures

Screen failures are defined as subjects who consent to participate in the clinical trial but are not subsequently enrolled in the study. A minimal set of screen failure information is required to ensure transparent reporting of screen failure subjects, to meet the Consolidated Standards of Reporting Trials (CONSORT) publishing requirements and to respond to queries from regulatory authorities. Minimal information includes demography, screen failure details, eligibility criteria, and any SAE.

Individuals who initially do not meet the criteria for participation in this trial (screen failure) because of an acute illness or a transient lab or other screening procedure findings (e.g., abnormal ECG, ALT, or other transient lab evaluations) may be rescreened or may repeat individual screening procedures at the investigator's discretion. Rescreened subjects should be assigned the same subject number as for the initial screening.

5.6 Strategies for Recruitment and Retention

Healthy adult volunteers will be selected based on the eligibility criteria described in sections 5.1 and 5.2. The total target sample size across both study sites is 18 subjects in the dose-escalation study (accrual ceiling 30) and 330 subjects in the efficacy study (accrual ceiling 460). We expect to enroll all subjects for both parts of the study within the first 6 months of the study. Efforts will be made to include women in proportions similar to that of the community from which they are recruited. Subject selection will not be limited based on sex, race, or ethnicity.

The study team will hold a community meeting in Kalifabougou and Torodo to explain and discuss the study and obtain community permission from the village elders, heads of families, and other community members in each village where the study will take place (section 10.1.1.1). Afterward, an announcement via local radio or another traditional channel of communication may be made to invite households to come to participating clinics to learn about the study.

5.6.1 Costs

There are no costs associated with participation in this trial.

5.6.2 Compensation

Subjects will be compensated for the time and inconvenience of participating in the study as follows:

- 5,000 Communauté Financière Africaine (CFA) Franc for each of the following: screening visit, enrollment visit and day 0 visit (infusion).
- 3,000 CFA Franc for all other visits.

The total compensation amount for dose-escalation study subjects will be 48,000 CFA Franc (valued at approximately USD $88) and for the efficacy study subjects will be 63,000 CFA Franc (valued at approximately USD $115). Payment will be provided in cash after the completion of each visit. Subjects who participate in extension visits will receive additional compensation as described below (see Appendix C).

Subjects will be provided with transportation to and from study visits but will not receive additional reimbursement for travel.

6 STUDY INTERVENTION

6.1 Study Intervention Administration

6.1.1 Study Intervention Description

The study intervention involves a single administration of CIS43LS or placebo to each subject. All study agent administration will take place at the Kalifabougou study site.

6.1.2 Dosing and Administration

Study agent and dosing will be dependent on the progress of the study and study arm assignment (Table 1). The dose-escalation study dosing plan is described in section 6.1.2.1. Procedures for IV infusion are described in section 6.1.2.4.

Table 1. Study agent assignment and dosing by arm.

Arm	Subjects	Study Agent and Dose
**Dose-escalation study**		
1	6	5 mg/kg IV
2	6	10 mg/kg IV
3	6	40 mg/kg IV
**Efficacy study**		
1	110	10 mg/kg IV
2	110	40 mg/kg IV
3	110	Placebo (normal saline) IV

6.1.2.1 Dose Escalation

In the dose-escalation study, dose escalation will proceed in 3 study arms. Enrollment will begin with the arm receiving the lowest of 3 dose levels of CIS43LS. Subjects enrolled in this arm will

be scheduled for study intervention, with at least 1 hour of observation between study agent administration in each subject, and follow-up procedures. Once all subjects at that dose level complete day 7 post-infusion, if no safety concerns have arisen, then enrollment will begin for the arm at the subsequent dose level. This process will be repeated to evaluate the second dose level prior to commencing with the highest dose level.

6.1.2.2 Dose Limiting Toxicity

Pausing and halting rules are provided in sections 8.4.5 and 8.4.6.

6.1.2.3 Dose Modifications

This study involves administering a single weight-based dose to each subject, so there will be no dose modifications.

6.1.2.4 Drug Administration

Prior to study agent administration on day 0, subjects will undergo vital signs measurement, a targeted physical examination (as needed based on signs, reported symptoms, or interim medical history), and, for women, a urine pregnancy test (confirmed negative prior to administration). Subjects will have IV access placed in an arm vein in an aseptic manner. A different site may be used for collection of PK blood samples; however, the same site may be used after flushing the line if another site is not available.

The study agent will be administered with approximately 100 mL of normal saline IV over about 30-60 minutes, with a target of 30 minutes. Infusions lasting longer than 30 minutes are allowed. The rate of infusion may range from 10-20 mg/kg/hr at the lowest dose level to 80-160 mg/kg/hr at the highest dose level. The mL/hr infusion rate may vary based on the total volume needed to administer a full dose. Vital signs will be monitored directly after infusion, and hourly during monitoring. If the subject experiences side effects during the infusion, the rate of infusion may be slowed or stopped to alleviate the symptoms. At the end of product administration, the IV administration set must be flushed with about 30 mL (or appropriate volume) of normal saline. The IV will remain in for safety through the end of post-infusion observation.

In the dose-escalation study, the first subject in each dose arm to receive a unique dose level will be observed for at least 4 hours following completion of initial product administration. All other subjects will be observed for at least 2 hours following completion of product administration. In the efficacy study, all subjects will be observed for at least 60 minutes following administration. Prior to discharge from the clinic, vital signs will be recorded and subjects will be assessed for local reactogenicity (including pain/tenderness, swelling, redness, bruising, and pruritus at the site of infusion) and systemic reactogenicity (including fever, feeling unusually tired or unwell, muscle aches, headache, chills, nausea, and joint pain). Any subject who is assessed as being unwell or has ongoing reactogenicity symptoms will be asked to remain in the clinic until evaluation and discharge by a study clinician. If necessary, the subject would be referred to the district hospital to evaluate for safety and possible treatment. Clinicians will follow any solicited symptoms that are ongoing until they have resolved.

6.2 PREPARATION/HANDLING/STORAGE/ACCOUNTABILITY

6.2.1 Acquisition and Accountability

Acquisition: CIS43LS will be shipped from the US to the study site where administration will take place, in compliance with all FDA, US Department of Transportation, and United Nations transport guidelines for shipping biohazardous materials. The placebo product will either be purchased in the US and shipped to Mali at ambient temperature or purchased in Mali.

Accountability: The study pharmacist will be responsible for maintaining an accurate record of the study arm codes, inventory, and an accountability record of study agent supplies. Electronic documentation as well as paper copies may be used.

The empty vials and the unused portion of a vial will be discarded in a biohazard containment bag and incinerated or autoclaved in accordance with the institutional or pharmacy policy. Any unopened vials that remain at the end of the study will be returned to the production facility or discarded at the discretion of the manufacturer in accordance with policies that apply to investigational agents. Partially used vials will not be administered to other subjects or used for *in vitro* experimental studies. These vials will be disposed of in accordance with institutional or pharmacy policy.

6.2.2 Formulation, Appearance, Packaging, and Labeling

VRC-MALMAB0100-00-AB (CIS43LS) is a sterile buffered solution that is filled into single-dose vials. The placebo product (used in the efficacy study) will be sterile isotonic (0.9%) normal saline. Both CIS43LS and placebo are clear liquids and are indistinguishable from one another. The products will be prepared by an unblinded pharmacist and placed in a standard IV infusion bag when preparing for administration.

Vials of CIS43LS will be individually labeled with the name of the material, volume, lot number, concentration, storage instructions, Investigational Use Statement ("Limited by Federal Law to Investigational Use"), and manufacturer information.

6.2.3 Product Storage and Stability

CIS43LS: CIS43LS vials should be stored frozen at −35°C to −15°C in a qualified, continuously-monitored, temperature-controlled freezer.[18] The site pharmacist must promptly report any storage temperature excursions outside of the normal allowance for the storage device to the manufacturer (VRC). The affected product must be quarantined in a separate area under protocol-specific temperature ranges until further notice from the manufacturer. If the excursion results in thawed material, DO NOT REFREEZE; store the thawed, vialed material at 2°C to 8°C.

Provide the following information regarding the excursion to the manufacturer: lot number, fill volume and number of vials affected; temperature range and length of excursion, including data log reports; handling of materials post excursion (e.g., transfer to alternate storage, including times); visual inspection data of the materials (e.g., did the materials appear to have remained frozen, were vials cracked, etc.). The manufacturer will notify the site pharmacist if continued clinical use of the product is acceptable.

Prior to preparation for IV administration, thaw and equilibrate vials for a minimum of 90 minutes at ambient temperature (15°C to 32°C). If thawed vials are removed from 2°C to 8°C, equilibrate at ambient temperature for a minimum of 30 minutes. Prior to preparation for administration in the IV bag, vials should be gently swirled for approximately 30 seconds while avoiding foaming. DO NOT SHAKE THE VIAL.[18]

After product preparation in an IV bag, the prepared CIS43LS may be stored at 2°C to 8°C for a maximum of 24 hours and/or at ambient temperature (15°C to 32°C) for a maximum of 4 hours total including the infusion time. Product may not be stored in direct sunlight.[18]

Placebo: Normal saline will be stored at room temperature in a controlled room per product standards.

6.2.4 Preparation

For each IV infusion order, the subject's weight, dose level, and study arm code will be included in the pharmacy order. To prepare an IV infusion, the pharmacist will do the following:

- For subjects assigned to CIS43LS:
    1) Calculate the total milligrams of CIS43LS needed.
    2) Retrieve the minimum number of thawed vials required to prepare the full dose. After vials are thawed, prior to preparation for administration in the IV bag, vials should be gently swirled for 30 seconds while avoiding foaming. DO NOT SHAKE THE VIAL.
    3) Withdraw the necessary amount of CIS43LS.
    4) Add this volume to a 150 mL capacity, partial fill, 100 mL bag of normal saline using sterile compounding techniques to maintain sterility.
- For subjects assigned to placebo:
    1) Obtain a 100 mL bag of normal saline for administration.

An in-line filter infusion set must be used for IV product administrations and MUST comply with the following specifications: 1.2-micron polyethersulfone filter membrane, diethylhexylphthalate-free, latex-free (equivalent to Braun #473994 filter extension set). When the in-line filter is added to the tubing, the administration set must then be primed.

6.3 Measures to Minimize Bias: Randomization and Blinding

6.3.1 Randomization

In the efficacy study, subjects will be randomized to 1 of 3 treatment arms, including 2 CIS43LS arms and a placebo arm. Randomization lists will be generated by the study statistician, and the randomization code list will be maintained by a designated pharmacist at the study site where the study intervention will take place.

Randomization is further described in section 9.4.1.

6.3.2 Blinding

The efficacy study will be conducted with a double blind. The subjects, the clinical staff, and the study team will be blinded to study treatment allocation. The pharmacy team at the study site

where administration is taking place will be unblinded, and they are responsible for maintaining security of study treatment assignments.

Data will remain blinded until the last subject completes the final study visit. Subjects will then be informed about their study treatment assignment.

Unscheduled unblinding, either intentional (e.g., in the case of a medical emergency in a subject) or unintentional, will be handled according to SOPs. Intentional and unintentional unscheduled unblinding will be documented in the appropriate source and/or research record and will include the reason for the unscheduled unblinding, the date it occurred, who approved the unblinding, who was unblinded, who was notified of the unblinding, and the plan for the subject.

The principal investigator will report all cases of intentional and unintentional unscheduled unblinding to the DSMB in writing within 1 business day after site awareness via email to the DSMB mailbox (niaiddsmbia@niaid.nih.gov) outlining the reason for the unblinding and the date it occurred. The report will also be submitted to the EC. If an SAE has resulted in unblinding, this information will be included in the SAE Report.

6.4 Study Intervention Compliance

Study intervention administration will be documented by study staff.

6.5 Concomitant Therapy

All concomitant prescription and nonprescription (including over-the-counter, herbal, or traditional) medications taken during study participation will be recorded. For this protocol, a prescription medication is defined as a medication that can be prescribed only by a properly authorized/licensed clinician.

Treatment with the following drugs and procedures will not be permitted unless discussed with and approved by the investigator:

- Live vaccines within 4 weeks of study agent administration.
- Killed vaccine within 2 weeks of study agent administration.
- Immunoglobulins and/or blood products for the duration of the study.
- Receipt of any investigational product or co-enrollment in other clinical studies of investigational products.
- Oral or IV corticosteroids at immunosuppressive doses (i.e., prednisone >10 mg/day) or immunosuppressive drugs for the duration of the study.
- Antimalarials and antibiotics with known antimalarial activity.

7 STUDY INTERVENTION DISCONTINUATION AND PARTICIPANT DISCONTINUATION/WITHDRAWAL

7.1 Discontinuation of Study Intervention

Study intervention may be discontinued for a protocol-defined group or arm (i.e., pausing), or it may be discontinued for all subjects and enrollment suspended (i.e., halting). Pausing and halting

are described in sections 8.4.5 and 8.4.6. Subjects who have already received the study agent at the time of a pause or halt will continue planned follow-up under the protocol.

7.2 Participant Discontinuation/Withdrawal from the Study

Plans for managing the involuntary withdrawal of a subject are provided in section 8.4.3. The reason for subject discontinuation or withdrawal from the study will be recorded on the case report form (CRF).

Subjects who withdraw after receiving study agent but prior to study completion will be encouraged to attend an early termination visit, where they will complete as many of the procedures and evaluations indicated in the schedule of activities (section 1.3) as possible.

7.3 Lost to Follow-up

A subject will be considered lost to follow-up if he or she fails to return for 3 consecutive scheduled visits and is unable to be contacted by the study site staff.

The following actions must be taken if a subject fails to return to the clinic for a required study visit:

- The site will attempt to contact the subject and reschedule the missed visit within 7 days, counsel the subject on the importance of maintaining the assigned visit schedule, and ascertain if the subject wishes to and/or should continue in the study.
- Before a subject is deemed lost to follow-up, the investigator or designee will make every effort to regain contact with the subject (where possible, 3 telephone calls). These contact attempts should be documented in the subject's medical record or study file.
- Should the subject continue to be unreachable, he or she will be considered to have withdrawn from the study with a primary reason of lost to follow-up.

8 STUDY ASSESSMENTS AND PROCEDURES

8.1 Screening Procedures

Screening will be performed at the Kalifabougou study site. The study staff will explain the study to the prospective subject, complete the study comprehension examination and obtain consent (section 10.1), and assess eligibility. Consent will be obtained before any study-related procedures are performed.

The following screening procedures and evaluations must be performed within -56 to -7 days of study intervention. Screening may take place over multiple visits if necessary.

- Confirmation of identity, age, and residency.
- Complete review of medical history and medication use.
- Complete physical examination, including height and weight.
- Vital signs (temperature, blood pressure, and pulse).
- 12-lead ECG.
- Urine collection for urinalysis (urine dipstick or formal urinalysis; acceptable laboratory parameters defined in Appendix A).

- For women, serum β-hCG pregnancy test.
- Blood collection via venipuncture for screening evaluations:
  - HIV tests: 2 rapid diagnostic tests (RDTs), plus ELISA if the RDTs are discordant. A subject will be referred for medical care for 2 positive RDTs or a positive ELISA. If the ELISA is negative, no further work-up will be done. Pre- and post-test HIV counseling will be provided.
  - HBV and HCV tests. If either test is positive, the subject will be referred for care regardless of the ALT result.
  - Hemoglobin typing.
  - Complete blood count (CBC) with differential.
  - ALT.
  - Cr.
- Pregnancy prevention confirmation and counseling.
- Pre- and post-test HIV counseling.

A prospective subject who has any clinically significant abnormal finding and/or is diagnosed with a medical condition at screening or during the conduct of the study will be notified and referred for medical care. Per national requirements for reporting communicable diseases, confirmed positive test results for HIV, HBV, and HCV will be reported to the local health department according to applicable laws and appropriate medical referrals initiated. The cost of initial and long-term treatment and care of medical conditions diagnosed during the screening process will not be reimbursed by the study but referrals to relevant specialist will be provided.

Screening evaluations may be repeated as described in section 5.5, at the discretion of the investigator. If screening is completed outside the specified window, all screening procedures and evaluations must be repeated. If an individual screens and is enrolled into the dose-escalation study but for any reason does not receive study agent, they may later consent to be screened and enrolled in the efficacy study.

Enrollment: If the individual is eligible and agrees to participate, he or she will be scheduled to come for an enrollment visit, as described in section 1.3. Enrollment is defined as the time of artemether-lumefantrine administration; at this time, the subject will be assigned a unique study identification number in the clinical database. A clinician will discuss the target dates and timing of the study agent administration and sample collections before completing an enrollment to help ensure that the subject can comply with the projected schedule. The identification number will link subject samples and data collected throughout the study.

8.2 EFFICACY ASSESSMENTS

8.2.1 Clinical Evaluations

The following clinical evaluations will be performed as efficacy assessments.

Medical History and Medication Review: A complete review of all medical history and medications will be conducted at screening. Subsequent visits will include a targeted review of changes in medical history or medications since the last study visit.

Physical Examination: A complete physical examination (including height and weight) will be done at screening. A targeted physical examination based on signs, reported symptoms, and

medical history will be conducted at subsequent study visits. Weight will also be recorded on day 0 prior to study agent administration.

Artemether-Lumefantrine: At enrollment, all subjects will be orally administered standard artemether-lumefantrine treatment (4 tablets twice daily for 3 days) to clear any possible Pf blood-stage infection prior to study agent administration.

Randomization Procedures: Randomization for the efficacy study is described in sections 6.3.1 and 9.4.1.

Study Agent Administration and Monitoring: Study agent infusion and monitoring will be performed according to the assigned arm, as described in section 6.1.

Illness Visit: A subject will be instructed to come in for an unscheduled visit if he or she has symptoms of malaria or other symptoms. The subject will be evaluated by the study team and referred for standard care according to local guidelines. At an illness visit, the subject may undergo review of medical history and concomitant medications, a focused physical exam for symptoms of malaria or other diseases, vital sign measurement, and a fingerprick or venipuncture blood collection for blood smear for malaria diagnosis as well as a dried blood spot for Pf RT-PCR for research purposes.

Malaria Diagnosis and Management: If a subject has a malarial infection, we will share these results with the subject and provide standard treatment in accordance with the recommendations of the Mali National Malaria Control Program. According to the national guidelines, asymptomatic malarial infections in adults are not treated. Malaria treatment is given only when symptoms are present along with positive blood smear results. RT-PCR is not commonly used for routine malaria diagnosis.

8.2.2 Biospecimen Evaluations

Blood will be collected under this protocol by the following methods:

- Venipuncture will be performed with single-use needles. Venous blood samples will be used as follows:
  - Safety evaluations described in section 8.3.
  - Shipment to the research laboratory in Bamako and NIAID for evaluation (including assays described in section 8.2.3) and storage.
  - Blood smear and dried blood spot for PfRT-PCR if unable to obtain ample sample from finger prick
- Fingerprick will be performed using single-use disposable lancets. Fingerprick blood samples will be used as follows:
  - Blood smear and dried blood spot for Pf RT-PCR.

The amount of blood drawn for research purposes will be within the limits allowed for adult research subjects by the NIH Clinical Center: no more than 10.5 mL/kg or 550 mL (whichever is smaller) over an 8-week period.

The collection schedule, volumes, and test tubes are presented in section 1.3.

8.2.3 Correlative Studies for Research/Pharmacokinetic Studies

The following evaluations will be performed according to the schedule presented in section 1.3.

Blood smear: Thick blood smears will be prepared and analyzed by the standard WHO method (section 2.2.3.3) to identify Pf infection for the primary endpoint. This evaluation will be performed centrally in the laboratory in Bamako.

Pf RT-PCR: RT-PCR will be performed to identify Pf infection as a secondary endpoint. This evaluation will be performed at the University of Washington on coded dried blood spots.

PK studies: Blood CIS43LS concentrations will be measured by Meso Scale Discovery LLC–based automation platform. The concentration at the visit prior to the first Pf infection will be used to assess CIS43LS-mediated protection. This evaluation will be performed at the NIAID.

ADA detection: Assays for detection of ADA will be performed at specified timepoints (section 1.3) following product administration and compared to baseline status. This evaluation will be performed at the NIAID.

Parasite genotyping: For subjects who become infected during the study, coded blood samples collected around the time of the first infection will be used to perform a genotypic sieve analysis to analyze sequences of breakthrough parasites (section 9.4.11). This evaluation will be performed by Dr. Daniel Neafsey at the Harvard School of Public Health.

8.2.4 Samples for Genetic/Genomic Analysis

8.2.4.1 Description of the scope of genetic/genomic analysis

Genetic testing performed in this protocol will be limited to analyzing the genetic material of infection-inducing parasites in blood samples. No human genetic analyses will be performed.

8.2.4.2 Description of how privacy and confidentiality of medical information/biological specimens will be maximized

Privacy and confidentiality of medical information and biological samples is described in section 10.3.

8.2.4.3 Management of Results

As no human genetic analyses will be performed in this protocol, no genetic results will be returned to subjects.

8.2.4.4 Genetic counseling

Not applicable.

8.3 Safety and Other Assessments

The following study procedures and evaluations will be done according to the schedule in section 1.3 to monitor safety and support the understanding of the study intervention's safety. The assessment and collection of safety events such as AEs are described in section 8.4.2.

Physical Examination: As described in section 8.2.1, physical examination will also be performed for assessment of safety.

Vital Signs: Vital signs (temperature, blood pressure, and heart rate) will be collected at visits, including before and after study agent infusion, as described in section 8.2.1.

Safety Blood Laboratory Evaluations: The following safety laboratory evaluations will be performed at a frequency presented in section 1.3:

- CBC with differential.
- ALT, Cr.

Pregnancy Testing: Women will have a serum pregnancy test at screening and urine tests at enrollment, day 0 (confirmed negative prior to administration of the study agent), and monthly through the final study visit.

Pregnancy Prevention Counseling: Female subjects will be counseled on the importance of not becoming pregnant during study participation and on acceptable methods of contraception.

8.4 Safety Definitions, Management, and Sponsor Reporting

8.4.1 Definitions

Adverse Event: An AE is any untoward or unfavorable medical occurrence in a human subject, including any abnormal sign (e.g., abnormal physical exam or laboratory finding), symptom, or disease, temporally associated with the subject's participation in the research, whether or not considered related to the research.

Adverse Reaction: An AR means any AE caused (see "Causality" below) by a study agent. ARs are a subset of all suspected adverse reactions (SARs; defined below) where there is reason to conclude that the study agent caused the event.

Suspected Adverse Reaction: SAR means any AE for which there is a reasonable possibility that the study agent caused the AE.

Per US FDA guidance:

For the purposes of IND safety reporting, "reasonable possibility" means there is evidence to suggest a causal (see "Causality" below) relationship between the study agent and the AE. A SAR implies a lesser degree of certainty about causality than an AR, which means any AE caused by a study agent.

SARs are the subset of all AEs for which there is a reasonable possibility that the study agent caused (see "Causality" below) the event. Inherent in this definition, and in the requirement to report SARs, is the need for the sponsor to evaluate the available evidence and make a judgment about the likelihood that the study agent actually caused the AE.

The sponsor is responsible for making the causality judgment.

Serious Adverse Event: An SAE:

- is an AE that results in death.

- is an AE that is life-threatening event (places the subject at immediate risk of death from the event as it occurred).
- is an AE that requires inpatient hospitalization or prolongs an existing hospitalization.

  NOTE:

  - Hospitalization is considered required if outpatient treatment would generally be considered inappropriate.
  - Same-day surgical procedures that are required to address an AE are considered hospitalizations, even if they do not involve an overnight admission.
  - Hospitalization due to a condition that has not worsened and that pre-dates study participation (e.g., elective correction of an unchanged baseline skin lesion), or due to social circumstance (e.g., prolonged stay to arrange aftercare), or that is planned/required "per protocol" AND that proceeds without prolongation or complication, is NOT considered an SAE by this criterion.
- is, or results in, a persistent or significant incapacity or substantial disruption of the ability to conduct normal life functions.
- is a congenital anomaly/birth defect/miscarriage/stillbirth.

  NOTE: This definition is more inclusive than some commonly published definitions. It includes an affected conceptus/neonate whose:

  - biological mother was exposed to a study agent at any point from conception through the end of the pregnancy, AND/OR, if breastfeeding, the 30-day neonatal period; or
  - biological father was exposed to a study agent at any point during the 90 days prior to conception.

  This is separate from, and in addition to, general reporting of pregnancy in a study participant or female partner of a male participant (see section 8.4.2.3.4 below).

- is a medically important event.

  NOTE: Medical and scientific judgment should be exercised. Events that significantly jeopardize the subject and/or require intervention to prevent one of the SAE outcomes listed above are generally considered medically important, and are thus SAEs.

Unexpected Adverse Event: An AE is unexpected if it is not listed in the investigator's brochure or package insert (for marketed products) at the frequency, AND specificity, AND severity that has been observed.

NOTE:

- Such events should also be evaluated for possible reporting as unanticipated problems (UPs) (see section 8.4.2.3.3 below).
- Unexpected, as used in this definition, also refers to AEs or SARs that are mentioned in the investigator's brochure as occurring with a class of drugs/biologics, or as anticipated from the pharmacological properties of the study agent but are not specifically mentioned as occurring with the particular study agent under investigation.

Serious and Unexpected Suspected Adverse Reaction (SUSAR): A SUSAR is an SAR (defined above) that is both serious and unexpected.

Unanticipated Problem: A UP is any incident, experience, or outcome that meets all the following criteria:

1. Unexpected (in terms of nature, severity, or frequency) given
   a. the research procedures that are described in the protocol-related documents, such as the EC-approved research protocol and informed consent document; and
   b. the characteristics of the subject population being studied; and
2. Related or possibly related to participation in the research (possibly related means there is a reasonable possibility that the incident, experience, or outcome may have been caused by the procedures involved in the research), and
3. Suggests the research places subjects or others (which may include research staff, family members or other individuals not directly participating in the research) at a greater risk of harm (including physical, psychological, economic, or social harm) related to the research than was previously known or expected.

NOTE:

- Per the sponsor, an SAE always meets this "greater risk" criterion.
- An incident, experience, or outcome that meets the definition of a UP generally will warrant consideration of changes to the protocol or informed consent form, or to study procedures (e.g., the manual of procedures for the study), in order to protect the safety, welfare, or rights of participants or others. Some UPs may warrant a corrective and preventive action plan at the discretion of the sponsor or other oversight entities.

Unanticipated Problem that is not an Adverse Event (UPnonAE): A UPnonAE belongs to a subset of UPs that:

- meets the definition of a UP, AND
- does NOT fit the definition of an AE or an SAE.

NOTE: Examples of UPnonAEs include, but are not limited to:

- a breach of confidentiality
- prolonged shedding of a vaccine virus beyond the anticipated timeline
- unexpectedly large number of pregnancies on a study
- subject departure from an isolation unit prior to meeting all discharge criteria
- accidental destruction of study records
- unaccounted-for study agent
- overdosage, underdosage, or other significant error in administration or use of study agent or intervention, even if there is no AE/SAE
- development of an actual or possible concern for study agent purity, sterility, potency, dosage, etc.

  NOTE: A decision to temporarily quarantine, or to permanently not use all or part of study agent supply due to an unexpected finding or event (e.g., particulate, cloudiness, temperature excursion), even if there is no known or

proven issue (i.e., out of an "abundance of caution"), is considered a UPnonAE.

Protocol Deviation: Any change, divergence, or departure from the EC-approved research protocol.

1. **Major deviations:** Deviations from the EC-approved protocol that have, or may have the potential to, negatively impact the rights, welfare, or safety of the subject, or to substantially negatively impact the scientific integrity or validity of the study.
2. **Minor deviations:** Deviations that do not have the potential to negatively impact the rights, safety, or welfare of subjects or others, or the scientific integrity or validity of the study.

Noncompliance: Failure of investigator(s) to follow the applicable laws, regulations, or institutional policies governing the protection of human subjects in research, or the requirements or determinations of the EC, whether intentional or not.

1. **Serious noncompliance:** Noncompliance, whether intentional or not, that results in harm or otherwise materially compromises the rights, welfare and/or safety of the subject. Noncompliance that materially affects the scientific integrity or validity of the research may be considered serious non-compliance, even if it does not result in direct harm to research subjects.
2. **Continuing noncompliance:** A pattern of recurring noncompliance that either has resulted, or, if continued, may result in harm to subjects or otherwise materially compromise the rights, welfare and/or safety of subjects, affect the scientific integrity of the study or validity of the results. The pattern may comprise repetition of the same non-compliant action(s), or different noncompliant events. Such non-compliance may be unintentional (e.g., due to lack of understanding, knowledge, or commitment), or intentional (e.g., due to deliberate choice to ignore or compromise the requirements of any applicable regulation, organizational policy, or determination of the EC).

8.4.2 Documenting, Assessing, Recording, and Reporting Events

ALL AEs, including those that may appear to have a non-study cause (see "Causality" below), will be documented (e.g., on the clinical chart/progress notes/clinical laboratory record), recorded (e.g., in the study-specified CRF/research database), and reported (e.g., cumulatively from the research database, or according to protocol-specified expedited reporting mechanism) to the sponsor from the time informed consent is obtained through the timeframe specified below. At each contact with the subject, information regarding AEs will be elicited by open-ended questioning and examinations.

AEs and SAEs will generally be recorded, assessed, and reported according to the timeframes outlined in Table 2.

Table 2. Standard event recording, assessment, and reporting timeframes.

Event type	Record, assess, and report through
Related SAEs	End of subject participation in study, or if study personnel become aware thereafter
Unrelated SAEs	End of subject participation in study
Related non-serious AEs of grade 1 to 3	End of subject participation in study
All other related non-serious AEs	End of subject participation in study
Unrelated non-serious AEs	End of subject participation in study

8.4.2.1 Investigator Assessment of Adverse Events

The investigator will assess all AEs with respect to **seriousness** (according to SAE definition above), **severity** (intensity or grade, see below), and **causality** (relationship to study agent and relationship to participation in the research, see below).

8.4.2.1.1 Severity Grading

The investigator will grade the severity of fever (by non-oral temperature reading) and each blood laboratory testing AE according to the "Mali Adverse Event Grading Scale" provided in APPENDIX B: MALI ADVERSE EVENT GRADING SCALE. Events that are not gradable using this table (e.g., urinalysis abnormalities) will be graded according to the "Guidance for Industry: Toxicity Grading Scale for Healthy Adult and Adolescent Volunteers Enrolled in Preventive Vaccine Clinical Trials" which can be found at: https://www.fda.gov/regulatory-information/search-fda-guidance-documents/toxicity-grading-scale-healthy-adult-and-adolescent-volunteers-enrolled-preventive-vaccine-clinical

Events that are NOT gradable using either of the above specified tables will be graded as follows:

- Mild = grade 1
- Moderate = grade 2
- Severe = grade 3
- Potentially life threatening = grade 4
- Death = grade 5

NOTE: A subject death should always be reported as grade 5.

8.4.2.1.1.1 Laboratory Value Assessment and Clinical Significance Criteria

Except as specified below, ALL abnormal lab values of grade 1 or above are REPORTABLE.

Grade 1 and 2 abnormal laboratory values are considered CLINICALLY SIGNIFICANT, and are to be recorded in the research database, and reported, if they meet ONE or more of the following criteria:

- result in a study agent dosage adjustment, interruption, or discontinuation
- are accompanied by clinically abnormal signs or symptoms that are likely related to the laboratory abnormality (e.g., clinical jaundice)
- indicate a possible organ toxicity (e.g., elevated serum creatinine)
- result in additional/repeat testing or medical intervention (procedures/treatments) (e.g., ECG to evaluate arrhythmia potential with a high serum potassium; one or more ECGs to assess an elevated troponin level; potassium supplementation for hypokalemia)
- indicates possible over-dosage
- are considered clinically significant by the investigator or SMM

8.4.2.1.2 Causality

Causality (likelihood that the event is caused by the study agents) will be assessed by the principal investigator or designee considering the factors listed under the following categories:

Definitely Related

- reasonable temporal relationship
- follows a known response pattern
- clear evidence to suggest a causal relationship
- there is no alternative etiology

Probably Related

- reasonable temporal relationship
- follows a suspected response pattern (based on similar agents)
- no evidence of a more likely alternative etiology

Possibly Related

- reasonable temporal relationship
- little evidence for a more likely alternative etiology

Unlikely Related

- does not have a reasonable temporal relationship

AND/OR

- there is good evidence for a more likely alternative etiology

Not Related

- does not have a temporal relationship

AND/OR

- definitely due to an alternative etiology

Note: Other factors (e.g., dechallenge, rechallenge, if applicable) should also be considered for each causality category when appropriate. Causality assessment is based on available information at the time of the assessment of the AE. The investigator may revise the causality assessment as additional information becomes available.

Causality assessment will be reviewed by the sponsor. The sponsor may make a separate and final determination on the "reasonable possibility" that the event was "related" (comprising definitely, probably, and possibly related) or "unrelated" (comprising unlikely and not related) to the study agent, in keeping with applicable (US FDA) guidance on sponsor IND safety reporting.

8.4.2.2 Recording of Events

AEs will be promptly recorded in the research database, regardless of possible relationship to study interventions. If a diagnosis is clinically evident (or subsequently determined), the diagnosis rather than the individual signs and symptoms or laboratory abnormalities will be recorded as the AE. The investigator will review events regularly to ensure they have been captured correctly and to perform assessment of events individually and cumulatively to assess possible safety trends.

8.4.2.3 Investigator Reporting Responsibilities

The principal investigators and/or equally qualified designees will check daily for events that may require expedited reporting.

The principal investigators and/or equally qualified designees will also monitor all accumulating data no less than weekly, or according to superseding NIH or NIAID policy, whichever is more frequent.

Data will be reviewed by the principal investigators/designees on a regular basis for accuracy and completeness.

Data will be submitted to the sponsor in keeping with all applicable agreements and when requested, such as for periodic safety assessments, review of IND annual reports, review of IND safety reports, and preparation of final study reports.

The principal investigators and/or other study designee will ensure prompt reporting to safety oversight bodies (e.g., Clinical Safety Office [CSO], DSMB), regulatory entities, and stakeholders as specified below, and according to any additional requirements or agreements.

8.4.2.3.1 Adverse Events

Unless otherwise specified above, AE data will be entered into the research database no less than every other week and will include all data through 1 week prior to database entry.

8.4.2.3.2 Serious Adverse Events (Expedited Reporting)

Unless otherwise specified above, all SAEs (regardless of relationship and whether or not they are also UPs) must be reported to the CSO as specified by the CSO (e.g., Research Electronic Data Capture [REDCap] system; use the Safety Expedited Report Form [SERF]/email if REDCap is not available). If the preferred/indicated mechanism for reporting is not available, the CSO/SMM should be contacted by telephone, fax, or other reasonable mechanism to avoid delays in reporting.

CSO CONTACT INFORMATION:

Clinical Safety Office

5705 Industry Lane
Frederick, MD 21704
Phone: [redacted]
Fax: [redacted]
Email: [redacted]

Unless otherwise specified above, deaths and immediately life-threatening SAEs must be reported to the CSO promptly, and no later than the **first business day** following the day of study personnel awareness.

All other SAEs must be reported to the CSO no later than the **third business day** following the day of study personnel awareness.

If an individual subject experiences multiple SAEs in a closely timed/overlapping "cause-and-effect" (cascade) sequence, the principal investigators, after careful evaluation, will report ONLY primary/precipitating event(s) individually. SAEs that are determined to be definitely secondary to other SAEs will be detailed in the narrative portion of the report of the relevant primary/precipitating SAE. A clinical rationale and findings to support such reporting should be part of the narrative.

For each SAE report, the research database entry MUST match the corresponding entries on the SAE report (e.g., start and stop dates, event type, relationship, and grade), and **must be updated if necessary** (e.g., if the SAE report was generated after the corresponding AE was entered in the research database).

Unless otherwise specified above, SAEs that have not resolved by the end of the per-protocol follow-up period for the subject are to be followed until final outcome is known (to the degree permitted by the EC-approved informed consent form). If it is not possible to obtain a final outcome for an SAE (e.g., the subject is lost to follow-up), and to update the CSO, the last known status and the reason a final outcome could not be obtained will be recorded by the investigator on an SAE report update and the CRF.

8.4.2.3.3 Unanticipated Problems

Unless otherwise specified above, UPs (as defined in this protocol, or as defined by the EC of record, whichever definition is more conservative) that are also AEs or SAEs, must be reported to the CSO (by REDCap, or by email and SERF if REDCap is not available) no later than when they are due to be reported to the EC.

UPnonAEs are NOT reported to the CSO but must be reported to the Clinical Trials Management (CTM) group according to their requirements and preferred methods. If the UPnonAE raises a significant potential subject safety concern, the SMM should be consulted by email or phone no later than when reports are made to the CTM.

8.4.2.3.4 Pregnancy

Unless otherwise specified above, all pregnancies will be reported (by REDCap, or by email and SERF if REDCap is not available) to the CSO no later than the first business day following the day of study personnel awareness.

Pregnancy outcome data (e.g., delivery outcome, spontaneous or elective termination of the pregnancy) will be reported to the CSO no later than the third business day following the day of study personnel awareness (by REDCap, or by email and SERF if REDCap is not available).

Pregnancy itself is not an AE. Events that meet AE or SAE criteria in relation to pregnancy, delivery, or the conceptus/neonate (see section 8.4.1) are reportable (by REDCap, or by email and SERF if REDCap is not available).

In the event of pregnancy in a study subject exposed to study agent, the following actions will be taken, with the goal of ensuring maternal and fetal well-being, in consultation with the SMM, independent safety monitor (ISM), and DSMB:

- Sample collection will continue (including participation in extension visits described in Appendix C) per guidelines below:
    - Only proceed with blood draw if subject's hemoglobin value is ≥ 8.0 gm/dL (assessed via finger prick).
    - PK samples (4 mL) will be collected according to the regular study schedule (Day 1, Day 7, Day 14, Day 28, Day 56, Day 84, Day 112, Day 140 and Day 168), plus optional extension visits.
    - The finger prick used to measure hemoglobin at the visits specified above will also be used to collect blood smears for detection of Pf infection by microscopic examination, and dried blood spots for PfRT-PCR. A venipuncture (up to 0.5 mL) may also be performed if the investigators believe that a finger prick would not provide a sufficient volume of blood to prepare dried blood spots for the PfRT-PCR assay.
    - Serum storage samples will not be drawn if a subject becomes pregnant.

- Continue to follow for safety for the duration of the pregnancy and for a period of up to 12 months (per investigator discretion) following delivery for assessment of the neonate (see Appendix D).
- Request to unblind the subject, if applicable, AND if doing so would offer a benefit to the subject.
- Report, no later than the first business day after study personnel awareness, to the ISM and DSMB.
- Advise subject to notify the obstetrician of study participation and study agent exposure, providing contact information for the obstetrician to contact the study principal investigator, should this be required, and with the subject's consent.

8.4.2.4 Sponsor's Reporting Responsibilities

Events reported to the sponsor will be promptly evaluated and will be reported as required according to FDA IND safety reporting guidance and regulations. IND safety reports will be sent to other investigators conducting research under the same IND and will be shared with other stakeholders according to applicable agreements.

The sponsor will also submit an IND annual report of the progress of the investigation to the FDA as defined in 21 CFR 312.33.

All UPs will be evaluated by the sponsor, and a summary of the event, and any necessary (corrective/preventative) actions, will be distributed to investigators conducting research under the same IND as may be relevant and appropriate.

8.4.3 Withdrawal Criteria for an Individual Subject

An individual subject will be withdrawn from the study for any of the following:

- An individual subject's decision. (The investigator should attempt to determine the reason for the subject's decision.)
- Non-compliance with study procedures to the extent that it is potentially harmful to the subject or to the integrity of the study data.
- A change in the subject's condition as follows:
  - Loss of the ability to provide informed consent.
  - Withdraws permission to have blood samples or data stored for future research.
- The investigator determines that continued participation in the study would not be in the best interest of the subject.

8.4.3.1 Re-enrollment and Unplanned Procedure Repetition

Unless otherwise specified within this protocol (e.g., rescreening as described in section 5.5), each person who is a subject in this study may be enrolled and may pass through each step and process outlined in the protocol, only **ONCE** (i.e., subjects may not "go back" and repeat a

protocol step already completed). On a case-by-case basis, a request for re-enrollment, or for repetition of a protocol step or procedure already completed, may be submitted to, reviewed by, and approved by the SMM in writing. The SMM may also recommend or require consultation of the EC and/or DSMB and ISM.

8.4.3.2 Replacement of Withdrawn Subjects or Subjects Who Discontinue Study Agent

In the dose-escalation study, subjects withdrawn prior to the day 7 safety evaluation will be replaced. In the efficacy study, subjects withdrawn prior to study agent administration will be replaced.

All subjects exposed to study agents MUST be included in the safety dataset.

8.4.4 Additional Safety Oversight

8.4.4.1 Safety Review and Communications Plan

A safety review and communication plan (SRCP) is required for this protocol. The SRCP is an internal communications document between the principal investigators and the CSO, as sponsor representative, which delineates key safety oversight responsibilities of the principal investigators, the CSO, and other stakeholders. The SRCP includes a plan for conducting periodic safety surveillance assessments by the CSO.

8.4.4.2 Sponsor Medical Monitor

A SMM, representing the sponsor, has been appointed for oversight of safety in this clinical study. The SMM will be responsible for performing safety assessments as outlined in the SRCP.

8.4.4.3 Oversight Committees

8.4.4.3.1 Independent Safety Monitor in Mali

The ISM is an expert who does not have direct involvement in the conduct of the study and has no significant conflicts of interest as defined by NIAID policy. An ISM in Mali will review the study prior to initiation and will be available to advise the investigators on study-related medical issues and act as a representative for the welfare of the subjects. The ISM will conduct independent safety monitoring. The ISM is an expert in the field of oversight of clinical trials conducted in Mali and internal medicine, specifically in the population under study in Mali.

All deaths, SAEs, UPs, pregnancies, and FDA IND safety reports will be reported by the principal investigators to the ISM prior to or at the same time they are submitted to the EC or CSO unless otherwise specified herein. The ISM will be notified immediately if any pausing rule is met. The principal investigators will also notify the ISM if intentional or unintentional unblinding occurs. If the ISM is unblinded to the study agent given to an individual subject during medical management, the ISM will report that unblinding to the DSMB Executive Secretary. The ISM will have access to unblinded data, by group, only as the entire DSMB does. The ISM may also serve as an *ad hoc* member of the DSMB.

8.4.4.3.2 Data and Safety Monitoring Board

The NIAID intramural DSMB includes independent experts that do not have direct involvement in the conduct of the study and have no significant conflicts of interest as defined by NIAID policy. The DSMB will review the study protocol, consent documents, and investigator brochure prior to initiation and twice a year thereafter, or as may be determined by the DSMB. Additionally, the DSMB will conduct 1 interim analysis when safety data are available from the dose-escalation study subjects (section 9.4.8).

The DSMB may convene additional reviews as necessary. The DSMB will review the study data as needed to evaluate the safety, efficacy, study progress, and conduct of the study.

All deaths, SAEs, UPs, pregnancies, and IND safety reports will be reported to the DSMB at the same time they are submitted to the EC and CSO unless otherwise specified herein.

All cases of intentional or unintentional unblinding will be reported to the DSMB not later than 1 business day from the time of study personnel awareness.

The principal investigators will notify the DSMB at the time pausing or halting criteria are met and obtain a recommendation concerning continuation, modification, or termination of the study.

8.4.5 Pausing Rules

"Pausing" is discontinuation of study intervention/treatment/dosing (agent/placebo/procedure, etc.) in a protocol-defined group or "arm," until a decision is made to either resume or permanently discontinue such activity. Subjects continue to be followed for safety during a pause.

The pausing criteria for a group or arm (e.g., a specific dosing group) in this study include any one or more of the following:

- A subject experiences an SAE that is unexpected (per the investigator's brochure or product label) and possibly, probably, or definitely related to a study agent;
- A subject experiences 2 grade 3 or greater AEs that are unexpected (per the investigator's brochure or product label) and possibly, probably, or definitely related to a study agent.

The principal investigators or the CSO may also pause dosing/study interventions for one or more subjects for any safety issue. The study safety oversight bodies (e.g., DSMB, ISM) may recommend a pause to the CSO.

8.4.5.1 Reporting a Pause

If a pausing criterion is met, a description of the AE(s) or safety issue must be reported by the principal investigators within 1 business day to the CSO and the EC according to their requirements. The principal investigators will also notify the DSMB and ISM. In addition, the CSO or designee will notify all other site investigators by email or through the specified pathway.

8.4.5.2 Resumption Following a Pause

The CSO, in collaboration with the principal investigators and DSMB and ISM, will determine if study activities, including agent administration and/or other study interventions may be resumed, and any additional modifications or requirements that may apply, for the impacted subject(s), or whether the events that triggered the pause require expansion to a study halt (see below).

The CSO or sponsor designee will notify the principal investigators of the decision. The principal investigators will notify the EC of the decision according to the EC's process.

8.4.5.3 Discontinuation of Study Agent

A subject who does not resume study agent/intervention/treatment will continue to be followed for protocol-specified safety assessments or as clinically indicated, whichever is more conservative.

8.4.6 Halting Rules for the Protocol

"Halting" is discontinuation of study intervention/treatment/dosing (agent/placebo/procedure, etc.) for all subjects in a study and suspension of enrollment until a decision is made to either resume or permanently discontinue such activity. Subjects continue to be followed for safety during a halt.

The halting rules are:

- Two or more subjects experience the same or similar grade 3 or greater AEs that are unexpected and possibly, probably, or definitely related to a study agent;

  OR

- Any safety issue that the principal investigators or the CSO determines should halt the study. The study safety oversight bodies (e.g., DSMB) may recommend a halt to the CSO.

In addition, the FDA, Malian Ministry of Health, FMPOS EC, or any regulatory body having oversight authority may halt the study at any time. The DSMB or ISM may recommend a study halt.

8.4.6.1 Reporting a Study Halt

If a halting criterion is met, a description of the AE(s) or safety issue must be reported by the principal investigators, within 1 business day to the CSO and the EC according to their requirements. The principal investigators will also notify the DSMB and ISM. In addition, the CSO or designee will notify all other site investigators by email or through the specified pathway.

8.4.6.2 Resumption of a Halted Study

The CSO, in collaboration with the principal investigators and DSMB and ISM will determine if study activities, including enrollment, study agent administration, and/or other study interventions, may be resumed and any additional modifications or requirements that may apply.

The CSO or sponsor designee will notify the principal investigators of the decision. The principal investigators will notify the EC of the decision according to the EC's process.

8.4.6.3 Discontinuation of Study Agent

Subjects who do not resume study agent/study intervention will continue to be followed for protocol-specified safety assessments or as clinically indicated, whichever is more conservative.

8.5 Unanticipated Problems

8.5.1 Definition of an Unanticipated Problem

The definition of a UP is provided in section 8.4.1.

8.5.2 Unanticipated Problem Reporting

The investigator will report UPs to the FMPOS EC according to NIH Human Research Protection Program (HRPP) Policy 801, as described in section 8.6.1.

8.6 ADDITIONAL REPORTING REQUIREMENTS

8.6.1 Reporting to the FMPOS EC

Non-compliance and other reportable events will be reported to the FMPOS EC according to NIH HRPP Policy 801, which requires reporting as described below.

The following will be reported within 7 calendar days of any investigator or individual associated with the protocol first becoming aware:

- Actual or suspected noncompliance.
- Actual or suspected major deviation.
- Actual or suspected UPs.
- New information that might affect the willingness of a subject to enroll or remain in the study.
- Suspension or termination of research activities, including holds on new enrollment, placed upon the research by the sponsor, NIH or NIAID leadership, or any regulatory agency.

Any death of a research subject that is possibly, probably, or definitely related to the research must be reported within 24 hours of the investigator becoming aware of the death.

Additionally, investigators must provide the following information to the EC in summary format at the time of continuing review, or when otherwise specifically requested by the EC or the Office of Human Subjects Research Protections (OHSRP) Office of Compliance and Training:

- Major and minor protocol deviations.
- Noncompliance reported to the EC that is not related to a protocol deviation.
- AEs and SAEs that do not meet the definition of an UP.
- UPs reported to the EC.

8.6.2 Reporting to the NIAID Clinical Director

The principal investigator will report UPs, major protocol deviations, and deaths to the NIAID clinical director according to institutional timelines.

8.6.3 Reporting Protocol Deviations that Result from the COVID-19 Pandemic

The following addresses the reporting requirements to the FMPOS EC with regard to protocol deviations that result from disruption of study visits from the COVID-19 pandemic. These requirements follow the direction of the FMPOS EC as well as the NIH reporting requirements.

Investigators may modify the protocol without prospective FMPOS EC approval when necessary to prevent an immediate apparent harm to a study subject. Typically, when this occurs the event must be reported to the EC via a Reportable Event Form (REF) within 7 days of the deviation. Given the potential need for this to occur on a much larger than usual scale, it is not required that all planned deviations be reported to the FMPOS EC in an expedited timeframe. Only those deviations which meet the definition of a major deviation will require reporting, as defined in section 8.4.1.

If a subject cannot complete a protocol-specified study visit or intervention, the principal investigator should assess the impact of the missed visit on the safety of the subject and the scientific validity of the trial. If in the principal investigator's determination neither of these are meaningfully impacted by the deviation, these do not need to be reported to the FMPOS EC in an expedited manner. The event should be included in the summary of events reported at the time of continuing review.

If in the opinion of the principal investigator the missed visit or intervention poses a risk to the safety of the subject, the investigator should develop a plan to minimize the impact of the deviation. For example, if the subject is scheduled to return to the study site for safety lab work, the investigator may arrange for labs to be drawn at a location closer to the subject's home. In cases such as this, if the change is necessary to assure the safety of the subject, the investigator may implement the change without prospective FMPOS EC approval. If the change meets the definition of a major deviation, it must be reported via a REF within 7 days.

9 STATISTICAL CONSIDERATIONS

9.1 Statistical Hypothesis

The hypotheses are that CIS43LS will be safe and will confer protection against Pf infection. In the dose-escalation study, the primary objective is to evaluate the safety and tolerability. In the efficacy study, the primary objective is to evaluate the safety and efficacy of CIS43LS compared to placebo.

Primary Endpoints:

- Dose escalation: Incidence and severity of local and systemic AEs occurring within 7 days after the administration of CIS43LS
- Efficacy: Pf blood stage infection as detected by microscopic examination of thick blood smear for 24 weeks after administration of CIS43LS or placebo

Secondary Endpoints:

- Dose escalation: Measurement of CIS43LS in sera of recipients
- Efficacy: Pf blood stage infection as detected by RT-PCR for 24 weeks after administration of CIS43LS or placebo
- Efficacy: Measurement of CIS43LS in sera of recipients

9.2 Sample Size Determination

9.2.1 Sample Size Considerations for the Dose-Escalation Study

The ability of the study to identify safety events can be expressed in terms of the probability of observing 1 or more event of interest (e.g., AEs) within each arm. With the sample size n=6 in each arm, there is over a 90% chance to observe at least 1 AE if the true rate is at least 0.319 and over a 90% chance to observe no AE if the true rate is no more than 0.017. With n=18 over 3 dose arms, there is over a 90% chance to observe at least 1 AE if the true rate is no less than 0.121 and over a 90% chance of observing no AE if the true rate is no more than 0.005. Probabilities of observing 0 or more than 1 AE within a group are presented in Table 3 for a range of possible true event rates.

Table 3. Probability (Pr) of events for different safety scenarios within an arm (n=6 or 18).

True event rate	n=6		n=18	
	Pr(0)	Pr(>1)	Pr(0)	Pr(>1)
0.005	0.970	0.000	0.914	0.004
0.01	0.941	0.001	0.835	0.014
0.02	0.886	0.006	0.695	0.050
0.035	0.808	0.017	0.527	0.130
0.05	0.735	0.033	0.397	0.226
0.1	0.531	0.114	0.150	0.550
0.15	0.377	0.224	0.054	0.776
0.2	0.262	0.345	0.018	0.901
0.3	0.118	0.580	0.002	0.986

9.2.2 Sample Size Considerations for the Efficacy Study

The efficacy study is designed to evaluate protective efficacy (PE) of CIS43LS at each dose by testing the null hypothesis,

H0: protective efficacy ≤ 0%,

versus the alternative hypothesis,

H1: protective efficacy > 0%,

where PE is 1 minus the ratio of the infection rate under CIS43LS over the infection rate under placebo.

The sample size considerations are based on the power of rejecting H0 over a range of possible protective efficacy and infection rates under placebo. Assuming a drop-out rate of 10%, Table 4 presents the power under a 2-sided significance level of 0.025. With 110 subjects enrolled in each arm, the trial has at least 80% power in each comparison to claim protective efficacy of CIS43LS if the underlying efficacy is greater than or equal to 0.5 and the infection rate under placebo is no less than 0.4.

Table 4. Power for efficacy evaluation under a 2-sided type I error rate of 0.025.

Sample size per arm	Infection rate under placebo	Protective efficacy	Power
100	0.4	0.4	52
	0.4	0.5	76
	0.4	0.6	92
	0.5	0.4	69
	0.5	0.5	90
	0.5	0.6	98
	0.6	0.4	84
	0.6	0.5	97
	0.6	0.6	100
	0.7	0.4	95
	0.7	0.5	100
	0.7	0.6	100
110	0.4	0.4	57
	**0.4**	**0.5**	**80**
	0.4	0.6	94
	0.5	0.4	74
	0.5	0.5	93
	0.5	0.6	99
	0.6	0.4	88
	0.6	0.5	98
	0.6	0.6	100
	0.7	0.4	96
	0.7	0.5	100
	0.7	0.6	100

Sample size per arm	Infection rate under placebo	Protective efficacy	Power
120	0.4	0.4	61
	0.4	0.5	84
	0.4	0.6	96
	0.5	0.4	78
	0.5	0.5	95
	0.5	0.6	99
	0.6	0.4	91
	0.6	0.5	99
	0.6	0.6	100
	0.7	0.4	98
	0.7	0.5	100
	0.7	0.6	100

Table 5 presents the minimum detectable difference between a CIS43LS arm and a placebo arm in terms of the AE rate based on a two-sided proportion test with type I error rate of 0.025.

Table 5. Minimum detectable difference in adverse event rate in arms receiving monoclonal antibody (MAb) or placebo under a 2-sided type I error rate of 0.025.

Sample size within each arm (#)	Event rate (in placebo) (%)	Detectable with 80% power		Detectable with 90% power	
		Difference (%)	Event rate (in MAb) (%)	Difference (%)	Event rate (in MAb) (%)
100	0.1	9.1	9.2	11.6	11.7
	0.5	9.8	10.3	12.3	12.8
	1.0	10.5	11.5	13.0	14.0
	2.0	11.6	13.6	14.2	16.2
	5.0	14.1	19.1	16.8	21.8
110	0.1	8.4	8.5	10.6	10.7
	0.5	9.0	9.5	11.3	11.8
	1.0	9.7	10.7	12.0	13
	2.0	10.8	12.8	13.2	15.2
	5.0	13.2	18.2	15.7	20.7
120	0.1	7.7	7.8	9.8	9.9
	0.5	8.4	8.9	10.5	11.0
	1.0	9.0	10.0	11.2	12.2
	2.0	10.2	12.2	12.3	14.3

Sample size within each arm (#)	Event rate (in placebo) (%)	Detectable with 80% power		Detectable with 90% power	
		Difference (%)	Event rate (in MAb) (%)	Difference (%)	Event rate (in MAb) (%)
	5.0	12.5	17.5	14.9	19.9

9.3 Populations for Analyses

The following datasets will be considered in study analyses:

- Intention-to-treat (ITT) analysis dataset will include all subjects that receive assignment and will be analyzed according to the initial randomization assignment.
- Modified intention-to-treat (MITT) analysis dataset will include all randomized subjects that receive the study intervention and will be analyzed according to the initial randomization assignment.
- Per-protocol analysis dataset will include all randomized subjects that receive the study intervention consistent with the initial randomization assignment and complete the scheduled visits, and will be analyzed according to the initial randomization assignment. In cases where subjects receive an intervention other than the one randomly assigned, an as-treated analysis will be additionally performed according to the actual intervention received.

9.3.1 Evaluable for Toxicity

All subjects will be evaluable for toxicity from the time of their first treatment with CIS43LS or placebo.

9.3.2 Evaluable for Objective Response

Not applicable.

9.3.3 Evaluable Non-Target Disease Response

Not applicable.

9.4 Statistical Analyses

9.4.1 General Approach

In general, descriptive statistics will be tabulated by treatment arm for endpoints of interest. This will include point estimates (mean, geometric mean, median, or proportions) and their respective 95% confidence intervals. Formal comparisons will use standard methods, contingency tables for categorical variables, t-tests for comparing means if data follow a normal distribution or geometric means if data after log transformation follow a normal distribution, or nonparametric analogs for comparing medians. Unless specified in the subsequent sections, comparisons will be two-sided with type I error rate of 0.05.

Missing data will be considered as "missing completely at random" provided missing data is modest (e.g., <10%). We will examine the "missing completely at random" assumption if missing data is more than 10%. If the assumption does not hold, missing data will be handled under the "missing at random" assumption (that is, missingness depends only on observed variables) via methods such as multiple imputation and inverse propensity weighting. To handle the possibility of "missing not at random," a sensitivity analysis will be performed by imputing missing binary observations with the observed proportion in the opposite arm. A secondary sensitivity analysis will be considered by imputing missing binary observations as failures.

Randomization: In the efficacy study, randomization will be 1:1:1 allocation to the 2 CIS43LS arms and the placebo arm. To limit the number of dropouts before vaccination, randomization and CIS43LS administration will occur as close in time as possible.

9.4.2 Analysis of the Primary Endpoints

Analysis for the primary endpoint for safety and tolerability is described in section 9.4.5.

Analysis for the primary efficacy endpoint, protective efficacy with Pf infection determined by blood smear, is described in section 9.4.4.

9.4.3 Analysis of the Secondary Endpoints

Analysis of the secondary efficacy endpoint, with Pf infection determined by RT-PCR, is described in section 9.4.4.

Analyses of the secondary endpoints for the PK of CIS43LS and the association of CIS43LS concentration with Pf infection risk are described in section 9.4.6.

9.4.4 Efficacy Analyses

The primary efficacy endpoint (efficacy study primary endpoint) is the incidence of malaria infection defined as blood smear–positive Pf infection through 24 weeks after administration. The secondary efficacy endpoint is the incidence of malaria infection as determined by RT-PCR. The efficacy analyses will be MITT.

The primary efficacy analysis will be based on time to the first infection. The survival patterns will be described by Kaplan-Meier curves for each arm and compared by the logrank test across different arms. The protective efficacy of the study product will be estimated by the hazard ratio from the Cox proportional hazards model. These analyses will be carried out by R packages that account for interval censoring: package "interval" for deriving a nonparametric maximum likelihood estimation based on the Kaplan-Meier survival curve and logrank test, and package "icenReg" for Cox proportional hazards regression. To address the heterogeneity of the study population, a Cox regression with regressors other than the study arm will be additionally performed to account for potential differences among participants. The regressors will include time of enrollment and possibly those covariates that are significantly different between the study product arms and the placebo arm in spite of randomization.

The secondary efficacy analysis will be based on the proportion of infection. The proportions of infection will be estimated for each arm and compared across arms based on Kaplan-Meier estimates along with 95% confidence intervals via R package "bpcp." This estimation accounts

for right censoring and the comparison should be equivalent to Fisher's exact test in case of no censoring. Though not accounting for interval censoring, the Kaplan-Meier estimates are appropriate as the interest is on infection at 24 weeks only.

The Holm method will be adopted to address the issue of multiplicity in comparing the 2 dose arms against the placebo.

The above analyses will apply to both the primary and the secondary efficacy endpoints.

9.4.5 Safety Analyses

Safety analysis will be primarily MITT where individuals who receive assignment but do not receive any product are excluded. Because of blinding and the brief length of time between assignment and administration, such cases will be very few.

Safety data will be presented by line listing and tables at the individual level to provide details on safety events such as severity, duration, and relationship to study product. The number and percentage of subjects with 1 or more AEs will be summarized by dose arm along with the exact 95% confidence intervals of the AE rate. For subjects experiencing more than 1 AE, the subjects will be counted once under the event of highest severity.

In the efficacy study, comparisons between the dose arms and the control arm will be additionally performed in terms of the proportions of solicited AEs, related AEs, and SAEs.

In the rare case of subjects receiving a regimen different from assignment, a per-protocol analysis will be performed as a secondary analysis, which will include subjects according to the product they actually receive in the study.

9.4.6 Pharmacokinetics Analysis

PK analysis will be carried out for subjects in the dose-escalation study and the efficacy study with blood samples collected at defined timepoints as listed in section 1.3. The following PK analysis will be performed as needed.

Individual Subject PK Analysis: A non-compartmental (NC) PK analysis will be performed on the CIS43LS concentration data generated from each subject. Individual subject and dosing arm concentration-versus-time profiles will be constructed in linear and semi-log scales. In the NC analysis, the maximum concentration (Cmax) and time of maximal concentration (Tmax) will be taken directly from the observed data. The area under the concentrations vs. time curve (AUC) will be calculated using the trapezoidal method and determined out to the final concentration collected. If a subject's CIS43LS concentration falls below the quantitative limit (QL) of the assay, the sample with concentration below the QL will be assigned a CIS43LS concentration value of "0" for AUC calculations. In addition to the total AUC, partial AUCs will also be determined over certain time intervals. The time-weighted average concentrations (Cave) during these intervals will be calculated as the AUC divided by the AUC collection interval (e.g., $Cave_{0\text{-}16WK} = (AUC_{0\text{-}16WK})$ / 16 weeks). The terminal slope, λ_z, will be determined by regression of the terminal, log-linear portion of the concentration-versus-time profile. If the final PK sample has measurable CIS43LS concentrations greater than the assay QL, the AUC post-final PK collection ($AUC_{last\text{-}infinity}$) will be estimated as C_{last} / λ_z and $AUC_{0\text{-}infinity}$ will be calculated as the sum of $AUC_{0\text{-}last} + AUC_{last\text{-}infinity}$.

Population PK Analyses: Based on preclinical PK results for CIS43LS and known PK behavior studies of MAbs, the two-compartment model will be used for population PK analysis. The population analysis will estimate compartmental PK parameters such as the clearance (CL), central and peripheral volumes of distribution (Vd1 and Vd2), and intercompartmental clearance (Q). Total volume of distribution at steady-state (Vdss), will be calculated as the sum of Vd1 + Vd2. Alpha and beta half-lives will be calculated from CL, Q, Vd1, and Vd2 using standard equations.[19]

To assess the association of CIS43LS concentration with protection, we will perform a Cox proportional hazards regression for the time to the first infection with CIS43LS concentration as a time-varying covariate. A logistic regression analysis will be additionally performed to model the infection rate as a function of CIS43LS concentration via the method of generalized estimating equation to account for repeated measures.

9.4.7 Baseline Descriptive Statistics

Treatment arms will be compared for baseline subject characteristics using descriptive statistics. For continuous variables, the mean or median will be calculated for each treatment arm. For categorial variables, the proportion under each category will be calculated for each arm.

9.4.8 Planned Interim Analyses

One interim safety analysis will be performed when safety data are available from the dose-escalation study subjects. The purpose of the interim analysis on safety data is to clear safety concerns for proceeding to the efficacy study. This interim analysis will not affect the power or type I error in the primary analysis on efficacy in the efficacy study.

9.4.9 Sub-group Analyses

Not applicable.

9.4.10 Tabulation of Individual Participant Data

Safety data will be presented by line listing and tables at the individual level, as described in section 9.4.5.

9.4.11 Exploratory Analyses

To explore the impact of CIS43LS on the genotype of infection-inducing parasites at the CSP locus, a genotypic sieve analysis will be performed to analyze CSP sequences of breakthrough parasites in the blood samples of infected subjects. The sieve analysis will differentiate protective efficacy against different genotypes of infection-inducing parasites with genotype defined by, for example, number of mismatches to the CIS43LS footprint.

10 REGULATORY AND OPERATIONAL CONSIDERATIONS

10.1 INFORMED CONSENT PROCESS

10.1.1 Consent Procedures and Documentation

The informed consent process for this study will involve obtaining initial community permission followed by individual informed consent.

10.1.1.1 Community Permission for the Conduct of the Study

Prior to the start of this study, community permission will be obtained as described in section 5.6. Following the process of Diallo and colleagues,[20] the community permission process will involve the following:

1. Study investigators/personnel explain the study to village leaders, including the village chief, family heads, women association, and elders.
2. The village leaders discuss the study with family heads and community members and relay any additional questions or concerns to the study personnel.
3. The study and the informed consent process are explained in detail to heads of families by study investigators/personnel.

Discussions during the community permission process will address the need for both a husband and wife to agree to avoid pregnancy for the specified period if a wife chooses to volunteer for the study.

10.1.1.2 Individual Informed Consent

The study informed consent form will be written in French. The study team will review the consent form word-for-word and will translate it orally into local languages, since most potential study subjects do not read or speak French. An independent witness who is not a member of the study team will verify that oral translations are accurate and that potential subjects understand the contents of the consent form.

Local households and families will be invited to come to the study clinic for review of the informed consent. At the consenting visit, the subject will read the consent form or have it explained to them (in cases of illiteracy). Individuals in each family will be separately consented, and not all individuals from a household need to participate. Individuals who agree to participate will sign or fingerprint (if illiterate) the consent form.

Also, a study comprehension examination will be conducted to make sure that the study is understood by the potential subjects prior to signing consent. The exam will be written in French and translated orally into local languages. All incorrect responses will be reviewed, and individuals must orally confirm their understanding of all incorrect responses. A score of at least 80% correct responses is mandatory to enroll. For individuals scoring below 80%, study staff may choose to review study details again and reassess comprehension by repeating the examination. At the discretion of the investigator, any individual whose comprehension is questionable, regardless of score, may be excluded from enrollment.

10.1.2 Consent for Minors When They Reach the Age of Majority

Not applicable.

10.1.3 Telephone Consent

Not applicable.

10.1.4 Telephone Assent

Not applicable.

10.1.5 Participation of Subjects who are/become Decisionally Impaired

Adults unable to give consent are excluded from enrolling in the protocol. However, reconsent may be necessary and there is a possibility, though unlikely, that subjects could become decisionally impaired. For this reason and because subjects might not benefit from research participation (section 2.3.3), subjects who lose the ability to consent during participation will be withdrawn.

10.2 Study Discontinuation and Closure

The study may be temporarily suspended or permanently terminated as described in the halting rules (section 8.4.6). In addition to the reporting described in that section, the principal investigator(s) will promptly contact the study subjects, provide the reason(s) for the termination or suspension, and, if applicable, inform them of changes to study visit schedule.

The principal investigators will consult with the EC prior to resuming the study following a halt.

10.3 Confidentiality and Privacy

All records will be kept confidential to the extent provided by federal, state, and local law. The study monitors and other authorized representatives of the sponsor may inspect all documents and records required to be maintained by the investigator, including but not limited to, medical records. Records will be kept locked and data will be coded. Any personally identifiable information maintained for this study will be kept on restricted-access computers and networks. Personally identifiable information will only be shared with individuals authorized to receive it under this protocol. Individuals not authorized to receive personally identifiable information will be provided with coded information only, as needed. Clinical information will not be released without written permission of the subject, except as necessary for monitoring by the FMPOS EC, FDA, NIAID, Office for Human Research Protections (OHRP), the VRC, or the sponsor's designee.

To further protect the privacy of study subjects, a Certificate of Confidentiality has been issued by the NIH. This certificate protects identifiable research information from forced disclosure. It allows the investigator and others who have access to research records to refuse to disclose identifying information on research participation in any civil, criminal, administrative, legislative, or other proceeding, whether at the federal, state, or local level. By protecting researchers and institutions from being compelled to disclose information that would identify

research subjects, Certificates of Confidentiality help achieve the research objectives and promote participation in studies by helping assure confidentiality and privacy to subjects.

Samples and data will be collected and stored under this protocol. All of the stored study research samples are labeled by a code that only the investigators can link to the subject. Samples are stored in secure research laboratories in locked freezers with limited access at the USTTB, Bamako, and the NIH. Data will be kept in password-protected computers. Only investigators or their designees will have access to the samples and data.

Samples and data acquired under this protocol will be tracked using BSI Systems software.

Any loss or unanticipated destruction of samples (for example, due to freezer malfunction) or data (for example, misplacing a printout of data with identifiers) that meets the definition of a reportable event will be reported to the FMPOS EC.

Additionally, subjects may decide at any point not to have their samples stored. In this case, the principal investigator will destroy all known remaining samples and report what was done to both the subject and to the EC. This decision will not affect the individual's participation in this protocol or any other protocols at NIH.

10.4 Future Use of Stored Specimens and Data

Subjects are consented at enrollment for permission to indefinite storage and future use of specimens and data. Samples, specimens, and data collected under this protocol may be used to study malaria and the immune system. Genetic testing may be performed.

Storage and Tracking: Access to and tracking of stored samples and data will be secured and limited as described above (section 10.3).

Disposition:

- In the future, other investigators (both at NIH and outside) may wish to use these samples and/or data for research purposes. If the planned research falls within the category of "human subjects research" on the part of the NIH researchers, EC review and approval will be obtained. This includes the NIH researchers sending out coded and linked samples or data and getting results that they can link back to their subjects.

10.5 Safety Oversight

Safety oversight is described in section 8.4.4.

10.6 Clinical Monitoring

According to the ICH E6(R2) GCP guidelines, section 5.18, and FDA 21 CFR 312.50, clinical protocols are required to be adequately monitored by the study sponsor. This study monitoring will be conducted according to the "NIAID Intramural Clinical Monitoring Guidelines." Monitors under contract to the NIAID/OCRPRO will visit the clinical research site to monitor aspects of the study in accordance with the appropriate regulations and the approved protocol. The objectives of a monitoring visit will be: 1) to verify the existence of signed informed consent documents and documentation of the consent process for each monitored subject; 2) to verify the prompt and accurate recording of all monitored data points in DFdiscover and prompt reporting

of all SAEs; 3) to compare abstracted information entered into DFdiscover with individual subjects' records and source documents (subjects' charts, laboratory analyses and test results, physicians' progress notes, nurses' notes, and any other relevant original subject information); and 4) to help ensure investigators are in compliance with the protocol. The monitors also will inspect the clinical site regulatory files to ensure that regulatory requirements (OHRP, FDA) and applicable guidelines (ICH GCP) are being followed. During the monitoring visits, the investigator (and/or designee) and other study personnel will be available to discuss the study progress and monitoring visit.

The investigator (and/or designee) will make study documents (e.g., consent forms, DFdiscover abstracts) and pertinent hospital or clinical records readily available for inspection by the local EC, FDA, the site monitors, and the NIAID staff for confirmation of the study data.

A specific protocol monitoring plan will be discussed with the principal investigator and study staff prior to enrollment. The plan will outline the frequency of monitoring visits based on such factors as study enrollment, data collection status, and regulatory obligations.

10.7 QUALITY ASSURANCE AND QUALITY CONTROL

During the study, the principal investigator and study team will be responsible for ensuring study activities are conducted in compliance with the protocol, ICH GCP, and applicable regulatory requirements. Each clinical site will perform internal quality management of study conduct, data and biological specimen collection, and documentation according to study standard operating procedures.

10.8 DATA HANDLING AND RECORD KEEPING

10.8.1 Data Collection and Management Responsibilities

Study data will be maintained in CRFs and collected directly from subjects during study visits and telephone calls or will be abstracted from subjects' medical records. Source documents include all recordings of observations or notations of clinical activities, including CRFs, and all reports and records necessary to confirm the data abstracted for this study. Data entry into CRFs will be performed by authorized individuals. The investigator is responsible for assuring that the data collected are complete, accurate, and recorded in a timely manner. Study data, including cumulative subject accrual numbers, should be generated via the chosen data capture method and submitted to the EC as needed.

10.8.2 Study Records Retention

The investigator is responsible for retaining all essential documents listed in the ICH GCP guidelines. Study records will be maintained by the principal investigator according to the timelines specified in 21 CFR 312.62 or a minimum of 7 years, and in compliance with institutional, EC, state, and federal medical records retention requirements, whichever is longest. All stored records will be kept confidential to the extent required by federal, state, and local law.

Should the investigator wish to assign the study records to another party and/or move them to another location, the investigator will provide written notification of such intent to OCRPRO/NIAID with the name of the person who will accept responsibility for the transferred

records and/or their new location. NIAID will be notified in writing and written OCRPRO/NIAID permission shall be obtained by the site prior to destruction or relocation of research records.

10.9 Protocol Deviations

It is the responsibility of the investigator to use continuous vigilance to identify and report deviations to the FMPOS EC according to NIH HRPP Policy 801 (as described in section 8.6.1). All deviations must be addressed in study source documents and reported to the NIAID Program Official and sponsor. The investigator is responsible for knowing and adhering to the reviewing EC requirements.

10.9.1 NIH Definition of Protocol Deviation

The definition of a protocol deviation is provided in section 8.4.1.

10.10 Publication and Data Sharing Policy

10.10.1 Human Data Sharing Plan

This study will be conducted in accordance with the following publication and data sharing policies and regulations:

- NIH Public Access Policy, which ensures that the public has access to the published results of NIH funded research. It requires scientists to submit final peer-reviewed journal manuscripts that arise from NIH funds to the digital archive PubMed Central upon acceptance for publication.
- This study will comply with the NIH Data Sharing Policy and Policy on the Dissemination of NIH-Funded Clinical Trial Information and the Clinical Trials Registration and Results Information Submission rule. As such, this trial will be registered at ClinicalTrials.gov, and results information from this trial will be submitted to ClinicalTrials.gov. In addition, every attempt will be made to publish results in peer-reviewed journals. Data from this study may be requested from other researchers indefinitely after the completion of the primary endpoint by contacting Peter Crompton or LIG.

Human data generated in this study for future research will be shared as follows:

- De-identified or identified data with approved outside collaborators under appropriate agreements.
- De-identified results or data in publication and/or public presentations.

Data will be shared at the time of publication or shortly thereafter.

10.10.2 Genomic Data Sharing Plan

Not applicable.

10.11 COLLABORATIVE AGREEMENTS

10.11.1 Agreement Type

Not applicable.

10.12 CONFLICT OF INTEREST POLICY

The independence of this study from any actual or perceived influence, such as by the pharmaceutical industry, is critical. Therefore, any actual conflict of interest of persons who have a role in the design, conduct, analysis, publication, or any aspect of this trial will be disclosed and managed. Furthermore, persons who have a perceived conflict of interest will be required to have such conflicts managed in a way that is appropriate to their participation in the design and conduct of this trial. The study leadership will follow policies and procedures for all study group members to disclose and manage all conflicts of interest and will establish a mechanism for the management of all reported dualities of interest.

11 ABBREVIATIONS

ADA	Anti-drug antibody
AE	Adverse event
ALT	Alanine transaminase
ANA	Anti-nuclear antibody
AR	Adverse reaction
AUC	Area under the curve
β-hCG	Beta-human choriogonadotropin
Cave	Time-weighted average concentrations
CBC	Complete blood count
CFA	Communauté Financière Africaine
CFR	Code of Federal Regulations
CHMI	Controlled human malaria infection
CIS43LS	VRC-MALMAB0100-00-AB
CL	Clearance
Cmax	Maximum concentration
CONSORT	Consolidated Standards of Reporting Trials
COVID-19	Coronavirus disease 2019
CPT	Cell preparation tube
Cr	Creatinine
CRF	Case report form
CRS	Cytokine release syndrome
CSO	Clinical Safety Office
CSP	Circumsporozoite protein
CTM	Clinical Trials Monitoring
DSMB	Data and safety monitoring board
EC	Ethics committee
ECG	Electrocardiogram

EDTA	Ethylenediaminetetraacetic acid
ELISA	Enzyme-linked immunosorbent assay
FDA	Food and Drug Administration
FMPOS	Faculté de Médecine Pharmacie d'Odontostomatologie
GCP	Good clinical practice
GLP	Good laboratory practices
GMP	Good manufacturing practices
HBV	Hepatitis B virus
HCV	Hepatitis C virus
HIV	Human immunodeficiency virus
HPF	High power field
HRPP	Human Research Protection Program
ICH	International Council on Harmonisation
Ig	Immunoglobulin
IND	Investigational new drug
ISM	Independent safety monitor
ITT	Intention to treat
IV	Intravenous(ly)
LIG	Laboratory of Immunogenetics
MAb	Monoclonal antibody
MITT	Modified intention to treat
MRTC	Malaria Research and Training Center
NC	Non-compartmental
NIAID	National Institute of Allergy and Infectious Diseases
NIH	National Institutes of Health
OCRPRO	Office of Clinical Research Policy and Regulatory Operations
OHRP	Office for Human Research Protections
OHSRP	Office of Human Subjects Research Protections
Pb	*Plasmodium berghei*
PBMC	Peripheral blood mononuclear cell
PCR	Polymerase chain reaction
Pf	*Plasmodium falciparum*
PK	Pharmacokinetics
Pr	Probability
Q	Intercompartmental clearance
QL	Quantitative limit
QTc	QT interval, corrected
RBC	Red blood cell
RDT	Rapid diagnostic test
REDCap	Research Electronic Data Capture
REF	Reportable Event Form
rPfCSP	Recombinant *Plasmodium falciparum* circumsporozoite protein
RT-PCR	Reverse transcription polymerase chain reaction
SAE	Serious adverse event

SAR	Suspected adverse reaction
SC	Subcutaneous(ly)
SERF	Safety Expedited Report Form
SMM	Sponsor medical monitor
SOA	Schedule of Activities
SOP	Standard operating procedure
SPZ	Sporozoite
SRCP	Safety review and communications plan
SST	Serum-separating tube
SUSAR	Serious and unexpected suspected adverse reaction
Tmax	Time of maximum concentration
UP	Unanticipated problem
UPnonAE	Unanticipated problem that is not an adverse event
US	United States
USTTB	University of Science, Techniques and Technologies of Bamako
VCMP	Vaccine Clinical Materials Program
Vd1, Vd2	Volumes of distribution
Vdss	Total volume of distribution at steady-state
VRC	Vaccine Research Center
WBC	White blood cell
WHO	World Health Organization

12 REFERENCES

1. WHO. World Malaria Report 2018. 2018.
2. Efficacy and safety of RTS,S/AS01 malaria vaccine with or without a booster dose in infants and children in Africa: final results of a phase 3, individually randomised, controlled trial. *Lancet.* 2015;386(9988):31-45.
3. Mahmoudi S, Keshavarz H. Efficacy of phase 3 trial of RTS, S/AS01 malaria vaccine: The need for an alternative development plan. *Hum Vaccin Immunother.* 2017;13(9):2098-2101.
4. Zalevsky J, Chamberlain AK, Horton HM, et al. Enhanced antibody half-life improves in vivo activity. *Nat Biotechnol.* 2010;28(2):157-159.
5. Ko SY, Pegu A, Rudicell RS, et al. Enhanced neonatal Fc receptor function improves protection against primate SHIV infection. *Nature.* 2014;514(7524):642-645.
6. Gaudinski MR, Coates EE, Houser KV, et al. Safety and pharmacokinetics of the Fc-modified HIV-1 human monoclonal antibody VRC01LS: A Phase 1 open-label clinical trial in healthy adults. *Plos Med.* 2018;15(1):e1002493.
7. Kisalu NK, Idris AH, Weidle C, et al. A human monoclonal antibody prevents malaria infection by targeting a new site of vulnerability on the parasite. *Nat Med.* 2018;24(4):408-416.

8. Ledgerwood JE, Coates EE, Yamshchikov G, et al. Safety, pharmacokinetics and neutralization of the broadly neutralizing HIV-1 human monoclonal antibody VRC01 in healthy adults. *Clin Exp Immunol.* 2015.

9. Gaudinski MR, Houser KV, Doria-Rose NA, et al. Safety and pharmacokinetics of broadly neutralising human monoclonal antibody VRC07-523LS in healthy adults: a phase 1 dose-escalation clinical trial. *Lancet HIV.* 2019;6(10):e667-e679.

10. Gaudinski MR, Coates EE, Novik L, et al. Safety, tolerability, pharmacokinetics, and immunogenicity of the therapeutic monoclonal antibody mAb114 targeting Ebola virus glycoprotein (VRC 608): an open-label phase 1 study. *Lancet.* 2019;393(10174):889-898.

11. US Food and Drug Administration. *Biomarker Qualification Decision Letter DDTBMQ000044, October 12 2018.* Silver Spring, MD: US Food and Drug Administration;2018.

12. Hansel TT, Kropshofer H, Singer T, Mitchell JA, George AJ. The safety and side effects of monoclonal antibodies. *Nat Rev Drug Discov.* 2010;9(4):325-338.

13. Bugelski PJ, Achuthanandam R, Capocasale RJ, Treacy G, Bouman-Thio E. Monoclonal antibody-induced cytokine-release syndrome. *Expert Rev Clin Immunol.* 2009;5(5):499-521.

14. Vogel WH. Infusion reactions: diagnosis, assessment, and management. *Clin J Oncol Nurs.* 2010;14(2):E10-21.

15. Drug allergy: an updated practice parameter. *Ann Allergy Asthma Immunol.* 2010;105(4):259-273.

16. Coartem (artemether and lumefantrine) tablets [package insert]. East Hanover, NJ: Novartis; 2019.

17. Tran TM, Li S, Doumbo S, et al. An intensive longitudinal cohort study of Malian children and adults reveals no evidence of acquired immunity to Plasmodium falciparum infection. *Clin Infect Dis.* 2013;57(1):40-47.

18. CIS43LS [investigator's brochure]. Bethesda, MD: NIAID Vaccine Research Center; 2019.

19. Gibaldi M, Perrier D. *Pharmacokinetics.* New York, NY: Marcel Dekker, Inc.; 1975.

20. Diallo DA, Doumbo OK, Plowe CV, Wellems TE, Emanuel EJ, Hurst SA. Community permission for medical research in developing countries. *Clin Infect Dis.* 2005;41(2):255-259.

21. Bain BJ. Ethnic and sex differences in the total and differential white cell count and platelet count. *J Clin Pathol.* 1996;49(8):664-666.

22. Haddy TB, Rana SR, Castro O. Benign ethnic neutropenia: what is a normal absolute neutrophil count? *J Lab Clin Med.* 1999;133(1):15-22.

APPENDIX A: MRTC URINE LABORATORY NORMAL VALUES

Urine Dip/Urinalysis

Urine[1]	Reference Ranges
Protein	None or Trace
Blood (Microscopic) –<br><br>RBC/HPF	None or Trace<br><br>< 5

Abbreviations: HPF, high power field; RBC, red blood cell.

[1] The laboratory values provided in the table are based on Bancoumana, Malian adults (age 18-45 years).

APPENDIX B: MALI ADVERSE EVENT GRADING SCALE

Evaluation	Mild (Grade 1)	Moderate (Grade 2)	Severe (Grade 3)	Potentially Life-threatening (Grade 4)
**Hematology and Biochemistry Values[1, 2]**				
**Hemoglobin (Female) – gm/dL**	8.0 – 9.0	7.0 – 7.9	6.0 – 6.9	< 6 and/or requiring transfusion
**Hemoglobin (Male) – gm/dL**	9.5 – 10.3	8.0 – 9.4	6.5 – 7.9	< 6.5 and/or requiring transfusion
**WBC Increase – 10^3/µL**	11.5 – 15.0	15.1 – 20.0	20.1 – 25.0	> 25.0
**WBC Decrease – 10^3/µL**	2.5 – 3.3	1.5 – 2.4	1.0 – 1.4	< 1.0 with fever
**Neutrophil/Granulocyte Decrease[3] – 10^3/µL**	0.80 – 1.00	0.50 – 0.79	< 0.50	< 0.50 with fever
**Platelet Decrease – 10^3/µL**	100 – 110	70 – 99	25 – 69	< 25
**Creatinine (Male) – µmol/L**	124.00 – 150.99	151.00 – 176.99	177.00 – 221.00	> 221.00 and requires dialysis
**Creatinine (Female) – µmol/L**	107.00 – 132.99	133.00 – 159.99	160.00 – 215.99	> 216.00 and requires dialysis
**Liver Function Tests/ALT – U/L**	75.0 – 150.9	151.0 – 300.9	301.0 – 600.0	> 600.0
**Other Values**				
**Fever[4] – °C**	37.5 – 37.9	38.0 – 38.4	38.5 – 39.5	> 39.5

Abbreviations: ALT, alanine transaminase; WBC, white blood cell.

[1] The laboratory values provided in the tables serve as guidelines and are dependent upon institutional normal parameters. Institutional normal reference ranges should be provided to demonstrate that they are appropriate.

[2] The clinical signs or symptoms associated with laboratory abnormalities might result in characterization of the laboratory abnormalities as potentially life-threatening (grade 4). For example, a low sodium value that falls within a grade 3 parameter should be recorded as a grade 4 hyponatremia event if the subject had a new seizure associated with the low sodium value.

[3] Note: Neutropenias are graded and followed, but based on previous experience in African populations, should be interpreted with caution since lower values are more frequently observed in people of African descent. [21,22]

[4] Values presented are for non-oral temperature reading (i.e., axillary or no-touch), which is the preferred method at the study site. If oral temperature is taken, the US Food and Drug Administration toxicity grading scale will be used to grade fever.

APPENDIX C: DOSE ESCALATION STUDY EXTENSION

Study Extension Rationale

Subjects who completed the Dose Escalation study may be followed with additional study visits through the end of the malaria season to 1) determine whether they become infected with *P. falciparum* (as detected by RT-PCR from dried blood spots collected every 2 weeks), and 2) to extend the PK analysis of CIS43LS to approximately 9 months (as determined by measuring the concentration of CIS43LS in blood samples collected every 4 weeks). The Dose Escalation component of the study had 3 dose groups (5 mg/kg, 10 mg/kg, and 40 mg/kg of CIS43LS), while the Efficacy component of the study only had 2 CIS43LS dose groups (10 mg/kg and 40 mg/kg). Following all subjects in the Dose Escalation group (including those who received 5 mg/kg of CIS43LS) will provide insight into the potential protective efficacy of a broader range of CIS43LS doses. Extending the PK analysis of subjects in the Dose Escalation group out to a total of 9 months will provide valuable information on the PK of CIS43LS in all 3 dose groups (5 mg/kg, 10 mg/kg, and 40 mg/kg) and will allow for exploratory analyses that assess the correlation between CIS43LS concentrations in blood and *P. falciparum* infection risk over a 9-month period.

Study Extension Procedures

Participants who completed the Dose Escalation study will be offered participation in the extension study visits and undergo an informed consent process (see section 10.1). Those who agree to participate will sign the extension consent and have a screening hemoglobin test. Those with hemoglobin <7 g/dL (<8 g/dL for pregnant women) will be excluded from participating in the Extension visits; those with hemoglobin ≥7 g/dL (≥8 g/dL for pregnant women) will be enrolled and begin bi-weekly Extension study visits at the MRTC clinic in Kalifabougou. At each study visit, up to 4.5 mL of blood will be collected by fingerprick or venipuncture for blood smear, Pf RT-PCR, and PK studies, as well as possible parasite genotyping in the case of a positive malaria parasite infection (see section 8.2.3). If a participant develops symptoms of malaria or other symptoms, they will be asked to return for an illness visit (see section 8.2.1). During the final extension visit, an additional 8mL CPT tube will be collected for PBMC storage, unless the subject declines.

AEs related to the blood collection procedures and all SAEs will be collected and followed through resolution.

Participants will attend bi-weekly study visits through a common end date of December 31, 2021, at which point all participants will be completed with study participation.

Compensation

Subjects will be compensated 3,000 Communauté Financière Africaine (CFA) Franc for each study visit for the time and inconvenience of participation. Payment will be provided in cash after the completion of each visit.

Subjects will be provided with transportation to and from study visits but will not receive additional reimbursement for travel.

Additional Procedures and Processes

Refer to main protocol sections 8-10 for additional information related to safety definitions and reporting, data evaluation, and human subject protection procedures.

APPENDIX D: PREGNANCY FOLLOW-UP

Pregnancy Follow-up Rationale

If a woman becomes pregnant during study participation, we will follow her for safety for the duration of the pregnancy and for 12 months following delivery for assessment of the neonate. In addition to the procedures described in section 8.4.2.3.4, we will perform additional research procedures to collect information regarding the delivery outcome and the health of the neonate/infant and mother over the months following delivery. This will allow an initial assessment of PK values after delivery as well as the effects of the investigational agent on pregnancy course and outcome, and infant health.

Pregnancy Follow-up Procedures

Participants who become pregnant during the study will be offered participation in the pregnancy follow-up phase and undergo an informed consent process (see section 10.1). Women who agree to participate will sign the pregnancy follow-up consent and will undergo the following assessments at the time of delivery:

- Hemoglobin measure using HemoCue
- Peripheral malaria test (RDT, blood smear, filter paper)
- Placental malaria using blood smear, filter paper
- Placental malaria histology
- PK blood draw
- AE assessment
- Breast milk to measure CIS43LS concentrations

After delivery, we will collect a breast milk sample at each of the scheduled infant visits described below.

At the infant follow-up visits during months 6, 9, and 12, 4 mL of blood will also be collected from the mother for detection of malaria and for PK analysis.

The neonate/infant and mother will undergo research assessments according to Table 6.

Table 6. Schedule of assessments for mother and neonate/infant participants.

**Study Month**	**Month 0/ Delivery**	3	6	9	12	**Illness Visit**	**ET Visit**
Window (days)	+7	±7	±7	±7	±7		
**Clinical Procedures/Evaluations**							
Heel/finger stick (infant)	X	X	X	X	X	X	X
Cord blood collection	X						

**Study Month**		**Month 0/ Delivery**	3	6	9	12	**Illness Visit**	**ET Visit**
Window (days)		+7	±7	±7	±7	±7		
Weight (infant)		X	X	X	X	X	X	X
Ballard assessment (infant)		X						
Apgar (infant)		X						
Length (infant)		X	X	X	X	X	X	X
Head circumference (infant)		X	X	X	X	X	X	X
Neurological assessment (infant)			X	X	X	X	X	X
Vaccination/EPI review [a] (infant)			X	X	X	X	X	X
AE assessment (mother and infant)		X	X	X	X	X	X	X
Breast milk sample (collected from mother)		X	X	X	X	X		X
Venous blood (collected from mother)				4 mL	4 mL	4 mL	4 mL	4 mL
**Laboratory Evaluations**								
Test	Tube	(infant blood volume)						
PK (heel/ finger stick)	SST	50 uL	50 uL	50 uL	50 uL	50 uL		50 uL
PK (cord blood)	SST	X						
Malaria blood smear/RDT [b]	N/A	(X)	(X)	(X)	(X)	(X)	3 to 4 drops	(X)
Hemoglobin	N/A		(X)	(X)	(X)	(X)	(X)	(X)

Abbreviations: AE, adverse event; ET, early termination; EPI, Expanded Programme of Immunization; PK, pharmacokinetics; RDT, rapid diagnostic test.

(X) indicates that no additional blood will be drawn; the test will be performed from blood collected for another evaluation listed.

[a] Record vaccination information from EPI card.

[b] Only performed if enough blood is available.

The month 12 visit will be the final study visit for participants; participation will be complete after this timepoint.

For both women and children, any AEs related to the blood collection procedures and all SAEs will be followed through resolution.

Compensation

Subjects will be compensated 3,000 Communauté Financière Africaine (CFA) Franc for each study visit for the time and inconvenience of participation. Mothers and neonates/newborns will each receive compensation for study visits. Payment will be provided in cash after the completion of each visit. The parent/guardian will receive the payments for the neonate/infant participants.

Subjects will be provided with transportation to and from study visits but will not receive additional reimbursement for travel.

Additional Procedures and Processes

Refer to main protocol sections 2.3.1, 8, and 10 for additional information related to risks, safety definitions and reporting, and human subject protection procedures.

PROTOCOL TRACKING LOG

Protocol Title: Safety and Efficacy of VRC-MALMAB0100-00-AB (CIS43LS), a Human Monoclonal Antibody against *Plasmodium falciparum*, in a Dose-Escalation Trial and a Randomized, Double-Blind Trial of Adults in Mali
Abbreviated Title: Anti-malaria MAb in Mali
FMPOS Protocol #: 2020/32/CE/FMOS/FAPH
FMPOS PI: Kassoum Kayentao, MD, MPH, PhD
NIH PI: Peter D. Crompton, MD, MPH

INITIAL APPROVAL Version 21 February 2020

AMENDMENT #1 Version 01 September 2020

1-Sep-2020	1-Sep-2020	Protocol<br>Consent Dose Escalation Study<br>Consent Efficacy Study	8-Sep-2020	N/A	N/A	20-Oct-2020	✓	

Summary of Changes:
1.Added FMPOS Protocol #: 2020/32/CE/FMOS/FAPH in the protocol and consent forms.
2.Added FDA IND #: 147485.
3.Added Study Coordinator Anne Preston, RN.
4.Added Associate Investigator Dr. Sean Murphy whose lab at the University of Washington will perform Pf RT-PCR. Throughout the protocol, all instances of Pf "PCR" were changed to Pf "RT-PCR" to more accurately reflect the assay conducted by Dr. Murphy. In addition, Sections 2.2.3.3 and 8.2.3 now indicate that Dr. Murphy will conduct the RT-PCR assay. Accordingly, the Study Staff Roster subheading was revised to "US Investigators" since Dr. Murphy (and Dr. Neafsey, mentioned below) is not affiliated with the NIH.
5.Added Associate Investigator Dr. Daniel Neafsey whose lab at Harvard University will perform Pf genotyping, which is now indicated in Sections 2.2.3.3 and 8.2.3.
6.As per DSMB recommendations, amended Section 8.4.4.3.1 to accurately reflect the role of the Independent Safety Monitor (ISM); we also listed the ISM in the study roster.
7.As per DSMB recommendations, amended Section 9.3 to clarify language concerning "per protocol analysis"; also, amended Section 9.4.1 to clarify language about sensitivity analyses.
8.Added a summary of interim phase 1 safety data to the consent forms and protocol Sections 2.2.2.1; 2.3.1; 4.1; and 4.3.
9.Added reporting requirements for protocol deviations that result from the COVID-19 pandemic (Section 8.6.3).
10.Added text "or designee" to allow PI designee to also assess Causality for AEs (Section 8.4.2.1.2).
11.Updated protocol to list specific reactogenicity events to be evaluated after study infusion (Sections 1.3.1, 1.3.2, and 6.1.2.4), and updated risk language to include all reactogenicity symptoms that will be assessed in the consents (Key Information and Risks and Discomforts) and the protocol (section 2.3.1).
12.Revised protocol Section 6.1.2.4 to remove language suggesting that overnight inpatient stays are planned as part of this protocol.
13.Revised Section 8.4.2.1.1 Severity Grading, revised Appendix A, and added Appendix B to update AE grading for fever and abnormal blood laboratory values due to the normal ranges of these values in the study population (healthy adults in Mali).

AMENDMENT #2 Version 04 January 2021
Summary of Changes to Protocol:<br>• In the study staff roster, moved the information on the independent safety monitor, per recommendations from the study monitoring team.<br>• In section 1.3, added a clarification to the footnote on artemether-lumefantrine administration per recommendations from the study monitoring team.<br>• Updated the protocol with recent data from the ongoing VRC 612 phase 1 protocol (edits to section 2.2.2.1, 2.3.1).<br>• For the dose-escalation study, revised the 20-mg/kg arm to 10 mg/kg based on phase 1 data and consistency with updates to the efficacy study. Edits were made to the following sections: Section 1.1 Synopsis (objectives and description of study intervention), Section 1.2 Schema (figure 1), Section 3 Objectives and Endpoints, Section 6.1.2 Dosing and Administration (table 1),<br>• For the efficacy study, added a third treatment arm at 10 mg/kg CIS43LS by intravenous infusion.<br>Edits were made to the following sections: Section 1.1 Synopsis (study description, objectives, and description of study intervention), Section 1.2 Schema (figure 2), Section 3 Objectives and Endpoints, Section 4.1 Overall Design, Section 4.3 Justification for Dose, Section 5.6 Strategies for Recruitment and Retention, Section 6.1.2 Dosing and Administration (table 1), Section 6.3.1 Randomization, Section 9 Statistical Considerations.<br>• Updated study duration due to increased time for recruitment in the efficacy portion of the study (edits to section 1.1 Synopsis and 5.6 Strategies for Recruitment and Retention).<br>• In section 10.4, deleted language about samples being transferred to a repository after study completion, as this is no longer required at the NIH.<br>• In section 10.11.1, corrected an error by adding the phrase “Not applicable” to an empty protocol subsection.<br>• Corrected a typo in the phrase “muscle aches” in sections 1.3 and 6.1.2.4.<br>• Corrected abbreviation of “PK” for consistency throughout the protocol.<br>• Deleted typo in section 7.1.<br>• Revised language related to screening to clarify that transient/modifiable screening evaluations may be repeated at the investigator’s discretion (edits to sections 5.5, 8.1, and 8.4.3.1).<br>Summary of Changes to Dose-escalation consent:<br>• Added background and risk language based on new data from the ongoing VRC 612 protocol (under “Background/Purpose of Study” and “Risks/Discomforts”).<br>• In “Background/Purpose of Study,” revised mention of the efficacy study testing one dose of study drug, since it will now test 2 doses.<br>• In “Risks/Discomforts,” added an explanation about artemether-lumefantrine and the electrocardiogram, per recommendations from the sponsor medical monitor.<br>Summary of Changes to Efficacy consent:<br>• Added a third treatment arm at 10 mg/kg CIS43LS by intravenous infusion.<br>Edits were made to the following sections: Key Information (updated sample size and descriptions of study treatment arms), Background/Purpose of the Study (updated sample size and description of study treatment arms), “Study Treatment” (updated description of study treatment arms), “Risks of IV Administration” (clarified a side effect that
**AMENDMENT #3 Version 02 February 2021**
<u>Summary of Changes:</u><br><u>Protocol:</u><br>•Updated version date (edits to the cover page and header).<br>•Updated compensation section 5.6.2 to increase compensation at screening and enrollment visits to 5,000 CFA, which also increased the total possible compensation for each study (dose-escalation and efficacy).<br><u>Dose-escalation consent:</u><br>•Updated version date (edit to the footer).<br>•Updated compensation section to increase compensation amount at screening and enrollment visits to 5,000 CFA, which also increased the total possible compensation.<br><u>Efficacy consent:</u><br>•Updated version date (edit to the footer).<br>•Updated compensation section to increase compensation amount at screening and enrollment visits to 5,000 CFA, which also increased the total possible compensation.

AMENDMENT #4 Version 02 March 2021

Summary of Changes:

Protocol:

•Updated version date (edits to the cover page and header).

•Updated Section 1.3 Schedule of Activities: For the Dose-scalation study (1.3.1), added Finger prick blood smear and dried blood spot for Pf RT-PCR to the Day 21 visit.

AMENDMENT #5 Version 22 April 2021

Summary of Changes:

Protocol:

• Updated version date (edits to the cover page and header).

• Updated Section 8.4.2.3.4 Pregnancy to allow for research sample collections to continue for volunteers that become pregnant during the study.

Dose-escalation consent:

• Updated version date (edit to the footer).

• Updated risks/discomforts section (Risks of Pregnancy or Breastfeeding During Study Participation) to remove text stating research samples would not be drawn on volunteers that become pregnant.

Efficacy consent:

• Updated version date (edit to the footer).

• Updated risks/discomforts section (Risks of Pregnancy or Breastfeeding During Study Participation) to remove text stating research samples would not be drawn on volunteers that become pregnant.

AMENDMENT #6 Version 04 June 2021

Summary of Changes:

Protocol:

• Updated version date (edits to the cover page and header).

• Updated Description of Sites/Facilities Enrolling Subjects to specify that subjects can be seen at either Kalifabougou or Torodo clinic for most visits except screening, day 0 and day 7.

• Updated Section 1.3 Schedule of Activities in Notes area to specify that visits can take place at Kalifabougou or Torodo for most visits except screening, day 0 and day 7.

• Updated Section 4.1 Overall Design to specify that subjects can be seen at either Kalifabougou or Torodo clinic for most visits except screening, day 0 and day 7.

Dose-escalation consent:

• NO UPDATES

Efficacy consent:

• Updated version date (edit to the footer).

• Updated Key Information, Schedule section to specify that most visits can occur at either Kalifabougou or Torodo clinic.

• Updated Procedures Explained section to specify that most visits can occur at either Kalifabougou or Torodo clinic except screening, study treatment and one follow up that will occur only in Kalifabougou.

AMENDMENT #7 Version 14 June 2021

Summary of Changes:

Protocol:

- Updated version date (edits to the cover page and header).
- Updated Section 1.1 Synopsis, study population to allow enrollment of subjects up to 55 years old (increased from 50 years old).
- Updated Section 5.1 Inclusion Criteria to allow enrollment of subjects up to age 55 years old (increased from 50 years old).
- Updated compensation section 5.6.2 to increase compensation at non-venipuncture visits from 1,500 CFA to 3,000 CFA (same as venipuncture visits). This also increased the total possible compensation for each study (dose-escalation and efficacy).

Dose-escalation consent:

- Updated version date (edit to the footer).
- Updated compensation section to increase compensation amount at non-venipuncture visits from 1,500 CFA to 3,000 CFA (same as venipuncture visits). This also increased the total possible compensation.

Efficacy consent:

- Updated version date (edit to the footer).
- Updated compensation section to increase compensation amount at non-venipuncture visits from 1,500 CFA to 3,000 CFA (same as venipuncture visits). This also increased

[illegible] possible compensation

AMENDMENT #8 Version 21 September 2021

Summary of Changes:

Protocol:

•Updated version date (edits to the cover page and header).

•Updated Section 1.1 Study Description for the Dose-escalation study adding the extension visits and Subject Duration for Dose-escalation study.

•Updated Section 1.3 Schedule of Activities to allow fingerprick or venipuncture for blood smear and PCR collection. Also increased blood collection volume on visits that previously only had a finger prick. This applies to both dose escalation and efficacy studies. Also added a note on the dose escalation table about the extension study.

•Updated Section 2.2.3.3 Measure of Mab-Mediated Protection and Parasitemia to allow for a small venipuncture sample for the thick blood smears.

•Updated Section 4.1 Overall design to add information about the dose escalation extension.

•Updated Section 5.6.2 to note the additional compensation that will be received for those that participate in the extension study.

•Updated Section 8.2.1 Illness Visit to add the option of venipuncture for blood smear.

•Updated Section 8.2.2 Biospecimen Evaluations to add blood smear and dried blood spot to the venipuncture samples.

•Updated Section 8.4.2.3.4 Pregnancy to include the extension study visits for sample collection and to allow for venipuncture for dried blood spot collection.

•Added APPENDIX C: Dose Escalation Study Extension which describes the rationale, procedures, and compensation for the extension visits.

AMENDMENT #9 Version 07 October 2021

Summary of Changes:

Protocol:

•Updated version date (edits to the cover page and header).

•Updated Section 8.2.1 Illness Visit to clarify that procedures may be conducted rather than will be conducted.

Dose Escalation Extension consent:

•Updated version date (edit to the footer).

•Updated text for Illness Visits to clarify that procedures may be conducted rather than will be conducted.

Efficacy consent:

•Updated version date (edit to the footer).

•Updated text for Illness Visits to clarify that procedures may be conducted rather than will be conducted.

AMENDMENT #10 Version 09 November 2021

Protocol:

•Updated version date (edits to the cover page and header).

•Updated Section 1.3.2 Efficacy Study Schedule of activities to add optional PBMC Storage (8mL) to final visit.

•Updated Appendix C, Study Extension Procedures to add optional PBMC storage (8mL) to final visit.

Extension consent addendum:

•New Informed Consent addendum to the Dose Escalation Study Extension consent for additional PBMC storage (8mL CPT tube), which is optional.

Efficacy consent addendum:

•New Informed Consent addendum to the Efficacy consent for additional PBMC storage (8mL CPT tube), which is optional.

AMENDMENT #11 Version 06 June 2022

Protocol:

•Updated version date (edits to the cover page and header).

•Removed NIH Employee Associate Investigator Martin Gaudinski, MD. He is no longer working for the NIH.

•Updated Section 8.4.2.3.4 Pregnancy changing 6 months follow up period to 12 month follow up period for the assessment of the neonate.

•Added Appendix D: Pregnancy follow up section that describes the pregnancy follow-up rationale, pregnancy follow-up procedures including the compensation to the volunteer and to the baby.

New Pregnancy Follow-up Consent to Participate:

•New Informed Consent document for the pregnant volunteer to participate in additional visits and assessments as well as consent for her baby to participate for up to 12 months after the baby is born.

AMENDMENT #12 Version 13 October 2022

Protocol:

•Updated version date (edits to the cover page and header).
•Updated section 2.2.2.1 Previous Human Experience to note that the VRC 612 study has completed.
•Updated section 2.3.1 Known Potential Risks to clarify Risks of Blood Drawing or IV Placement section adding “via venipuncture or heel/finger sick”.
•Updated Appendix D Pregnancy Follow-Up
oAdded text noting the overall health of the mother will also be followed.
oAdded blood draw for the mother at months 6, 9 and 12 following the birth of the infant for detection of malaria and PK analysis. Updates to text and to Table 6 with these changes.
oRemoved option for Ear stick from infant for blood draw due to increased risks from this method.
oUpdated text for Additional Procedures and Processes with hyperlinks to main protocol sections and included the word “risks” in the text.

Pregnancy Follow-up Consent to Participate:

•Updated version date in footer
•Updated Key Information with “and your baby” for clarification.
•Updated General Information with “and your baby” for clarification.
•Updated Procedures Explained to remove “ear” as option for blood collection and clarified that the participant and her baby would come back for the follow up visits.
•Updated Procedures Explained, Study Visits to add sample collection from the participant as well as the infant.
•Updated Procedures Explained, Illness Visits to add the option of heel stick.

Summary of amendments to "Section 9: Statistical Considerations"

1. Per the initial recommendations of the DSMB, Section 9.3 was amended to clarify language concerning "per protocol analysis"; and Section 9.4.1 was amended to clarify language concerning “sensitivity analyses” (Amendment #1, 01 September 2020). This amendment preceded the initiation of the study in February 2021.

2. Based on CIS43LS PK data that became available from the phase 1 study in U.S. adults (Gaudinski et al. N Engl J Med 2021; 385:803-814), a third study arm, 10 mg/kg IV of CIS43LS, was added to the protocol that originally included only two study arms: placebo and 40 mg/kg IV of CIS43LS. This amendment to the study design, that preceded the initiation of the study in February 2021, resulted in the following changes to “Section 9: Statistical Considerations” (Amendment #2, 04 January 2021):

(a) Randomization was changed from 1:1 to 1:1:1.
(b) The sample size was changed from 90 per arm to 110 per arm as the two CIS43LS arms (10 mg/kg IV and 40 mg/kg IV) were each compared against the placebo arm at a type I error rate of 0.025 (changed from 0.05).
(c) The Holm method was added to address the issue of multiplicity in comparing the two CIS43LS arms against the placebo arm.
