# Supplementary material for: Anti-sporozoite monoclonal antibody for malaria prevention: secondary efficacy outcome of a phase 2 randomized trial
Source: Nat Med. 2025 Jun 3;31(8):2682–90. doi: 10.1038/s41591-025-03739-y (PMC12353790; doi:10.1038/s41591-025-03739-y)
Supplement: Supplementary file 2 — Reporting Summary [file 41591_2025_3739_MOESM2_ESM.pdf]

nature portfolio

Corresponding author(s): Peter D. Crompton

Last updated by author(s): Apr 21, 2025

Reporting Summary

Nature Portfolio wishes to improve the reproducibility of the work that we publish. This form provides structure for consistency and transparency in reporting. For further information on Nature Portfolio policies, see our Editorial Policies and the Editorial Policy Checklist.

Statistics

For all statistical analyses, confirm that the following items are present in the figure legend, table legend, main text, or Methods section.

n/a	Confirmed	
☐	☒	The exact sample size (*n*) for each experimental group/condition, given as a discrete number and unit of measurement
☒	☐	A statement on whether measurements were taken from distinct samples or whether the same sample was measured repeatedly
☐	☒	The statistical test(s) used AND whether they are one- or two-sided<br>*Only common tests should be described solely by name; describe more complex techniques in the Methods section.*
☐	☒	A description of all covariates tested
☐	☒	A description of any assumptions or corrections, such as tests of normality and adjustment for multiple comparisons
☐	☒	A full description of the statistical parameters including central tendency (e.g. means) or other basic estimates (e.g. regression coefficient) AND variation (e.g. standard deviation) or associated estimates of uncertainty (e.g. confidence intervals)
☐	☒	For null hypothesis testing, the test statistic (e.g. *F*, *t*, *r*) with confidence intervals, effect sizes, degrees of freedom and *P* value noted<br>*Give P values as exact values whenever suitable.*
☒	☐	For Bayesian analysis, information on the choice of priors and Markov chain Monte Carlo settings
☒	☐	For hierarchical and complex designs, identification of the appropriate level for tests and full reporting of outcomes
☐	☒	Estimates of effect sizes (e.g. Cohen's *d*, Pearson's *r*), indicating how they were calculated

Our web collection on statistics for biologists contains articles on many of the points above.

Software and code

Policy information about availability of computer code

Data collection	DFexplore 2023 (version 5.7.0) secures data with AES 256 encryption and is fully compliant with HIPAA, GDPR, FDA 21 CFR Part 11 regulations and is ISO 9001:2015 certified.
Data analysis	Analyses were performed with JMP version 16.2.0 and RStudio version 2024.04.1, and the R packages “icenReg” (version 2.0.16) and “bpcp” (version1.4.2) were used for time-to-event and proportional analyses, respectively. Parasite genotype analysis used the "stringr" package (version 1.5.1), the "DADA2" algorithm (version 1.14.0), and the "scikit-allel" package (version 1.2.0).

For manuscripts utilizing custom algorithms or software that are central to the research but not yet described in published literature, software must be made available to editors and reviewers. We strongly encourage code deposition in a community repository (e.g. GitHub). See the Nature Portfolio guidelines for submitting code & software for further information.

Data

Policy information about availability of data

All manuscripts must include a data availability statement. This statement should provide the following information, where applicable:

- Accession codes, unique identifiers, or web links for publicly available datasets
- A description of any restrictions on data availability
- For clinical datasets or third party data, please ensure that the statement adheres to our policy

Requests for access to the individual de-identified trial participant data set (including data dictionaries) can be submitted to corresponding author P.D.C. Upon

reasonable request, the corresponding author will respond within 2 weeks. In addition, reported results from this trial are publicly available at: https://clinicaltrials.gov/study/NCT04329104?intr=cis43ls&rank=1. Supporting clinical documents (protocol and statistical analysis plan) are provided as Supplementary Information.

Research involving human participants, their data, or biological material

Policy information about studies with human participants or human data. See also policy information about sex, gender (identity/presentation), and sexual orientation and race, ethnicity and racism.

Reporting on sex and gender	Sex was determined based on self reporting. Gender data was not collected. Sex is reported for each of the three trial arms in Table 1, but was not included as a covariate in the pre-specified efficacy analysis since sex ratios were evenly distributed across the three arms of this randomized trial. A post hoc sex-stratified analysis of 18S qRT-PCR-defined efficacy is reported in the manuscript.
Reporting on race, ethnicity, or other socially relevant groupings	This trial was conducted in two rural communities of Mali where all participants are of the African/black race. Ethnicity was determined based on self-reporting. We reported ethnicity for each of the three trial arms in Table 1, but it was not included as a covariate in the analysis because most participants were of a single ethnicity and other ethnicities were evenly distributed across arms of this randomized trial.
Population characteristics	The covariate-relevant population characteristics of research participants are reported in Table 1 and include age, sex, ethnicity, weight, study site, P. falciparum infection at baseline, and hemoglobin genotype. These characteristics were evenly distributed across the three arms of this randomized trial.
Recruitment	Community meetings were held at study sites to explain the study to village elders and community members. An announcement was made via local radio inviting households to come to participating clinics to learn about the study. Recruitment was not limited based on sex, race, or ethnicity. By design the trial only included healthy adults, so the efficacy of CIS43LS in unhealthy populations is unknown.
Ethics oversight	As indicated in the manuscript, the study protocol was approved by the ethics committee at Faculté de Médecine et d'Odonto-Stomatologie and Faculté de Pharmacie at the University of Sciences, Techniques, and Technologies of Bamako.

Note that full information on the approval of the study protocol must also be provided in the manuscript.

Field-specific reporting

Please select the one below that is the best fit for your research. If you are not sure, read the appropriate sections before making your selection.

☒ Life sciences ☐ Behavioural & social sciences ☐ Ecological, evolutionary & environmental sciences

For a reference copy of the document with all sections, see nature.com/documents/nr-reporting-summary-flat.pdf

Life sciences study design

All studies must disclose on these points even when the disclosure is negative.

Sample size	With 110 subjects in each of the 3 trial arms, the trial had at least 80% power in each comparison to claim protective efficacy of CIS43LS if the underlying efficacy was greater than or equal to 0.5 and the infection rate under placebo was no less than 0.4.
Data exclusions	No data were excluded from the analyses.
Replication	There was no formal replicate testing. All dried blood spot samples were tested in singlet for the 18S qRT-PCR endpoint included in the manuscript.
Randomization	The 330 participants were randomly assigned (in a 1:1:1 ratio) by block randomization to receive 10 mg/kg of CIS43LS, 40 mg/kg of CIS43LS, or placebo (110 participants in each group).
Blinding	The investigators were blinded to group allocation during data collection and analysis.

Reporting for specific materials, systems and methods

We require information from authors about some types of materials, experimental systems and methods used in many studies. Here, indicate whether each material, system or method listed is relevant to your study. If you are not sure if a list item applies to your research, read the appropriate section before selecting a response.

Materials & experimental systems

n/a	Involved in the study	
☒	☐	Antibodies
☒	☐	Eukaryotic cell lines
☒	☐	Palaeontology and archaeology
☒	☐	Animals and other organisms
☐	☒	Clinical data
☒	☐	Dual use research of concern
☒	☐	Plants

Methods

n/a	Involved in the study	
☒	☐	ChIP-seq
☒	☐	Flow cytometry
☒	☐	MRI-based neuroimaging

Clinical data

Policy information about clinical studies

All manuscripts should comply with the ICMJE guidelines for publication of clinical research and a completed CONSORT checklist must be included with all submissions.

Clinical trial registration	NCT04329104
Study protocol	The original and final versions of the protocol along with a summary of all amendments are included as Supplementary Information.
Data collection	All recruitment and clinical data/biospecimen collection occurred at the Kalifabougou, Mali study site from 15 Feb 2021 to.24 Jan 2022.
Outcomes	The pre-defined primary outcome measure was P. falciparum infection assessed by microscopic examination of thick blood smears. The pre-defined secondary outcome measure was P. falciparum infection assessed by 18S rRNA quantitative reverse transcription PCR.

Plants

Seed stocks	*Report on the source of all seed stocks or other plant material used. If applicable, state the seed stock centre and catalogue number. If plant specimens were collected from the field, describe the collection location, date and sampling procedures.*
Novel plant genotypes	*Describe the methods by which all novel plant genotypes were produced. This includes those generated by transgenic approaches, gene editing, chemical/radiation-based mutagenesis and hybridization. For transgenic lines, describe the transformation method, the number of independent lines analyzed and the generation upon which experiments were performed. For gene-edited lines, describe the editor used, the endogenous sequence targeted for editing, the targeting guide RNA sequence (if applicable) and how the editor was applied.*
Authentication	*Describe any authentication procedures for each seed stock used or novel genotype generated. Describe any experiments used to assess the effect of a mutation and, where applicable, how potential secondary effects (e.g. second site T-DNA insertions, mosiacism, off-target gene editing) were examined.*
